# Supplementary material for: An exceptionally flexible hydrogen-bonded organic framework with large-scale void regulation and adaptive guest accommodation abilities
Source: Nat Commun. 2019 Jul 12;10:3074. doi: 10.1038/s41467-019-10575-5 (PMC6625987; doi:10.1038/s41467-019-10575-5)
Supplement: Supplementary file 1 — Supplementary Information [file 41467_2019_10575_MOESM1_ESM.pdf]

Supplementary Information

An exceptionally flexible hydrogen-bonded organic framework with large-scale void regulation and adaptive guest accommodation abilities

Huang et al.

Supplementary Methods

Bis(4-bromophenyl)methanone, titanium tetrachloride ($TiCl_4$), tetrakis(triphenylphosphine) palladium(0) ($Pd(PPh_3)_4$), diphenylamine, pyrene and *N*-phenylnaphthalen-2-amine were purchased from J&K Scientific. Zinc dust and 3-hexylthiophene were purchased from Aladdin Industrial Co.. (4-nitrophenyl)boronic acid was purchased from Sukailu Co. (China). Potassium carbonate (K_2CO_3), tetrahydrofuran (THF) and other solvents were purchased from Guangzhou Dongzheng Co. (China) as analytical grade. All these materials above were used as received without further purification.

^1H NMR and ^{13}C NMR spectra were measured on a Bruker AVANCE III spectrometer in DMSO-d_6 or $CDCl_3$ (tetramethylsilane as the internal standard). The mass spectra were recorded on Thermo spectrometers (DSQ & MAT95XP-HRMS). Wide-angle X-ray diffraction patterns were obtained by using a Rigaku X-ray diffractometer (D/max-2200) with an X-ray source of Cu Kα (λ = 0.15406 nm) at 40 kV and 30 mA, at a scan rate of 10° (2θ) per 1 min. Variable-temperature powder X-ray diffraction (PXRD) data were performed using a Bruker X-ray diffractometer (D8 ADVANCE, Germany). The thermal behaviors were determined by differential scanning calorimetry (DSC) on a NETZSCH DSC 204 F1 instrument under nitrogen atmosphere at heating and cooling rates of 10 °C/min. TGA data was obtained on a Shimadzu TGA-50 thermogravimetric analyzer at a heating rate of 20 °C /min in nitrogen. CO_2 gas adsorption/desorption isotherms were obtained by a Micromeritics ASAP 2020 surface area analyzer. N_2 gas adsorption/desorption isotherms were obtained by a Micromeritics ASAP 2460 surface area analyzer. PL spectra were obtained on an Ocean Optics (QE65 Pro) with a 365 nm LED as the excitation light source. Luminescent images were taken by excitation with a 365 nm UV lamp. Time-resolved emission decay behaviors were monitored by using a spectrofluorometer (Horiba Scientific Fluorolog-3). The fluorescence quantum yields were achieved on a spectrofluorometer (Horiba Scientific Fluorolog-3) equipped with a Horiba Scientific Quanta-φ calibrated integrating sphere. The single-crystal X-ray diffraction data were collected from an Agilent Technologies Gemini A Ultra system with Cu-Kα radiation (λ = 1.54178 Å). All the structures were solved by direct methods following the difference Fourier syntheses and all non-hydrogen atoms were refined anisotropically through least-squares on F^2 by using the SHELXTL program suite. The scattering from the highly disordered guest molecules in voids was removed by using the SQUEEZE subroutine of the PLATON software suit. The theoretical calculations based on these single-crystal structures were performed using the B3LYP time-dependent density functional theory method at the 6-31G(d) level in the Gaussian 09 program.

General procedure for the synthesis of 1,1,2,2-tetrakis(4-bromophenyl)ethene (TPE-4Br). TPE-4Br was synthesized according to a literature procedure.[1] Bis(4-bromophenyl)methanone (5.00 g, 14.7 mmol), zinc dust (1.80 g, 28.1 mmol) and THF (50.0 mL) were added into a 250 mL three-necked flask under nitrogen atmosphere. After the mixture was cooled to -78 °C, $TiCl_4$ (1.75 mL, 16.1 mmol) was added into the flask slowly by a syringe. The mixture was stirred for 40 min at room temperature and then refluxed for 12 h. After the reaction was completed, the cooled mixture was poured into dilute hydrochloric acid (250 mL) and extracted with dichloromethane (3 x 100 mL). The collected organic layer was washed with water for three times and desiccated with anhydrous sodium sulfate. After filtration, the crude product was further purified by silica gel column chromatography with *n*-hexane as eluent. Compound TPE-4Br was obtained as a white solid in 51% yield (4.90 g).

General procedure for the synthesis of 1,1,2,2-tetrakis(4'-nitro-[1,1'-biphenyl]-4-yl)ethene (TPE-4pn). TPE-4Br (2.00 g, 3.10 mmol) and (4-nitrophenyl)boronic acid (3.09 g, 18.5 mmol) were dissolved in THF (50.0 mL). Subsequently, 2 M aqueous K_2CO_3 solution (6.00 mL) and Aliquat 336 (0.50 mL) were added. The mixture was stirred for 10 minutes under nitrogen atmosphere at room temperature. Then $Pd(PPh_3)_4$ (0.50 mg) was added and the mixture was stirred at 80 °C for 24 h. After cooled down to room temperature, the crude product was concentrated and purified by silica gel column chromatography with DCM/*n*-hexane (v/v = 1:1) as eluent. Compound TPE-4pn was obtained as a yellow solid in 68% yield (1.70 g). ^{1}H NMR (500 MHz, DMSO-d_6, δ): 8.24 (d, J = 8.9 Hz, 1H), 7.93 (d, J = 8.9 Hz, 1H), 7.69 (d, J = 8.4 Hz, 1H), 7.24 (d, J = 8.4 Hz, 1H); ^{13}C NMR (125 MHz, $CDCl_3$, δ): 147.12, 146.69, 143.84, 140.80, 137.20, 132.19, 127.51, 126.98, 124.15; EIMS m/z: $[M]^+$ calcd for $C_{50}H_{32}N_4O_8$, 816; found, 816.

Supplementary Figures

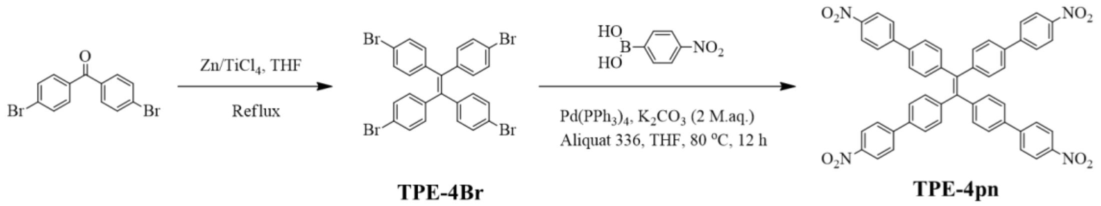

Supplementary Figure 1 | Synthetic routes of the organic building block TPE-4pn.

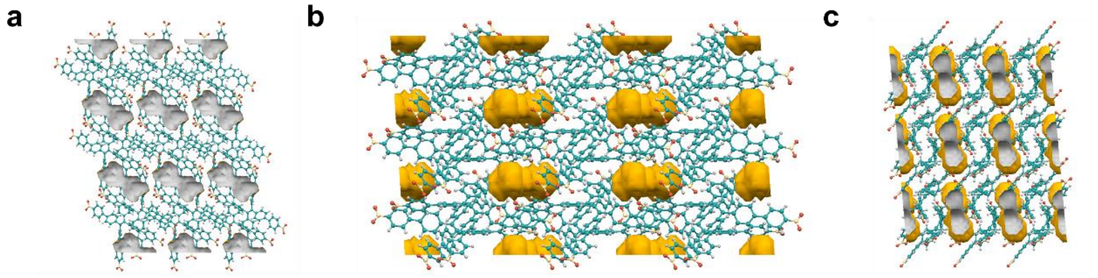

Supplementary Figure 2 | Packing diagrams of 8PN-ACT in different views: **a,** *a*-axis; **b,** *b*-axis and **c,** *c*-axis, with the solvent-accessible void space visualized by grey/yellow (inner/outer) curved planes generated with a probe of 1.2 Å. Color code: green, C; yellow, N; orange, O; grey, H.

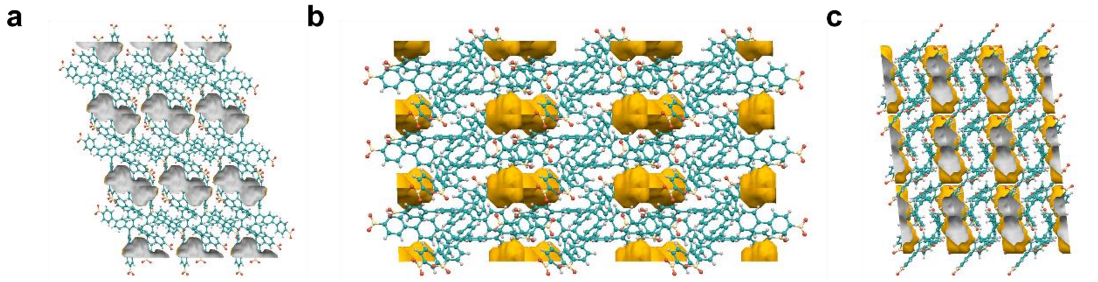

Supplementary Figure 3 | Packing diagrams of 8PN-DMF in different views: **a,** *a*-axis; **b,** *b*-axis and **c,** *c*-axis, with the solvent-accessible void space visualized by grey/yellow (inner/outer) curved planes generated with a probe of 1.2 Å. Color code: green, C; yellow, N; orange, O; grey, H.

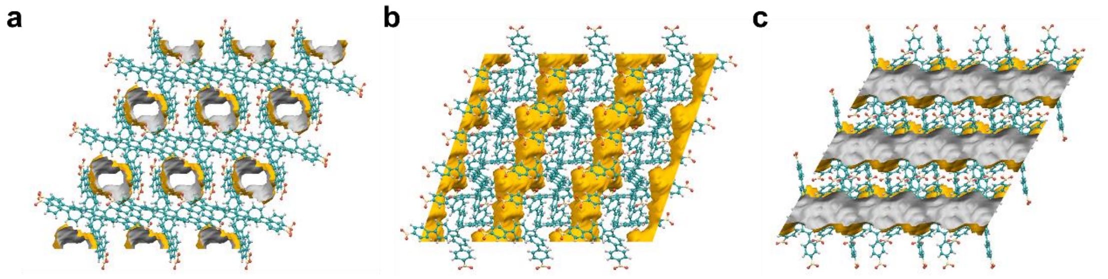

Supplementary Figure 4 | Packing diagrams of 8PN-EA in different views: **a,** *a*-axis; **b,** *b*-axis and **c,** *c*-axis, with the solvent-accessible void space visualized by grey/yellow (inner/outer) curved planes generated with a probe of 1.2 Å. Color code: green, C; yellow, N; orange, O; grey, H.

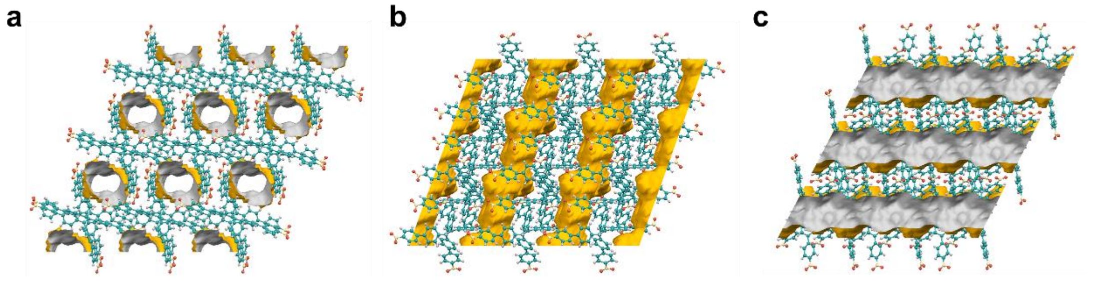

Supplementary Figure 5 | Packing diagrams of 8PN-2ACT in different views: **a,** *a*-axis; **b,** *b*-axis and **c,** *c*-axis, with the solvent-accessible void space visualized by grey/yellow (inner/outer) curved planes generated with a probe of 1.2 Å. Color code: green, C; yellow, N; orange, O; grey, H.

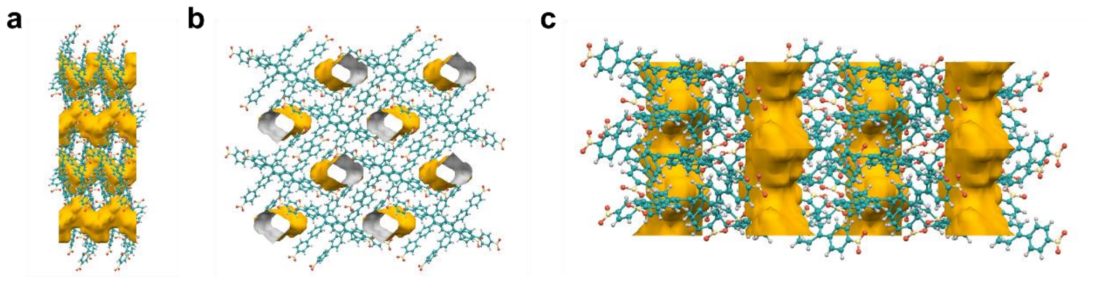

Supplementary Figure 6 | Packing diagrams of 8PN-TCM in different views: a, a-axis; b, b-axis and c, c-axis, with the solvent-accessible void space visualized by grey/yellow (inner/outer) curved planes generated with a probe of 1.2 Å. Color code: green, C; yellow, N; orange, O; grey, H.

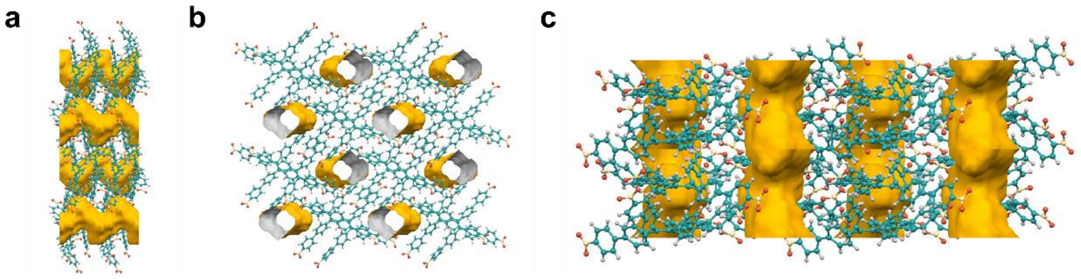

Supplementary Figure 7 | Packing diagrams of 8PN-THF in different views: **a,** *a*-axis; **b,** *b*-axis and **c,** *c*-axis, with the solvent-accessible void space visualized by grey/yellow (inner/outer) curved planes generated with a probe of 1.2 Å. Color code: green, C; yellow, N; orange, O; grey, H.

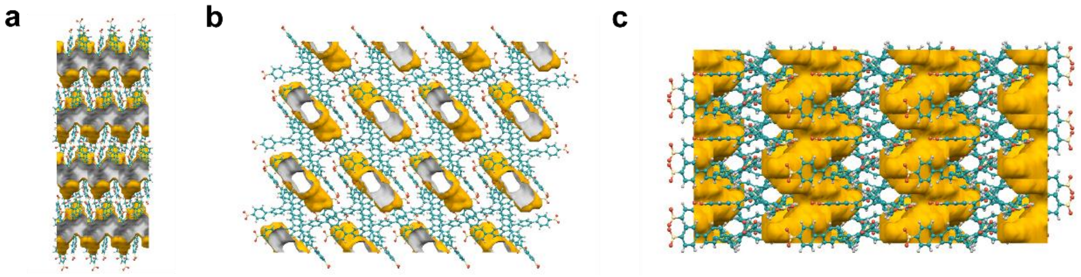

Supplementary Figure 8 | Packing diagrams of 8PN-DCM in different views: **a,** *a*-axis; **b,** *b*-axis and **c,** *c*-axis, with the solvent-accessible void space visualized by grey/yellow (inner/outer) curved planes generated with a probe of 1.2 Å. Color code: green, C; yellow, N; orange, O; grey, H.

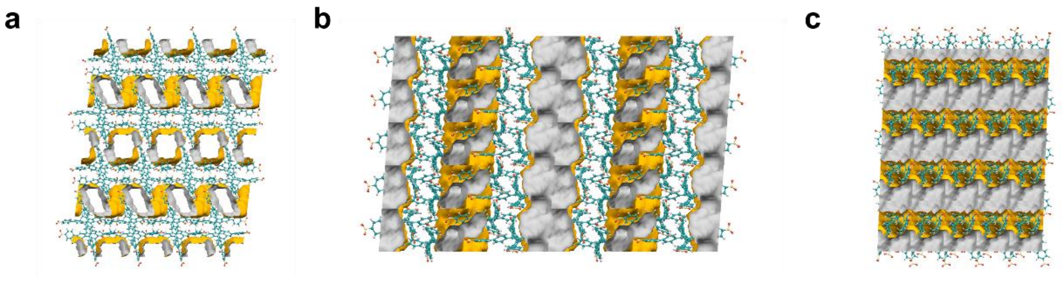

Supplementary Figure 9 | Packing diagrams of 8PN-TOL in different views: **a,** *a*-axis; **b,** *b*-axis and **c,** *c*-axis, with the solvent-accessible void space visualized by grey/yellow (inner/outer) curved planes generated with a probe of 1.2 Å. Color code: green, C; yellow, N; orange, O; grey, H.

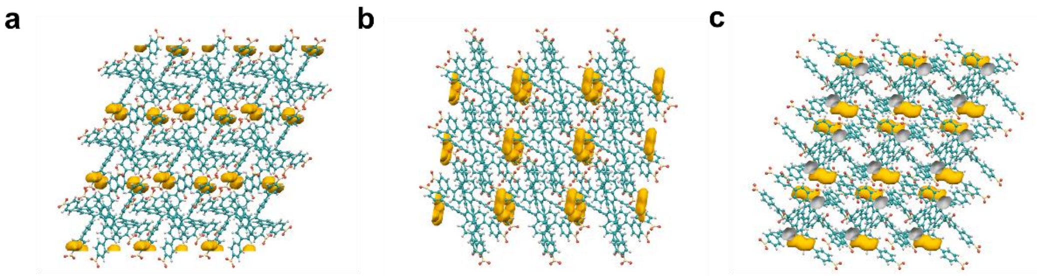

Supplementary Figure 10 | Packing diagrams of 8PN-Heated in different views: **a,** *a*-axis; **b,** *b*-axis and **c,** *c*-axis, with the solvent-accessible void space visualized by grey/yellow (inner/outer) curved planes generated with a probe of 1.2 Å. Color code: green, C; yellow, N; orange, O; grey, H.

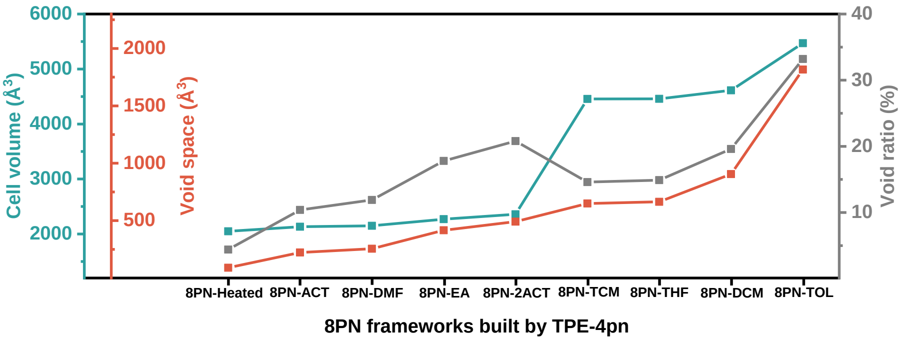

Supplementary Figure 11 | Void spaces, crystal volumes, and void ratios of 8PN frameworks.

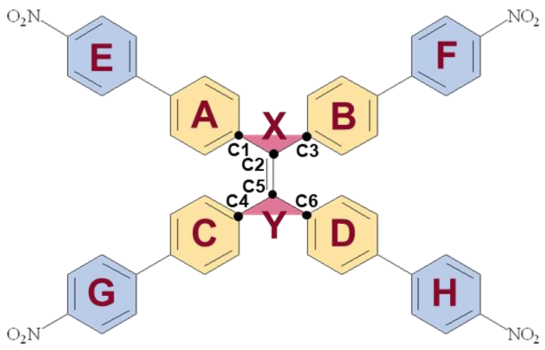

Supplementary Figure 12 | Illustration used to define the planes of the ethylene core and phenyl rings. Planes X and Y used to define the planes of the ethyl core are constructed by carbon atoms C1/C2/C3 and C4/C5/C6, respectively. The four phenyl rings in TPE moieties are defined as planes A, B, C and D, whereas planes E, F, G and H stand for four phenyl rings close to the nitro groups. For unification and convenience, in such mode of labeling, planes X and A of TPE-4pn are identified as planes which exhibit the largest dihedral angle among the four dihedral angles A^X, B^X, C^Y and D^Y. In terms of the impact on the pore size, two sets of dihedral angles (namely A^X, B^X, C^Y, D^Y and E^X, F^X, G^Y, H^Y) in TPE-4pn play similar roles. The relationship between dihedral angles A^X, B^X, C^Y and D^Y and the pore size is emphasized in analyses and the other four dihedral angles affect the pores in an analogical way.

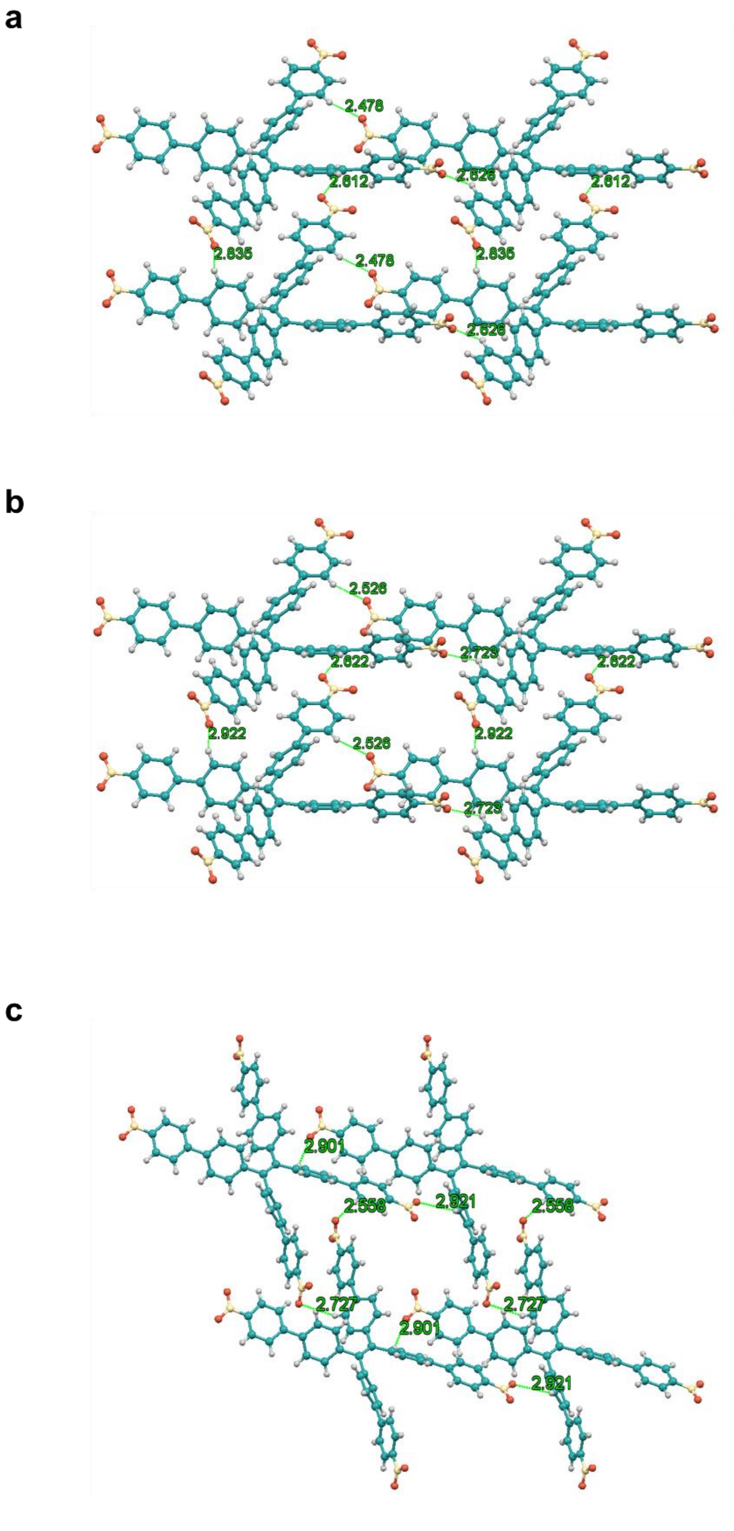
a
2.478
2.612
2.526
2.612
2.835
2.478
2.835
2.526
b
2.526
2.622
2.729
2.622
2.922
2.526
2.922
2.729
c
2.901
2.558
2.921
2.558
2.727
2.727
2.901
2.921

d

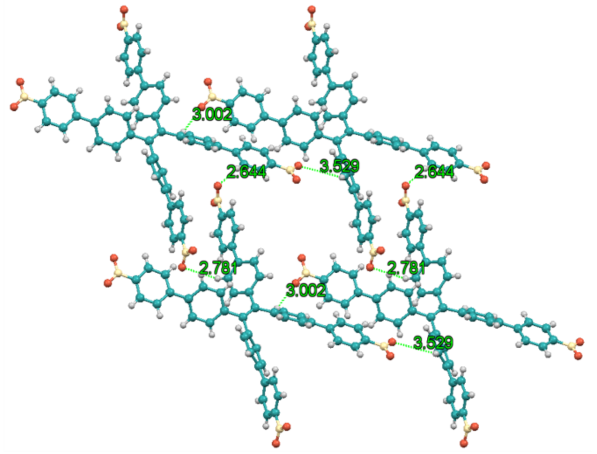

e

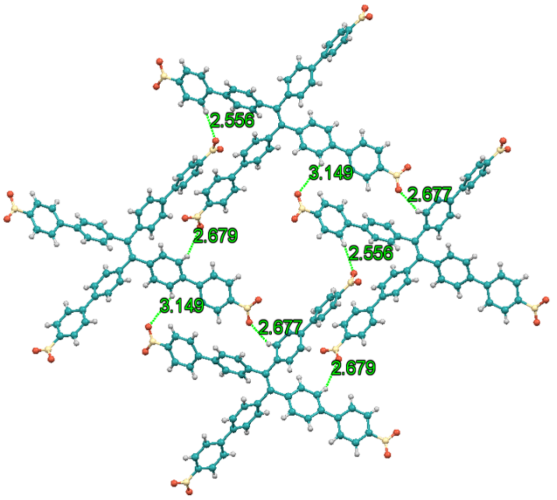

f

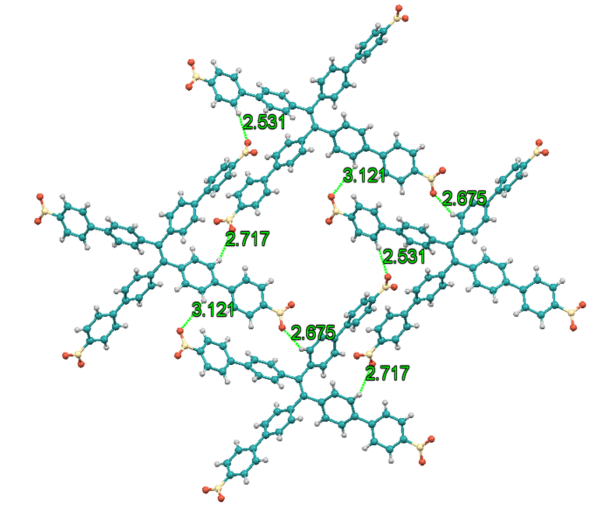

Supplementary Figure 13 | Measurements of hydrogen bond distances in **a,** 8PN-ACT; **b,** 8PN-DMF; **c,** 8PN-EA; **d,** 8PN-2ACT; **e,** 8PN-TCM and **f,** 8PN-THF. Color code: green, C; yellow, N; orange, O; grey, H.

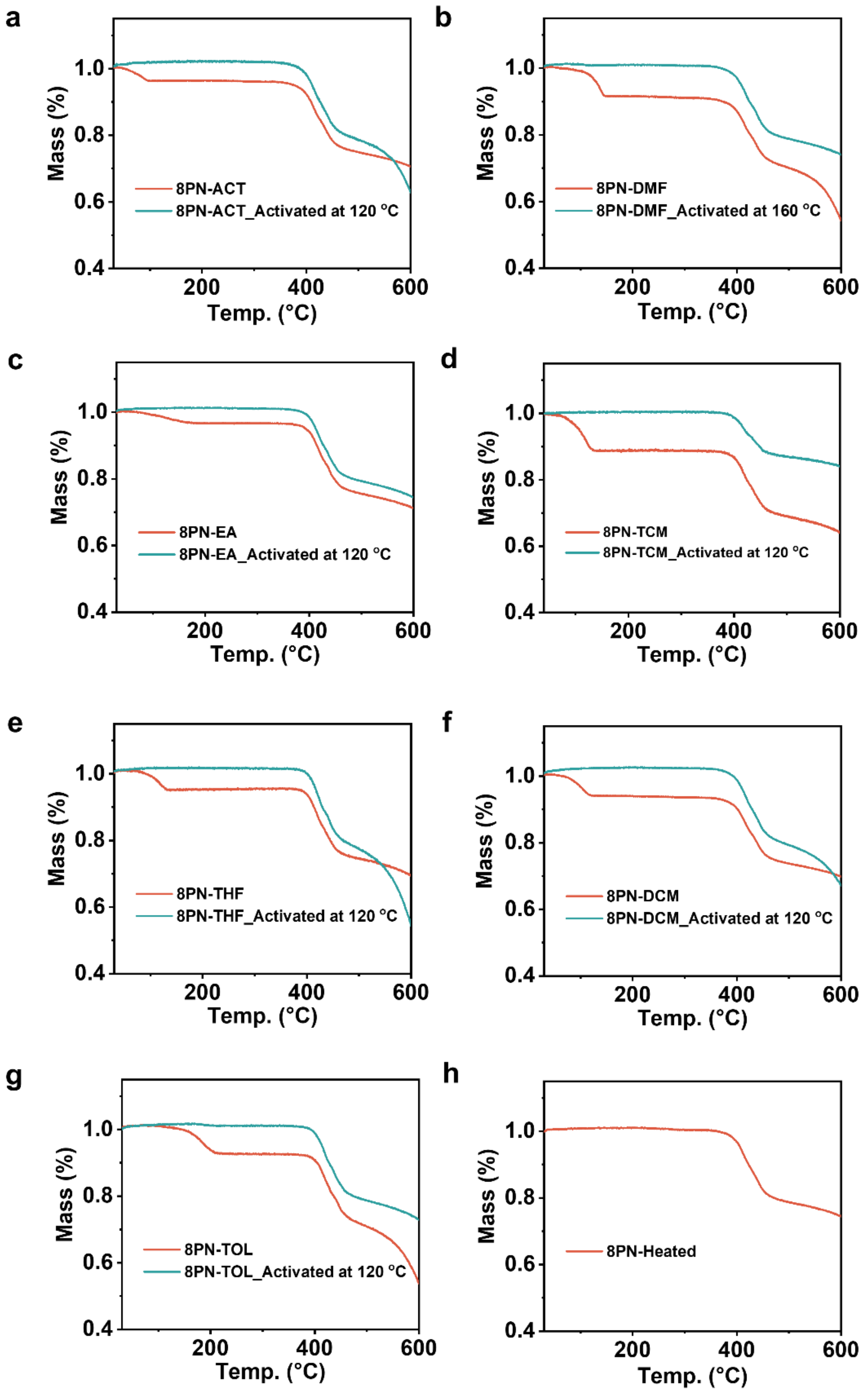

Supplementary Figure 14 | TGA curves of as-prepared and activated samples of **a,** 8PN-ACT; **b,** 8PN-DMF; **c,** 8PN-EA; **d,** 8PN-TCM; **e,** 8PN-THF; **f,** 8PN-DCM; **g,** 8PN-TOL and **h,** 8PN-Heated.

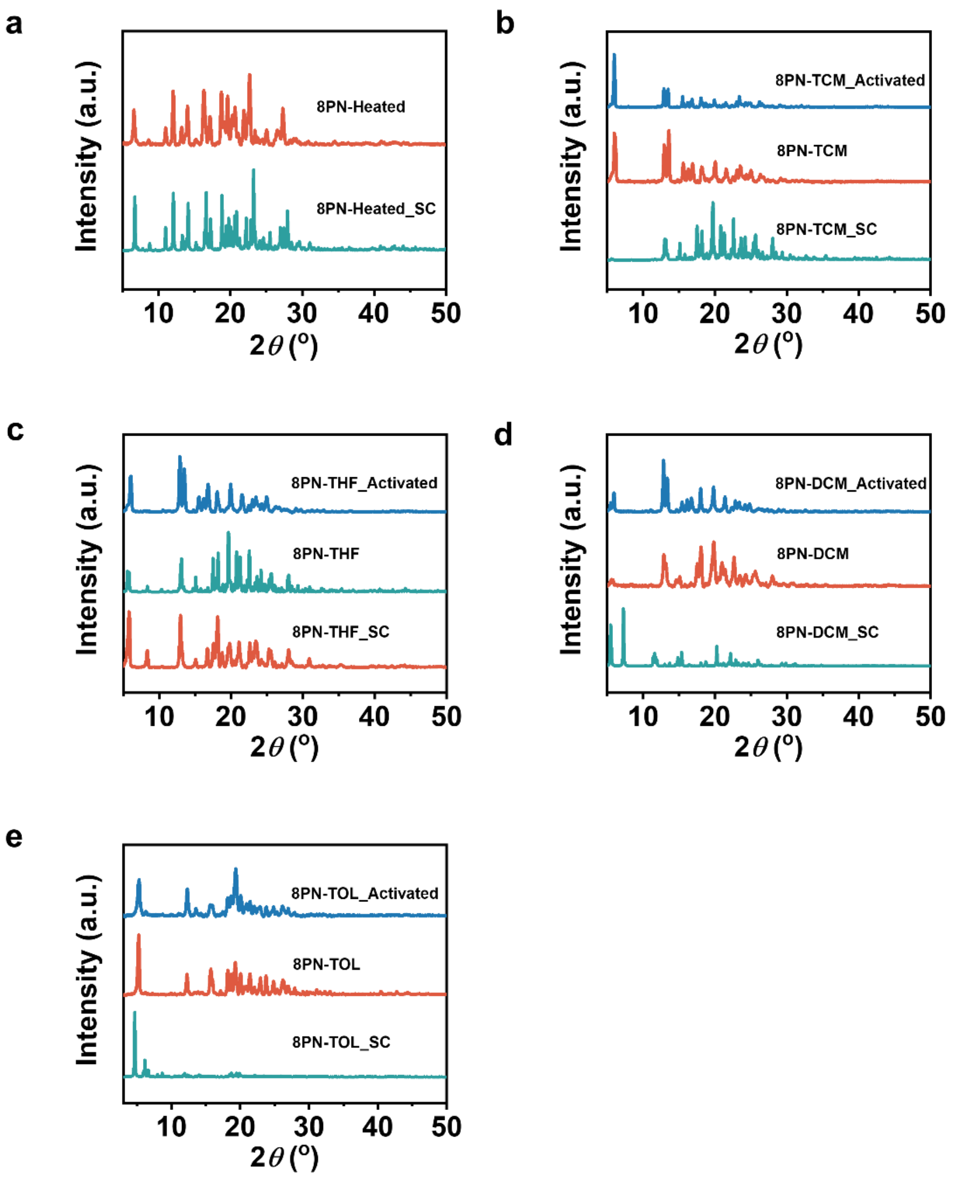

Supplementary Figure 15 | PXRD patterns of as-prepared samples, activated samples for CO_2 gas adsorption measurements and the simulated PXRD spectra from single-crystal structures of **a,** 8PN-Heated; **b,** 8PN-TCM; **c,** 8PN-THF; **d,** 8PN-DCM and **e,** 8PN-TOL.

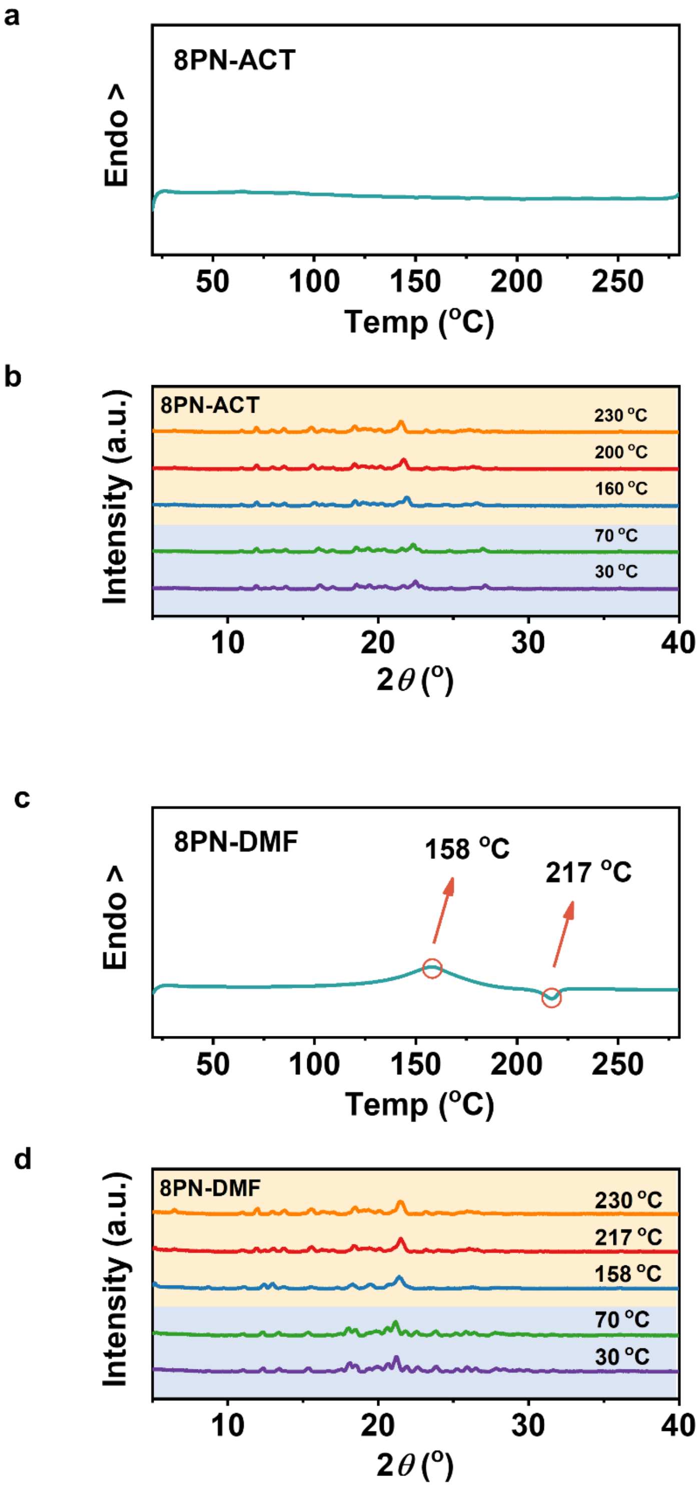
a
8PN-ACT
Endo >
50
100
150
200
250
Temp (°C)
b
8PN-ACT
230 °C
200 °C
160 °C
70 °C
30 °C
Intensity (a.u.)
10
20
30
40
2θ (°)
c
8PN-DMF
158 °C
217 °C
Endo >
50
100
150
200
250
Temp (°C)
d
8PN-DMF
230 °C
217 °C
158 °C
70 °C
30 °C
Intensity (a.u.)
10
20
30
40
2θ (°)

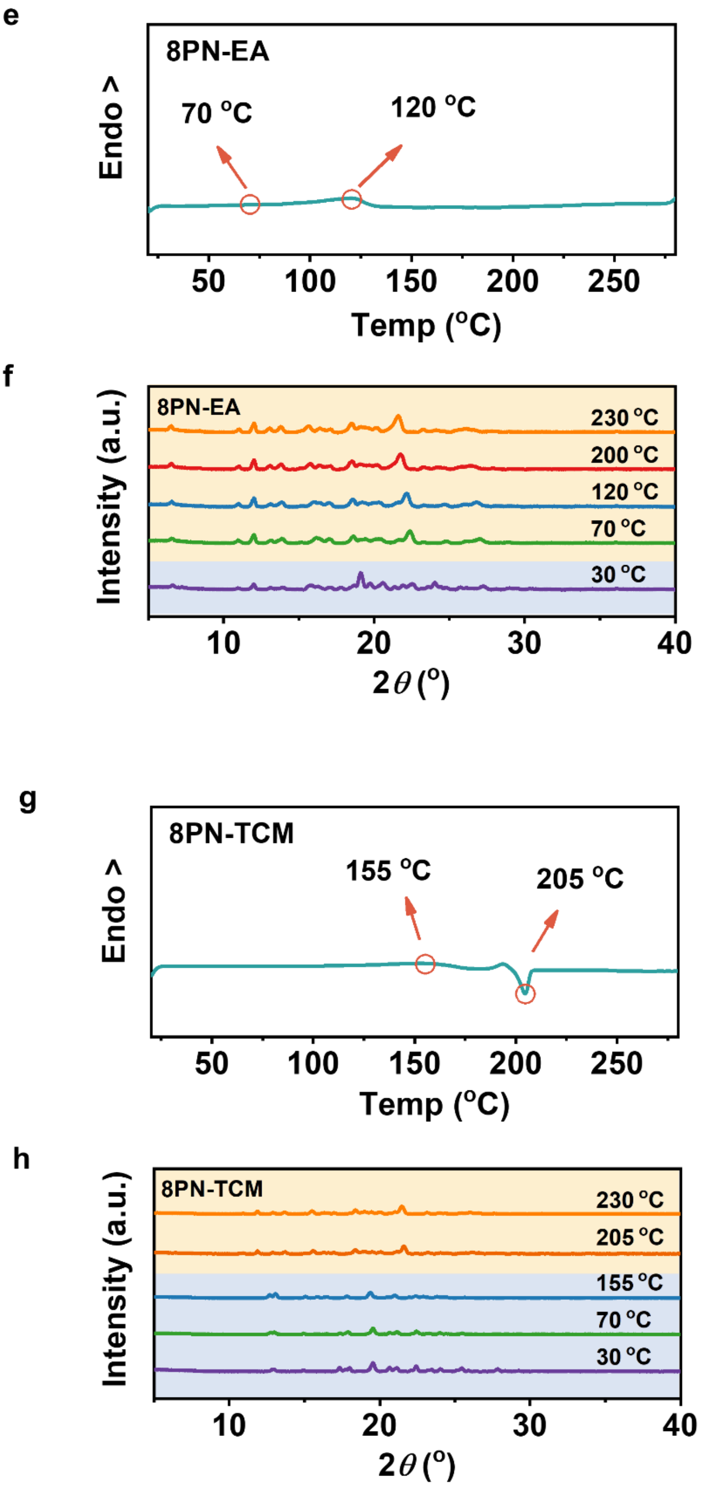
e
8PN-EA
Endo >
70 °C
120 °C
50
100
150
200
250
Temp (°C)
f
8PN-EA
Intensity (a.u.)
230 °C
200 °C
120 °C
70 °C
30 °C
10
20
30
40
2θ (°)
g
8PN-TCM
Endo >
155 °C
205 °C
50
100
150
200
250
Temp (°C)
h
8PN-TCM
Intensity (a.u.)
230 °C
205 °C
155 °C
70 °C
30 °C
10
20
30
40
2θ (°)

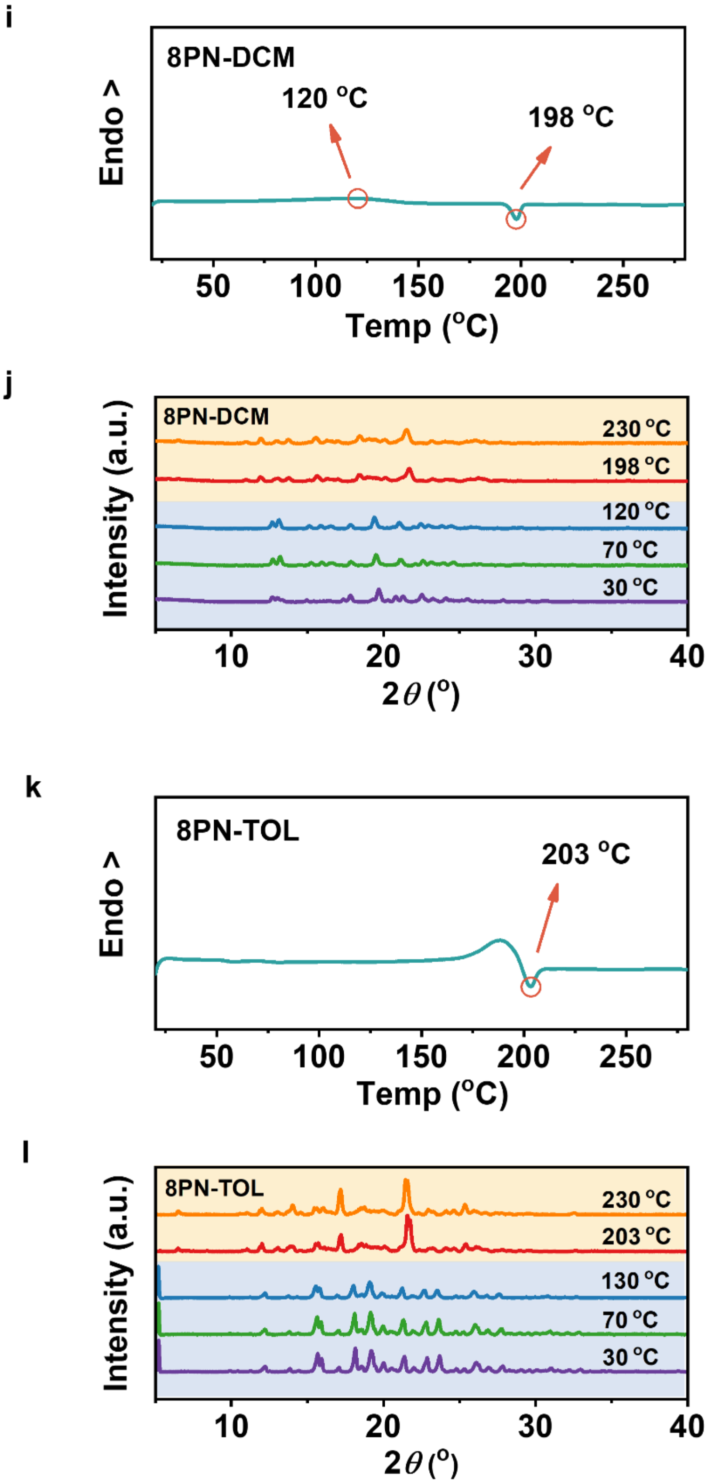

Supplementary Figure 16 | DSC curves and variable-temperature PXRD patterns of **a, b,** 8PN-ACT; **c, d,** 8PN-DMF; **e, f,** 8PN-EA; **g, h,** 8PN-TCM; **i, j,** 8PN-DCM and **k, l,** 8PN-TOL, indicating that the crystal structures of 8PN-ACT, 8PN-DMF and 8PN-EA are changed upon solvent removal and 8PN-TCM, 8PN-DCM and 8PN-TOL will maintain their crystal structures until heated to 200 °C.

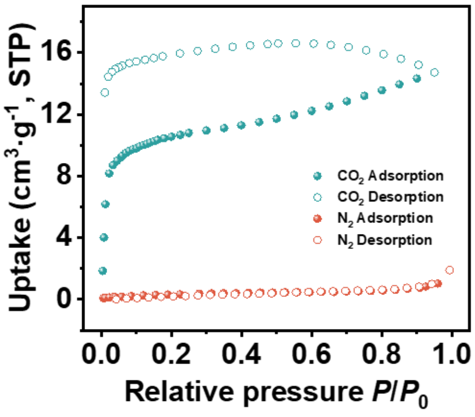

Supplementary Figure 17 | CO_2 (195 K) and N_2 (77 K) adsorption/desorption isotherms for 8PN-Heated. Source data are provided as a Source Data file**.**

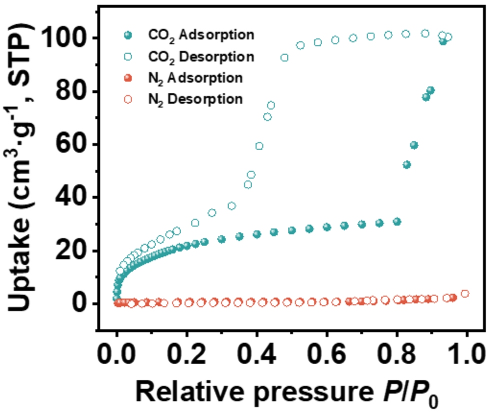

Supplementary Figure 18 | CO_2 (195 K) and N_2 (77 K) adsorption/desorption isotherms for 8PN-TCM. Source data are provided as a Source Data file**.**

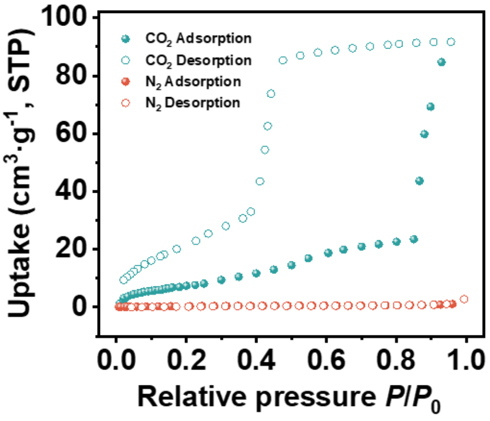

Supplementary Figure 19 | CO_2 (195 K) and N_2 (77 K) adsorption/desorption isotherms for 8PN-DCM. Source data are provided as a Source Data file**.**

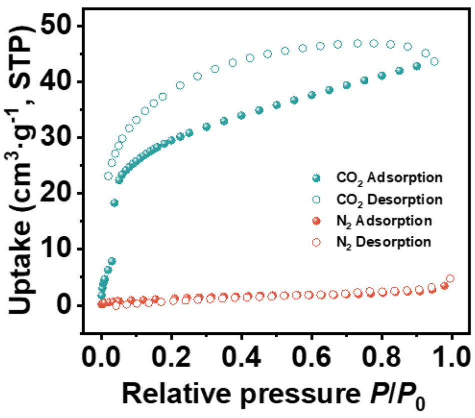

Supplementary Figure 20 | CO_2 (195 K) and N_2 (77 K) adsorption/desorption isotherms for 8PN-TOL. Source data are provided as a Source Data file**.**

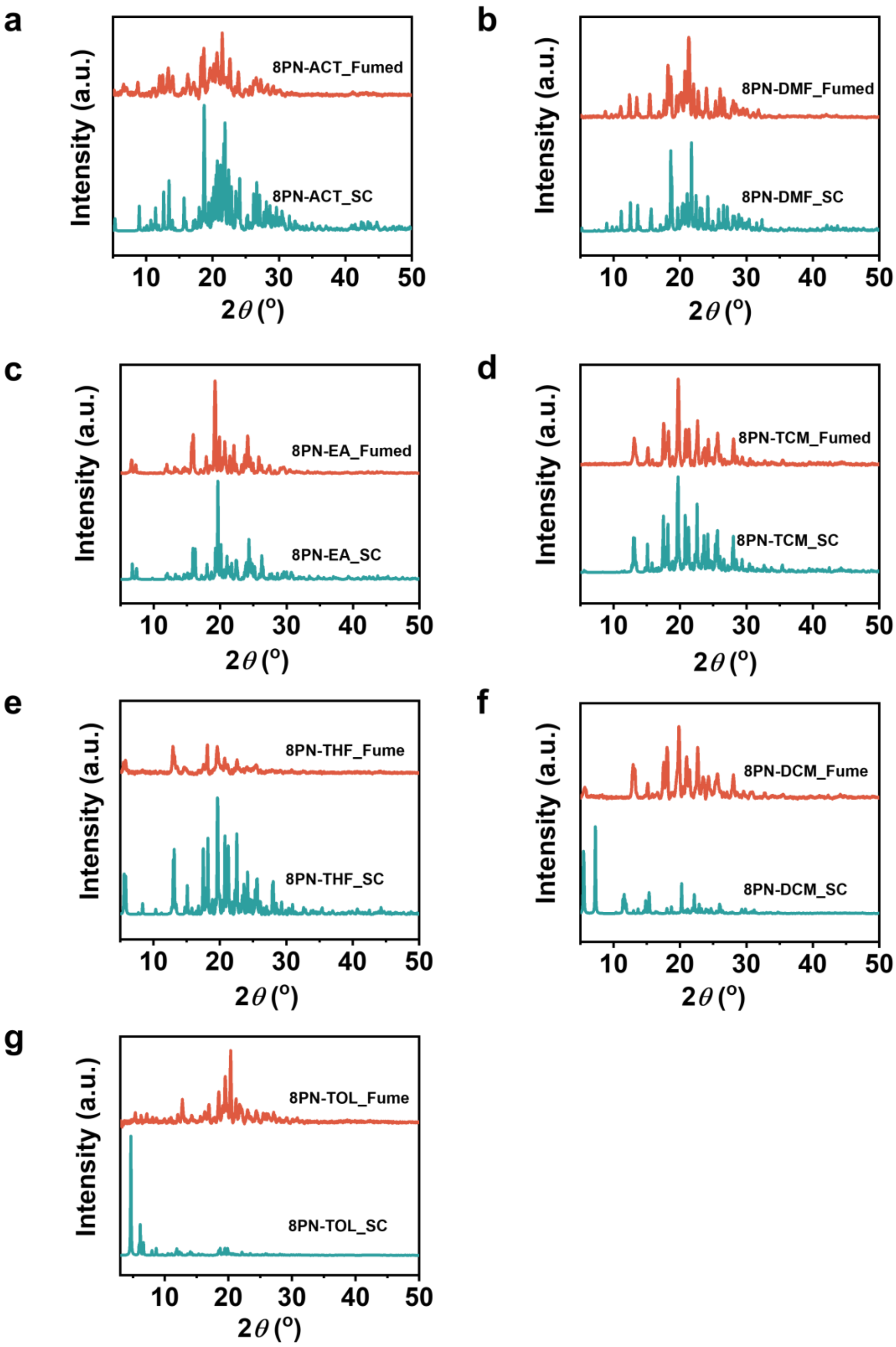

Supplementary Figure 21 | PXRD patterns of crystals of 8PN-Heated exposed to different solvent vapors and the simulated PXRD spectra from single-crystal structures of **a,** 8PN-ACT; **b,** 8PN-DMF; **c,** 8PN-EA; **d,** 8PN-TCM; **e,** 8PN-THF; **f,** 8PN-DCM and **g,** 8PN-TOL.

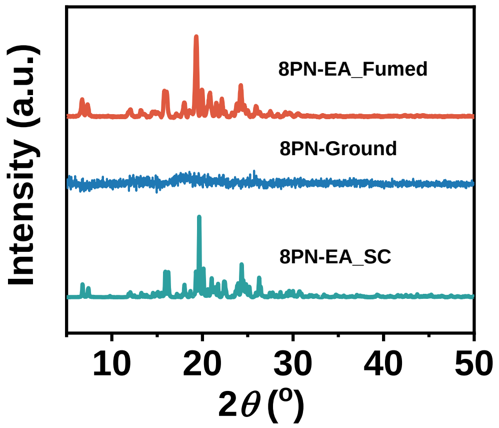

Supplementary Figure 22 | PXRD patterns of 8PN-Ground, the EA fumed ground 8PN sample and the simulated PXRD spectra from single-crystal structures of 8PN-EA.

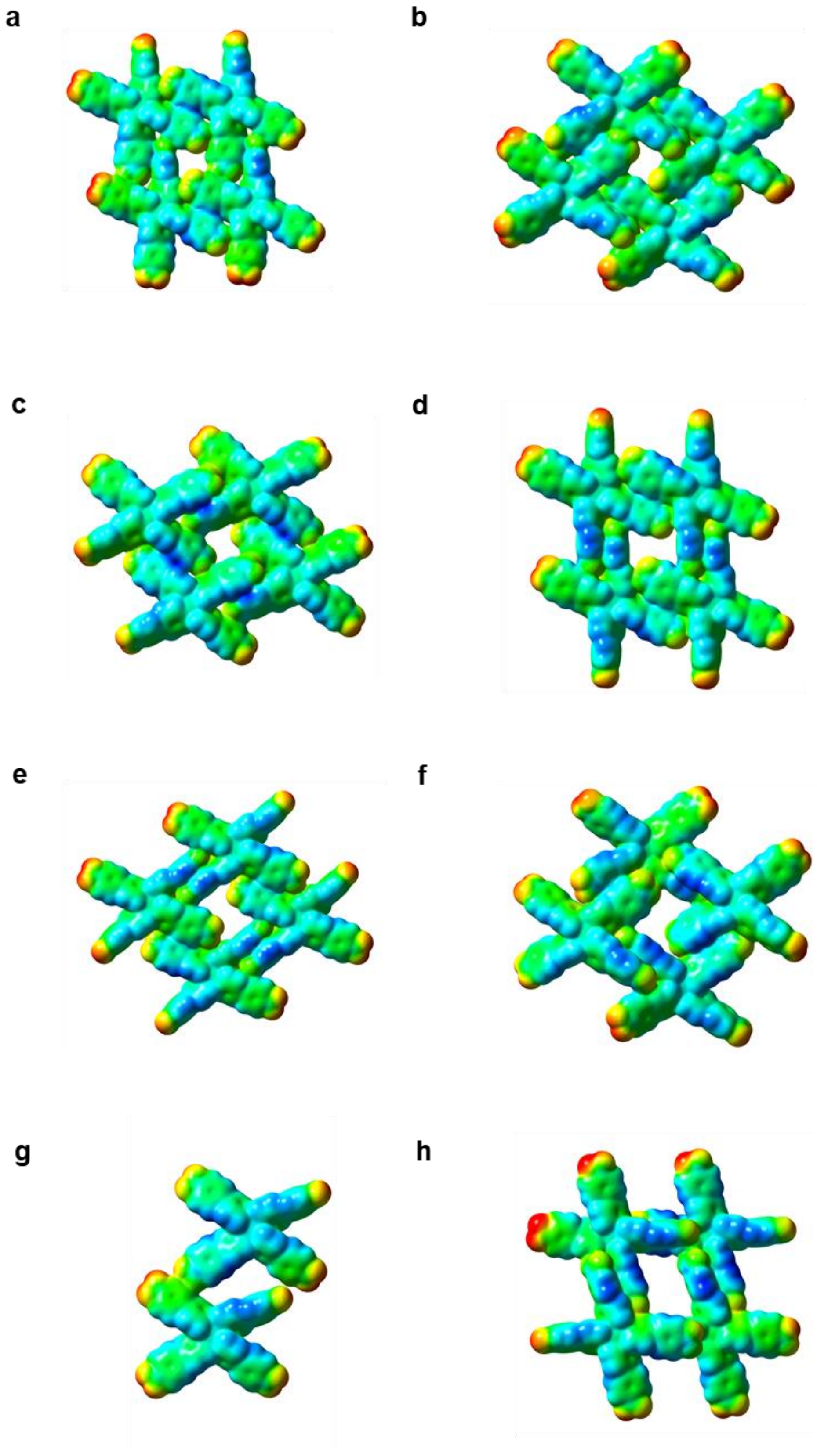
a
b
c
d
e
f
g
h

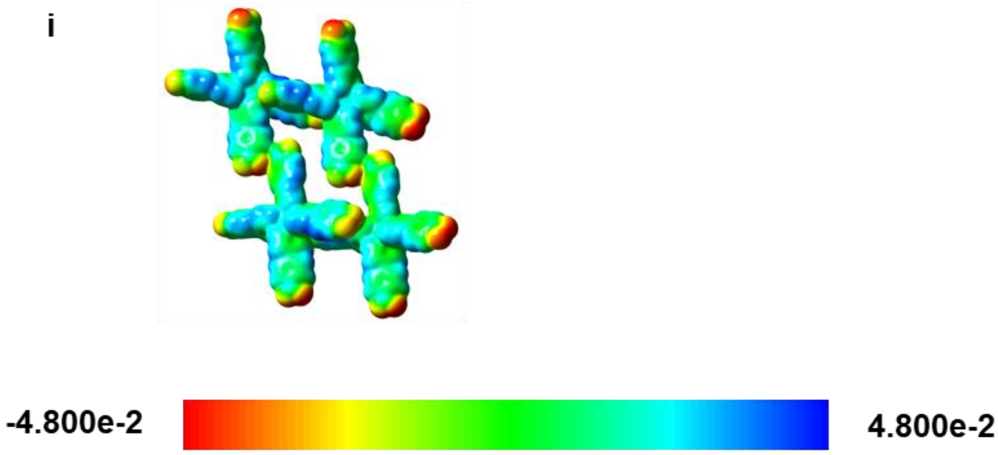

Supplementary Figure 23 | Electrostatic potential analyses of **a,** 8PN-Heated; **b,** 8PN-ACT; **c,** 8PN-DMF; **d,** 8PN-EA; **e,** 8PN-2ACT; **f,** 8PN-TCM; **g,** 8PN-DCM; **h,** LP of 8PN-TOL and **i,** NP of 8PN-TOL. The potential energy range is -4.8×10^{-2} to 4.8×10^{-2} for all surfaces shown.

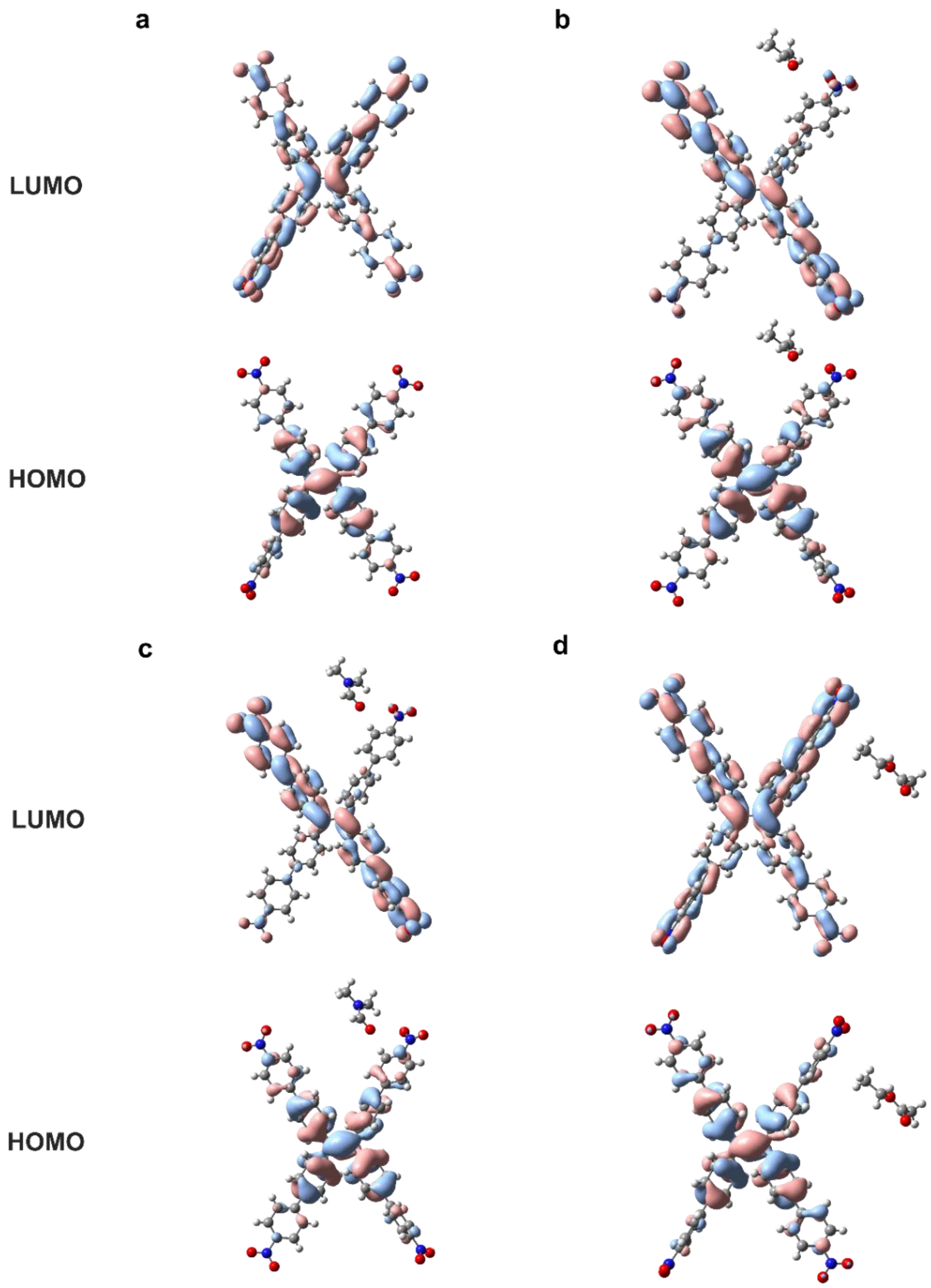
a
b
LUMO
HOMO
c
d
LUMO
HOMO

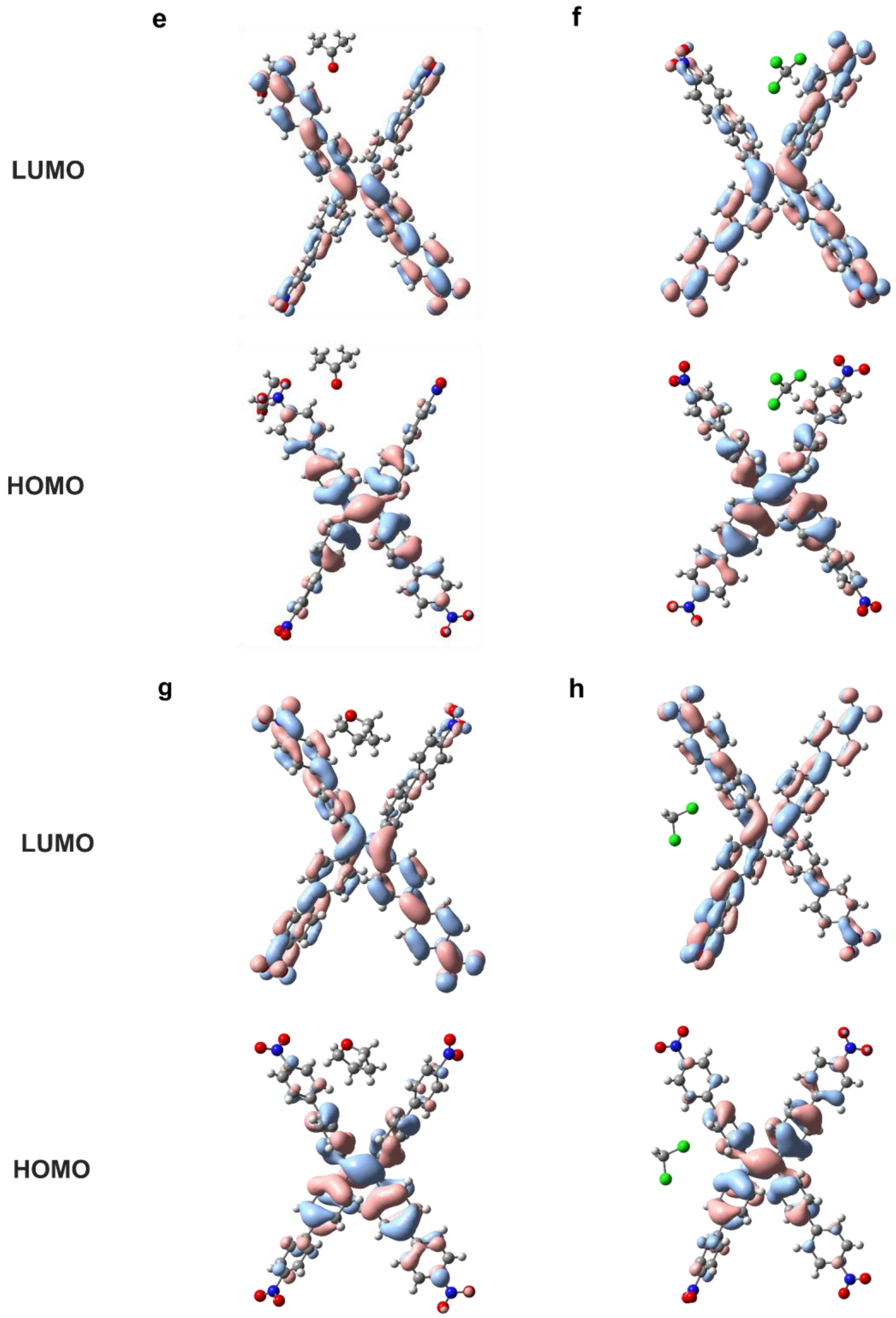
e
f
LUMO
HOMO
g
h
LUMO
HOMO

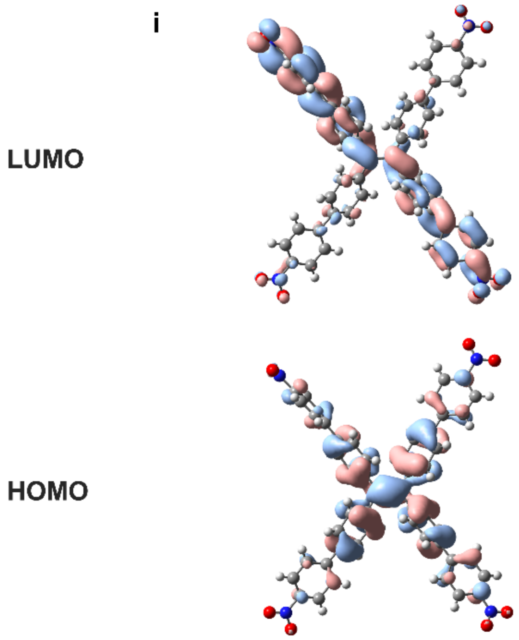

Supplementary Figure 24 | Electrical contour diagrams of HOMO and LUMO of **a,** 8PN-Heated; **b,** 8PN-ACT; **c,** 8PN-DMF; **d,** 8PN-EA; **e,** 8PN-2ACT; **f,** 8PN-TCM; **g,** 8PN-THF; **h,** 8PN-DCM and **i,** 8PN-TOL. For the TPE-4pn molecules in all the crystalline structures of 8PN, the highest occupied molecular orbitals (HOMOs) are mainly distributed on the TPE core, whereas the lowest unoccupied molecular orbitals (LUMOs) shift towards the nitro groups to some extent. The electronic clouds on HOMOs and LUMOs exhibit obvious spatial separations. Color code: grey, C; blue, N; red, O; white, H; brilliant green, Cl.

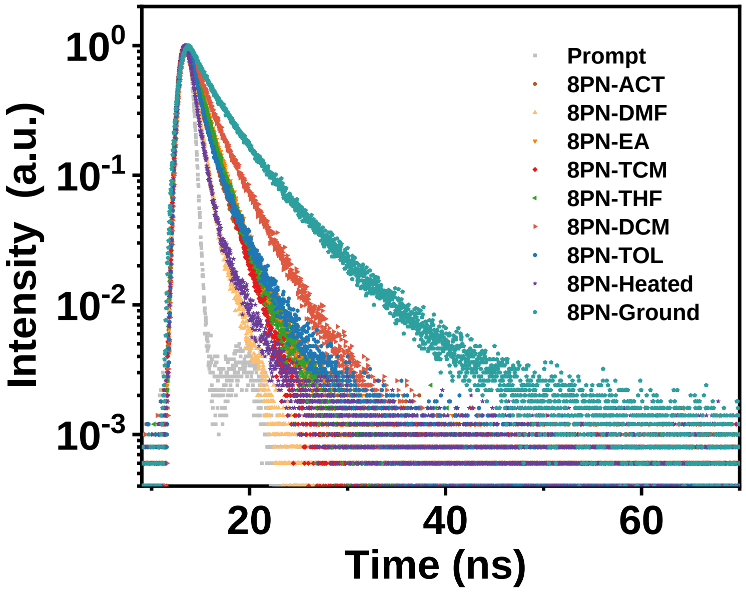

Supplementary Figure 25 | Time-resolved PL-decay curves of different forms of 8PN.

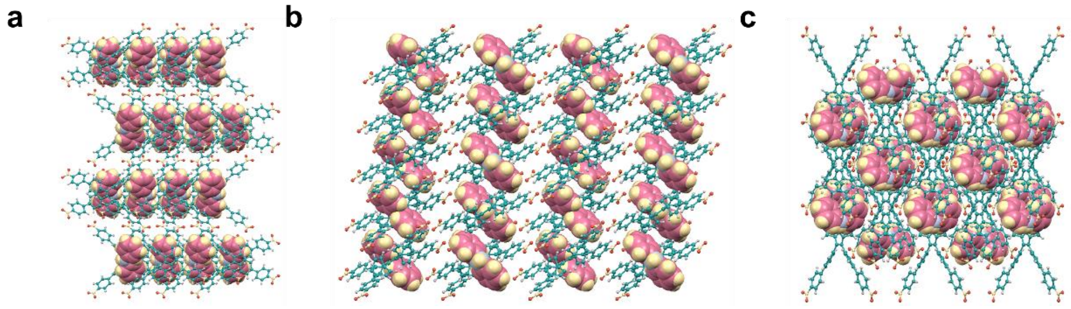

Supplementary Figure 26 | Packing diagrams of 8PN-NDP in different views: **a,** *a*-axis; **b,** *b*-axis and **c,** *c*-axis. Color code for TPE-4pn: green, C; yellow, N; orange, O; grey, H. Color code for diphenylamine: pink, C; blue, N; yellow, H. solvent molecules in voids are omitted for clarity.

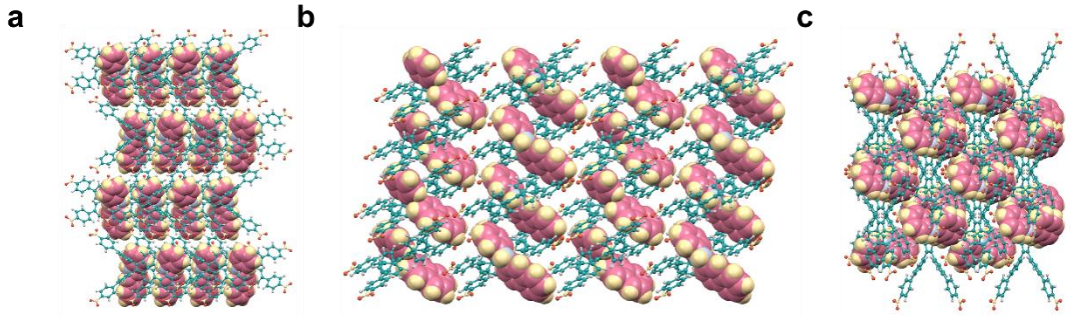

Supplementary Figure 27 | Packing diagrams of 8PN-NPNA in different views: **a,** *a*-axis; **b,** *b*-axis and **c,** *c*-axis. Color code for TPE-4pn: green, C; yellow, N; orange, O; grey, H. Color code for N-phenylnaphthalen-2-amine: pink, C; blue, N; yellow, H. solvent molecules in voids are omitted for clarity.

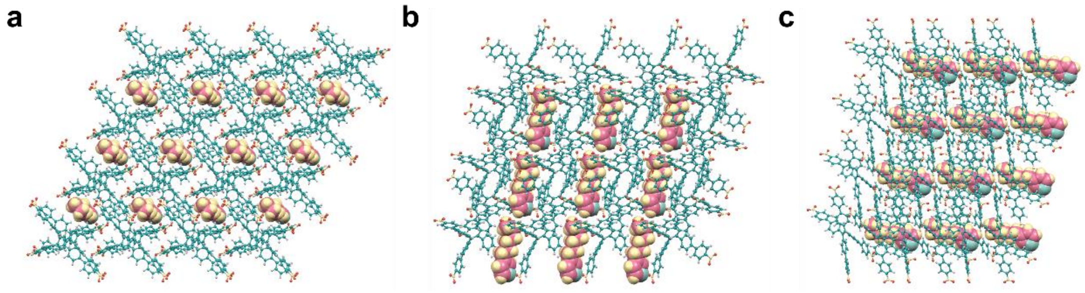

Supplementary Figure 28 | Packing diagrams of 8PN-SC6 in different views: **a,** *a*-axis; **b,** *b*-axis and **c,** *c*-axis. Color code for TPE-4pn: green, C; yellow, N; orange, O; grey, H. Color code for 3-hexylthiophene: pink, C; light green, S; yellow, H.

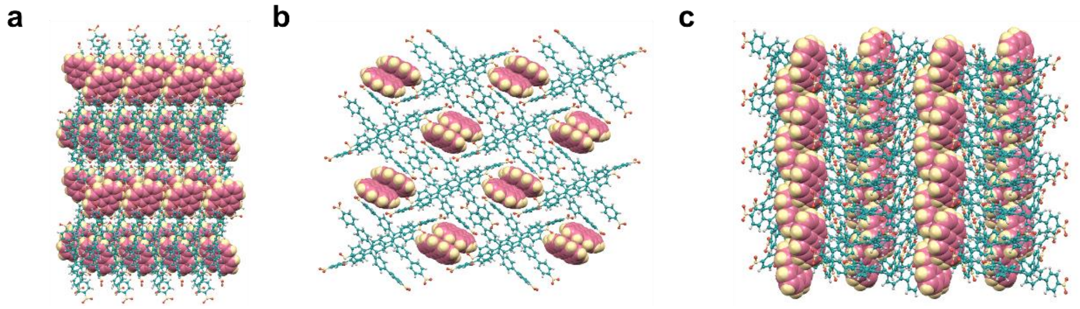

Supplementary Figure 29 | Packing diagrams of 8PN-PY in different views: **a,** *a*-axis; **b,** *b*-axis and **c,** *c*-axis. Color code for TPE-4pn: green, C; yellow, N; orange, O; grey, H. Color code for pyrene: pink, C; yellow, H.

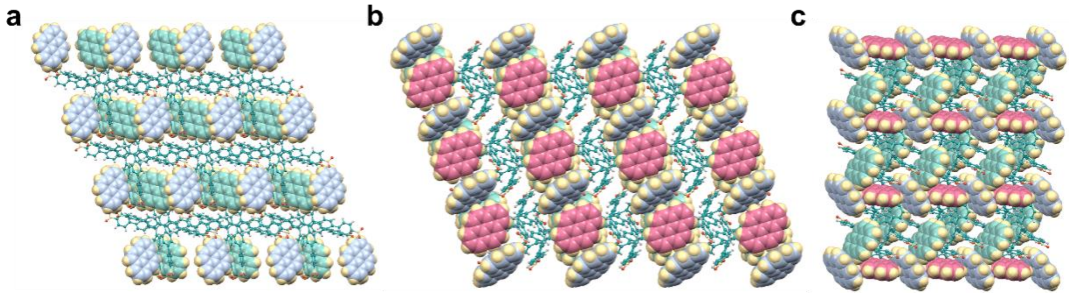

Supplementary Figure 30 | Packing diagrams of 8PN-3PY in different views: **a,** *a*-axis; **b,** *b*-axis and **c,** *c*-axis. Color code for TPE-4pn: green, C; yellow, N; orange, O; grey, H. H atoms of pyrene are colored yellow and C atoms of the three pyrene molecules in the unit cell are colored pink, light green and light blue respectively for clarity.

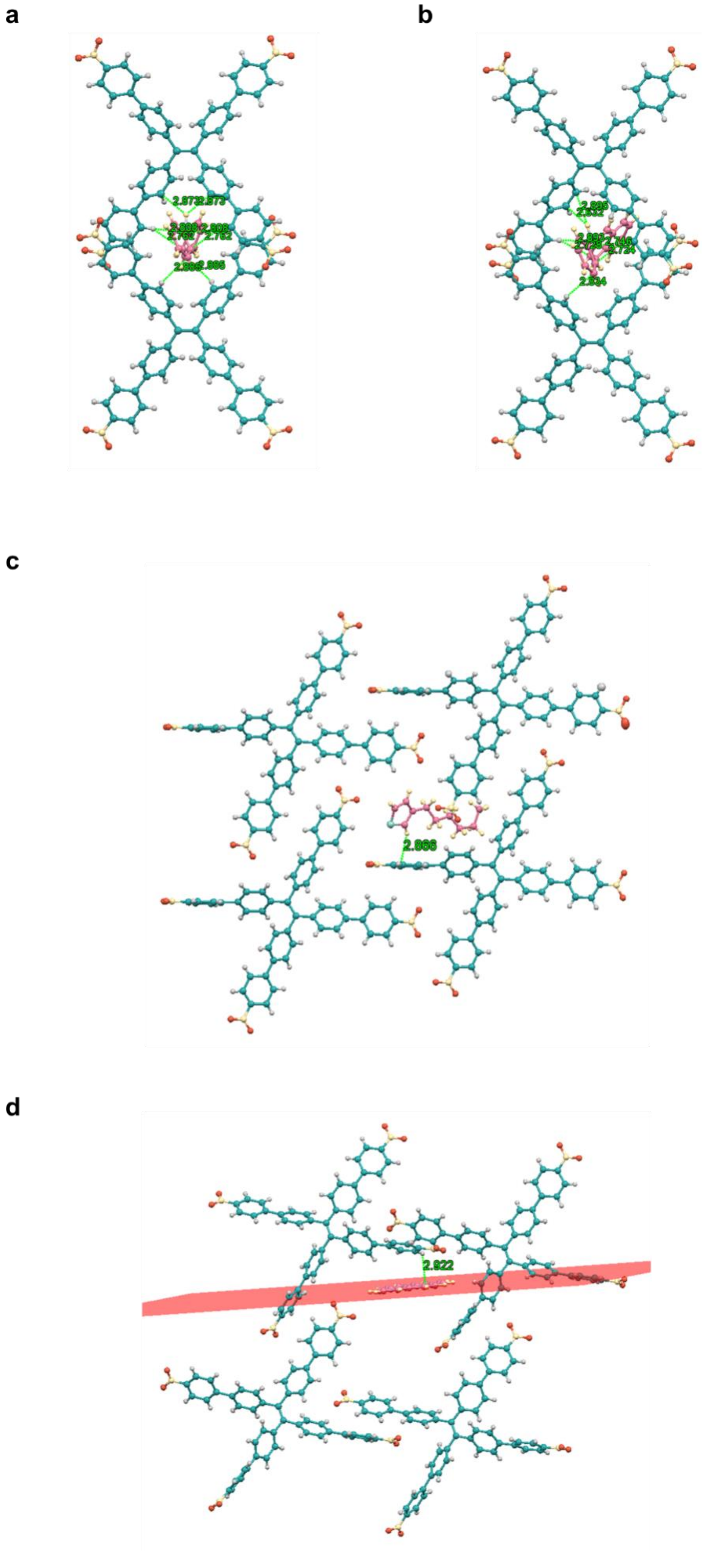
a
b
c
2.866
d
2.822

e

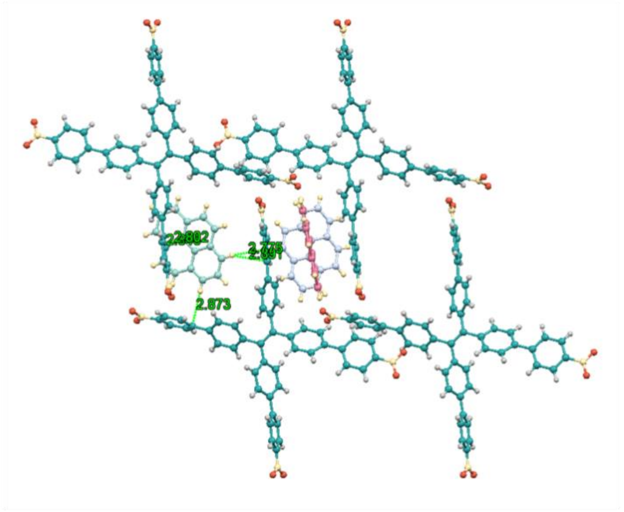

f

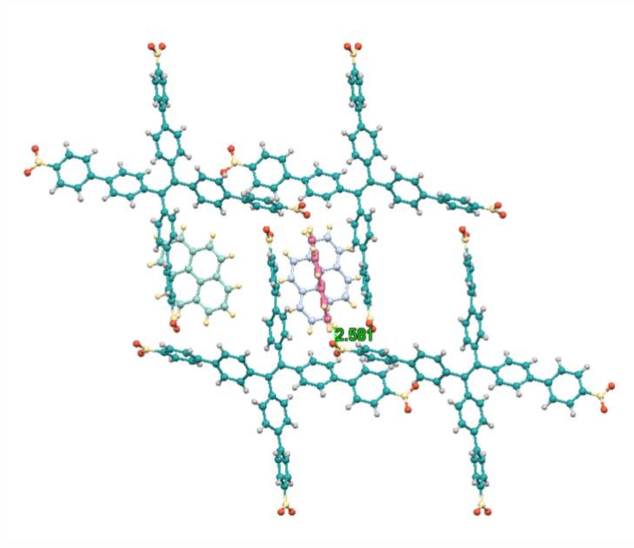

g

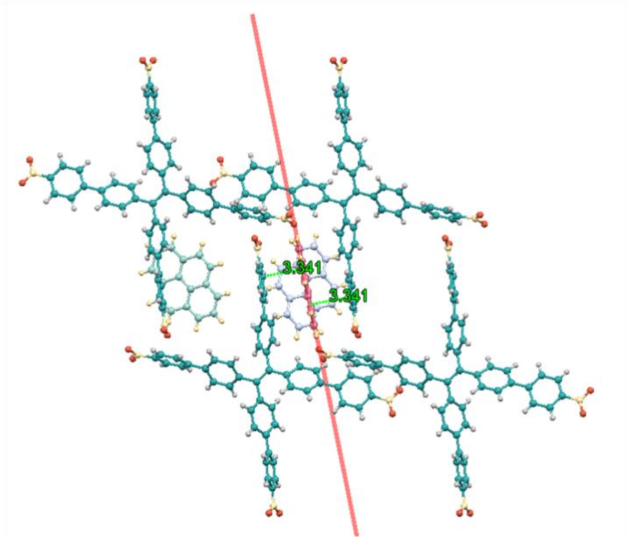

Supplementary Figure 31 | Intermolecular interactions in **a,** 8PN-NDP; **b,** 8PN-NPNA; **c,** 8PN-SC6; **d,** 8PN-PY and **e-g,** 8PN-3PY. Color code for TPE-4pn: green, C; yellow, N; orange, O;

grey, H. Color code for diphenylamine, N-phenylnaphthalen-2-amine and 3-hexylthiophene: pink, C; blue, N; light green, S; yellow, H. Color code for pyrene in 8PN-PY: pink, C; yellow, H. H atoms of pyrene in 8PN-3PY are colored yellow and C atoms of the three pyrene molecules in the unit cell of 8PN-3PY are colored pink, light green and light blue respectively for clarity.

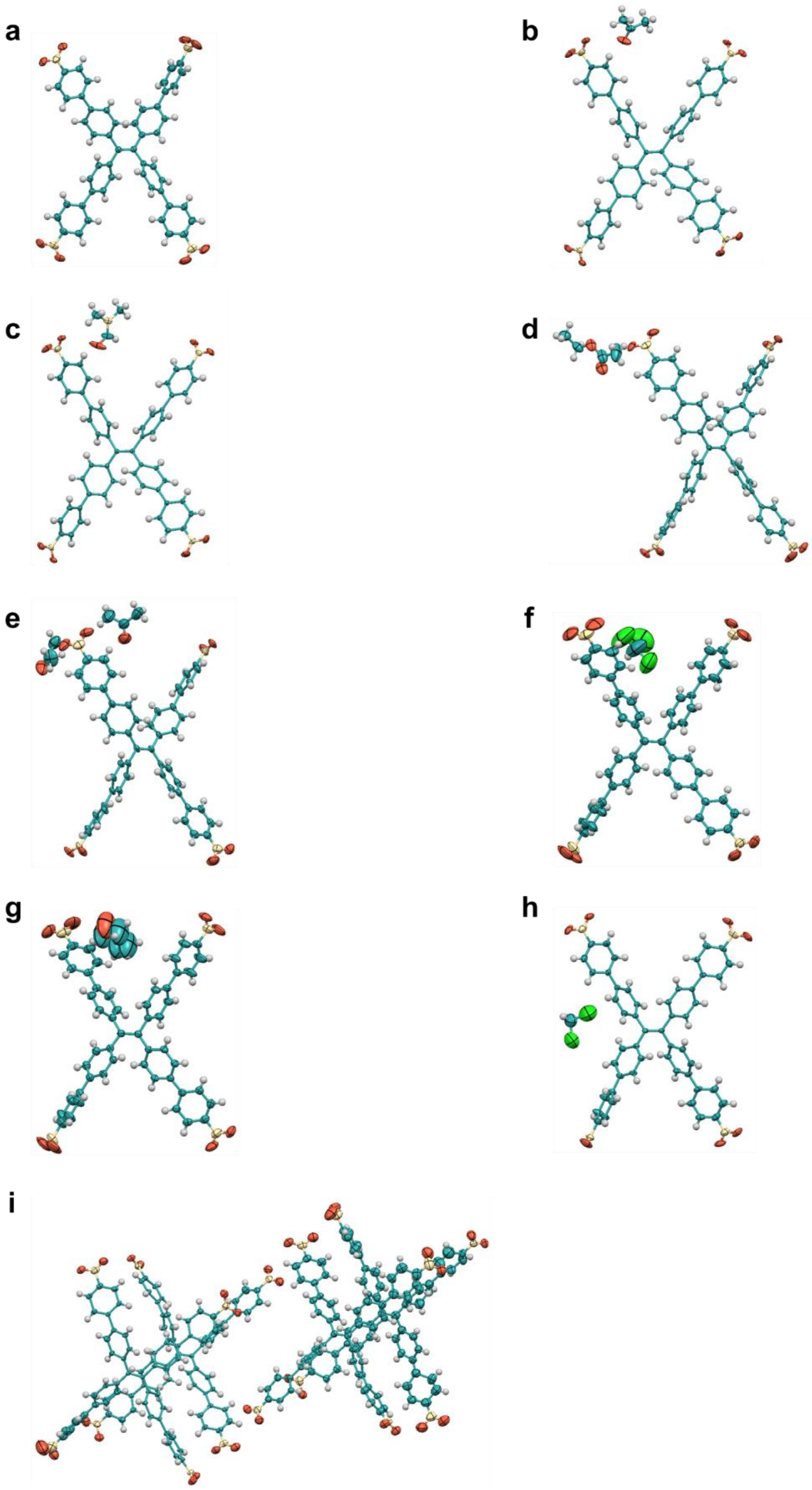

Supplementary Figure 32 | Structural figures with probability ellipsoids of **a,** 8PN-Heated; **b,** 8PN-ACT; **c,** 8PN-DMF; **d,** 8PN-EA; **e,** 8PN-2ACT; **f,** 8PN-TCM; **g,** 8PN-THF; **h,** 8PN-DCM and **i,** 8PN-TOL at the 50 % level. Color code: green, C; yellow, N; orange, O; grey, H; brilliant green, Cl.

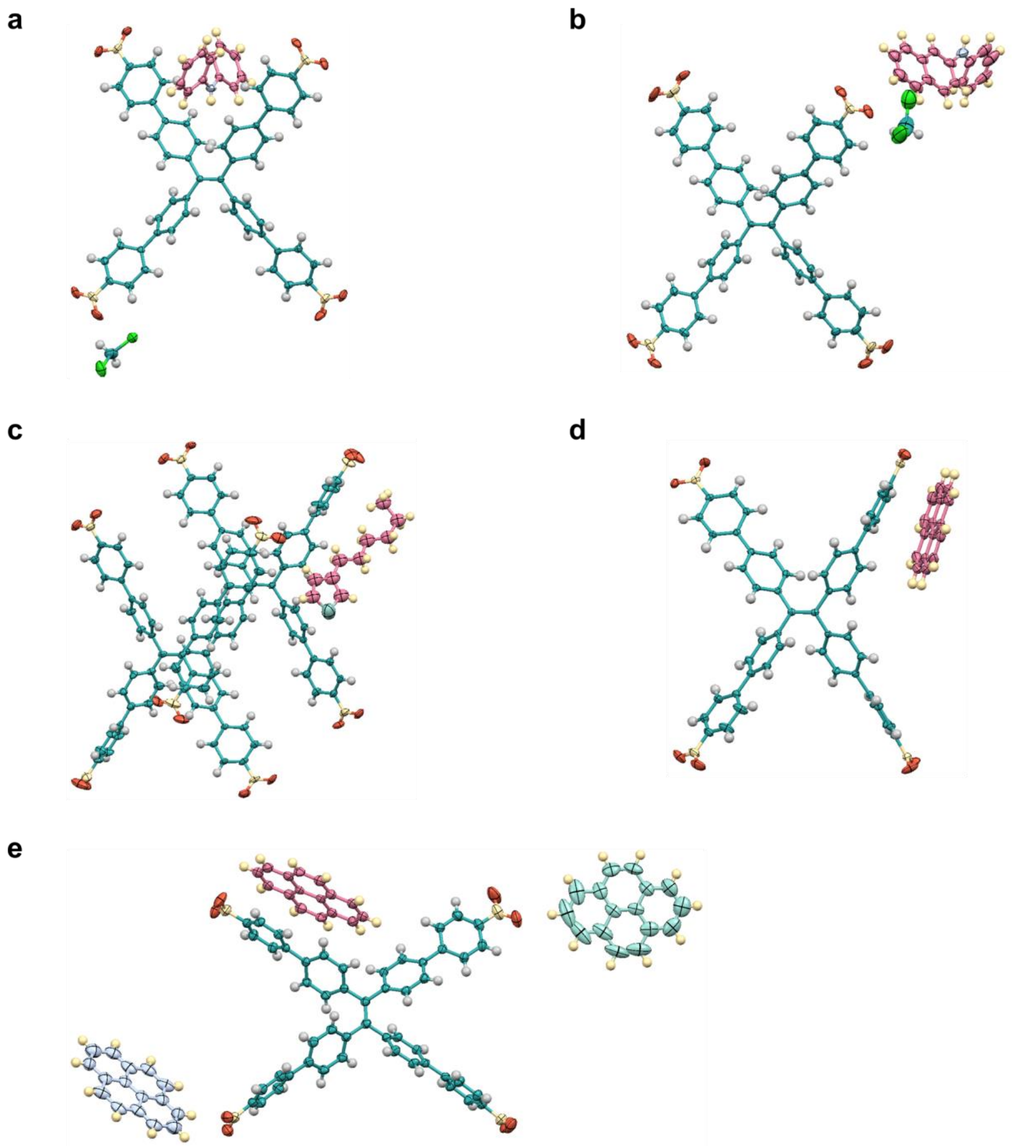

Supplementary Figure 33 | Structural figures with probability ellipsoids of **a,** 8PN-NDP; **b,** 8PN-NPNA; **c,** 8PN-SC6; **d,** 8PN-PY and **e,** 8PN-3PY at the 50 % level. Color code for TPE-4pn: green, C; yellow, N; orange, O; grey, H. Color code for the solvent molecule (dichloromethane) in 8PN-NDP and 8PN-NPNA: green, C; brilliant green, Cl; grey, H. Color code for diphenylamine, N-phenylnaphthalen-2-amine and 3-hexylthiophene: pink, C; blue, N; light green, S; yellow, H. Color code for pyrene in 8PN-PY: pink, C; yellow, H. H atoms of pyrene in 8PN-3PY are colored yellow and C atoms of the three pyrene molecules in the unit cell of 8PN-3PY are colored pink, light green and light blue respectively for clarity.

Supplementary Tables

Supplementary Table 1 | Crystallographic data for different frameworks of 8PN

HOFs	8PN-ACT	8PN-DMF	8PN-EA	8PN-2ACT	8PN-TCM	8PN-THF	8PN-DCM	8PN-TOL	8PN-Heated
**Formula**	$C_{53}H_{38}N_4O_9$	$C_{53}H_{39}N_5O_9$	$C_{54}H_{40}N_4O_{10}$	$C_{56}H_{44}N_4O_{10}$	$C_{51}H_{33}Cl_3N_4O_8$	$C_{54}H_{40}N_4O_9$	$C_{51}H_{34}Cl_2N_4O_8$	$C_{200}H_{128}N_{16}O_{32}$	$C_{50}H_{32}N_4O_8$
**Formula Weight**	874.87	889.89	904.90	932.95	936.16	888.90	901.72	3267.18	816.79
**Temperature (K)**	150.01(10)	150.00(10)	150.00(10)	150.00(10)	293(2)	276(12)	293(2)	150.00(10)	150.00(10)
**Crystal system**	triclinic	triclinic	triclinic	triclinic	monoclinic	monoclinic	monoclinic	triclinic	triclinic
**Space group**	P-1	P-1	P-1	P-1	$P2_1/n$	$P2_1/n$	$P2_1/c$	P1	P-1
a **(Å)**	9.9457(2)	9.9345(2)	12.9258(5)	12.8652(4)	21.1646(4)	21.1458(4)	16.3153(3)	9.6684(3)	11.9156(8)
b **(Å)**	12.9400(4)	12.8683(3)	14.7393(5)	15.0275(7)	9.2083(2)	9.2215(2)	8.8528(2)	14.9387(4)	13.5914(8)
c **(Å)**	17.1224(6)	17.4699(4)	14.7646(4)	15.0449(5)	22.8847(4)	22.8834(5)	32.2167(6)	38.2371(12)	13.7643(8)
**α (°)**	77.212(3)	75.903(2)	110.538(3)	112.104(4)	90	90	90	96.459(2)	104.460(5)
**β (°)**	84.983(2)	87.087(2)	97.904(3)	98.811(3)	92.391(2)	92.371(2)	97.492(2)	94.067(2)	93.273(5)
**γ (°)**	83.907(2)	83.146(2)	114.140(3)	111.543(4)	90	90	90	91.611(2)	106.577(5)
V **($Å^3$)**	2132.16(11)	2150.07(9)	2270.10(15)	2359.02(18)	4456.12(15)	4458.35(16)	4613.53(16)	5470.2(3)	2048.9(2)
Z	2	2	2	2	4	4	4	4	2
F **(000)**	912.0	928.0	944.0	976.0	1928.0	1856.0	1864.0	1696.0	848.0
**D_c ($g·cm^{-3}$)**	1.363	1.375	1.324	1.313	1.395	1.324	1.298	0.992	1.324
**Reflections collected**	14951	32209	23546	39513	14540	16324	20383	30863	13616
**Unique reflns**	8365	8545	8938	9428	7023	8672	9116	23153	8060
**Parameters**	597	606	615	635	595	580	586	2233	559
**R_{int}**	0.0263	0.0327	0.0309	0.0329	0.0283	0.0534	0.0313	0.0641	0.0324
**μ (mm^{-1})**	0.771	0.781	0.759	0.746	2.374	0.745	1.752	0.559	0.746
**R_1 [$I \geq 2\sigma(I)$] [a]**	0.0478	0.0410	0.0546	0.0802	0.0882	0.0823	0.1020	0.0858	0.0631
**wR_2 [$I \geq 2\sigma(I)$] [b]**	0.1262	0.1108	0.1509	0.2353	0.2660	0.2418	0.3002	0.2416	0.1684
**R_1 (all data)**	0.0543	0.0474	0.0677	0.0883	0.1027	0.0964	0.1192	0.0940	0.0908
**wR_2 (all data)**	0.1338	0.1167	0.1569	0.2462	0.2853	0.2748	0.3227	0.2556	0.1902
**GOF**	1.013	1.067	1.112	1.036	1.053	1.053	1.051	1.059	1.038

[a] $R_1=\Sigma||F_o|-|F_c||/\Sigma|F_o|$.

[b] $wR_2=[\Sigma w(F_o^2-F_c^2)^2/\Sigma w(F_o^2)^2]^{1/2}$.

Supplementary Table 2 | Crystallographic data for different host-guest systems

**Co-crystals**	**8PN-NDP**	**8PN-NPNA**	**8PN-SC6**	**8PN-PY**	**8PN-3PY**
**Formula**	$C_{64}H_{47}Cl_4N_5O_8$	$C_{67}H_{47}Cl_2N_5O_8$	$C_{110}H_{80}N_8O_{16}S$	$C_{66}H_{42}N_4O_8$	$C_{82}H_{52}N_4O_8$
**Formula weight**	1155.86	1120.99	1801.88	1019.03	1221.27
**Temperature (K)**	150.00(10)	150.00(10)	150.00(10)	149.99(10)	150.00(10)
**Crystal system**	monoclinic	monoclinic	triclinic	monoclinic	triclinic
**Space group**	*C*2/*c*	*Cc*	*P*1	$P2_1/n$	*P*-1
a **(Å)**	15.0398(2)	15.4523(5)	13.3288(3)	21.8778(3)	13.5717(6)
b **(Å)**	13.8135(2)	13.7876(3)	13.7815(5)	9.02760(10)	15.3754(6)
c **(Å)**	26.5612(3)	25.9476(7)	14.2147(5)	25.1726(3)	17.5775(9)
α **(°)**	90	90	115.048(4)	90	65.787(4)
β **(°)**	97.1480(10)	100.089(3)	99.908(2)	92.9490(10)	68.112(4)
γ **(°)**	90	90	97.246(2)	90	82.586(3)
**V (Å^3)**	5475.26(12)	5442.7(3)	2271.60(14)	4965.11(11)	3102.9(3)
Z	4	4	1	4	2
F **(000)**	2392.0	2328.0	940.0	2120.0	1272.0
**D_c (g·cm^{-3})**	1.402	1.368	1.317	1.363	1.307
**Reflections collected**	10016	10414	16148	18704	18577
**Unique reflns**	4935	6220	10523	9791	11897
**Parameters**	368	739	1163	703	847
**R_{int}**	0.0183	0.0200	0.0209	0.0379	0.0322
**μ/ mm^{-1}**	2.487	1.604	0.931	0.732	0.679
**R_1 [$I \geq 2\sigma(I)$] [a]**	0.0630	0.1061	0.0684	0.0440	0.0715
**wR_2 [$I \geq 2\sigma(I)$] [b]**	0.1783	0.2970	0.1895	0.1185	0.1999
**R_1 (all data)**	0.0668	0.1073	0.0795	0.0515	0.0840
**wR_2 (all data)**	0.1817	0.3012	0.2092	0.1239	0.2113
**GOF**	1.050	1.483	1.087	1.023	1.078

[a] $R_1 = \Sigma||F_o|-|F_c||/\Sigma|F_o|$.

[b] $wR_2 = [\Sigma w(F_o^2-F_c^2)^2/\Sigma w(F_o^2)^2]^{1/2}$.

Supplementary Table 3 | Data for voids

HOFs	*V* (Å^3) [a]	Ratio (%) [b]	Pore size (Å^2) [c]
**8PN-Heated**	89.4	4.4	6.232 × 9.624
**8PN-ACT**	222.1	10.4	6.621 × 9.892
**8PN-DMF**	255.1	11.9	6.709 × 10.253
**8PN-EA**	380.3	16.8	6.507 × 12.290
**8PN-2ACT**	491.5	20.8	8.679 × 12.912
**8PN-TCM**	649.6	14.6	7.547 × 13.106
**8PN-THF**	662.9	14.9	7.704 × 13.110
**8PN-DCM**	904.9	19.6	12.666 × 15.023
**8PN-TOL (L)**	1816.0	33.2	10.638 × 17.606
**8PN-TOL (N)**			9.143 × 18.582

[a] *V*: Solvent-accessible void space (Å^3).

[b] Ratio: Void space ratio (%).

[c] Pore size: Distance of atom centers including vdW radii (Å^2).

Supplementary Table 4 | Data of dihedral angles regulating void space among different types of 8PN-200, 8PN-400 and 8PN-600

Types	HOFs	A^X (°)	B^X (°)	C^Y (°)	D^Y (°)	E^X (°)	F^X (°)	G^Y (°)	H^Y (°)
8PN-200	8PN-ACT	55.13	29.53	44.06	46.65	26.32	65.95	18.65	3.47
	8PN-DMF	57.10	29.12	43.57	45.55	31.06	65.14	21.28	3.07
8PN-400	8PN-EA	50.13	44.04	43.61	44.72	88.34	20.07	17.95	75.23
	8PN-2ACT	49.64	43.37	41.19	45.60	88.35	17.16	15.56	79.03
8PN-600	8PN-TCM	56.80	40.45	55.53	39.40	44.66	22.04	29.68	65.42
	8PN-THF	56.41	38.27	55.95	40.55	31.26	63.47	45.19	22.21
	8PN-Heated	46.60	38.74	36.39	42.80	9.05	75.21	6.12	10.76

Supplementary Table 5 | Data of hydrogen bond distances regulating void space of the two frameworks within the same type, that is 8PN-ACT and 8PN-DMF in 8PN-200 type, 8PN-EA and 8PN-2ACT in 8PN-400 type or 8PN-TCM and 8PN-THF in 8PN-600 type

Types	HOFs	C-H···O (Å)	C-H···O (Å)	C-H···O (Å)	C-H···O (Å)
8PN-200	8PN-ACT	2.612 (×2)	2.478 (×2)	2.835 (×2)	2.626 (×2)
	8PN-DMF	2.622 (×2)	2.526 (×2)	2.922 (×2)	2.723 (×2)
8PN-400	8PN-EA	2.558 (×2)	2.901 (×2)	2.727 (×2)	2.921 (×2)
	8PN-2ACT	2.644 (×2)	3.002 (×2)	2.781 (×2)	3.529 (×2)
8PN-600	8PN-TCM	2.679 (×2)	3.149 (×2)	2.677 (×2)	2.556 (×2)
	8PN-THF	2.717 (×2)	3.121 (×2)	2.675 (×2)	2.531 (×2)

Supplementary Table 6 | Data of λ, τ, Φ_F, k_r and k_{nr} for 8PN

8PN	λ (nm)	τ (ns)	Φ_F	k_r (s^{-1}) [a]	k_{nr} (s^{-1}) [b]
**8PN-ACT**	530	1.75	0.14	5.71×10^8	3.51×10^9
**8PN-DMF**	518	0.93	0.2	1.08×10^9	4.30×10^9
**8PN-EA**	527	1.75	0.1	5.71×10^8	5.14×10^9
**8PN-2ACT** [c]	535	/	/	/	/
**8PN-TCM**	551	1.74	0.31	5.75×10^8	1.28×10^9
**8PN-THF**	550	1.72	0.55	5.81×10^8	4.76×10^8
**8PN-DCM**	563	2.09	0.3	4.78×10^8	1.12×10^9
**8PN-TOL**	529	2.37	0.31	4.22×10^8	9.39×10^8
**8PN-Heated**	531	0.93	0.1	1.08×10^9	9.68×10^9
**8PN-Ground**	580	4.4	0.29	2.27×10^8	5.56×10^8

[a] $k_r = 1/\tau$.

[b] $k_{nr} = k_r/\Phi_F - k_r$.

[c] Once the crystals of 8PN-2ACT were filtered from the solution, the solvent ACT molecules could easily escape from the voids of 8PN-2ACT at a fast rate, making 8PN-2ACT partially lose crystallinity, which resulted in changes of photoluminescent properties. So, τ, Φ_F, k_r and k_{nr} of 8PN-2ACT are not given. In addition, the gas adsorption measurement and the relative thermal analyses of 8PN-2ACT was not performed.

Supplementary Table 7 | Data of Intermolecular interactions in host-guest co-crystal structures

Co-crystal	N-H···C (Å)	C-H···C (Å)	C-H···O (Å)	C-H···π (Å)	π-π (Å)
**8PN-NDP**	2.873×2	2.808×2; 2.762×2; 2.885×2	/	/	/
**8PN-NPNA**	2.632; 2.895	2.724; 2.728; 2.746; 2.893; 2.834	/	/	/
**8PN-SC6**	/	2.866	/	/	/
**8PN-PY**	/	/	/	2.922	/
**8PN-3PY**	/	2.802; 2.846; 2.775; 2.891; 2.873	2.581	/	3.341×2

Supplementary Reference

1. Chang, Z. *et al.* Aggregation-enhanced emission and efficient electroluminescence of tetraphenylethene-cored luminogens. *Chem. Commun*. **49,** 594-596 (2013).
