# Supplementary material for: An exceptionally flexible hydrogen-bonded organic framework with large-scale void regulation and adaptive guest accommodation abilities
Source: Nat Commun. 2019 Jul 12;10:3074. doi: 10.1038/s41467-019-10575-5 (PMC6625987; doi:10.1038/s41467-019-10575-5)
Supplement: Supplementary file 3 — Source Data [file 41467_2019_10575_MOESM3_ESM.pdf]

Figure 4a

Relative pressure *P* /*P* 0	Uptake ($cm^3 \cdot g^{-1}$, STP)	Relative pressure *P* /*P* 0	Uptake ($cm^3 \cdot g^{-1}$, STP)	Relative pressure *P* /*P* 0	Uptake ($cm^3 \cdot g^{-1}$, STP)	Relative pressure *P* /*P* 0	Uptake ($cm^3 \cdot g^{-1}$, STP)
2.77E-04	1.6573	0.94719	106.9397	0.00565	0.67914	0.99208	7.22814
8.74E-04	3.7484	0.93026	107.41238	0.01151	0.80889	0.94495	3.77939
0.0013	5.879	0.8802	107.85399	0.023	0.91387	0.90678	3.10576
0.00176	8.00342	0.82566	107.6382	0.03451	0.996	0.87142	2.80409
0.00244	10.11271	0.77538	107.24767	0.04003	1.03216	0.83625	2.61914
0.00403	11.92759	0.72548	106.63511	0.05004	1.05876	0.80114	2.51146
0.00745	13.38048	0.67435	105.87803	0.08501	1.17233	0.76608	2.41749
0.0113	14.33333	0.62466	104.85176	0.11999	1.24865	0.73101	2.36356
0.0191	15.63356	0.57551	103.59128	0.15497	1.32397	0.69598	2.30769
0.02966	16.88451	0.52768	100.0041	0.19001	1.39158	0.66094	2.27105
0.03982	17.79732	0.48348	88.7294	0.22502	1.44482	0.62592	2.23588
0.04982	18.62523	0.46492	85.1034	0.26006	1.47082	0.59071	2.22283
0.06064	19.38014	0.42397	80.21876	0.31429	1.56513	0.55575	2.20113
0.06997	19.92655	0.37363	78.81555	0.34926	1.62967	0.52068	2.14883
0.08034	20.51859	0.32614	77.69686	0.38424	1.65517	0.48574	2.10906
0.09048	21.0507	0.28134	68.3947	0.41925	1.70116	0.45073	2.08895
0.1	21.53318	0.25977	56.8748	0.45419	1.74134	0.41571	2.05535
0.11042	22.03585	0.23898	45.86419	0.48923	1.77887	0.38074	2.00118
0.11991	22.53127	0.22275	39.96441	0.52423	1.83482	0.3458	1.96331
0.13024	23.02283	0.17261	32.15472	0.55907	1.85935	0.3108	1.93135
0.13992	23.46938	0.14377	30.16472	0.59416	1.91952	0.27585	1.88131
0.14985	23.90974	0.12851	29.23177	0.62912	1.9667	0.24083	1.82596
0.15998	24.31067	0.10044	27.50385	0.66422	1.99811	0.20588	1.77368
0.18049	25.0736	0.08048	26.00237	0.69898	2.05202	0.1709	1.72393
0.20076	25.7844	0.06013	24.18656	0.73395	2.13725	0.13595	1.64698
0.2254	26.55567	0.04994	23.14101	0.76896	2.19323	0.12057	1.62156
0.2504	27.36919	0.04012	21.99728	0.80388	2.29733	0.07245	1.5111
0.29827	29.52416	0.03009	20.72032	0.8389	2.40259	0.0501	1.43425
0.35081	31.60337	0.02057	19.06321	0.87382	2.57338		
0.40106	32.75513	0.00983	16.60129	0.89007	2.6939		
0.44974	33.58352			0.92492	3.0825		
0.50017	34.26182			0.95954	3.94462		
0.55035	34.79028			0.99208	7.22814		
0.60038	35.20679						
0.6504	35.64477						
0.70158	45.5503						
0.74262	57.43657						
0.75488	60.64166						
0.79589	74.03305						
0.84264	85.86659						

0.85657	87.89661
0.89643	98.31885
0.94719	106.9397

Figure 4b

Temp (°C)	Endo >
-0.153	-0.03
-0.053	-0.03
0.047	-0.02637
0.147	-0.02058
0.247	-0.01065
0.347	0.00281
0.447	0.01376
0.547	0.02702
0.647	0.03822
0.747	0.04918
0.847	0.06004
0.947	0.06948
1.047	0.0789
1.147	0.08765
1.247	0.09621
1.347	0.10392
1.447	0.11145
1.547	0.11839
1.647	0.12363
1.747	0.13085
1.847	0.13688
1.947	0.14308
2.047	0.14769
2.147	0.15293
2.247	0.15811
2.347	0.1638
2.447	0.16817
2.547	0.17246
2.647	0.17608
2.747	0.17985
2.847	0.18353
2.947	0.18728
3.047	0.19019
3.147	0.19306
3.247	0.19634
3.347	0.19826
3.447	0.19987
3.547	0.2038
3.647	0.20641
3.747	0.20844

Figure 4c

2ϑ (°)	Intensity (a.u.)	2ϑ (°)	Intensity (a.u.)	2ϑ (°)	Intensity (a.u.)	2ϑ (°)	Intensity (a.u.)	2ϑ (°)	Intensity (a.u.)
5	0	5	0.6	5	1.2	5	1.8	5	2.4
5.01969	0.00228	5.01969	0.59622	5.01969	1.19944	5.01969	1.80882	5.01969	2.39687
5.03938	0.00184	5.03938	0.59914	5.03938	1.20326	5.03938	1.79713	5.03938	2.40217
5.05907	0.00121	5.05907	0.60437	5.05907	1.20317	5.05907	1.80214	5.05907	2.39862
5.07876	0.00226	5.07876	0.6004	5.07876	1.20126	5.07876	1.80845	5.07876	2.39198
5.09844	-6.49E-04	5.09844	0.60864	5.09844	1.20003	5.09844	1.80908	5.09844	2.39485
5.11813	0.00546	5.11813	0.60148	5.11813	1.19905	5.11813	1.80596	5.11813	2.3973
5.13782	0.00807	5.13782	0.60183	5.13782	1.20592	5.13782	1.80534	5.13782	2.39684
5.15751	0.00166	5.15751	0.6038	5.15751	1.20113	5.15751	1.80459	5.15751	2.3968
5.1772	0.00446	5.1772	0.59777	5.1772	1.20366	5.1772	1.79991	5.1772	2.39895
5.19689	0	5.19689	0.6	5.19689	1.2	5.19689	1.80323	5.19689	2.4
5.21658	0.00487	5.21658	0.60314	5.21658	1.19925	5.21658	1.80305	5.21658	2.406
5.23627	9.86E-04	5.23627	0.59932	5.23627	1.20103	5.23627	1.80817	5.23627	2.40128
5.25596	0.00586	5.25596	0.60578	5.25596	1.19862	5.25596	1.79912	5.25596	2.40092
5.27565	0.008	5.27565	0.60121	5.27565	1.20205	5.27565	1.80537	5.27565	2.40256
5.29534	0.00691	5.29534	0.60773	5.29534	1.20781	5.29534	1.80262	5.29534	2.39953
5.31503	0.01405	5.31503	0.61599	5.31503	1.20623	5.31503	1.79738	5.31503	2.3979
5.33472	0.00906	5.33472	0.61068	5.33472	1.20899	5.33472	1.80707	5.33472	2.39826
5.3544	0.01555	5.3544	0.61876	5.3544	1.20974	5.3544	1.8097	5.3544	2.39372
5.37409	0.02042	5.37409	0.61858	5.37409	1.2063	5.37409	1.81414	5.37409	2.39688
5.39378	0.01303	5.39378	0.63342	5.39378	1.21407	5.39378	1.82083	5.39378	2.40542
5.41347	0.02355	5.41347	0.63774	5.41347	1.21807	5.41347	1.82521	5.41347	2.39155
5.43316	0.02705	5.43316	0.64676	5.43316	1.22687	5.43316	1.83052	5.43316	2.40107
5.45285	0.0434	5.45285	0.66285	5.45285	1.22787	5.45285	1.83528	5.45285	2.39992
5.47254	0.03978	5.47254	0.67118	5.47254	1.23497	5.47254	1.85353	5.47254	2.39713
5.49223	0.04873	5.49223	0.69715	5.49223	1.23948	5.49223	1.86728	5.49223	2.40083
5.51192	0.06262	5.51192	0.71287	5.51192	1.25283	5.51192	1.86666	5.51192	2.40399
5.53161	0.06554	5.53161	0.71769	5.53161	1.24175	5.53161	1.85535	5.53161	2.4006
5.55129	0.07171	5.55129	0.71401	5.55129	1.24131	5.55129	1.83905	5.55129	2.39981
5.57098	0.06387	5.57098	0.70437	5.57098	1.22888	5.57098	1.82149	5.57098	2.40048
5.59067	0.06005	5.59067	0.68573	5.59067	1.22658	5.59067	1.82212	5.59067	2.4
5.61036	0.05448	5.61036	0.67472	5.61036	1.21623	5.61036	1.82013	5.61036	2.39834
5.63005	0.04398	5.63005	0.67836	5.63005	1.21811	5.63005	1.82794	5.63005	2.39808
5.64974	0.0493	5.64974	0.68707	5.64974	1.21493	5.64974	1.83226	5.64974	2.40091
5.66943	0.05709	5.66943	0.70466	5.66943	1.2292	5.66943	1.84176	5.66943	2.40234
5.68912	0.07655	5.68912	0.73701	5.68912	1.22861	5.68912	1.85489	5.68912	2.39353
5.70881	0.09653	5.70881	0.77776	5.70881	1.23906	5.70881	1.86545	5.70881	2.39745
5.7285	0.1193	5.7285	0.81588	5.7285	1.25168	5.7285	1.89414	5.7285	2.40355
5.74819	0.14615	5.74819	0.85631	5.74819	1.26167	5.74819	1.91639	5.74819	2.40541
5.76788	0.17534	5.76788	0.90394	5.76788	1.28085	5.76788	1.95776	5.76788	2.39308

3.847	0.21098	5.78756	0.19117	5.78756	0.95957	5.78756	1.31175	5.78756	1.99332	5.78756	2.40403
3.947	0.21273	5.80725	0.20149	5.80725	0.96934	5.80725	1.33372	5.80725	2.0575	5.80725	2.39365
4.047	0.21501	5.82694	0.20772	5.82694	0.95264	5.82694	1.37082	5.82694	2.13186	5.82694	2.40908
4.147	0.21722	5.84663	0.18833	5.84663	0.89983	5.84663	1.42408	5.84663	2.19579	5.84663	2.40815
4.247	0.21894	5.86632	0.13932	5.86632	0.82693	5.86632	1.48354	5.86632	2.23979	5.86632	2.40455
4.347	0.2202	5.88601	0.09866	5.88601	0.75523	5.88601	1.54857	5.88601	2.23748	5.88601	2.40108
4.447	0.2218	5.9057	0.06125	5.9057	0.70642	5.9057	1.62057	5.9057	2.13488	5.9057	2.40402
4.547	0.22376	5.92539	0.04148	5.92539	0.662	5.92539	1.64915	5.92539	2.01322	5.92539	2.40188
4.647	0.22539	5.94508	0.02093	5.94508	0.64342	5.94508	1.6437	5.94508	1.89	5.94508	2.39926
4.747	0.22606	5.96477	0.01121	5.96477	0.63272	5.96477	1.57933	5.96477	1.84495	5.96477	2.40354
4.847	0.22679	5.98445	0.01323	5.98445	0.62547	5.98445	1.49245	5.98445	1.81702	5.98445	2.4
4.947	0.22784	6.00414	0.00695	6.00414	0.61377	6.00414	1.37996	6.00414	1.80796	6.00414	2.40029
5.047	0.22911	6.02383	0.01383	6.02383	0.61516	6.02383	1.29504	6.02383	1.80528	6.02383	2.40082
5.147	0.23059	6.04352	0.01091	6.04352	0.61523	6.04352	1.24745	6.04352	1.80884	6.04352	2.40384
5.247	0.23201	6.06321	0.00119	6.06321	0.61974	6.06321	1.22284	6.06321	1.8026	6.06321	2.40522
5.347	0.23241	6.0829	0.00626	6.0829	0.60798	6.0829	1.21822	6.0829	1.80716	6.0829	2.40442
5.447	0.23315	6.10259	0.01022	6.10259	0.60673	6.10259	1.20683	6.10259	1.80598	6.10259	2.40434
5.547	0.23406	6.12228	0.00303	6.12228	0.60305	6.12228	1.20835	6.12228	1.80399	6.12228	2.40324
5.647	0.23508	6.14197	1.17E-04	6.14197	0.59861	6.14197	1.20703	6.14197	1.80893	6.14197	2.40941
5.747	0.23579	6.16166	0.01017	6.16166	0.5953	6.16166	1.20421	6.16166	1.80631	6.16166	2.40388
5.847	0.23616	6.18135	0	6.18135	0.60019	6.18135	1.2	6.18135	1.8	6.18135	2.40459
5.947	0.23641	6.20104	0.01646	6.20104	0.6027	6.20104	1.20293	6.20104	1.80155	6.20104	2.41033
6.047	0.23695	6.22072	0.00952	6.22072	0.60877	6.22072	1.20358	6.22072	1.79835	6.22072	2.41723
6.147	0.23825	6.24041	0.00225	6.24041	0.60734	6.24041	1.2032	6.24041	1.80915	6.24041	2.41134
6.247	0.23809	6.2601	0.0049	6.2601	0.60628	6.2601	1.20262	6.2601	1.80151	6.2601	2.42096
6.347	0.2392	6.27979	0.00612	6.27979	0.60479	6.27979	1.20224	6.27979	1.80518	6.27979	2.42204
6.447	0.23947	6.29948	0.01558	6.29948	0.60799	6.29948	1.19417	6.29948	1.80442	6.29948	2.43863
6.547	0.23959	6.31917	0.01208	6.31917	0.60568	6.31917	1.20092	6.31917	1.81428	6.31917	2.44765
6.647	0.23975	6.33886	0.00597	6.33886	0.60481	6.33886	1.1961	6.33886	1.81583	6.33886	2.45309
6.747	0.23983	6.35855	0.00363	6.35855	0.59987	6.35855	1.20713	6.35855	1.81956	6.35855	2.47168
6.847	0.24075	6.37824	0.00116	6.37824	0.6	6.37824	1.20128	6.37824	1.8078	6.37824	2.48397
6.947	0.24084	6.39793	-0.00222	6.39793	0.6064	6.39793	1.19904	6.39793	1.8161	6.39793	2.50765
7.047	0.24079	6.41761	0.00873	6.41761	0.60974	6.41761	1.20063	6.41761	1.8239	6.41761	2.52043
7.147	0.24112	6.4373	0.00503	6.4373	0.60463	6.4373	1.20025	6.4373	1.81182	6.4373	2.54338
7.247	0.24105	6.45699	0.01066	6.45699	0.6049	6.45699	1.19791	6.45699	1.82631	6.45699	2.5607
7.347	0.24122	6.47668	0.00774	6.47668	0.61312	6.47668	1.19376	6.47668	1.82386	6.47668	2.55524
7.447	0.24177	6.49637	0.00779	6.49637	0.60375	6.49637	1.20159	6.49637	1.8196	6.49637	2.54292
7.547	0.24187	6.51606	0.00526	6.51606	0.60296	6.51606	1.19672	6.51606	1.81358	6.51606	2.51327
7.647	0.2415	6.53575	0.0105	6.53575	0.61249	6.53575	1.20083	6.53575	1.81982	6.53575	2.48998
7.747	0.24145	6.55544	0.00764	6.55544	0.60932	6.55544	1.2003	6.55544	1.82155	6.55544	2.45421
7.847	0.24159	6.57513	0.00835	6.57513	0.61047	6.57513	1.20354	6.57513	1.80848	6.57513	2.43692
7.947	0.24167	6.59482	0.00679	6.59482	0.60367	6.59482	1.19598	6.59482	1.8144	6.59482	2.42406
8.047	0.24195	6.61451	0.00924	6.61451	0.60669	6.61451	1.20872	6.61451	1.80777	6.61451	2.41362
8.147	0.24195	6.6342	0.00658	6.6342	0.61015	6.6342	1.1992	6.6342	1.80457	6.6342	2.41719

8.247	0.24167	6.65388	0.01422	6.65388	0.60905	6.65388	1.20115	6.65388	1.8068	6.65388	2.4062
8.347	0.24175	6.67357	0.00721	6.67357	0.60513	6.67357	1.20087	6.67357	1.80666	6.67357	2.4151
8.447	0.24172	6.69326	0.01181	6.69326	0.60264	6.69326	1.20576	6.69326	1.80615	6.69326	2.41393
8.547	0.24127	6.71295	0.01569	6.71295	0.61042	6.71295	1.20172	6.71295	1.80264	6.71295	2.4078
8.647	0.24137	6.73264	0.01114	6.73264	0.59887	6.73264	1.20655	6.73264	1.80231	6.73264	2.41481
8.747	0.24148	6.75233	0.0088	6.75233	0.60658	6.75233	1.20576	6.75233	1.80699	6.75233	2.40662
8.847	0.24136	6.77202	0.01489	6.77202	0.60604	6.77202	1.20373	6.77202	1.8091	6.77202	2.42012
8.947	0.24121	6.79171	0.01041	6.79171	0.61076	6.79171	1.20531	6.79171	1.8039	6.79171	2.41859
9.047	0.24108	6.8114	0.01138	6.8114	0.61003	6.8114	1.19915	6.8114	1.79739	6.8114	2.41416
9.147	0.24123	6.83109	0.00631	6.83109	0.61568	6.83109	1.20322	6.83109	1.80587	6.83109	2.41911
9.247	0.24108	6.85077	0.00831	6.85077	0.60888	6.85077	1.20253	6.85077	1.80448	6.85077	2.4237
9.347	0.24082	6.87046	0.00902	6.87046	0.60921	6.87046	1.21041	6.87046	1.80822	6.87046	2.42181
9.447	0.24086	6.89015	0.01647	6.89015	0.61111	6.89015	1.20218	6.89015	1.80608	6.89015	2.41798
9.547	0.24087	6.90984	0.01024	6.90984	0.611	6.90984	1.20454	6.90984	1.80613	6.90984	2.42057
9.647	0.24059	6.92953	0.01049	6.92953	0.61397	6.92953	1.21098	6.92953	1.80393	6.92953	2.41262
9.747	0.24054	6.94922	0.01385	6.94922	0.61117	6.94922	1.20606	6.94922	1.80604	6.94922	2.40909
9.847	0.24049	6.96891	0.01929	6.96891	0.6147	6.96891	1.20759	6.96891	1.80928	6.96891	2.41302
9.947	0.24017	6.9886	0.01825	6.9886	0.61422	6.9886	1.20566	6.9886	1.80364	6.9886	2.407
10.047	0.23994	7.00829	0.01481	7.00829	0.61411	7.00829	1.20921	7.00829	1.80744	7.00829	2.4062
10.147	0.23987	7.02798	0.01655	7.02798	0.61319	7.02798	1.20775	7.02798	1.80367	7.02798	2.41091
10.247	0.23969	7.04767	0.01758	7.04767	0.61691	7.04767	1.20561	7.04767	1.80553	7.04767	2.4132
10.347	0.23964	7.06736	0.019	7.06736	0.61367	7.06736	1.20518	7.06736	1.80758	7.06736	2.4064
10.447	0.2393	7.08704	0.01212	7.08704	0.60399	7.08704	1.20713	7.08704	1.80345	7.08704	2.40657
10.547	0.23956	7.10673	0.01743	7.10673	0.60477	7.10673	1.20716	7.10673	1.80293	7.10673	2.40716
10.647	0.23868	7.12642	0.00685	7.12642	0.61386	7.12642	1.20689	7.12642	1.80648	7.12642	2.40345
10.747	0.23876	7.14611	0.01294	7.14611	0.61063	7.14611	1.20661	7.14611	1.80822	7.14611	2.41168
10.847	0.23915	7.1658	0.01948	7.1658	0.61403	7.1658	1.20613	7.1658	1.81389	7.1658	2.40912
10.947	0.23908	7.18549	0.01202	7.18549	0.6143	7.18549	1.20348	7.18549	1.80525	7.18549	2.4139
11.047	0.23884	7.20518	0.01642	7.20518	0.60694	7.20518	1.2045	7.20518	1.80724	7.20518	2.40934
11.147	0.23896	7.22487	0.01466	7.22487	0.61303	7.22487	1.21011	7.22487	1.80979	7.22487	2.4126
11.247	0.23867	7.24456	0.01498	7.24456	0.60967	7.24456	1.2089	7.24456	1.80528	7.24456	2.40682
11.347	0.2387	7.26425	0.01582	7.26425	0.61714	7.26425	1.20543	7.26425	1.81301	7.26425	2.41778
11.447	0.23876	7.28394	0.00712	7.28394	0.61303	7.28394	1.20025	7.28394	1.80275	7.28394	2.40625
11.547	0.23884	7.30363	0.00543	7.30363	0.60879	7.30363	1.20281	7.30363	1.80886	7.30363	2.40545
11.647	0.23822	7.32331	0.01437	7.32331	0.60956	7.32331	1.21023	7.32331	1.81122	7.32331	2.40798
11.747	0.23809	7.343	0.00788	7.343	0.6109	7.343	1.20768	7.343	1.80377	7.343	2.40633
11.847	0.2383	7.36269	0.01066	7.36269	0.60961	7.36269	1.20338	7.36269	1.81132	7.36269	2.40783
11.947	0.23797	7.38238	0.01785	7.38238	0.60762	7.38238	1.20683	7.38238	1.79893	7.38238	2.40733
12.047	0.23797	7.40207	0.0135	7.40207	0.61165	7.40207	1.21626	7.40207	1.80273	7.40207	2.40199
12.147	0.23807	7.42176	0.01388	7.42176	0.6133	7.42176	1.20798	7.42176	1.81072	7.42176	2.40785
12.247	0.23796	7.44145	0.0092	7.44145	0.60944	7.44145	1.20445	7.44145	1.80489	7.44145	2.41087
12.347	0.23778	7.46114	0.00913	7.46114	0.6032	7.46114	1.20975	7.46114	1.80494	7.46114	2.40425
12.447	0.23721	7.48083	0.01341	7.48083	0.60873	7.48083	1.20793	7.48083	1.79949	7.48083	2.40205
12.547	0.23743	7.50052	0.00575	7.50052	0.60456	7.50052	1.20585	7.50052	1.80441	7.50052	2.4041

12.647	0.23758	7.5202	0.01047	7.5202	0.60834	7.5202	1.20573	7.5202	1.7959	7.5202	2.40839
12.747	0.23785	7.53989	0.00392	7.53989	0.60742	7.53989	1.2006	7.53989	1.80639	7.53989	2.41353
12.847	0.2376	7.55958	0.00825	7.55958	0.61182	7.55958	1.20419	7.55958	1.8	7.55958	2.4
12.947	0.23756	7.57927	0.00974	7.57927	0.6109	7.57927	1.20774	7.57927	1.79574	7.57927	2.4115
13.047	0.23761	7.59896	0.01038	7.59896	0.60986	7.59896	1.20494	7.59896	1.80635	7.59896	2.40609
13.147	0.23753	7.61865	0.00888	7.61865	0.60675	7.61865	1.20853	7.61865	1.80146	7.61865	2.40601
13.247	0.2378	7.63834	0.00609	7.63834	0.60271	7.63834	1.20232	7.63834	1.80113	7.63834	2.40291
13.347	0.23767	7.65803	0.00679	7.65803	0.60673	7.65803	1.20742	7.65803	1.80511	7.65803	2.39907
13.447	0.23744	7.67772	0.00147	7.67772	0.60876	7.67772	1.20224	7.67772	1.80304	7.67772	2.40184
13.547	0.23785	7.69741	0.0097	7.69741	0.60308	7.69741	1.20377	7.69741	1.80096	7.69741	2.40389
13.647	0.23773	7.7171	0.01131	7.7171	0.60905	7.7171	1.21062	7.7171	1.8057	7.7171	2.40381
13.747	0.23753	7.73679	0.00618	7.73679	0.61151	7.73679	1.20487	7.73679	1.80237	7.73679	2.40416
13.847	0.23769	7.75647	0.0052	7.75647	0.60959	7.75647	1.2	7.75647	1.80567	7.75647	2.40578
13.947	0.23796	7.77616	0.01213	7.77616	0.60498	7.77616	1.20265	7.77616	1.80515	7.77616	2.40231
14.047	0.23796	7.79585	0.00713	7.79585	0.60845	7.79585	1.20117	7.79585	1.79895	7.79585	2.40436
14.147	0.23808	7.81554	0.00252	7.81554	0.60865	7.81554	1.20155	7.81554	1.80343	7.81554	2.39483
14.247	0.23794	7.83523	0.00511	7.83523	0.60818	7.83523	1.20472	7.83523	1.80336	7.83523	2.40287
14.347	0.23789	7.85492	-0.00164	7.85492	0.60357	7.85492	1.20908	7.85492	1.80503	7.85492	2.40189
14.447	0.23812	7.87461	0.00918	7.87461	0.60959	7.87461	1.20868	7.87461	1.80095	7.87461	2.3986
14.547	0.23822	7.8943	0.00113	7.8943	0.60661	7.8943	1.20963	7.8943	1.801	7.8943	2.40319
14.647	0.23838	7.91399	0.00502	7.91399	0.60382	7.91399	1.20743	7.91399	1.80436	7.91399	2.4059
14.747	0.23872	7.93368	7.31E-04	7.93368	0.60384	7.93368	1.20492	7.93368	1.79972	7.93368	2.40437
14.847	0.23839	7.95336	0.00306	7.95336	0.60655	7.95336	1.20323	7.95336	1.80015	7.95336	2.4035
14.947	0.23852	7.97305	0.00143	7.97305	0.60908	7.97305	1.20552	7.97305	1.79713	7.97305	2.40294
15.047	0.23865	7.99274	0.00512	7.99274	0.60979	7.99274	1.20466	7.99274	1.79574	7.99274	2.39947
15.147	0.23884	8.01243	0.00109	8.01243	0.61237	8.01243	1.19998	8.01243	1.80279	8.01243	2.40237
15.247	0.23914	8.03212	-6.65E-04	8.03212	0.61133	8.03212	1.19825	8.03212	1.80371	8.03212	2.40532
15.347	0.23922	8.05181	0.00419	8.05181	0.6173	8.05181	1.1977	8.05181	1.79864	8.05181	2.39954
15.447	0.23891	8.0715	-0.00159	8.0715	0.62258	8.0715	1.20897	8.0715	1.80268	8.0715	2.39789
15.547	0.2387	8.09119	0.00502	8.09119	0.62735	8.09119	1.19861	8.09119	1.80411	8.09119	2.40588
15.647	0.2386	8.11088	0.00696	8.11088	0.63858	8.11088	1.20126	8.11088	1.80803	8.11088	2.40477
15.747	0.23871	8.13057	0.00793	8.13057	0.64942	8.13057	1.19777	8.13057	1.80495	8.13057	2.40197
15.847	0.239	8.15026	0.01687	8.15026	0.65983	8.15026	1.2045	8.15026	1.81	8.15026	2.40013
15.947	0.23881	8.16995	0.02192	8.16995	0.67262	8.16995	1.20566	8.16995	1.81567	8.16995	2.40612
16.047	0.239	8.18963	0.0236	8.18963	0.68741	8.18963	1.20464	8.18963	1.82053	8.18963	2.40786
16.147	0.23915	8.20932	0.0341	8.20932	0.70589	8.20932	1.20673	8.20932	1.82233	8.20932	2.41233
16.247	0.23902	8.22901	0.042	8.22901	0.72106	8.22901	1.20664	8.22901	1.837	8.22901	2.40765
16.347	0.23947	8.2487	0.05568	8.2487	0.74755	8.2487	1.21002	8.2487	1.8398	8.2487	2.41108
16.447	0.23933	8.26839	0.0604	8.26839	0.75258	8.26839	1.21344	8.26839	1.83922	8.26839	2.42083
16.547	0.23936	8.28808	0.07434	8.28808	0.76768	8.28808	1.21563	8.28808	1.83614	8.28808	2.41414
16.647	0.23955	8.30777	0.08568	8.30777	0.75419	8.30777	1.21451	8.30777	1.82494	8.30777	2.42534
16.747	0.23943	8.32746	0.08807	8.32746	0.71385	8.32746	1.21397	8.32746	1.81799	8.32746	2.42248
16.847	0.23972	8.34715	0.08807	8.34715	0.67864	8.34715	1.21305	8.34715	1.81873	8.34715	2.43167
16.947	0.23978	8.36684	0.0819	8.36684	0.64868	8.36684	1.20755	8.36684	1.80928	8.36684	2.42105

17.047	0.23957	8.38653	0.0531	8.38653	0.63143	8.38653	1.20529	8.38653	1.80276	8.38653	2.41619
17.147	0.2399	8.40621	0.04071	8.40621	0.60724	8.40621	1.20542	8.40621	1.80356	8.40621	2.42332
17.247	0.23994	8.4259	0.02663	8.4259	0.60457	8.4259	1.19996	8.4259	1.80486	8.4259	2.40973
17.347	0.23967	8.44559	0.00568	8.44559	0.60728	8.44559	1.20215	8.44559	1.80578	8.44559	2.41129
17.447	0.23982	8.46528	0.0071	8.46528	0.60042	8.46528	1.19928	8.46528	1.80464	8.46528	2.4043
17.547	0.23975	8.48497	0.00352	8.48497	0.60482	8.48497	1.19707	8.48497	1.80806	8.48497	2.40441
17.647	0.24006	8.50466	0.00248	8.50466	0.59777	8.50466	1.2021	8.50466	1.80624	8.50466	2.40191
17.747	0.24022	8.52435	0.00519	8.52435	0.60643	8.52435	1.20227	8.52435	1.80322	8.52435	2.4002
17.847	0.24037	8.54404	0	8.54404	0.60301	8.54404	1.20002	8.54404	1.80233	8.54404	2.4
17.947	0.24038	8.56373	0.00382	8.56373	0.59965	8.56373	1.20159	8.56373	1.79919	8.56373	2.40476
18.047	0.24021	8.58342	-3.29E-04	8.58342	0.60524	8.58342	1.19779	8.58342	1.80143	8.58342	2.40218
18.147	0.24002	8.60311	-0.00455	8.60311	0.59732	8.60311	1.19817	8.60311	1.8016	8.60311	2.39954
18.247	0.24035	8.62279	-0.00137	8.62279	0.6046	8.62279	1.2004	8.62279	1.80165	8.62279	2.40472
18.347	0.2403	8.64248	-0.00144	8.64248	0.6013	8.64248	1.19511	8.64248	1.80176	8.64248	2.40487
18.447	0.24025	8.66217	0.00271	8.66217	0.60376	8.66217	1.19941	8.66217	1.80425	8.66217	2.39563
18.547	0.24041	8.68186	-0.00397	8.68186	0.60128	8.68186	1.19969	8.68186	1.79686	8.68186	2.40269
18.647	0.24058	8.70155	-2.12E-04	8.70155	0.59817	8.70155	1.20626	8.70155	1.80522	8.70155	2.39866
18.747	0.2404	8.72124	-0.00216	8.72124	0.60427	8.72124	1.2004	8.72124	1.79989	8.72124	2.40232
18.847	0.24051	8.74093	0.00225	8.74093	0.60166	8.74093	1.2	8.74093	1.8	8.74093	2.40126
18.947	0.24038	8.76062	6.25E-04	8.76062	0.60049	8.76062	1.19745	8.76062	1.80501	8.76062	2.39905
19.047	0.24047	8.78031	8.82E-04	8.78031	0.60396	8.78031	1.1998	8.78031	1.80715	8.78031	2.40417
19.147	0.24036	8.8	0.00308	8.8	0.60429	8.8	1.20324	8.8	1.80254	8.8	2.39559
19.247	0.24059	8.81968	-0.00353	8.81968	0.60619	8.81968	1.20085	8.81968	1.80662	8.81968	2.39889
19.347	0.24064	8.83938	-0.00315	8.83938	0.60458	8.83938	1.20269	8.83938	1.80776	8.83938	2.40243
19.447	0.24052	8.85906	-0.0047	8.85906	0.60767	8.85906	1.20287	8.85906	1.8024	8.85906	2.40143
19.547	0.24045	8.87875	-0.00717	8.87875	0.60519	8.87875	1.19743	8.87875	1.8086	8.87875	2.39389
19.647	0.24058	8.89844	-0.00776	8.89844	0.60621	8.89844	1.1986	8.89844	1.80505	8.89844	2.39955
19.747	0.24015	8.91813	0.00152	8.91813	0.60811	8.91813	1.19651	8.91813	1.80362	8.91813	2.40304
19.847	0.24052	8.93782	5.40E-04	8.93782	0.6	8.93782	1.20449	8.93782	1.80451	8.93782	2.40125
19.947	0.24065	8.95751	-0.00536	8.95751	0.60501	8.95751	1.19631	8.95751	1.80803	8.95751	2.40097
20.047	0.2409	8.9772	-0.00588	8.9772	0.60295	8.9772	1.20218	8.9772	1.8011	8.9772	2.40264
20.147	0.241	8.99689	-0.00154	8.99689	0.60164	8.99689	1.20092	8.99689	1.8043	8.99689	2.40206
20.247	0.24098	9.01658	0.00222	9.01658	0.60684	9.01658	1.19889	9.01658	1.80163	9.01658	2.40524
20.347	0.24083	9.03627	0.00241	9.03627	0.60396	9.03627	1.19948	9.03627	1.80527	9.03627	2.40133
20.447	0.24105	9.05596	-0.00317	9.05596	0.60422	9.05596	1.19941	9.05596	1.80447	9.05596	2.40136
20.547	0.24139	9.07564	-3.83E-04	9.07564	0.60147	9.07564	1.198	9.07564	1.80236	9.07564	2.40339
20.647	0.24151	9.09533	-0.00272	9.09533	0.60673	9.09533	1.20247	9.09533	1.80037	9.09533	2.40184
20.747	0.24158	9.11502	-0.0022	9.11502	0.60642	9.11502	1.20172	9.11502	1.80495	9.11502	2.40454
20.847	0.24165	9.13471	0.00279	9.13471	0.60724	9.13471	1.20021	9.13471	1.80659	9.13471	2.40184
20.947	0.24165	9.1544	0.00421	9.1544	0.60055	9.1544	1.20029	9.1544	1.80298	9.1544	2.40084
21.047	0.24166	9.17409	-0.00124	9.17409	0.60124	9.17409	1.19831	9.17409	1.80524	9.17409	2.39717
21.147	0.24139	9.19378	5.77E-04	9.19378	0.60143	9.19378	1.19663	9.19378	1.80463	9.19378	2.39993
21.247	0.24158	9.21347	7.05E-04	9.21347	0.60438	9.21347	1.19636	9.21347	1.80014	9.21347	2.40098
21.347	0.24195	9.23316	-0.00338	9.23316	0.6035	9.23316	1.20744	9.23316	1.80241	9.23316	2.39823

21.447	0.24218	9.25285	0.00693	9.25285	0.60107	9.25285	1.20421	9.25285	1.79892	9.25285	2.40407
21.547	0.24196	9.27253	-1.45E-04	9.27253	0.6002	9.27253	1.19908	9.27253	1.80368	9.27253	2.40065
21.647	0.24192	9.29222	-0.00709	9.29222	0.60665	9.29222	1.19746	9.29222	1.80014	9.29222	2.39662
21.747	0.24242	9.31191	-0.00151	9.31191	0.6064	9.31191	1.2058	9.31191	1.79884	9.31191	2.39416
21.847	0.24234	9.3316	0.00439	9.3316	0.60265	9.3316	1.2	9.3316	1.80029	9.3316	2.40092
21.947	0.24231	9.35129	0.00186	9.35129	0.60071	9.35129	1.2027	9.35129	1.8008	9.35129	2.39822
22.047	0.24224	9.37098	0.00108	9.37098	0.60547	9.37098	1.2006	9.37098	1.8015	9.37098	2.40037
22.147	0.24228	9.39067	-0.00807	9.39067	0.60542	9.39067	1.20464	9.39067	1.8017	9.39067	2.39646
22.247	0.24278	9.41036	5.53E-04	9.41036	0.60217	9.41036	1.19954	9.41036	1.80578	9.41036	2.39982
22.347	0.24291	9.43005	0.00587	9.43005	0.60117	9.43005	1.20063	9.43005	1.81185	9.43005	2.40155
22.447	0.24291	9.44974	0.00768	9.44974	0.60399	9.44974	1.2007	9.44974	1.80806	9.44974	2.40012
22.547	0.24315	9.46943	-2.31E-04	9.46943	0.60218	9.46943	1.19942	9.46943	1.81051	9.46943	2.40148
22.647	0.24311	9.48912	9.34E-04	9.48912	0.60512	9.48912	1.19758	9.48912	1.80027	9.48912	2.39739
22.747	0.24323	9.5088	0.00372	9.5088	0.60087	9.5088	1.19899	9.5088	1.80397	9.5088	2.39815
22.847	0.24337	9.52849	0.00171	9.52849	0.60663	9.52849	1.20081	9.52849	1.80686	9.52849	2.40296
22.947	0.24301	9.54818	-6.94E-04	9.54818	0.60457	9.54818	1.20185	9.54818	1.79919	9.54818	2.39796
23.047	0.24331	9.56787	-0.00154	9.56787	0.60558	9.56787	1.20336	9.56787	1.80189	9.56787	2.39635
23.147	0.2434	9.58756	0.003	9.58756	0.60539	9.58756	1.19837	9.58756	1.80709	9.58756	2.3985
23.247	0.24358	9.60725	0.00319	9.60725	0.60296	9.60725	1.19988	9.60725	1.80123	9.60725	2.3989
23.347	0.24368	9.62694	0.0041	9.62694	0.60534	9.62694	1.19999	9.62694	1.80362	9.62694	2.39905
23.447	0.24383	9.64663	-0.00168	9.64663	0.60215	9.64663	1.20228	9.64663	1.80313	9.64663	2.40108
23.547	0.24392	9.66632	0.00182	9.66632	0.60184	9.66632	1.20054	9.66632	1.80558	9.66632	2.40177
23.647	0.24415	9.68601	0.00357	9.68601	0.60304	9.68601	1.2022	9.68601	1.79903	9.68601	2.40059
23.747	0.24404	9.7057	0.00772	9.7057	0.60379	9.7057	1.20072	9.7057	1.80236	9.7057	2.40128
23.847	0.24418	9.72538	0	9.72538	0.6006	9.72538	1.20213	9.72538	1.8	9.72538	2.4004
23.947	0.24424	9.74507	0.00454	9.74507	0.59992	9.74507	1.19884	9.74507	1.80723	9.74507	2.40086
24.047	0.24429	9.76476	-0.00136	9.76476	0.60431	9.76476	1.19911	9.76476	1.80328	9.76476	2.39707
24.147	0.24425	9.78445	0.00285	9.78445	0.60325	9.78445	1.20206	9.78445	1.80851	9.78445	2.40079
24.247	0.24451	9.80414	-0.00285	9.80414	0.60162	9.80414	1.20363	9.80414	1.80024	9.80414	2.39876
24.347	0.24483	9.82383	0.00266	9.82383	0.59919	9.82383	1.19982	9.82383	1.80179	9.82383	2.39849
24.447	0.24476	9.84352	0.00752	9.84352	0.60482	9.84352	1.20334	9.84352	1.79996	9.84352	2.39888
24.547	0.24522	9.86321	0.00357	9.86321	0.60414	9.86321	1.20238	9.86321	1.80344	9.86321	2.39849
24.647	0.245	9.8829	4.54E-04	9.8829	0.6002	9.8829	1.20017	9.8829	1.80649	9.8829	2.40039
24.747	0.24494	9.90259	0.00208	9.90259	0.60778	9.90259	1.20524	9.90259	1.80916	9.90259	2.39969
24.847	0.24532	9.92228	0.00169	9.92228	0.60528	9.92228	1.20133	9.92228	1.8077	9.92228	2.40239
24.947	0.24554	9.94196	0.00292	9.94196	0.60178	9.94196	1.20326	9.94196	1.80281	9.94196	2.39781
25.047	0.24565	9.96165	5.19E-04	9.96165	0.60785	9.96165	1.19909	9.96165	1.79979	9.96165	2.40469
25.147	0.24549	9.98134	-1.95E-04	9.98134	0.5959	9.98134	1.19735	9.98134	1.8019	9.98134	2.3989
25.247	0.24572	10.00103	0.00681	10.00103	0.60323	10.00103	1.20485	10.001	1.80351	10.00103	2.40051
25.347	0.24578	10.02072	0.0083	10.02072	0.60286	10.02072	1.20109	10.0207	1.79943	10.02072	2.39957
25.447	0.24565	10.04041	0.00674	10.04041	0.60205	10.04041	1.20358	10.0404	1.8046	10.04041	2.40081
25.547	0.24542	10.0601	0.00357	10.0601	0.60312	10.0601	1.2023	10.0601	1.80427	10.0601	2.3975
25.647	0.24539	10.07979	-0.00383	10.07979	0.60081	10.07979	1.19984	10.0798	1.80725	10.07979	2.39935
25.747	0.24537	10.09948	0.00182	10.09948	0.60525	10.09948	1.1998	10.0995	1.8063	10.09948	2.40605

25.847	0.24515	10.11917	0	10.11917	0.60432	10.11917	1.20229	10.1192	1.80084	10.11917	2.40056
25.947	0.24528	10.13885	-3.40E-05	10.13885	0.60313	10.13885	1.20422	10.1389	1.8017	10.13885	2.40265
26.047	0.24558	10.15854	0.0059	10.15854	0.60564	10.15854	1.19927	10.1585	1.80037	10.15854	2.40128
26.147	0.24548	10.17823	0.00301	10.17823	0.60652	10.17823	1.20233	10.1782	1.80985	10.17823	2.39919
26.247	0.24533	10.19792	0.00421	10.19792	0.60621	10.19792	1.20369	10.1979	1.79909	10.19792	2.39837
26.347	0.24516	10.21761	0.0056	10.21761	0.60772	10.21761	1.19973	10.2176	1.79907	10.21761	2.39707
26.447	0.24536	10.2373	0.00472	10.2373	0.60196	10.2373	1.20036	10.2373	1.80462	10.2373	2.4014
26.547	0.24508	10.25699	0.00463	10.25699	0.60654	10.25699	1.20471	10.257	1.80553	10.25699	2.39658
26.647	0.24536	10.27668	5.06E-04	10.27668	0.60078	10.27668	1.20503	10.2767	1.80621	10.27668	2.40491
26.747	0.24535	10.29637	0.00585	10.29637	0.60799	10.29637	1.19957	10.2964	1.80787	10.29637	2.40046
26.847	0.24536	10.31606	0.00511	10.31606	0.60474	10.31606	1.2	10.3161	1.80461	10.31606	2.4
26.947	0.2455	10.33575	0.00812	10.33575	0.60311	10.33575	1.20158	10.3358	1.80565	10.33575	2.39738
27.047	0.2453	10.35544	0.00348	10.35544	0.5998	10.35544	1.20708	10.3554	1.80751	10.35544	2.40258
27.147	0.24523	10.37512	0.0098	10.37512	0.60118	10.37512	1.202	10.3751	1.81024	10.37512	2.40014
27.247	0.24539	10.39481	0.00828	10.39481	0.60582	10.39481	1.20512	10.3948	1.8001	10.39481	2.40243
27.347	0.24556	10.4145	7.30E-05	10.4145	0.60244	10.4145	1.20391	10.4145	1.80527	10.4145	2.4032
27.447	0.24562	10.43419	0.00224	10.43419	0.60338	10.43419	1.20621	10.4342	1.80419	10.43419	2.40137
27.547	0.24528	10.45388	-3.84E-04	10.45388	0.60082	10.45388	1.19994	10.4539	1.80118	10.45388	2.40378
27.647	0.24556	10.47357	-0.00172	10.47357	0.6012	10.47357	1.20214	10.4736	1.80722	10.47357	2.4077
27.747	0.24566	10.49326	-9.71E-04	10.49326	0.6029	10.49326	1.20444	10.4933	1.79814	10.49326	2.41042
27.847	0.24545	10.51295	0.00185	10.51295	0.60547	10.51295	1.20018	10.513	1.8	10.51295	2.40295
27.947	0.24545	10.53264	-0.0035	10.53264	0.60516	10.53264	1.19959	10.5326	1.80372	10.53264	2.4059
28.047	0.24565	10.55233	0.00587	10.55233	0.60153	10.55233	1.19941	10.5523	1.80575	10.55233	2.4034
28.147	0.24588	10.57202	0.00609	10.57202	0.60141	10.57202	1.19923	10.572	1.80472	10.57202	2.40642
28.247	0.24608	10.5917	-0.00147	10.5917	0.59929	10.5917	1.19911	10.5917	1.80251	10.5917	2.40683
28.347	0.24581	10.61139	0.00395	10.61139	0.60204	10.61139	1.20058	10.6114	1.80279	10.61139	2.40887
28.447	0.24596	10.63108	-0.00568	10.63108	0.59961	10.63108	1.20128	10.6311	1.80701	10.63108	2.41159
28.547	0.24603	10.65077	-0.00468	10.65077	0.60174	10.65077	1.19847	10.6508	1.80641	10.65077	2.41387
28.647	0.24609	10.67046	0.00248	10.67046	0.60012	10.67046	1.20196	10.6705	1.80882	10.67046	2.41428
28.747	0.24597	10.69015	0.00524	10.69015	0.60632	10.69015	1.20204	10.6902	1.80823	10.69015	2.42154
28.847	0.24602	10.70984	0.00196	10.70984	0.6	10.70984	1.2	10.7098	1.80957	10.70984	2.41559
28.947	0.24615	10.72953	0.00115	10.72953	0.60275	10.72953	1.19418	10.7295	1.80961	10.72953	2.41382
29.047	0.24598	10.74922	0.00137	10.74922	0.60174	10.74922	1.19951	10.7492	1.81514	10.74922	2.41732
29.147	0.24623	10.76891	0.00458	10.76891	0.60236	10.76891	1.20232	10.7689	1.81467	10.76891	2.41973
29.247	0.24599	10.7886	0.00247	10.7886	0.60899	10.7886	1.19964	10.7886	1.80714	10.7886	2.41868
29.347	0.24589	10.80828	-0.00223	10.80828	0.60585	10.80828	1.20358	10.8083	1.81698	10.80828	2.41782
29.447	0.24577	10.82797	0.00409	10.82797	0.60084	10.82797	1.20231	10.828	1.81339	10.82797	2.41974
29.547	0.24619	10.84766	0.00146	10.84766	0.60509	10.84766	1.2017	10.8477	1.81755	10.84766	2.41682
29.647	0.24633	10.86735	0.00318	10.86735	0.59801	10.86735	1.19985	10.8674	1.82008	10.86735	2.41584
29.747	0.24646	10.88704	0.00159	10.88704	0.6017	10.88704	1.2008	10.887	1.81224	10.88704	2.42661
29.847	0.24653	10.90673	0	10.90673	0.60613	10.90673	1.20686	10.9067	1.82115	10.90673	2.4335
29.947	0.24655	10.92642	0.00679	10.92642	0.60306	10.92642	1.2046	10.9264	1.82049	10.92642	2.43579
30.047	0.24628	10.94611	0.00535	10.94611	0.59886	10.94611	1.20492	10.9461	1.81796	10.94611	2.44626
30.147	0.2464	10.9658	5.38E-04	10.9658	0.60449	10.9658	1.20535	10.9658	1.82543	10.9658	2.45031

30.247	0.24655	10.98549	0.00428	10.98549	0.6006	10.98549	1.20996	10.9855	1.82253	10.98549	2.45617
30.347	0.24634	11.00518	0.00401	11.00518	0.59997	11.00518	1.21018	11.0052	1.82206	11.00518	2.45804
30.447	0.24621	11.02486	0.00425	11.02486	0.60084	11.02486	1.20498	11.0249	1.82109	11.02486	2.46093
30.547	0.24606	11.04455	0.00489	11.04455	0.60434	11.04455	1.21527	11.0446	1.8205	11.04455	2.45783
30.647	0.24618	11.06424	0.00688	11.06424	0.60264	11.06424	1.21063	11.0642	1.81353	11.06424	2.44672
30.747	0.24614	11.08393	0.00641	11.08393	0.60952	11.08393	1.2075	11.0839	1.81213	11.08393	2.44258
30.847	0.24625	11.10362	0.00996	11.10362	0.60588	11.10362	1.20555	11.1036	1.81116	11.10362	2.43093
30.947	0.24622	11.12331	0.00444	11.12331	0.59999	11.12331	1.20562	11.1233	1.80676	11.12331	2.41583
31.047	0.24612	11.143	0.00754	11.143	0.60312	11.143	1.20811	11.143	1.8041	11.143	2.41272
31.147	0.24581	11.16269	0.00681	11.16269	0.60155	11.16269	1.20642	11.1627	1.8067	11.16269	2.40816
31.247	0.24571	11.18238	-0.00436	11.18238	0.60523	11.18238	1.20855	11.1824	1.80442	11.18238	2.40742
31.347	0.24573	11.20207	-0.00185	11.20207	0.60335	11.20207	1.19948	11.2021	1.80789	11.20207	2.40292
31.447	0.24586	11.22176	0.00255	11.22176	0.60447	11.22176	1.20517	11.2218	1.80105	11.22176	2.40672
31.547	0.24566	11.24144	0.00227	11.24144	0.60064	11.24144	1.20405	11.2414	1.80076	11.24144	2.4032
31.647	0.24573	11.26113	0.00563	11.26113	0.60314	11.26113	1.20122	11.2611	1.80411	11.26113	2.41033
31.747	0.24563	11.28082	0.00484	11.28082	0.60276	11.28082	1.20506	11.2808	1.80064	11.28082	2.40311
31.847	0.24536	11.30051	0.0019	11.30051	0.60638	11.30051	1.20419	11.3005	1.80036	11.30051	2.4
31.947	0.24531	11.3202	0.00104	11.3202	0.60431	11.3202	1.20271	11.3202	1.79952	11.3202	2.40352
32.047	0.24511	11.33989	0.0044	11.33989	0.60143	11.33989	1.20107	11.3399	1.80761	11.33989	2.40934
32.147	0.24506	11.35958	0.00251	11.35958	0.60849	11.35958	1.20212	11.3596	1.80565	11.35958	2.40819
32.247	0.24495	11.37927	0.00171	11.37927	0.60367	11.37927	1.19728	11.3793	1.80512	11.37927	2.40698
32.347	0.24477	11.39896	0.0015	11.39896	0.59747	11.39896	1.20602	11.399	1.80384	11.39896	2.4025
32.447	0.24469	11.41865	0.00376	11.41865	0.61016	11.41865	1.20263	11.4187	1.80031	11.41865	2.40517
32.547	0.24479	11.43834	0.00439	11.43834	0.60452	11.43834	1.2012	11.4383	1.80546	11.43834	2.40165
32.647	0.24476	11.45802	0.00664	11.45802	0.60596	11.45802	1.20354	11.458	1.80206	11.45802	2.40299
32.747	0.24448	11.47771	0.00241	11.47771	0.60257	11.47771	1.20438	11.4777	1.79903	11.47771	2.40444
32.847	0.24407	11.4974	0.00149	11.4974	0.60388	11.4974	1.2	11.4974	1.8	11.4974	2.40457
32.947	0.2441	11.51709	0.00122	11.51709	0.59524	11.51709	1.2024	11.5171	1.807	11.51709	2.40396
33.047	0.24402	11.53678	0.00256	11.53678	0.60443	11.53678	1.20372	11.5368	1.80087	11.53678	2.4093
33.147	0.24397	11.55647	1.49E-04	11.55647	0.60805	11.55647	1.20251	11.5565	1.80574	11.55647	2.40209
33.247	0.24397	11.57616	0.00441	11.57616	0.60611	11.57616	1.20352	11.5762	1.8073	11.57616	2.40518
33.347	0.24383	11.59585	6.38E-04	11.59585	0.60529	11.59585	1.2038	11.5959	1.80774	11.59585	2.40949
33.447	0.24404	11.61554	0.00451	11.61554	0.6034	11.61554	1.20068	11.6155	1.8018	11.61554	2.40652
33.547	0.24364	11.63523	0.00755	11.63523	0.60496	11.63523	1.20463	11.6352	1.80617	11.63523	2.41737
33.647	0.24368	11.65492	3.33E-04	11.65492	0.60539	11.65492	1.19955	11.6549	1.80916	11.65492	2.40762
33.747	0.24359	11.67461	0.00544	11.67461	0.59913	11.67461	1.20335	11.6746	1.80816	11.67461	2.4112
33.847	0.24343	11.69429	0.00588	11.69429	0.60188	11.69429	1.20497	11.6943	1.80635	11.69429	2.41859
33.947	0.24324	11.71398	0.00379	11.71398	0.59918	11.71398	1.20836	11.714	1.81297	11.71398	2.41702
34.047	0.24296	11.73367	0.00241	11.73367	0.60224	11.73367	1.20374	11.7337	1.81984	11.73367	2.42411
34.147	0.24282	11.75336	-5.86E-04	11.75336	0.60267	11.75336	1.20909	11.7534	1.83127	11.75336	2.43218
34.247	0.24301	11.77305	0.00524	11.77305	0.60016	11.77305	1.20426	11.7731	1.84221	11.77305	2.4423
34.347	0.24254	11.79274	0.00172	11.79274	0.60303	11.79274	1.20362	11.7927	1.85989	11.79274	2.45642
34.447	0.24265	11.81243	0.00157	11.81243	0.60616	11.81243	1.20313	11.8124	1.86626	11.81243	2.46709
34.547	0.24268	11.83212	0.00506	11.83212	0.60384	11.83212	1.21126	11.8321	1.87432	11.83212	2.49703

34.647	0.2427	11.85181	-2.08E-04	11.85181	0.60208	11.85181	1.21826	11.8518	1.88319	11.85181	2.50667
34.747	0.24255	11.8715	0.00464	11.8715	0.59506	11.8715	1.20899	11.8715	1.899	11.8715	2.53868
34.847	0.24262	11.89118	0.00294	11.89118	0.6	11.89118	1.21005	11.8912	1.90812	11.89118	2.56722
34.947	0.24287	11.91087	0.00435	11.91087	0.60177	11.91087	1.21034	11.9109	1.90736	11.91087	2.60026
35.047	0.2425	11.93056	0.0044	11.93056	0.59792	11.93056	1.20877	11.9306	1.9053	11.93056	2.61899
35.147	0.24249	11.95025	0.00322	11.95025	0.60201	11.95025	1.20807	11.9503	1.90055	11.95025	2.66178
35.247	0.24262	11.96994	0.00385	11.96994	0.60322	11.96994	1.20903	11.9699	1.89736	11.96994	2.69215
35.347	0.24248	11.98963	0.0017	11.98963	0.59923	11.98963	1.20766	11.9896	1.88217	11.98963	2.72269
35.447	0.24249	12.00932	0.00155	12.00932	0.60263	12.00932	1.20242	12.0093	1.87642	12.00932	2.72082
35.547	0.24236	12.02901	4.54E-05	12.02901	0.60597	12.02901	1.20348	12.029	1.86655	12.02901	2.687
35.647	0.24218	12.0487	0.00152	12.0487	0.60499	12.0487	1.2016	12.0487	1.85274	12.0487	2.64295
35.747	0.24218	12.06839	0.00216	12.06839	0.60451	12.06839	1.20261	12.0684	1.8358	12.06839	2.57404
35.847	0.24163	12.08808	0	12.08808	0.60066	12.08808	1.2	12.0881	1.82855	12.08808	2.51198
35.947	0.24173	12.10777	0.00566	12.10777	0.60687	12.10777	1.20318	12.1078	1.82661	12.10777	2.4629
36.047	0.24143	12.12745	-0.00192	12.12745	0.60483	12.12745	1.21148	12.1275	1.81443	12.12745	2.43526
36.147	0.24151	12.14714	0.00108	12.14714	0.60554	12.14714	1.20031	12.1471	1.80768	12.14714	2.42193
36.247	0.24119	12.16683	0.00187	12.16683	0.602	12.16683	1.20354	12.1668	1.80393	12.16683	2.41254
36.347	0.24067	12.18652	0.00247	12.18652	0.60959	12.18652	1.20735	12.1865	1.80555	12.18652	2.41279
36.447	0.2404	12.20621	0.00333	12.20621	0.60029	12.20621	1.2128	12.2062	1.80392	12.20621	2.41049
36.547	0.24026	12.2259	0.00295	12.2259	0.60431	12.2259	1.20432	12.2259	1.80192	12.2259	2.4057
36.647	0.24021	12.24559	0.00504	12.24559	0.60139	12.24559	1.21044	12.2456	1.80511	12.24559	2.40995
36.747	0.23991	12.26528	-9.73E-04	12.26528	0.60461	12.26528	1.21063	12.2653	1.80642	12.26528	2.40765
36.847	0.23981	12.28497	0.00617	12.28497	0.60425	12.28497	1.20907	12.285	1.80455	12.28497	2.40268
36.947	0.23987	12.30466	0.00807	12.30466	0.6044	12.30466	1.21111	12.3047	1.80698	12.30466	2.40505
37.047	0.23939	12.32435	0.00821	12.32435	0.60718	12.32435	1.21636	12.3244	1.80267	12.32435	2.40051
37.147	0.23911	12.34403	0.00226	12.34403	0.60883	12.34403	1.21397	12.344	1.80929	12.34403	2.4033
37.247	0.239	12.36372	0.00176	12.36372	0.60541	12.36372	1.21323	12.3637	1.80448	12.36372	2.40216
37.347	0.23845	12.38341	0.00417	12.38341	0.60706	12.38341	1.21688	12.3834	1.81198	12.38341	2.40695
37.447	0.23871	12.4031	-2.73E-05	12.4031	0.61477	12.4031	1.21681	12.4031	1.81017	12.4031	2.40198
37.547	0.23873	12.42279	0.00304	12.42279	0.61142	12.42279	1.22273	12.4228	1.81173	12.42279	2.40344
37.647	0.23849	12.44248	0.00759	12.44248	0.61707	12.44248	1.22726	12.4425	1.81042	12.44248	2.40411
37.747	0.23824	12.46217	0.00533	12.46217	0.61816	12.46217	1.2309	12.4622	1.81629	12.46217	2.40151
37.847	0.23839	12.48186	0.0093	12.48186	0.624	12.48186	1.24044	12.4819	1.81729	12.48186	2.40145
37.947	0.23854	12.50155	0.01107	12.50155	0.62953	12.50155	1.24646	12.5016	1.82379	12.50155	2.40321
38.047	0.23798	12.52124	0.00933	12.52124	0.63011	12.52124	1.26292	12.5212	1.82323	12.52124	2.40649
38.147	0.23803	12.54093	0.01499	12.54093	0.64409	12.54093	1.28154	12.5409	1.82311	12.54093	2.40474
38.247	0.23783	12.56061	0.01838	12.56061	0.65125	12.56061	1.30445	12.5606	1.83585	12.56061	2.40474
38.347	0.23763	12.5803	0.01865	12.5803	0.66554	12.5803	1.33464	12.5803	1.83036	12.5803	2.40335
38.447	0.23753	12.59999	0.02839	12.59999	0.69234	12.59999	1.37118	12.6	1.84104	12.59999	2.40209
38.547	0.23739	12.61968	0.02808	12.61968	0.71546	12.61968	1.39719	12.6197	1.85248	12.61968	2.40712
38.647	0.23739	12.63937	0.03912	12.63937	0.73782	12.63937	1.43678	12.6394	1.87435	12.63937	2.40955
38.747	0.23706	12.65906	0.05211	12.65906	0.76763	12.65906	1.49253	12.6591	1.90078	12.65906	2.40428
38.847	0.2374	12.67875	0.06373	12.67875	0.79706	12.67875	1.54213	12.6788	1.95296	12.67875	2.4115
38.947	0.23721	12.69844	0.08158	12.69844	0.83206	12.69844	1.61229	12.6984	2.0212	12.69844	2.40896

39.047	0.23693	12.71813	0.10637	12.71813	0.8886	12.71813	1.65725	12.7181	2.13212	12.71813	2.41448
39.147	0.23694	12.73782	0.12778	12.73782	0.93098	12.73782	1.72988	12.7378	2.24835	12.73782	2.41618
39.247	0.23675	12.75751	0.14868	12.75751	0.99922	12.75751	1.80469	12.7575	2.37414	12.75751	2.42861
39.347	0.23694	12.77719	0.18637	12.77719	1.06031	12.77719	1.90325	12.7772	2.48574	12.77719	2.43316
39.447	0.23682	12.79688	0.21881	12.79688	1.13437	12.79688	1.98234	12.7969	2.52386	12.79688	2.43783
39.547	0.23693	12.81657	0.25676	12.81657	1.22832	12.81657	2.059	12.8166	2.49537	12.81657	2.44868
39.647	0.23682	12.83626	0.31682	12.83626	1.28166	12.83626	2.08723	12.8363	2.35259	12.83626	2.45651
39.747	0.23655	12.85595	0.35808	12.85595	1.33062	12.85595	2.04456	12.856	2.18393	12.85595	2.46894
39.847	0.23672	12.87564	0.43338	12.87564	1.3312	12.87564	1.92681	12.8756	2.04109	12.87564	2.47894
39.947	0.23657	12.89533	0.48261	12.89533	1.25206	12.89533	1.72809	12.8953	1.95317	12.89533	2.49155
40.047	0.23662	12.91502	0.53062	12.91502	1.12292	12.91502	1.5475	12.915	1.9118	12.91502	2.50623
40.147	0.23671	12.93471	0.56487	12.93471	0.97883	12.93471	1.41374	12.9347	1.91393	12.93471	2.52944
40.247	0.23693	12.9544	0.56236	12.9544	0.85995	12.9544	1.32801	12.9544	1.90406	12.9544	2.52993
40.347	0.2372	12.97409	0.49416	12.97409	0.76667	12.97409	1.2873	12.9741	1.90269	12.97409	2.5477
40.447	0.23713	12.99377	0.3921	12.99377	0.72358	12.99377	1.26792	12.9938	1.90125	12.99377	2.56122
40.547	0.23724	13.01346	0.27792	13.01346	0.71021	13.01346	1.26826	13.0135	1.90351	13.01346	2.57056
40.647	0.23774	13.03315	0.19753	13.03315	0.70566	13.03315	1.26768	13.0332	1.8972	13.03315	2.57074
40.747	0.23787	13.05284	0.13218	13.05284	0.71495	13.05284	1.27024	13.0528	1.89876	13.05284	2.56978
40.847	0.2381	13.07253	0.12441	13.07253	0.72354	13.07253	1.27131	13.0725	1.8977	13.07253	2.54148
40.947	0.23796	13.09222	0.10952	13.09222	0.72632	13.09222	1.28632	13.0922	1.89183	13.09222	2.52294
41.047	0.23823	13.11191	0.10986	13.11191	0.73078	13.11191	1.28966	13.1119	1.8949	13.11191	2.48562
41.147	0.2385	13.1316	0.09904	13.1316	0.73156	13.1316	1.29532	13.1316	1.88478	13.1316	2.47187
41.247	0.23843	13.15129	0.09433	13.15129	0.7277	13.15129	1.31043	13.1513	1.88272	13.15129	2.45078
41.347	0.23869	13.17098	0.07521	13.17098	0.71201	13.17098	1.32224	13.171	1.88098	13.17098	2.43891
41.447	0.23865	13.19067	0.06803	13.19067	0.69151	13.19067	1.32542	13.1907	1.86636	13.19067	2.43679
41.547	0.23877	13.21035	0.05761	13.21035	0.68064	13.21035	1.32587	13.2104	1.86661	13.21035	2.4225
41.647	0.2387	13.23004	0.04427	13.23004	0.66421	13.23004	1.33536	13.23	1.86118	13.23004	2.42547
41.747	0.239	13.24973	0.04487	13.24973	0.64577	13.24973	1.33534	13.2497	1.85012	13.24973	2.41778
41.847	0.23907	13.26942	0.04307	13.26942	0.63991	13.26942	1.32489	13.2694	1.84431	13.26942	2.41639
41.947	0.23956	13.28911	0.03472	13.28911	0.63537	13.28911	1.32394	13.2891	1.83719	13.28911	2.41864
42.047	0.23951	13.3088	0.03052	13.3088	0.63576	13.3088	1.30942	13.3088	1.83351	13.3088	2.41932
42.147	0.23977	13.32849	0.03994	13.32849	0.62834	13.32849	1.30346	13.3285	1.83052	13.32849	2.41271
42.247	0.23999	13.34818	0.03898	13.34818	0.62573	13.34818	1.2847	13.3482	1.82252	13.34818	2.41569
42.347	0.23993	13.36787	0.03453	13.36787	0.62307	13.36787	1.27234	13.3679	1.82128	13.36787	2.41467
42.447	0.24018	13.38756	0.02728	13.38756	0.61927	13.38756	1.25694	13.3876	1.81322	13.38756	2.41043
42.547	0.24022	13.40725	0.02555	13.40725	0.61717	13.40725	1.24664	13.4073	1.8226	13.40725	2.41153
42.647	0.24036	13.42694	0.02355	13.42694	0.61606	13.42694	1.24146	13.4269	1.81835	13.42694	2.40826
42.747	0.2406	13.44662	0.0215	13.44662	0.6082	13.44662	1.23437	13.4466	1.81898	13.44662	2.41579
42.847	0.24108	13.46631	0.01127	13.46631	0.61267	13.46631	1.23079	13.4663	1.81349	13.46631	2.41706
42.947	0.24138	13.486	0.01109	13.486	0.61069	13.486	1.22561	13.486	1.81693	13.486	2.41519
43.047	0.24131	13.50569	0.00579	13.50569	0.60552	13.50569	1.22193	13.5057	1.82325	13.50569	2.42514
43.147	0.24166	13.52538	0.00892	13.52538	0.60216	13.52538	1.2186	13.5254	1.82269	13.52538	2.42854
43.247	0.2416	13.54507	0.00407	13.54507	0.60988	13.54507	1.21761	13.5451	1.82338	13.54507	2.43273
43.347	0.2418	13.56476	0.00137	13.56476	0.60095	13.56476	1.2132	13.5648	1.83882	13.56476	2.43716

43.447	0.24214	13.58445	0.00145	13.58445	0.60529	13.58445	1.21261	13.5845	1.83933	13.58445	2.45135
43.547	0.24223	13.60414	0.00263	13.60414	0.60494	13.60414	1.21141	13.6041	1.84633	13.60414	2.45972
43.647	0.24277	13.62383	0.00674	13.62383	0.60478	13.62383	1.21155	13.6238	1.85784	13.62383	2.47482
43.747	0.24288	13.64351	0.00526	13.64351	0.60655	13.64351	1.21029	13.6435	1.87009	13.64351	2.49095
43.847	0.24311	13.6632	0.00437	13.6632	0.6062	13.6632	1.20805	13.6632	1.87291	13.6632	2.49556
43.947	0.24356	13.68289	0.00238	13.68289	0.60497	13.68289	1.20654	13.6829	1.88566	13.68289	2.50926
44.047	0.24395	13.70258	-0.00117	13.70258	0.60449	13.70258	1.20275	13.7026	1.89554	13.70258	2.51721
44.147	0.24387	13.72227	0.00255	13.72227	0.60633	13.72227	1.20723	13.7223	1.89036	13.72227	2.52752
44.247	0.24402	13.74196	0.00211	13.74196	0.6046	13.74196	1.20329	13.742	1.89105	13.74196	2.54535
44.347	0.24418	13.76165	8.97E-04	13.76165	0.59818	13.76165	1.20668	13.7617	1.89081	13.76165	2.55359
44.447	0.24452	13.78134	0.00202	13.78134	0.6064	13.78134	1.2079	13.7813	1.88375	13.78134	2.56409
44.547	0.24485	13.80103	0.00333	13.80103	0.60342	13.80103	1.20246	13.801	1.8712	13.80103	2.56167
44.647	0.24512	13.82072	0.00102	13.82072	0.60156	13.82072	1.20554	13.8207	1.86714	13.82072	2.55852
44.747	0.24514	13.84041	-0.0026	13.84041	0.60446	13.84041	1.20351	13.8404	1.86702	13.84041	2.55859
44.847	0.24517	13.8601	0.00371	13.8601	0.60311	13.8601	1.20571	13.8601	1.85484	13.8601	2.53769
44.947	0.24572	13.87978	-7.44E-04	13.87978	0.60138	13.87978	1.20595	13.8798	1.84429	13.87978	2.52049
45.047	0.24558	13.89947	-0.00397	13.89947	0.59946	13.89947	1.20387	13.8995	1.84005	13.89947	2.49559
45.147	0.24566	13.91916	7.85E-04	13.91916	0.59823	13.91916	1.20086	13.9192	1.83655	13.91916	2.47008
45.247	0.24585	13.93885	0.00255	13.93885	0.61109	13.93885	1.20213	13.9389	1.82937	13.93885	2.45045
45.347	0.24627	13.95854	8.87E-04	13.95854	0.60103	13.95854	1.19928	13.9585	1.82476	13.95854	2.4367
45.447	0.24648	13.97823	-0.00383	13.97823	0.6065	13.97823	1.20081	13.9782	1.81601	13.97823	2.42889
45.547	0.24606	13.99792	0.00125	13.99792	0.60102	13.99792	1.20311	13.9979	1.81402	13.99792	2.40811
45.647	0.24609	14.01761	-5.48E-04	14.01761	0.59973	14.01761	1.20263	14.0176	1.80441	14.01761	2.40781
45.747	0.24624	14.0373	-2.05E-04	14.0373	0.59843	14.0373	1.20282	14.0373	1.80379	14.0373	2.40473
45.847	0.24615	14.05699	0.00189	14.05699	0.60165	14.05699	1.20224	14.057	1.80148	14.05699	2.40462
45.947	0.24634	14.07668	2.87E-04	14.07668	0.60305	14.07668	1.20238	14.0767	1.80699	14.07668	2.40632
46.047	0.24646	14.09636	4.36E-04	14.09636	0.6047	14.09636	1.20184	14.0964	1.80262	14.09636	2.4036
46.147	0.24677	14.11605	0.00104	14.11605	0.59821	14.11605	1.20327	14.1161	1.80319	14.11605	2.40203
46.247	0.2471	14.13574	4.10E-04	14.13574	0.6013	14.13574	1.20413	14.1357	1.80451	14.13574	2.40156
46.347	0.24711	14.15543	-8.03E-04	14.15543	0.60001	14.15543	1.2022	14.1554	1.80176	14.15543	2.40175
46.447	0.24724	14.17512	-0.00253	14.17512	0.60066	14.17512	1.20053	14.1751	1.79533	14.17512	2.40333
46.547	0.24715	14.19481	-0.00342	14.19481	0.59618	14.19481	1.20501	14.1948	1.7994	14.19481	2.40606
46.647	0.24697	14.2145	0.00191	14.2145	0.60114	14.2145	1.20101	14.2145	1.79897	14.2145	2.40995
46.747	0.24736	14.23419	-0.00176	14.23419	0.60117	14.23419	1.20322	14.2342	1.80029	14.23419	2.39935
46.847	0.24752	14.25388	7.85E-04	14.25388	0.6	14.25388	1.2	14.2539	1.80299	14.25388	2.40039
46.947	0.24753	14.27357	-8.17E-04	14.27357	0.60255	14.27357	1.20175	14.2736	1.79937	14.27357	2.40482
47.047	0.2474	14.29325	-0.00235	14.29325	0.6029	14.29325	1.20444	14.2933	1.79838	14.29325	2.40234
47.147	0.24757	14.31294	0.00298	14.31294	0.60714	14.31294	1.20077	14.3129	1.79994	14.31294	2.39799
47.247	0.2477	14.33263	-0.00277	14.33263	0.60605	14.33263	1.19969	14.3326	1.79764	14.33263	2.40291
47.347	0.24765	14.35232	-6.72E-04	14.35232	0.60197	14.35232	1.20304	14.3523	1.8044	14.35232	2.40055
47.447	0.24787	14.37201	0.00531	14.37201	0.59976	14.37201	1.20407	14.372	1.80059	14.37201	2.39668
47.547	0.24783	14.3917	-0.00212	14.3917	0.60581	14.3917	1.20283	14.3917	1.80522	14.3917	2.39705
47.647	0.24801	14.41139	0.00321	14.41139	0.61048	14.41139	1.20304	14.4114	1.80323	14.41139	2.40082
47.747	0.24818	14.43108	0.00336	14.43108	0.60208	14.43108	1.19772	14.4311	1.80249	14.43108	2.39743

47.847	0.24871	14.45077	0.00461	14.45077	0.607	14.45077	1.20046	14.4508	1.80068	14.45077	2.40047
47.947	0.24854	14.47046	0.00853	14.47046	0.60385	14.47046	1.20102	14.4705	1.80238	14.47046	2.40023
48.047	0.24835	14.49015	0.00206	14.49015	0.60934	14.49015	1.20578	14.4902	1.80363	14.49015	2.4
48.147	0.2485	14.50984	0.00526	14.50984	0.60969	14.50984	1.19927	14.5098	1.80008	14.50984	2.39867
48.247	0.24863	14.52952	0.013	14.52952	0.6113	14.52952	1.20268	14.5295	1.80227	14.52952	2.39765
48.347	0.2487	14.54921	0.01101	14.54921	0.60509	14.54921	1.20619	14.5492	1.80578	14.54921	2.40311
48.447	0.24871	14.5689	0.01375	14.5689	0.61326	14.5689	1.20557	14.5689	1.80803	14.5689	2.39773
48.547	0.24866	14.58859	0.0117	14.58859	0.60724	14.58859	1.21099	14.5886	1.80542	14.58859	2.40179
48.647	0.24838	14.60828	0.01989	14.60828	0.61923	14.60828	1.20846	14.6083	1.80436	14.60828	2.40065
48.747	0.24907	14.62797	0.01465	14.62797	0.61277	14.62797	1.20918	14.628	1.79637	14.62797	2.39436
48.847	0.24901	14.64766	0.01623	14.64766	0.61212	14.64766	1.21383	14.6477	1.8	14.64766	2.4
48.947	0.24915	14.66735	0.01755	14.66735	0.61129	14.66735	1.20464	14.6674	1.80314	14.66735	2.40064
49.047	0.24919	14.68704	0.02386	14.68704	0.61972	14.68704	1.2081	14.687	1.79929	14.68704	2.39685
49.147	0.24941	14.70673	0.02511	14.70673	0.6242	14.70673	1.20469	14.7067	1.80374	14.70673	2.40046
49.247	0.24908	14.72642	0.0215	14.72642	0.61905	14.72642	1.20552	14.7264	1.80313	14.72642	2.40225
49.347	0.24923	14.7461	0.01906	14.7461	0.62416	14.7461	1.20588	14.7461	1.79896	14.7461	2.40095
49.447	0.24955	14.76579	0.02038	14.76579	0.62233	14.76579	1.20051	14.7658	1.79535	14.76579	2.4008
49.547	0.24946	14.78548	0.03246	14.78548	0.62725	14.78548	1.20712	14.7855	1.80281	14.78548	2.39762
49.647	0.24953	14.80517	0.03066	14.80517	0.62949	14.80517	1.20557	14.8052	1.8017	14.80517	2.39989
49.747	0.24947	14.82486	0.03263	14.82486	0.62934	14.82486	1.20707	14.8249	1.80428	14.82486	2.3995
49.847	0.24957	14.84455	0.03524	14.84455	0.63082	14.84455	1.20779	14.8446	1.80473	14.84455	2.40559
49.947	0.24907	14.86424	0.03131	14.86424	0.63293	14.86424	1.20996	14.8642	1.80706	14.86424	2.40386
50.047	0.24927	14.88393	0.0349	14.88393	0.63786	14.88393	1.20909	14.8839	1.80683	14.88393	2.40771
50.147	0.24941	14.90362	0.03226	14.90362	0.63177	14.90362	1.21131	14.9036	1.80922	14.90362	2.40835
50.247	0.24963	14.92331	0.03448	14.92331	0.634	14.92331	1.21002	14.9233	1.81536	14.92331	2.41414
50.347	0.24969	14.943	0.0395	14.943	0.64093	14.943	1.2095	14.943	1.81601	14.943	2.41739
50.447	0.24985	14.96268	0.02992	14.96268	0.64491	14.96268	1.21957	14.9627	1.8154	14.96268	2.41651
50.547	0.25013	14.98237	0.03798	14.98237	0.63563	14.98237	1.21643	14.9824	1.82179	14.98237	2.42157
50.647	0.25027	15.00206	0.03865	15.00206	0.63035	15.00206	1.22722	15.0021	1.82475	15.00206	2.42379
50.747	0.25018	15.02175	0.02798	15.02175	0.62608	15.02175	1.22464	15.0218	1.83401	15.02175	2.42412
50.847	0.24981	15.04144	0.03721	15.04144	0.63188	15.04144	1.22665	15.0414	1.83797	15.04144	2.43179
50.947	0.25002	15.06113	0.02867	15.06113	0.62322	15.06113	1.22857	15.0611	1.8433	15.06113	2.43412
51.047	0.25005	15.08082	0.02797	15.08082	0.62126	15.08082	1.23507	15.0808	1.85094	15.08082	2.43907
51.147	0.25017	15.10051	0.02521	15.10051	0.61761	15.10051	1.23131	15.1005	1.8474	15.10051	2.43746
51.247	0.2503	15.1202	0.01427	15.1202	0.61503	15.1202	1.23761	15.1202	1.84485	15.1202	2.44513
51.347	0.25012	15.13989	0.01429	15.13989	0.60881	15.13989	1.23637	15.1399	1.83868	15.13989	2.44559
51.447	0.25017	15.15958	0.01334	15.15958	0.61436	15.15958	1.24175	15.1596	1.83852	15.15958	2.44138
51.547	0.25029	15.17927	0.00876	15.17927	0.60659	15.17927	1.24165	15.1793	1.83916	15.17927	2.43978
51.647	0.25038	15.19895	0.00949	15.19895	0.60619	15.19895	1.24118	15.199	1.8363	15.19895	2.44502
51.747	0.25066	15.21864	0.00932	15.21864	0.60348	15.21864	1.23876	15.2186	1.82295	15.21864	2.43706
51.847	0.25025	15.23833	0.00752	15.23833	0.60164	15.23833	1.24429	15.2383	1.82722	15.23833	2.438
51.947	0.25053	15.25802	0.0056	15.25802	0.60651	15.25802	1.23959	15.258	1.81874	15.25802	2.44209
52.047	0.25067	15.27771	0.00614	15.27771	0.59985	15.27771	1.2366	15.2777	1.81532	15.27771	2.44364
52.147	0.25136	15.2974	0.00324	15.2974	0.60177	15.2974	1.2428	15.2974	1.81559	15.2974	2.45422

52.247	0.25129	15.31709	3.47E-04	15.31709	0.60075	15.31709	1.23418	15.3171	1.82848	15.31709	2.46722
52.347	0.25105	15.33678	0.00439	15.33678	0.59942	15.33678	1.22918	15.3368	1.821	15.33678	2.47641
52.447	0.25116	15.35647	0.0024	15.35647	0.60835	15.35647	1.22649	15.3565	1.82789	15.35647	2.48808
52.547	0.25114	15.37616	0.00573	15.37616	0.59387	15.37616	1.21519	15.3762	1.8366	15.37616	2.5072
52.647	0.25116	15.39584	0.00867	15.39584	0.60249	15.39584	1.21819	15.3958	1.84362	15.39584	2.5199
52.747	0.25127	15.41553	0.00564	15.41553	0.60153	15.41553	1.21608	15.4155	1.8497	15.41553	2.53666
52.847	0.25151	15.43522	0.0045	15.43522	0.60833	15.43522	1.21463	15.4352	1.86434	15.43522	2.54991
52.947	0.25158	15.45491	0.00484	15.45491	0.60299	15.45491	1.21531	15.4549	1.87586	15.45491	2.56473
53.047	0.25158	15.4746	0.00525	15.4746	0.60316	15.4746	1.21051	15.4746	1.89094	15.4746	2.57313
53.147	0.25161	15.49429	0.00534	15.49429	0.59989	15.49429	1.21279	15.4943	1.90246	15.49429	2.58153
53.247	0.25148	15.51398	0.00783	15.51398	0.60031	15.51398	1.21254	15.514	1.91354	15.51398	2.59835
53.347	0.25152	15.53367	0.01362	15.53367	0.59766	15.53367	1.21006	15.5337	1.91287	15.53367	2.60663
53.447	0.25161	15.55336	0.00242	15.55336	0.60271	15.55336	1.21327	15.5534	1.93207	15.55336	2.62079
53.547	0.25159	15.57305	0.00186	15.57305	0.60137	15.57305	1.20945	15.5731	1.92747	15.57305	2.62573
53.647	0.25153	15.59274	0.00979	15.59274	0.60242	15.59274	1.20512	15.5927	1.93924	15.59274	2.63546
53.747	0.25171	15.61242	0.00463	15.61242	0.60966	15.61242	1.2105	15.6124	1.94057	15.61242	2.62889
53.847	0.2518	15.63211	0.00692	15.63211	0.6	15.63211	1.21195	15.6321	1.94515	15.63211	2.61874
53.947	0.25206	15.6518	0.00655	15.6518	0.60407	15.6518	1.20699	15.6518	1.93268	15.6518	2.61078
54.047	0.25222	15.67149	0.00579	15.67149	0.60839	15.67149	1.21547	15.6715	1.92614	15.67149	2.59487
54.147	0.25219	15.69118	7.57E-04	15.69118	0.60933	15.69118	1.21129	15.6912	1.91553	15.69118	2.56127
54.247	0.2521	15.71087	0.00272	15.71087	0.60326	15.71087	1.21672	15.7109	1.90537	15.71087	2.54046
54.347	0.25208	15.73056	0.00612	15.73056	0.59982	15.73056	1.21699	15.7306	1.90377	15.73056	2.50535
54.447	0.25193	15.75025	0.00141	15.75025	0.60269	15.75025	1.2174	15.7503	1.89541	15.75025	2.48005
54.547	0.252	15.76994	9.73E-04	15.76994	0.6082	15.76994	1.22004	15.7699	1.88444	15.76994	2.46069
54.647	0.25182	15.78963	0.00223	15.78963	0.59713	15.78963	1.22346	15.7896	1.87609	15.78963	2.44309
54.747	0.25198	15.80932	0.00166	15.80932	0.60777	15.80932	1.22326	15.8093	1.86336	15.80932	2.43367
54.847	0.25218	15.82901	0	15.82901	0.60476	15.82901	1.22657	15.829	1.84888	15.82901	2.42419
54.947	0.25232	15.84869	0.00274	15.84869	0.6047	15.84869	1.22797	15.8487	1.84597	15.84869	2.41865
55.047	0.25206	15.86838	0.00237	15.86838	0.60108	15.86838	1.23319	15.8684	1.84474	15.86838	2.41366
55.147	0.25206	15.88807	4.47E-04	15.88807	0.60784	15.88807	1.23805	15.8881	1.84295	15.88807	2.4126
55.247	0.25206	15.90776	-0.00122	15.90776	0.60215	15.90776	1.24229	15.9078	1.83573	15.90776	2.41446
55.347	0.25213	15.92745	-0.00496	15.92745	0.59996	15.92745	1.24401	15.9275	1.84918	15.92745	2.41243
55.447	0.25217	15.94714	-0.00144	15.94714	0.59877	15.94714	1.24443	15.9471	1.83258	15.94714	2.40623
55.547	0.25219	15.96683	-0.00194	15.96683	0.60553	15.96683	1.25177	15.9668	1.83054	15.96683	2.40542
55.647	0.25208	15.98652	-0.00315	15.98652	0.60453	15.98652	1.25719	15.9865	1.83319	15.98652	2.41297
55.747	0.25211	16.00621	0.00588	16.00621	0.60135	16.00621	1.25601	16.0062	1.82334	16.00621	2.41258
55.847	0.25205	16.0259	0.00201	16.0259	0.61105	16.0259	1.25334	16.0259	1.82017	16.0259	2.41862
55.947	0.25212	16.04559	-4.35E-04	16.04559	0.61005	16.04559	1.24942	16.0456	1.81776	16.04559	2.41248
56.047	0.25216	16.06527	0.00717	16.06527	0.60443	16.06527	1.25118	16.0653	1.81803	16.06527	2.41778
56.147	0.25231	16.08496	0.00233	16.08496	0.60462	16.08496	1.24128	16.085	1.82399	16.08496	2.42304
56.247	0.25192	16.10465	0.00468	16.10465	0.60269	16.10465	1.23917	16.1047	1.82727	16.10465	2.42853
56.347	0.25165	16.12434	0.006	16.12434	0.61214	16.12434	1.23649	16.1243	1.82548	16.12434	2.43196
56.447	0.25179	16.14403	0.00459	16.14403	0.60689	16.14403	1.23066	16.144	1.834	16.14403	2.43333
56.547	0.252	16.16372	0.00396	16.16372	0.60946	16.16372	1.22706	16.1637	1.84508	16.16372	2.44137

56.647	0.25187	16.18341	0.00236	16.18341	0.60872	16.18341	1.22598	16.1834	1.84736	16.18341	2.44935
56.747	0.25181	16.2031	9.53E-04	16.2031	0.60603	16.2031	1.22893	16.2031	1.85307	16.2031	2.44538
56.847	0.25214	16.22279	0	16.22279	0.60923	16.22279	1.22832	16.2228	1.86459	16.22279	2.45875
56.947	0.25184	16.24248	0.00586	16.24248	0.60799	16.24248	1.2321	16.2425	1.85805	16.24248	2.46631
57.047	0.25199	16.26217	0.0077	16.26217	0.61332	16.26217	1.23221	16.2622	1.86376	16.26217	2.46986
57.147	0.2521	16.28185	0.00462	16.28185	0.61433	16.28185	1.23336	16.2819	1.86941	16.28185	2.47651
57.247	0.25201	16.30154	0.01067	16.30154	0.62836	16.30154	1.24225	16.3015	1.87556	16.30154	2.48055
57.347	0.25218	16.32123	0.00947	16.32123	0.62355	16.32123	1.24443	16.3212	1.87052	16.32123	2.48883
57.447	0.25224	16.34092	0.00748	16.34092	0.63895	16.34092	1.2543	16.3409	1.87061	16.34092	2.49438
57.547	0.25213	16.36061	0.00679	16.36061	0.64747	16.36061	1.26593	16.3606	1.87332	16.36061	2.49387
57.647	0.25215	16.3803	0.01596	16.3803	0.65574	16.3803	1.2852	16.3803	1.85747	16.3803	2.49082
57.747	0.25192	16.39999	0.00613	16.39999	0.66251	16.39999	1.2929	16.4	1.85574	16.39999	2.48595
57.847	0.25207	16.41968	0.01478	16.41968	0.67297	16.41968	1.31609	16.4197	1.85671	16.41968	2.48084
57.947	0.25222	16.43937	0.01792	16.43937	0.69445	16.43937	1.33041	16.4394	1.84636	16.43937	2.46718
58.047	0.25223	16.45906	0.02139	16.45906	0.70341	16.45906	1.35407	16.4591	1.85138	16.45906	2.45922
58.147	0.25237	16.47874	0.02505	16.47874	0.71688	16.47874	1.38206	16.4787	1.86215	16.47874	2.45011
58.247	0.25224	16.49843	0.03785	16.49843	0.72941	16.49843	1.39395	16.4984	1.8828	16.49843	2.44627
58.347	0.25191	16.51812	0.04281	16.51812	0.74826	16.51812	1.41632	16.5181	1.9092	16.51812	2.44746
58.447	0.2519	16.53781	0.05055	16.53781	0.76349	16.53781	1.43559	16.5378	1.9554	16.53781	2.4475
58.547	0.25187	16.5575	0.05843	16.5575	0.79135	16.5575	1.45305	16.5575	2.00566	16.5575	2.44705
58.647	0.25177	16.57719	0.06273	16.57719	0.803	16.57719	1.48585	16.5772	2.04631	16.57719	2.44885
58.747	0.25172	16.59688	0.07865	16.59688	0.82254	16.59688	1.51302	16.5969	2.0897	16.59688	2.44841
58.847	0.25187	16.61657	0.08828	16.61657	0.83432	16.61657	1.55248	16.6166	2.08985	16.61657	2.45123
58.947	0.25184	16.63626	0.10465	16.63626	0.84054	16.63626	1.59267	16.6363	2.06644	16.63626	2.45364
59.047	0.25196	16.65595	0.11519	16.65595	0.8288	16.65595	1.61344	16.656	2.03447	16.65595	2.45246
59.147	0.25229	16.67564	0.12268	16.67564	0.80522	16.67564	1.64226	16.6756	1.9617	16.67564	2.45196
59.247	0.25237	16.69533	0.14768	16.69533	0.78672	16.69533	1.65038	16.6953	1.90792	16.69533	2.45521
59.347	0.25258	16.71502	0.15795	16.71502	0.75502	16.71502	1.63531	16.715	1.87589	16.71502	2.44258
59.447	0.25232	16.7347	0.16985	16.7347	0.72037	16.7347	1.58327	16.7347	1.85067	16.7347	2.44444
59.547	0.25246	16.75439	0.16755	16.75439	0.68948	16.75439	1.519	16.7544	1.84357	16.75439	2.43563
59.647	0.25239	16.77408	0.14476	16.77408	0.66778	16.77408	1.43556	16.7741	1.82879	16.77408	2.42786
59.747	0.2525	16.79377	0.1326	16.79377	0.65604	16.79377	1.36855	16.7938	1.82363	16.79377	2.43184
59.847	0.25217	16.81346	0.1113	16.81346	0.63378	16.81346	1.31269	16.8135	1.82084	16.81346	2.42733
59.947	0.25213	16.83315	0.08306	16.83315	0.62906	16.83315	1.27149	16.8332	1.81949	16.83315	2.42107
60.047	0.25201	16.85284	0.06313	16.85284	0.62289	16.85284	1.2525	16.8528	1.81814	16.85284	2.42553
60.147	0.25202	16.87253	0.04806	16.87253	0.61935	16.87253	1.23635	16.8725	1.82417	16.87253	2.42272
60.247	0.25201	16.89222	0.03422	16.89222	0.61105	16.89222	1.22743	16.8922	1.82932	16.89222	2.42882
60.347	0.25168	16.91191	0.02492	16.91191	0.6119	16.91191	1.21743	16.9119	1.83222	16.91191	2.4345
60.447	0.25189	16.93159	0.02035	16.93159	0.60955	16.93159	1.21496	16.9316	1.83381	16.93159	2.43678
60.547	0.25176	16.95128	0.01144	16.95128	0.60476	16.95128	1.21219	16.9513	1.84852	16.95128	2.44585
60.647	0.25198	16.97097	0.01271	16.97097	0.60485	16.97097	1.21478	16.971	1.84129	16.97097	2.45129
60.747	0.25211	16.99066	0.01398	16.99066	0.60769	16.99066	1.20911	16.9907	1.84563	16.99066	2.45824
60.847	0.25222	17.01035	0.00442	17.01035	0.60415	17.01035	1.20866	17.0104	1.84466	17.01035	2.47398
60.947	0.2519	17.03004	0.01232	17.03004	0.60438	17.03004	1.20573	17.03	1.84287	17.03004	2.46862

61.047	0.2517	17.04973	0.00866	17.04973	0.60316	17.04973	1.20683	17.0497	1.85021	17.04973	2.48315
61.147	0.25147	17.06942	0.00689	17.06942	0.6047	17.06942	1.2087	17.0694	1.84455	17.06942	2.48567
61.247	0.25149	17.08911	0.00953	17.08911	0.60167	17.08911	1.20128	17.0891	1.83945	17.08911	2.48596
61.347	0.25154	17.1088	0.01561	17.1088	0.60389	17.1088	1.20615	17.1088	1.83417	17.1088	2.49478
61.447	0.25146	17.12848	0.01591	17.12848	0.60205	17.12848	1.20105	17.1285	1.82675	17.12848	2.4887
61.547	0.25115	17.14817	0.0168	17.14817	0.60878	17.14817	1.19936	17.1482	1.82572	17.14817	2.46517
61.647	0.2512	17.16786	0.01775	17.16786	0.60494	17.16786	1.20547	17.1679	1.82424	17.16786	2.4677
61.747	0.25074	17.18755	0.02137	17.18755	0.60742	17.18755	1.20161	17.1876	1.81784	17.18755	2.44519
61.847	0.25065	17.20724	0.02284	17.20724	0.60783	17.20724	1.20007	17.2072	1.80824	17.20724	2.42687
61.947	0.25074	17.22693	0.0308	17.22693	0.60844	17.22693	1.20561	17.2269	1.80314	17.22693	2.42079
62.047	0.25079	17.24662	0.03993	17.24662	0.61436	17.24662	1.20656	17.2466	1.80148	17.24662	2.40877
62.147	0.25077	17.26631	0.05289	17.26631	0.6161	17.26631	1.20518	17.2663	1.80025	17.26631	2.4076
62.247	0.25056	17.286	0.05515	17.286	0.62616	17.286	1.20711	17.286	1.80034	17.286	2.40818
62.347	0.25013	17.30569	0.06623	17.30569	0.61613	17.30569	1.20748	17.3057	1.79425	17.30569	2.40586
62.447	0.24998	17.32538	0.07036	17.32538	0.63494	17.32538	1.20771	17.3254	1.79683	17.32538	2.40808
62.547	0.24999	17.34507	0.07509	17.34507	0.6343	17.34507	1.20189	17.3451	1.80267	17.34507	2.40291
62.647	0.25035	17.36476	0.08804	17.36476	0.64849	17.36476	1.20345	17.3648	1.7982	17.36476	2.40392
62.747	0.25004	17.38444	0.09724	17.38444	0.64246	17.38444	1.21189	17.3844	1.79785	17.38444	2.40463
62.847	0.24978	17.40413	0.10994	17.40413	0.65834	17.40413	1.2101	17.4041	1.8	17.40413	2.4
62.947	0.24922	17.42382	0.11739	17.42382	0.65664	17.42382	1.20676	17.4238	1.79503	17.42382	2.40124
63.047	0.24937	17.44351	0.12031	17.44351	0.66214	17.44351	1.21235	17.4435	1.80024	17.44351	2.39769
63.147	0.2489	17.4632	0.13918	17.4632	0.6789	17.4632	1.20396	17.4632	1.79439	17.4632	2.40117
63.247	0.24878	17.48289	0.13729	17.48289	0.67877	17.48289	1.2109	17.4829	1.79448	17.48289	2.40108
63.347	0.24853	17.50258	0.13463	17.50258	0.68495	17.50258	1.20998	17.5026	1.79044	17.50258	2.40274
63.447	0.2486	17.52227	0.13567	17.52227	0.68958	17.52227	1.21067	17.5223	1.79209	17.52227	2.39331
63.547	0.24825	17.54196	0.1437	17.54196	0.68688	17.54196	1.20791	17.542	1.79486	17.54196	2.39625
63.647	0.248	17.56165	0.1293	17.56165	0.69206	17.56165	1.21154	17.5617	1.7932	17.56165	2.39512
63.747	0.24758	17.58134	0.12061	17.58134	0.67854	17.58134	1.20965	17.5813	1.79735	17.58134	2.3969
63.847	0.24779	17.60102	0.11549	17.60102	0.67496	17.60102	1.22061	17.601	1.8	17.60102	2.40069
63.947	0.2476	17.62071	0.09857	17.62071	0.67753	17.62071	1.218	17.6207	1.79569	17.62071	2.39811
64.047	0.24759	17.6404	0.086	17.6404	0.6709	17.6404	1.22252	17.6404	1.79394	17.6404	2.39505
64.147	0.24756	17.66009	0.08263	17.66009	0.67491	17.66009	1.22073	17.6601	1.79731	17.66009	2.4
64.247	0.24755	17.67978	0.08439	17.67978	0.67572	17.67978	1.23495	17.6798	1.7965	17.67978	2.39712
64.347	0.24715	17.69947	0.0912	17.69947	0.68436	17.69947	1.24427	17.6995	1.79681	17.69947	2.39994
64.447	0.24716	17.71916	0.09251	17.71916	0.68699	17.71916	1.25317	17.7192	1.79712	17.71916	2.40179
64.547	0.24695	17.73885	0.10393	17.73885	0.7154	17.73885	1.26296	17.7389	1.80312	17.73885	2.40273
64.647	0.24711	17.75854	0.11042	17.75854	0.72767	17.75854	1.26923	17.7585	1.79656	17.75854	2.39785
64.747	0.24712	17.77823	0.11951	17.77823	0.74613	17.77823	1.27854	17.7782	1.80687	17.77823	2.39643
64.847	0.24675	17.79791	0.13125	17.79791	0.7676	17.79791	1.28549	17.7979	1.80787	17.79791	2.40252
64.947	0.24658	17.8176	0.14761	17.8176	0.77681	17.8176	1.30255	17.8176	1.80881	17.8176	2.40965
65.047	0.24649	17.83729	0.16844	17.83729	0.81518	17.83729	1.31089	17.8373	1.824	17.83729	2.40484
65.147	0.24631	17.85698	0.20043	17.85698	0.83184	17.85698	1.33116	17.857	1.83181	17.85698	2.41391
65.247	0.24638	17.87667	0.22101	17.87667	0.855	17.87667	1.3427	17.8767	1.84581	17.87667	2.42038
65.347	0.24614	17.89636	0.24858	17.89636	0.87617	17.89636	1.353	17.8964	1.86162	17.89636	2.42533

65.447	0.24648	17.91605	0.26371	17.91605	0.90716	17.91605	1.37291	17.9161	1.89393	17.91605	2.42652
65.547	0.24635	17.93574	0.27657	17.93574	0.94334	17.93574	1.38967	17.9357	1.91043	17.93574	2.43583
65.647	0.24643	17.95543	0.29312	17.95543	0.97339	17.95543	1.39883	17.9554	1.91306	17.95543	2.43787
65.747	0.24656	17.97512	0.31856	17.97512	1.01351	17.97512	1.41146	17.9751	1.89594	17.97512	2.43149
65.847	0.24665	17.99481	0.34283	17.99481	1.0333	17.99481	1.4057	17.9948	1.87045	17.99481	2.43686
65.947	0.24645	18.0145	0.3736	18.0145	1.08689	18.0145	1.38213	18.0145	1.84753	18.0145	2.4474
66.047	0.24608	18.03418	0.40397	18.03418	1.08779	18.03418	1.35304	18.0342	1.8366	18.03418	2.44799
66.147	0.24591	18.05387	0.44226	18.05387	1.06704	18.05387	1.3144	18.0539	1.82974	18.05387	2.45222
66.247	0.24631	18.07356	0.49449	18.07356	1.017	18.07356	1.28272	18.0736	1.8335	18.07356	2.45609
66.347	0.24631	18.09325	0.51955	18.09325	0.9261	18.09325	1.25492	18.0933	1.83163	18.09325	2.46384
66.447	0.2465	18.11294	0.56244	18.11294	0.8382	18.11294	1.2374	18.1129	1.83552	18.11294	2.4611
66.547	0.24632	18.13263	0.57616	18.13263	0.76494	18.13263	1.22534	18.1326	1.84127	18.13263	2.46376
66.647	0.24632	18.15232	0.54422	18.15232	0.71259	18.15232	1.22388	18.1523	1.84684	18.15232	2.46872
66.747	0.24668	18.17201	0.45242	18.17201	0.66837	18.17201	1.21694	18.172	1.84429	18.17201	2.47428
66.847	0.24647	18.1917	0.34649	18.1917	0.6433	18.1917	1.21284	18.1917	1.84224	18.1917	2.48052
66.947	0.24642	18.21139	0.22916	18.21139	0.63833	18.21139	1.21071	18.2114	1.86213	18.21139	2.48809
67.047	0.24658	18.23108	0.12681	18.23108	0.61959	18.23108	1.20899	18.2311	1.87189	18.23108	2.4967
67.147	0.24689	18.25076	0.0716	18.25076	0.62676	18.25076	1.21553	18.2508	1.8751	18.25076	2.50974
67.247	0.24713	18.27045	0.03948	18.27045	0.62617	18.27045	1.21293	18.2705	1.90317	18.27045	2.51774
67.347	0.24676	18.29014	0.02084	18.29014	0.61926	18.29014	1.21545	18.2901	1.91812	18.29014	2.52465
67.447	0.24658	18.30983	0.02497	18.30983	0.61347	18.30983	1.21579	18.3098	1.93539	18.30983	2.54393
67.547	0.24682	18.32952	0.01236	18.32952	0.61664	18.32952	1.22012	18.3295	1.95521	18.32952	2.55114
67.647	0.24687	18.34921	0.01571	18.34921	0.61166	18.34921	1.22103	18.3492	1.96392	18.34921	2.56757
67.747	0.24694	18.3689	0.01154	18.3689	0.61639	18.3689	1.22483	18.3689	1.96612	18.3689	2.58915
67.847	0.24699	18.38859	0.01495	18.38859	0.61067	18.38859	1.23009	18.3886	1.97364	18.38859	2.61072
67.947	0.24699	18.40828	0.01072	18.40828	0.61097	18.40828	1.23034	18.4083	1.98166	18.40828	2.62971
68.047	0.24737	18.42797	0.01097	18.42797	0.61202	18.42797	1.23058	18.428	1.98644	18.42797	2.65646
68.147	0.24738	18.44765	0.01874	18.44765	0.61444	18.44765	1.23011	18.4477	1.99265	18.44765	2.68557
68.247	0.24741	18.46734	0.01653	18.46734	0.61311	18.46734	1.23454	18.4673	1.99518	18.46734	2.71564
68.347	0.24759	18.48703	0.01898	18.48703	0.62054	18.48703	1.23138	18.487	1.99276	18.48703	2.75002
68.447	0.24792	18.50672	0.02428	18.50672	0.62541	18.50672	1.22993	18.5067	1.98973	18.50672	2.76647
68.547	0.24797	18.52641	0.02823	18.52641	0.62733	18.52641	1.22589	18.5264	1.98894	18.52641	2.77745
68.647	0.24796	18.5461	0.03036	18.5461	0.624	18.5461	1.22211	18.5461	1.97572	18.5461	2.76965
68.747	0.24812	18.56579	0.02736	18.56579	0.62354	18.56579	1.22133	18.5658	1.96894	18.56579	2.74203
68.847	0.24826	18.58548	0.03364	18.58548	0.63085	18.58548	1.21249	18.5855	1.96828	18.58548	2.70532
68.947	0.24804	18.60517	0.04518	18.60517	0.6418	18.60517	1.20742	18.6052	1.94982	18.60517	2.65972
69.047	0.24812	18.62486	0.03922	18.62486	0.64111	18.62486	1.20613	18.6249	1.92586	18.62486	2.61248
69.147	0.24782	18.64455	0.05744	18.64455	0.64248	18.64455	1.20814	18.6446	1.91252	18.64455	2.57427
69.247	0.24822	18.66424	0.05621	18.66424	0.64529	18.66424	1.20628	18.6642	1.894	18.66424	2.55279
69.347	0.24838	18.68392	0.06049	18.68392	0.64942	18.68392	1.20596	18.6839	1.88848	18.68392	2.53731
69.447	0.24855	18.70361	0.06918	18.70361	0.64478	18.70361	1.20162	18.7036	1.88645	18.70361	2.5428
69.547	0.24868	18.7233	0.06406	18.7233	0.64659	18.7233	1.20554	18.7233	1.87968	18.7233	2.52295
69.647	0.24885	18.74299	0.07748	18.74299	0.65297	18.74299	1.20921	18.743	1.87709	18.74299	2.53153
69.747	0.24897	18.76268	0.0754	18.76268	0.64646	18.76268	1.20631	18.7627	1.88975	18.76268	2.52914

69.847	0.2492	18.78237	0.08636	18.78237	0.6412	18.78237	1.21855	18.7824	1.89116	18.78237	2.525
69.947	0.24915	18.80206	0.08683	18.80206	0.6342	18.80206	1.21648	18.8021	1.89715	18.80206	2.52311
70.047	0.24908	18.82175	0.08477	18.82175	0.6332	18.82175	1.21328	18.8218	1.90838	18.82175	2.50783
70.147	0.24918	18.84144	0.07492	18.84144	0.62902	18.84144	1.22041	18.8414	1.91399	18.84144	2.51145
70.247	0.24909	18.86113	0.07753	18.86113	0.63047	18.86113	1.22264	18.8611	1.91217	18.86113	2.50702
70.347	0.24933	18.88082	0.06393	18.88082	0.62609	18.88082	1.22667	18.8808	1.92228	18.88082	2.50592
70.447	0.2496	18.90051	0.05142	18.90051	0.62316	18.90051	1.22925	18.9005	1.92189	18.90051	2.50415
70.547	0.24961	18.92019	0.04845	18.92019	0.63099	18.92019	1.23685	18.9202	1.91675	18.92019	2.51051
70.647	0.2501	18.93988	0.039	18.93988	0.62205	18.93988	1.24191	18.9399	1.91924	18.93988	2.51874
70.747	0.25018	18.95957	0.03998	18.95957	0.6183	18.95957	1.24636	18.9596	1.91885	18.95957	2.52807
70.847	0.25018	18.97926	0.0326	18.97926	0.61756	18.97926	1.24982	18.9793	1.92096	18.97926	2.53139
70.947	0.25061	18.99895	0.03029	18.99895	0.61376	18.99895	1.25556	18.999	1.91577	18.99895	2.54661
71.047	0.25045	19.01864	0.02526	19.01864	0.61121	19.01864	1.24849	19.0186	1.92339	19.01864	2.54583
71.147	0.25038	19.03833	0.02055	19.03833	0.61511	19.03833	1.24907	19.0383	1.92171	19.03833	2.54997
71.247	0.2504	19.05802	0.01455	19.05802	0.61038	19.05802	1.24691	19.058	1.91846	19.05802	2.54955
71.347	0.25061	19.07771	0.02073	19.07771	0.6169	19.07771	1.24192	19.0777	1.91939	19.07771	2.54714
71.447	0.25068	19.09739	0.02782	19.09739	0.61098	19.09739	1.23841	19.0974	1.92208	19.09739	2.55194
71.547	0.25062	19.11708	0.01352	19.11708	0.61882	19.11708	1.22959	19.1171	1.91477	19.11708	2.54892
71.647	0.25077	19.13677	0.0175	19.13677	0.62028	19.13677	1.22573	19.1368	1.90952	19.13677	2.55135
71.747	0.25077	19.15646	0.0146	19.15646	0.61917	19.15646	1.22435	19.1565	1.90433	19.15646	2.54718
71.847	0.25085	19.17615	0.02338	19.17615	0.62294	19.17615	1.22596	19.1762	1.89908	19.17615	2.54179
71.947	0.25092	19.19584	0.02277	19.19584	0.6363	19.19584	1.22091	19.1958	1.89796	19.19584	2.54952
72.047	0.25107	19.21553	0.02754	19.21553	0.64209	19.21553	1.22103	19.2155	1.88879	19.21553	2.54428
72.147	0.25093	19.23522	0.02965	19.23522	0.64331	19.23522	1.22219	19.2352	1.88762	19.23522	2.54413
72.247	0.25114	19.25491	0.04078	19.25491	0.65072	19.25491	1.22747	19.2549	1.88838	19.25491	2.5464
72.347	0.25148	19.2746	0.04723	19.2746	0.66433	19.2746	1.22738	19.2746	1.88277	19.2746	2.55079
72.447	0.25254	19.29429	0.05012	19.29429	0.67225	19.29429	1.23788	19.2943	1.88653	19.29429	2.54064
72.547	0.25214	19.31398	0.06657	19.31398	0.67184	19.31398	1.23088	19.314	1.88435	19.31398	2.54067
72.647	0.25235	19.33366	0.06913	19.33366	0.68414	19.33366	1.23699	19.3337	1.89561	19.33366	2.53482
72.747	0.25247	19.35335	0.07656	19.35335	0.69086	19.35335	1.2399	19.3534	1.88444	19.35335	2.5334
72.847	0.25247	19.37304	0.09346	19.37304	0.70247	19.37304	1.25329	19.373	1.90482	19.37304	2.51833
72.947	0.25257	19.39273	0.10706	19.39273	0.72348	19.39273	1.2529	19.3927	1.89104	19.39273	2.52408
73.047	0.25305	19.41242	0.11813	19.41242	0.72934	19.41242	1.25922	19.4124	1.89838	19.41242	2.52128
73.147	0.25344	19.43211	0.13614	19.43211	0.74684	19.43211	1.26838	19.4321	1.89922	19.43211	2.52509
73.247	0.2537	19.4518	0.1559	19.4518	0.75795	19.4518	1.27161	19.4518	1.90137	19.4518	2.52568
73.347	0.25361	19.47149	0.1754	19.47149	0.75218	19.47149	1.26352	19.4715	1.90708	19.47149	2.53925
73.447	0.25371	19.49118	0.20139	19.49118	0.74802	19.49118	1.26969	19.4912	1.90111	19.49118	2.53918
73.547	0.25352	19.51087	0.22335	19.51087	0.74913	19.51087	1.27116	19.5109	1.90644	19.51087	2.54523
73.647	0.25322	19.53056	0.2269	19.53056	0.73741	19.53056	1.27909	19.5306	1.90153	19.53056	2.54637
73.747	0.25352	19.55025	0.23655	19.55025	0.72743	19.55025	1.27395	19.5503	1.90068	19.55025	2.5406
73.847	0.25368	19.56993	0.23361	19.56993	0.73042	19.56993	1.27108	19.5699	1.90115	19.56993	2.5401
73.947	0.25363	19.58962	0.23134	19.58962	0.72302	19.58962	1.27395	19.5896	1.89394	19.58962	2.54175
74.047	0.25362	19.60931	0.19917	19.60931	0.72032	19.60931	1.27129	19.6093	1.89479	19.60931	2.52249
74.147	0.25365	19.629	0.17471	19.629	0.71711	19.629	1.26342	19.629	1.88671	19.629	2.52195

74.247	0.25372	19.64869	0.15104	19.64869	0.71385	19.64869	1.25364	19.6487	1.8805	19.64869	2.5085
74.347	0.25402	19.66838	0.13774	19.66838	0.71384	19.66838	1.24717	19.6684	1.88129	19.66838	2.49572
74.447	0.25411	19.68807	0.10628	19.68807	0.71495	19.68807	1.23599	19.6881	1.8847	19.68807	2.48767
74.547	0.25397	19.70776	0.11483	19.70776	0.71588	19.70776	1.23726	19.7078	1.87443	19.70776	2.4798
74.647	0.25371	19.72745	0.10458	19.72745	0.7143	19.72745	1.23006	19.7275	1.86547	19.72745	2.47405
74.747	0.25356	19.74714	0.09796	19.74714	0.71173	19.74714	1.22885	19.7471	1.8707	19.74714	2.46903
74.847	0.25352	19.76682	0.10495	19.76682	0.71503	19.76682	1.23275	19.7668	1.86468	19.76682	2.46819
74.947	0.25387	19.78651	0.09341	19.78651	0.70971	19.78651	1.23248	19.7865	1.85736	19.78651	2.46041
75.047	0.2538	19.8062	0.09504	19.8062	0.69669	19.8062	1.23443	19.8062	1.86016	19.8062	2.46243
75.147	0.25389	19.82589	0.10055	19.82589	0.68492	19.82589	1.23596	19.8259	1.85697	19.82589	2.46652
75.247	0.25395	19.84558	0.10231	19.84558	0.68386	19.84558	1.23409	19.8456	1.85671	19.84558	2.47686
75.347	0.25375	19.86527	0.0907	19.86527	0.66871	19.86527	1.23758	19.8653	1.85814	19.86527	2.4847
75.447	0.25355	19.88496	0.08818	19.88496	0.66001	19.88496	1.23736	19.885	1.85682	19.88496	2.49134
75.547	0.2533	19.90465	0.0804	19.90465	0.64993	19.90465	1.23972	19.9047	1.86724	19.90465	2.49203
75.647	0.25357	19.92434	0.07703	19.92434	0.64073	19.92434	1.23832	19.9243	1.87061	19.92434	2.51352
75.747	0.25338	19.94403	0.05978	19.94403	0.63835	19.94403	1.23784	19.944	1.86641	19.94403	2.51445
75.847	0.25359	19.96372	0.05349	19.96372	0.63184	19.96372	1.23002	19.9637	1.88603	19.96372	2.53291
75.947	0.25359	19.9834	0.04041	19.9834	0.62597	19.9834	1.22733	19.9834	1.89091	19.9834	2.54399
76.047	0.25381	20.00309	0.03102	20.00309	0.62409	20.00309	1.22371	20.0031	1.89166	20.00309	2.54161
76.147	0.25361	20.02278	0.03388	20.02278	0.61947	20.02278	1.22256	20.0228	1.90498	20.02278	2.54263
76.247	0.25343	20.04247	0.02974	20.04247	0.61353	20.04247	1.22116	20.0425	1.90779	20.04247	2.5497
76.347	0.25321	20.06216	0.02723	20.06216	0.62274	20.06216	1.21965	20.0622	1.91499	20.06216	2.55054
76.447	0.2533	20.08185	0.02503	20.08185	0.62093	20.08185	1.22052	20.0819	1.91712	20.08185	2.55119
76.547	0.25351	20.10154	0.02258	20.10154	0.61843	20.10154	1.22025	20.1015	1.90919	20.10154	2.56621
76.647	0.25323	20.12123	0.02551	20.12123	0.62382	20.12123	1.2109	20.1212	1.91207	20.12123	2.56038
76.747	0.25324	20.14092	0.01826	20.14092	0.62045	20.14092	1.21115	20.1409	1.91945	20.14092	2.56589
76.847	0.2533	20.16061	0.02326	20.16061	0.62502	20.16061	1.21047	20.1606	1.92308	20.16061	2.57254
76.947	0.25308	20.1803	0.02348	20.1803	0.62441	20.1803	1.2147	20.1803	1.91078	20.1803	2.57491
77.047	0.25271	20.19999	0.02641	20.19999	0.62505	20.19999	1.21449	20.2	1.91936	20.19999	2.57528
77.147	0.25292	20.21968	0.02585	20.21968	0.62506	20.21968	1.21361	20.2197	1.90364	20.21968	2.57152
77.247	0.25294	20.23936	0.0306	20.23936	0.62789	20.23936	1.21216	20.2394	1.89384	20.23936	2.56129
77.347	0.25326	20.25905	0.03186	20.25905	0.63454	20.25905	1.21169	20.2591	1.89505	20.25905	2.54996
77.447	0.25306	20.27874	0.02539	20.27874	0.63543	20.27874	1.21076	20.2787	1.88613	20.27874	2.52536
77.547	0.25278	20.29843	0.02658	20.29843	0.62999	20.29843	1.21607	20.2984	1.8884	20.29843	2.51009
77.647	0.25267	20.31812	0.02355	20.31812	0.63057	20.31812	1.21338	20.3181	1.8693	20.31812	2.47816
77.747	0.25296	20.33781	0.02798	20.33781	0.63459	20.33781	1.22113	20.3378	1.86213	20.33781	2.4641
77.847	0.25279	20.3575	0.01704	20.3575	0.62872	20.3575	1.21229	20.3575	1.85228	20.3575	2.45053
77.947	0.2527	20.37719	0.01706	20.37719	0.63093	20.37719	1.21622	20.3772	1.83819	20.37719	2.43789
78.047	0.2524	20.39688	0.01223	20.39688	0.62932	20.39688	1.21343	20.3969	1.83391	20.39688	2.42476
78.147	0.25243	20.41656	0.02062	20.41656	0.62978	20.41656	1.20863	20.4166	1.82663	20.41656	2.42205
78.247	0.25235	20.43625	0.01695	20.43625	0.63086	20.43625	1.20864	20.4363	1.82004	20.43625	2.42226
78.347	0.25194	20.45594	0.01632	20.45594	0.62738	20.45594	1.20554	20.4559	1.82363	20.45594	2.4184
78.447	0.25201	20.47563	0.01739	20.47563	0.62533	20.47563	1.20296	20.4756	1.81591	20.47563	2.42073
78.547	0.25194	20.49532	0.02377	20.49532	0.62485	20.49532	1.20539	20.4953	1.8112	20.49532	2.42099

78.647	0.25195	20.51501	0.02587	20.51501	0.62882	20.51501	1.20916	20.515	1.80873	20.51501	2.41265
78.747	0.25182	20.5347	0.03167	20.5347	0.62101	20.5347	1.2103	20.5347	1.80614	20.5347	2.41813
78.847	0.25196	20.55439	0.04168	20.55439	0.62917	20.55439	1.20854	20.5544	1.80898	20.55439	2.41973
78.947	0.25197	20.57408	0.03607	20.57408	0.62606	20.57408	1.21155	20.5741	1.81278	20.57408	2.42025
79.047	0.25225	20.59377	0.04038	20.59377	0.6371	20.59377	1.21382	20.5938	1.81545	20.59377	2.42047
79.147	0.25246	20.61346	0.04242	20.61346	0.63688	20.61346	1.21419	20.6135	1.81137	20.61346	2.42517
79.247	0.25268	20.63315	0.05036	20.63315	0.6426	20.63315	1.21306	20.6332	1.81098	20.63315	2.42206
79.347	0.25234	20.65283	0.04981	20.65283	0.64268	20.65283	1.21653	20.6528	1.81227	20.65283	2.42422
79.447	0.25296	20.67252	0.05814	20.67252	0.65785	20.67252	1.21519	20.6725	1.81413	20.67252	2.42565
79.547	0.25311	20.69221	0.0574	20.69221	0.66351	20.69221	1.21102	20.6922	1.81492	20.69221	2.43151
79.647	0.25271	20.7119	0.06035	20.7119	0.66197	20.7119	1.20958	20.7119	1.81578	20.7119	2.43245
79.747	0.25272	20.73159	0.04994	20.73159	0.66412	20.73159	1.21165	20.7316	1.81626	20.73159	2.43728
79.847	0.25265	20.75128	0.05438	20.75128	0.66809	20.75128	1.21047	20.7513	1.81237	20.75128	2.43731
79.947	0.25253	20.77097	0.05649	20.77097	0.67844	20.77097	1.21487	20.771	1.81155	20.77097	2.43803
80.047	0.25245	20.79066	0.06191	20.79066	0.6806	20.79066	1.21142	20.7907	1.80617	20.79066	2.43857
80.147	0.25231	20.81035	0.05623	20.81035	0.68751	20.81035	1.21226	20.8104	1.81323	20.81035	2.44147
80.247	0.25237	20.83004	0.04803	20.83004	0.69117	20.83004	1.21175	20.83	1.80866	20.83004	2.45231
80.347	0.25288	20.84973	0.05909	20.84973	0.69389	20.84973	1.21377	20.8497	1.81153	20.84973	2.4563
80.447	0.25291	20.86942	0.07288	20.86942	0.70199	20.86942	1.21151	20.8694	1.80996	20.86942	2.45829
80.547	0.2529	20.8891	0.0698	20.8891	0.70803	20.8891	1.21509	20.8891	1.81739	20.8891	2.45889
80.647	0.25284	20.90879	0.08819	20.90879	0.71294	20.90879	1.21443	20.9088	1.82451	20.90879	2.46682
80.747	0.253	20.92848	0.10476	20.92848	0.71735	20.92848	1.21309	20.9285	1.83175	20.92848	2.47299
80.847	0.25318	20.94817	0.11082	20.94817	0.72914	20.94817	1.2193	20.9482	1.83556	20.94817	2.48359
80.947	0.25335	20.96786	0.13025	20.96786	0.743	20.96786	1.22293	20.9679	1.851	20.96786	2.48365
81.047	0.25347	20.98755	0.14222	20.98755	0.73527	20.98755	1.22212	20.9876	1.85519	20.98755	2.49886
81.147	0.25367	21.00724	0.15769	21.00724	0.74087	21.00724	1.22807	21.0072	1.86076	21.00724	2.49832
81.247	0.25393	21.02693	0.17063	21.02693	0.73671	21.02693	1.23056	21.0269	1.86508	21.02693	2.50856
81.347	0.25399	21.04662	0.17307	21.04662	0.73136	21.04662	1.23424	21.0466	1.87464	21.04662	2.51608
81.447	0.25389	21.06631	0.19425	21.06631	0.71725	21.06631	1.2342	21.0663	1.8824	21.06631	2.5233
81.547	0.25384	21.08599	0.2011	21.08599	0.70589	21.08599	1.24165	21.086	1.88403	21.08599	2.53136
81.647	0.25395	21.10568	0.22383	21.10568	0.69071	21.10568	1.24301	21.1057	1.89035	21.10568	2.53882
81.747	0.25487	21.12537	0.22731	21.12537	0.66715	21.12537	1.24576	21.1254	1.89085	21.12537	2.54391
81.847	0.25466	21.14506	0.2278	21.14506	0.66675	21.14506	1.24438	21.1451	1.89548	21.14506	2.55803
81.947	0.25457	21.16475	0.2266	21.16475	0.6537	21.16475	1.25606	21.1648	1.89263	21.16475	2.56271
82.047	0.25483	21.18444	0.2151	21.18444	0.64072	21.18444	1.2472	21.1844	1.90176	21.18444	2.57575
82.147	0.25507	21.20413	0.18213	21.20413	0.6445	21.20413	1.24737	21.2041	1.88866	21.20413	2.58607
82.247	0.25533	21.22382	0.14994	21.22382	0.64616	21.22382	1.24279	21.2238	1.89611	21.22382	2.60747
82.347	0.25566	21.24351	0.11386	21.24351	0.63631	21.24351	1.24089	21.2435	1.89769	21.24351	2.62215
82.447	0.25554	21.2632	0.08706	21.2632	0.62733	21.2632	1.23838	21.2632	1.90702	21.2632	2.65537
82.547	0.25587	21.28289	0.05772	21.28289	0.62711	21.28289	1.23597	21.2829	1.90035	21.28289	2.66842
82.647	0.25597	21.30257	0.04103	21.30257	0.62132	21.30257	1.23325	21.3026	1.91436	21.30257	2.70231
82.747	0.25621	21.32226	0.02745	21.32226	0.61679	21.32226	1.23414	21.3223	1.91232	21.32226	2.70941
82.847	0.25626	21.34195	0.01284	21.34195	0.60912	21.34195	1.2324	21.342	1.92433	21.34195	2.74657
82.947	0.25669	21.36164	0.01022	21.36164	0.61316	21.36164	1.23288	21.3616	1.92786	21.36164	2.77235

83.047	0.25671	21.38133	7.23E-04	21.38133	0.61107	21.38133	1.2281	21.3813	1.96282	21.38133	2.78964
83.147	0.25712	21.40102	0.00355	21.40102	0.6121	21.40102	1.23044	21.401	1.98459	21.40102	2.81814
83.247	0.25726	21.42071	-0.00173	21.42071	0.60932	21.42071	1.23134	21.4207	2.00224	21.42071	2.82531
83.347	0.25735	21.4404	-0.00772	21.4404	0.60284	21.4404	1.22795	21.4404	2.04026	21.4404	2.852
83.447	0.25756	21.46009	-0.00132	21.46009	0.60744	21.46009	1.23715	21.4601	2.06103	21.46009	2.8739
83.547	0.25794	21.47978	-0.00349	21.47978	0.5999	21.47978	1.24068	21.4798	2.0883	21.47978	2.88252
83.647	0.25801	21.49947	-0.0065	21.49947	0.6025	21.49947	1.24086	21.4995	2.1112	21.49947	2.89266
83.747	0.25808	21.51916	-0.00218	21.51916	0.60297	21.51916	1.24407	21.5192	2.11897	21.51916	2.90777
83.847	0.25844	21.53884	0	21.53884	0.6	21.53884	1.24843	21.5388	2.14893	21.53884	2.91446
83.947	0.25853	21.55853	-1.21E-04	21.55853	0.59864	21.55853	1.25604	21.5585	2.16322	21.55853	2.90212
84.047	0.25862	21.57822	5.35E-04	21.57822	0.60391	21.57822	1.2494	21.5782	2.15894	21.57822	2.89584
84.147	0.25877	21.59791	-0.00134	21.59791	0.60718	21.59791	1.24239	21.5979	2.16566	21.59791	2.88986
84.247	0.25896	21.6176	2.93E-04	21.6176	0.60207	21.6176	1.24763	21.6176	2.18394	21.6176	2.86607
84.347	0.25909	21.63729	0.00406	21.63729	0.60353	21.63729	1.23969	21.6373	2.18973	21.63729	2.84221
84.447	0.25948	21.65698	0.00174	21.65698	0.60467	21.65698	1.23233	21.657	2.19108	21.65698	2.79696
84.547	0.25975	21.67667	0.00557	21.67667	0.60137	21.67667	1.22821	21.6767	2.18855	21.67667	2.75238
84.647	0.25961	21.69636	0.01252	21.69636	0.60289	21.69636	1.21625	21.6964	2.17609	21.69636	2.70889
84.747	0.26021	21.71605	0.01181	21.71605	0.60979	21.71605	1.20687	21.7161	2.16469	21.71605	2.65437
84.847	0.26062	21.73573	0.00851	21.73573	0.6053	21.73573	1.20658	21.7357	2.14754	21.73573	2.61081
84.947	0.26041	21.75542	0.01274	21.75542	0.60263	21.75542	1.20314	21.7554	2.13053	21.75542	2.56211
85.047	0.26041	21.77511	0.01055	21.77511	0.60828	21.77511	1.20156	21.7751	2.10633	21.77511	2.53232
85.147	0.26093	21.7948	0.01186	21.7948	0.60323	21.7948	1.2024	21.7948	2.07631	21.7948	2.50944
85.247	0.26067	21.81449	0.00461	21.81449	0.6082	21.81449	1.19834	21.8145	2.04474	21.81449	2.48292
85.347	0.26137	21.83418	0.01071	21.83418	0.61291	21.83418	1.19604	21.8342	2.02266	21.83418	2.46555
85.447	0.2615	21.85387	0.00424	21.85387	0.59991	21.85387	1.20618	21.8539	1.98328	21.85387	2.45218
85.547	0.26142	21.87356	0.00425	21.87356	0.60212	21.87356	1.2	21.8736	1.94858	21.87356	2.439
85.647	0.26148	21.89325	-0.00313	21.89325	0.60271	21.89325	1.2012	21.8933	1.926	21.89325	2.42793
85.747	0.26152	21.91294	-0.0061	21.91294	0.60185	21.91294	1.19941	21.9129	1.89568	21.91294	2.42747
85.847	0.26205	21.93263	0.00442	21.93263	0.60431	21.93263	1.2	21.9326	1.87317	21.93263	2.42235
85.947	0.26204	21.95231	-0.00335	21.95231	0.60634	21.95231	1.20379	21.9523	1.86103	21.95231	2.41643
86.047	0.26225	21.972	-8.01E-04	21.972	0.61162	21.972	1.20204	21.972	1.84909	21.972	2.41609
86.147	0.26237	21.99169	2.54E-04	21.99169	0.61033	21.99169	1.20774	21.9917	1.83289	21.99169	2.41091
86.247	0.26275	22.01138	0.00766	22.01138	0.60591	22.01138	1.20951	22.0114	1.82551	22.01138	2.40669
86.347	0.26295	22.03107	0.00716	22.03107	0.61432	22.03107	1.20808	22.0311	1.81831	22.03107	2.41054
86.447	0.26302	22.05076	0.01418	22.05076	0.61315	22.05076	1.20897	22.0508	1.81143	22.05076	2.40238
86.547	0.26332	22.07045	0.0201	22.07045	0.61781	22.07045	1.20625	22.0705	1.81173	22.07045	2.39944
86.647	0.26323	22.09014	0.01927	22.09014	0.62272	22.09014	1.21473	22.0901	1.80735	22.09014	2.40625
86.747	0.26345	22.10983	0.02532	22.10983	0.6141	22.10983	1.21191	22.1098	1.80134	22.10983	2.40058
86.847	0.26383	22.12952	0.03046	22.12952	0.6237	22.12952	1.2097	22.1295	1.80289	22.12952	2.4
86.947	0.26403	22.14921	0.03717	22.14921	0.62311	22.14921	1.21106	22.1492	1.79889	22.14921	2.40016
87.047	0.26427	22.1689	0.0379	22.1689	0.62045	22.1689	1.21232	22.1689	1.80032	22.1689	2.40154
87.147	0.26458	22.18858	0.04123	22.18858	0.62292	22.18858	1.21554	22.1886	1.79876	22.18858	2.39855
87.247	0.26473	22.20827	0.048	22.20827	0.62845	22.20827	1.2123	22.2083	1.79494	22.20827	2.39908
87.347	0.26476	22.22796	0.04388	22.22796	0.62761	22.22796	1.21351	22.228	1.79756	22.22796	2.39961

87.447	0.26478	22.24765	0.03871	22.24765	0.62545	22.24765	1.21311	22.2477	1.79481	22.24765	2.40474
87.547	0.26501	22.26734	0.0401	22.26734	0.63249	22.26734	1.21333	22.2673	1.80106	22.26734	2.40969
87.647	0.26528	22.28703	0.0374	22.28703	0.62845	22.28703	1.21671	22.287	1.80099	22.28703	2.41022
87.747	0.26525	22.30672	0.04903	22.30672	0.63755	22.30672	1.21616	22.3067	1.8003	22.30672	2.41002
87.847	0.26559	22.32641	0.05081	22.32641	0.64134	22.32641	1.21153	22.3264	1.80255	22.32641	2.4077
87.947	0.26608	22.3461	0.05434	22.3461	0.64888	22.3461	1.21346	22.3461	1.80518	22.3461	2.40792
88.047	0.26621	22.36579	0.05651	22.36579	0.65048	22.36579	1.21513	22.3658	1.80349	22.36579	2.4079
88.147	0.26655	22.38548	0.06147	22.38548	0.65728	22.38548	1.21768	22.3855	1.81037	22.38548	2.41261
88.247	0.26677	22.40516	0.07318	22.40516	0.66207	22.40516	1.21419	22.4052	1.80637	22.40516	2.41974
88.347	0.26692	22.42485	0.07729	22.42485	0.66686	22.42485	1.22123	22.4249	1.805	22.42485	2.4133
88.447	0.26713	22.44454	0.0925	22.44454	0.67654	22.44454	1.22264	22.4445	1.80482	22.44454	2.41395
88.547	0.26768	22.46423	0.10946	22.46423	0.68771	22.46423	1.22746	22.4642	1.80239	22.46423	2.41854
88.647	0.2679	22.48392	0.1021	22.48392	0.68763	22.48392	1.22795	22.4839	1.8102	22.48392	2.41222
88.747	0.26823	22.50361	0.11821	22.50361	0.69955	22.50361	1.22518	22.5036	1.80289	22.50361	2.41735
88.847	0.26847	22.5233	0.11811	22.5233	0.70184	22.5233	1.22577	22.5233	1.81239	22.5233	2.41388
88.947	0.26864	22.54299	0.11524	22.54299	0.69544	22.54299	1.22605	22.543	1.80953	22.54299	2.41065
89.047	0.26898	22.56268	0.11722	22.56268	0.70299	22.56268	1.22974	22.5627	1.80466	22.56268	2.4116
89.147	0.26939	22.58237	0.10507	22.58237	0.69553	22.58237	1.23033	22.5824	1.80118	22.58237	2.41595
89.247	0.26997	22.60205	0.11231	22.60205	0.69532	22.60205	1.22839	22.6021	1.80131	22.60205	2.41423
89.347	0.27041	22.62174	0.09764	22.62174	0.68461	22.62174	1.22784	22.6217	1.80238	22.62174	2.40924
89.447	0.27075	22.64143	0.08866	22.64143	0.68815	22.64143	1.2291	22.6414	1.80408	22.64143	2.40922
89.547	0.27072	22.66112	0.09039	22.66112	0.67662	22.66112	1.2267	22.6611	1.80134	22.66112	2.41648
89.647	0.271	22.68081	0.07999	22.68081	0.68574	22.68081	1.22992	22.6808	1.80628	22.68081	2.4064
89.747	0.2715	22.7005	0.06888	22.7005	0.6779	22.7005	1.23072	22.7005	1.80273	22.7005	2.40462
89.847	0.27185	22.72019	0.06814	22.72019	0.67738	22.72019	1.22894	22.7202	1.80218	22.72019	2.4
89.947	0.27218	22.73988	0.05957	22.73988	0.67744	22.73988	1.23805	22.7399	1.80307	22.73988	2.40379
90.047	0.2722	22.75957	0.05353	22.75957	0.67248	22.75957	1.23818	22.7596	1.80052	22.75957	2.39794
90.147	0.27251	22.77926	0.05403	22.77926	0.67216	22.77926	1.23336	22.7793	1.80197	22.77926	2.39694
90.247	0.27268	22.79895	0.05292	22.79895	0.66464	22.79895	1.23679	22.799	1.80324	22.79895	2.39885
90.347	0.27264	22.81864	0.05219	22.81864	0.66664	22.81864	1.2425	22.8186	1.79488	22.81864	2.39421
90.447	0.27298	22.83833	0.04959	22.83833	0.66744	22.83833	1.23736	22.8383	1.79558	22.83833	2.39388
90.547	0.2733	22.85801	0.05307	22.85801	0.66605	22.85801	1.24472	22.858	1.79516	22.85801	2.39743
90.647	0.27344	22.8777	0.05624	22.8777	0.67762	22.8777	1.24764	22.8777	1.80504	22.8777	2.39715
90.747	0.27373	22.89739	0.05733	22.89739	0.66685	22.89739	1.25014	22.8974	1.80381	22.89739	2.397
90.847	0.27367	22.91708	0.06153	22.91708	0.6731	22.91708	1.24718	22.9171	1.80195	22.91708	2.40455
90.947	0.27388	22.93677	0.06873	22.93677	0.6818	22.93677	1.25103	22.9368	1.80672	22.93677	2.39858
91.047	0.27389	22.95646	0.06477	22.95646	0.67278	22.95646	1.25643	22.9565	1.80592	22.95646	2.40122
91.147	0.27396	22.97615	0.06834	22.97615	0.67917	22.97615	1.26003	22.9762	1.80732	22.97615	2.41252
91.247	0.27396	22.99584	0.07644	22.99584	0.6771	22.99584	1.26424	22.9958	1.81796	22.99584	2.41249
91.347	0.27406	23.01553	0.07975	23.01553	0.67435	23.01553	1.2637	23.0155	1.81642	23.01553	2.42174
91.447	0.27423	23.03522	0.07754	23.03522	0.68067	23.03522	1.27442	23.0352	1.82112	23.03522	2.43146
91.547	0.27447	23.0549	0.07948	23.0549	0.68055	23.0549	1.27616	23.0549	1.82714	23.0549	2.43319
91.647	0.27452	23.07459	0.08571	23.07459	0.67416	23.07459	1.27092	23.0746	1.83191	23.07459	2.44516
91.747	0.27435	23.09428	0.08447	23.09428	0.67792	23.09428	1.27059	23.0943	1.8398	23.09428	2.44592

91.847	0.27437	23.11397	0.08953	23.11397	0.67817	23.11397	1.27305	23.114	1.84601	23.11397	2.4528
91.947	0.27436	23.13366	0.0863	23.13366	0.68099	23.13366	1.26576	23.1337	1.84741	23.13366	2.46283
92.047	0.27446	23.15335	0.09195	23.15335	0.68363	23.15335	1.26424	23.1534	1.85574	23.15335	2.4642
92.147	0.27443	23.17304	0.08865	23.17304	0.68984	23.17304	1.26299	23.173	1.86426	23.17304	2.47151
92.247	0.27459	23.19273	0.09204	23.19273	0.68735	23.19273	1.26111	23.1927	1.8536	23.19273	2.47293
92.347	0.27478	23.21242	0.08537	23.21242	0.69405	23.21242	1.26213	23.2124	1.86949	23.21242	2.48854
92.447	0.27444	23.23211	0.09964	23.23211	0.69068	23.23211	1.25783	23.2321	1.86139	23.23211	2.48779
92.547	0.2744	23.25179	0.09712	23.25179	0.69526	23.25179	1.25663	23.2518	1.86629	23.25179	2.49521
92.647	0.2742	23.27148	0.10109	23.27148	0.70284	23.27148	1.26147	23.2715	1.87056	23.27148	2.49488
92.747	0.27427	23.29117	0.10629	23.29117	0.70692	23.29117	1.26088	23.2912	1.86302	23.29117	2.48285
92.847	0.27436	23.31086	0.11376	23.31086	0.71213	23.31086	1.25978	23.3109	1.86017	23.31086	2.46731
92.947	0.2744	23.33055	0.12266	23.33055	0.71853	23.33055	1.25952	23.3306	1.85795	23.33055	2.46723
93.047	0.27466	23.35024	0.12444	23.35024	0.71298	23.35024	1.25971	23.3502	1.85517	23.35024	2.4589
93.147	0.27445	23.36993	0.13114	23.36993	0.71707	23.36993	1.26399	23.3699	1.84376	23.36993	2.44354
93.247	0.27454	23.38962	0.13583	23.38962	0.72003	23.38962	1.26011	23.3896	1.83872	23.38962	2.42903
93.347	0.27481	23.40931	0.13514	23.40931	0.71592	23.40931	1.26268	23.4093	1.83706	23.40931	2.42088
93.447	0.27485	23.429	0.1362	23.429	0.71482	23.429	1.25616	23.429	1.83421	23.429	2.41855
93.547	0.27499	23.44869	0.14349	23.44869	0.71277	23.44869	1.25078	23.4487	1.82693	23.44869	2.41368
93.647	0.27498	23.46838	0.14941	23.46838	0.69846	23.46838	1.25103	23.4684	1.82208	23.46838	2.40571
93.747	0.27491	23.48807	0.14321	23.48807	0.69129	23.48807	1.24596	23.4881	1.82473	23.48807	2.40938
93.847	0.27531	23.50775	0.13882	23.50775	0.67447	23.50775	1.25117	23.5078	1.82507	23.50775	2.40512
93.947	0.27561	23.52744	0.14041	23.52744	0.6735	23.52744	1.24677	23.5274	1.81479	23.52744	2.40212
94.047	0.27544	23.54713	0.13305	23.54713	0.67347	23.54713	1.23603	23.5471	1.81451	23.54713	2.40671
94.147	0.2752	23.56682	0.13075	23.56682	0.6618	23.56682	1.24909	23.5668	1.81167	23.56682	2.40274
94.247	0.2754	23.58651	0.12301	23.58651	0.6607	23.58651	1.24506	23.5865	1.80826	23.58651	2.40454
94.347	0.27534	23.6062	0.10819	23.6062	0.64447	23.6062	1.24732	23.6062	1.80935	23.6062	2.40567
94.447	0.27537	23.62589	0.09221	23.62589	0.65132	23.62589	1.24959	23.6259	1.80988	23.62589	2.40455
94.547	0.27543	23.64558	0.08356	23.64558	0.6588	23.64558	1.23812	23.6456	1.81116	23.64558	2.40611
94.647	0.27556	23.66527	0.07108	23.66527	0.65351	23.66527	1.24148	23.6653	1.80963	23.66527	2.40257
94.747	0.27567	23.68496	0.06515	23.68496	0.6491	23.68496	1.23879	23.685	1.80935	23.68496	2.40842
94.847	0.27582	23.70465	0.05746	23.70465	0.64737	23.70465	1.23889	23.7047	1.81006	23.70465	2.41567
94.947	0.27631	23.72433	0.04979	23.72433	0.64548	23.72433	1.2407	23.7243	1.81072	23.72433	2.40705
95.047	0.27639	23.74402	0.04102	23.74402	0.64527	23.74402	1.24359	23.744	1.81481	23.74402	2.41539
95.147	0.27678	23.76371	0.04477	23.76371	0.64387	23.76371	1.24307	23.7637	1.81528	23.76371	2.41949
95.247	0.27662	23.7834	0.04708	23.7834	0.6416	23.7834	1.23765	23.7834	1.8155	23.7834	2.42262
95.347	0.27686	23.80309	0.04032	23.80309	0.64508	23.80309	1.2421	23.8031	1.81297	23.80309	2.42563
95.447	0.27662	23.82278	0.03842	23.82278	0.64437	23.82278	1.24174	23.8228	1.81494	23.82278	2.43616
95.547	0.27684	23.84247	0.03912	23.84247	0.64373	23.84247	1.2438	23.8425	1.82141	23.84247	2.43365
95.647	0.27719	23.86216	0.03677	23.86216	0.64508	23.86216	1.24029	23.8622	1.81551	23.86216	2.44242
95.747	0.27738	23.88185	0.03837	23.88185	0.6465	23.88185	1.24123	23.8819	1.82141	23.88185	2.44337
95.847	0.27738	23.90154	0.03304	23.90154	0.64742	23.90154	1.24753	23.9015	1.83351	23.90154	2.45038
95.947	0.27744	23.92122	0.03855	23.92122	0.64684	23.92122	1.24924	23.9212	1.8376	23.92122	2.45115
96.047	0.27747	23.94091	0.04172	23.94091	0.64708	23.94091	1.25363	23.9409	1.83464	23.94091	2.45629
96.147	0.27765	23.9606	0.04528	23.9606	0.6492	23.9606	1.25152	23.9606	1.83811	23.9606	2.45815

96.247	0.27765	23.98029	0.0491	23.98029	0.65257	23.98029	1.2505	23.9803	1.84708	23.98029	2.46334
96.347	0.278	23.99998	0.05532	23.99998	0.65406	23.99998	1.24503	24	1.8473	23.99998	2.46824
96.447	0.2781	24.01967	0.04714	24.01967	0.65643	24.01967	1.24612	24.0197	1.8479	24.01967	2.46992
96.547	0.27835	24.03936	0.05058	24.03936	0.65473	24.03936	1.23978	24.0394	1.85824	24.03936	2.46529
96.647	0.27849	24.05905	0.04772	24.05905	0.65209	24.05905	1.23209	24.0591	1.85815	24.05905	2.47746
96.747	0.2785	24.07874	0.05517	24.07874	0.65064	24.07874	1.23122	24.0787	1.8615	24.07874	2.47599
96.847	0.27861	24.09843	0.05296	24.09843	0.64475	24.09843	1.22741	24.0984	1.86997	24.09843	2.47694
96.947	0.27931	24.11812	0.04921	24.11812	0.63824	24.11812	1.22763	24.1181	1.86201	24.11812	2.47487
97.047	0.27938	24.13781	0.04454	24.13781	0.63993	24.13781	1.22227	24.1378	1.86854	24.13781	2.47213
97.147	0.27972	24.15749	0.04053	24.15749	0.63887	24.15749	1.22069	24.1575	1.86377	24.15749	2.46448
97.247	0.27969	24.17718	0.0336	24.17718	0.63342	24.17718	1.22349	24.1772	1.86355	24.17718	2.4615
97.347	0.27982	24.19687	0.02971	24.19687	0.62573	24.19687	1.22603	24.1969	1.85803	24.19687	2.45524
97.447	0.27989	24.21656	0.02914	24.21656	0.62592	24.21656	1.22501	24.2166	1.85906	24.21656	2.45032
97.547	0.28033	24.23625	0.0213	24.23625	0.62642	24.23625	1.22285	24.2363	1.85604	24.23625	2.44776
97.647	0.28028	24.25594	0.0169	24.25594	0.62692	24.25594	1.22442	24.2559	1.8527	24.25594	2.44357
97.747	0.28103	24.27563	0.01878	24.27563	0.62373	24.27563	1.22789	24.2756	1.85399	24.27563	2.42986
97.847	0.28124	24.29532	0.02313	24.29532	0.6196	24.29532	1.22945	24.2953	1.85134	24.29532	2.42863
97.947	0.2818	24.31501	0.01466	24.31501	0.6228	24.31501	1.22988	24.315	1.84856	24.31501	2.42796
98.047	0.28181	24.3347	0.01805	24.3347	0.61774	24.3347	1.23083	24.3347	1.84929	24.3347	2.42862
98.147	0.28215	24.35439	0.01794	24.35439	0.6175	24.35439	1.23173	24.3544	1.84164	24.35439	2.42776
98.247	0.2823	24.37407	0.01899	24.37407	0.61989	24.37407	1.22989	24.3741	1.84224	24.37407	2.43157
98.347	0.28239	24.39376	0.0205	24.39376	0.6137	24.39376	1.2346	24.3938	1.8414	24.39376	2.43314
98.447	0.28263	24.41345	0.0178	24.41345	0.61715	24.41345	1.22967	24.4135	1.83825	24.41345	2.43719
98.547	0.28279	24.43314	0.02047	24.43314	0.62129	24.43314	1.23304	24.4331	1.83848	24.43314	2.43173
98.647	0.28295	24.45283	0.01692	24.45283	0.6126	24.45283	1.23352	24.4528	1.83508	24.45283	2.43245
98.747	0.28342	24.47252	0.01	24.47252	0.61493	24.47252	1.22977	24.4725	1.84361	24.47252	2.44286
98.847	0.28406	24.49221	0.01456	24.49221	0.60912	24.49221	1.23036	24.4922	1.83934	24.49221	2.43413
98.947	0.28432	24.5119	0.01089	24.5119	0.60488	24.5119	1.23064	24.5119	1.84125	24.5119	2.44291
99.047	0.28419	24.53159	0.01715	24.53159	0.6096	24.53159	1.23092	24.5316	1.84448	24.53159	2.43145
99.147	0.28487	24.55128	0.00952	24.55128	0.61437	24.55128	1.23229	24.5513	1.84076	24.55128	2.43388
99.247	0.28475	24.57096	0.01221	24.57096	0.61245	24.57096	1.22891	24.571	1.84136	24.57096	2.43587
99.347	0.28475	24.59065	0.01321	24.59065	0.60659	24.59065	1.2358	24.5907	1.84596	24.59065	2.42878
99.447	0.28482	24.61034	0.01687	24.61034	0.6123	24.61034	1.22813	24.6103	1.84419	24.61034	2.43187
99.547	0.2852	24.63003	0.01113	24.63003	0.6112	24.63003	1.22836	24.63	1.83929	24.63003	2.43004
99.647	0.28566	24.64972	0.01161	24.64972	0.60984	24.64972	1.22312	24.6497	1.83814	24.64972	2.42259
99.747	0.28573	24.66941	0.0204	24.66941	0.61086	24.66941	1.22717	24.6694	1.83081	24.66941	2.42095
99.847	0.28593	24.6891	0.01796	24.6891	0.61119	24.6891	1.22414	24.6891	1.82672	24.6891	2.41731
99.947	0.28642	24.70879	0.0121	24.70879	0.61003	24.70879	1.21896	24.7088	1.82851	24.70879	2.42549
100.047	0.28658	24.72848	0.0124	24.72848	0.60799	24.72848	1.21992	24.7285	1.8233	24.72848	2.4164
100.147	0.28689	24.74817	0.01581	24.74817	0.60978	24.74817	1.21695	24.7482	1.82184	24.74817	2.40877
100.247	0.28729	24.76786	0.01546	24.76786	0.60943	24.76786	1.21502	24.7679	1.82188	24.76786	2.41592
100.347	0.28715	24.78755	0.01037	24.78755	0.61083	24.78755	1.21458	24.7876	1.81461	24.78755	2.41804
100.447	0.28744	24.80723	0.01112	24.80723	0.61224	24.80723	1.21218	24.8072	1.81552	24.80723	2.40695
100.547	0.28752	24.82692	0.01136	24.82692	0.60876	24.82692	1.21686	24.8269	1.80975	24.82692	2.41283

100.647	0.28798	24.84661	0.01924	24.84661	0.61311	24.84661	1.20961	24.8466	1.80885	24.84661	2.40853
100.747	0.2883	24.8663	0.02038	24.8663	0.61564	24.8663	1.2086	24.8663	1.8052	24.8663	2.40811
100.847	0.28831	24.88599	0.02321	24.88599	0.60991	24.88599	1.20347	24.886	1.80286	24.88599	2.40254
100.947	0.28832	24.90568	0.01988	24.90568	0.62021	24.90568	1.21083	24.9057	1.79853	24.90568	2.39776
101.047	0.28871	24.92537	0.03199	24.92537	0.61837	24.92537	1.20736	24.9254	1.79419	24.92537	2.39783
101.147	0.28856	24.94506	0.03295	24.94506	0.62667	24.94506	1.2076	24.9451	1.79217	24.94506	2.39899
101.247	0.28888	24.96475	0.04305	24.96475	0.6237	24.96475	1.20531	24.9648	1.79464	24.96475	2.40105
101.347	0.28923	24.98444	0.05152	24.98444	0.63199	24.98444	1.20761	24.9844	1.79587	24.98444	2.39197
101.447	0.28932	25.00413	0.05708	25.00413	0.64386	25.00413	1.21513	25.0041	1.79541	25.00413	2.39428
101.547	0.28946	25.02382	0.0653	25.02382	0.63832	25.02382	1.20644	25.0238	1.80088	25.02382	2.39992
101.647	0.28985	25.0435	0.06917	25.0435	0.64756	25.0435	1.20172	25.0435	1.79611	25.0435	2.40145
101.747	0.29022	25.06319	0.07707	25.06319	0.65135	25.06319	1.20465	25.0632	1.79403	25.06319	2.39891
101.847	0.29054	25.08288	0.08781	25.08288	0.65208	25.08288	1.21057	25.0829	1.8	25.08288	2.41025
101.947	0.29061	25.10257	0.09001	25.10257	0.65781	25.10257	1.20771	25.1026	1.80208	25.10257	2.40681
102.047	0.2907	25.12226	0.10083	25.12226	0.66149	25.12226	1.20878	25.1223	1.80277	25.12226	2.40809
102.147	0.29104	25.14195	0.11528	25.14195	0.66629	25.14195	1.21228	25.142	1.80441	25.14195	2.41199
102.247	0.29138	25.16164	0.11864	25.16164	0.67321	25.16164	1.2087	25.1616	1.8068	25.16164	2.40806
102.347	0.29158	25.18133	0.10904	25.18133	0.66925	25.18133	1.20951	25.1813	1.80462	25.18133	2.4105
102.447	0.29171	25.20102	0.117	25.20102	0.67512	25.20102	1.21234	25.201	1.80864	25.20102	2.41966
102.547	0.29215	25.22071	0.12776	25.22071	0.66809	25.22071	1.20882	25.2207	1.8134	25.22071	2.42356
102.647	0.29242	25.24039	0.1175	25.24039	0.66819	25.24039	1.20916	25.2404	1.81147	25.24039	2.43024
102.747	0.29284	25.26008	0.11548	25.26008	0.6718	25.26008	1.21323	25.2601	1.81892	25.26008	2.42873
102.847	0.29294	25.27977	0.10944	25.27977	0.66772	25.27977	1.21058	25.2798	1.81637	25.27977	2.42705
102.947	0.29395	25.29946	0.09945	25.29946	0.65951	25.29946	1.20659	25.2995	1.82076	25.29946	2.43919
103.047	0.29424	25.31915	0.09899	25.31915	0.664	25.31915	1.2087	25.3192	1.8204	25.31915	2.43576
103.147	0.29442	25.33884	0.09523	25.33884	0.66055	25.33884	1.20848	25.3388	1.8256	25.33884	2.43899
103.247	0.29459	25.35853	0.10067	25.35853	0.65897	25.35853	1.20997	25.3585	1.83367	25.35853	2.43961
103.347	0.29471	25.37822	0.10113	25.37822	0.66478	25.37822	1.21125	25.3782	1.83693	25.37822	2.44188
103.447	0.29547	25.39791	0.09321	25.39791	0.66345	25.39791	1.21831	25.3979	1.83238	25.39791	2.44159
103.547	0.29593	25.4176	0.09431	25.4176	0.66369	25.4176	1.21474	25.4176	1.84127	25.4176	2.44355
103.647	0.29632	25.43729	0.09217	25.43729	0.67425	25.43729	1.21762	25.4373	1.83997	25.43729	2.45005
103.747	0.29688	25.45697	0.09372	25.45697	0.66854	25.45697	1.21906	25.457	1.84436	25.45697	2.4388
103.847	0.29731	25.47666	0.08749	25.47666	0.66258	25.47666	1.21884	25.4767	1.84993	25.47666	2.4447
103.947	0.29802	25.49635	0.07965	25.49635	0.66214	25.49635	1.22038	25.4964	1.84695	25.49635	2.44667
104.047	0.29842	25.51604	0.0674	25.51604	0.66801	25.51604	1.22383	25.516	1.84309	25.51604	2.45263
104.147	0.29911	25.53573	0.06344	25.53573	0.66256	25.53573	1.2231	25.5357	1.85023	25.53573	2.45017
104.247	0.29965	25.55542	0.05203	25.55542	0.66149	25.55542	1.22279	25.5554	1.85012	25.55542	2.45129
104.347	0.3	25.57511	0.03673	25.57511	0.65009	25.57511	1.22598	25.5751	1.85001	25.57511	2.44556
104.447	0.30064	25.5948	0.03453	25.5948	0.65121	25.5948	1.23212	25.5948	1.85127	25.5948	2.44692
104.547	0.30123	25.61449	0.02649	25.61449	0.63944	25.61449	1.23567	25.6145	1.85622	25.61449	2.44894
104.647	0.30208	25.63418	0.0182	25.63418	0.63512	25.63418	1.23944	25.6342	1.85499	25.63418	2.44727
104.747	0.30222	25.65387	0.01969	25.65387	0.63473	25.65387	1.23457	25.6539	1.858	25.65387	2.44596
104.847	0.30266	25.67356	0.00575	25.67356	0.62521	25.67356	1.23715	25.6736	1.85708	25.67356	2.45168
104.947	0.30348	25.69324	0.00537	25.69324	0.62415	25.69324	1.23874	25.6932	1.85941	25.69324	2.45547

105.047	0.30376	25.71293	0.00505	25.71293	0.62321	25.71293	1.24318	25.7129	1.85867	25.71293	2.45811
105.147	0.30439	25.73262	0.00357	25.73262	0.62721	25.73262	1.24571	25.7326	1.85881	25.73262	2.46002
105.247	0.30488	25.75231	0.00358	25.75231	0.62395	25.75231	1.2472	25.7523	1.86126	25.75231	2.4666
105.347	0.30488	25.772	0.00157	25.772	0.62282	25.772	1.25184	25.772	1.86384	25.772	2.47342
105.447	0.30511	25.79169	0.00534	25.79169	0.62601	25.79169	1.25483	25.7917	1.86586	25.79169	2.48024
105.547	0.30569	25.81138	0.00716	25.81138	0.62888	25.81138	1.2557	25.8114	1.86206	25.81138	2.47712
105.647	0.30585	25.83107	0.01255	25.83107	0.62957	25.83107	1.25229	25.8311	1.8612	25.83107	2.48776
105.747	0.30679	25.85076	0.01302	25.85076	0.63232	25.85076	1.25807	25.8508	1.8596	25.85076	2.497
105.847	0.30684	25.87045	0.02243	25.87045	0.63251	25.87045	1.25455	25.8705	1.86186	25.87045	2.49643
105.947	0.30708	25.89013	0.01881	25.89013	0.63301	25.89013	1.25408	25.8901	1.86732	25.89013	2.49519
106.047	0.30727	25.90982	0.01616	25.90982	0.63964	25.90982	1.25	25.9098	1.86452	25.90982	2.49342
106.147	0.30733	25.92951	0.02661	25.92951	0.63438	25.92951	1.23735	25.9295	1.87279	25.92951	2.49509
106.247	0.30779	25.9492	0.02844	25.9492	0.63294	25.9492	1.23853	25.9492	1.87143	25.9492	2.50004
106.347	0.30821	25.96889	0.03091	25.96889	0.62825	25.96889	1.2377	25.9689	1.87126	25.96889	2.49765
106.447	0.30809	25.98858	0.03267	25.98858	0.62525	25.98858	1.23088	25.9886	1.87665	25.98858	2.50466
106.547	0.30779	26.00827	0.03469	26.00827	0.6198	26.00827	1.23305	26.0083	1.87255	26.00827	2.49979
106.647	0.30837	26.02796	0.03717	26.02796	0.6183	26.02796	1.23227	26.028	1.88525	26.02796	2.4997
106.747	0.30851	26.04765	0.03757	26.04765	0.61686	26.04765	1.22576	26.0477	1.88877	26.04765	2.49853
106.847	0.30902	26.06734	0.03803	26.06734	0.61561	26.06734	1.22437	26.0673	1.89366	26.06734	2.4996
106.947	0.30924	26.08703	0.03163	26.08703	0.60203	26.08703	1.22318	26.087	1.88806	26.08703	2.49643
107.047	0.30903	26.10671	0.03702	26.10671	0.60961	26.10671	1.2224	26.1067	1.89001	26.10671	2.50084
107.147	0.30889	26.1264	0.02647	26.1264	0.60367	26.1264	1.2177	26.1264	1.89522	26.1264	2.48385
107.247	0.30929	26.14609	0.01993	26.14609	0.6021	26.14609	1.21909	26.1461	1.89799	26.14609	2.4882
107.347	0.30907	26.16578	0.01618	26.16578	0.60035	26.16578	1.21889	26.1658	1.89682	26.16578	2.48406
107.447	0.30916	26.18547	0.01217	26.18547	0.60668	26.18547	1.21955	26.1855	1.89072	26.18547	2.47156
107.547	0.3094	26.20516	0.00369	26.20516	0.60443	26.20516	1.21392	26.2052	1.89449	26.20516	2.47112
107.647	0.30956	26.22485	0.00474	26.22485	0.6085	26.22485	1.21537	26.2249	1.89694	26.22485	2.46995
107.747	0.30957	26.24454	-0.00192	26.24454	0.60237	26.24454	1.21361	26.2445	1.90128	26.24454	2.45127
107.847	0.30961	26.26423	4.25E-04	26.26423	0.60381	26.26423	1.20979	26.2642	1.89573	26.26423	2.45107
107.947	0.30943	26.28392	-0.00235	26.28392	0.60331	26.28392	1.21201	26.2839	1.89388	26.28392	2.45197
108.047	0.30976	26.30361	-7.78E-04	26.30361	0.59712	26.30361	1.21206	26.3036	1.89234	26.30361	2.44511
108.147	0.30942	26.3233	-0.00141	26.3233	0.60276	26.3233	1.20927	26.3233	1.88611	26.3233	2.43771
108.247	0.30947	26.34299	-0.00438	26.34299	0.60201	26.34299	1.21051	26.343	1.88601	26.34299	2.43915
108.347	0.30968	26.36267	4.32E-04	26.36267	0.6032	26.36267	1.20767	26.3627	1.87685	26.36267	2.44217
108.447	0.30967	26.38236	0.00272	26.38236	0.60683	26.38236	1.2103	26.3824	1.87443	26.38236	2.43804
108.547	0.30952	26.40205	0.00267	26.40205	0.60427	26.40205	1.20901	26.4021	1.86714	26.40205	2.437
108.647	0.30963	26.42174	0.00262	26.42174	0.60183	26.42174	1.20607	26.4217	1.86817	26.42174	2.4402
108.747	0.30977	26.44143	0.00496	26.44143	0.6111	26.44143	1.20447	26.4414	1.85857	26.44143	2.44055
108.847	0.3099	26.46112	4.40E-04	26.46112	0.60591	26.46112	1.2039	26.4611	1.8569	26.46112	2.44176
108.947	0.30987	26.48081	0.00175	26.48081	0.60091	26.48081	1.20695	26.4808	1.85649	26.48081	2.43745
109.047	0.31006	26.5005	0.0076	26.5005	0.60279	26.5005	1.2086	26.5005	1.85739	26.5005	2.44078
109.147	0.30998	26.52019	2.88E-05	26.52019	0.60561	26.52019	1.20638	26.5202	1.85197	26.52019	2.44738
109.247	0.31015	26.53988	0.00153	26.53988	0.60298	26.53988	1.20876	26.5399	1.85094	26.53988	2.44743
109.347	0.31036	26.55956	0.00129	26.55956	0.60749	26.55956	1.2037	26.5596	1.85746	26.55956	2.44634

109.447	0.3106	26.57925	-0.002	26.57925	0.59967	26.57925	1.20917	26.5793	1.86355	26.57925	2.45264
109.547	0.31106	26.59894	0.00573	26.59894	0.60337	26.59894	1.20726	26.5989	1.8577	26.59894	2.44784
109.647	0.31112	26.61863	0.006	26.61863	0.60012	26.61863	1.20349	26.6186	1.85566	26.61863	2.45178
109.747	0.3112	26.63832	1.80E-04	26.63832	0.60244	26.63832	1.20886	26.6383	1.86193	26.63832	2.45856
109.847	0.31132	26.65801	0	26.65801	0.6	26.65801	1.20003	26.658	1.86396	26.65801	2.46189
109.947	0.3115	26.6777	0.00335	26.6777	0.60142	26.6777	1.2084	26.6777	1.85955	26.6777	2.45389
110.047	0.31177	26.69739	0.00909	26.69739	0.59596	26.69739	1.20411	26.6974	1.8572	26.69739	2.46019
110.147	0.31224	26.71708	0.0068	26.71708	0.60082	26.71708	1.20664	26.7171	1.85935	26.71708	2.45589
110.247	0.31239	26.73677	0.00386	26.73677	0.60424	26.73677	1.20572	26.7368	1.85606	26.73677	2.46177
110.347	0.31264	26.75646	0.00818	26.75646	0.60428	26.75646	1.21057	26.7565	1.85172	26.75646	2.4525
110.447	0.31301	26.77614	0.0053	26.77614	0.6017	26.77614	1.21088	26.7761	1.85599	26.77614	2.45583
110.547	0.31319	26.79583	0.00741	26.79583	0.6115	26.79583	1.21228	26.7958	1.85015	26.79583	2.44723
110.647	0.31372	26.81552	0.0175	26.81552	0.61198	26.81552	1.20908	26.8155	1.83992	26.81552	2.43838
110.747	0.31377	26.83521	0.01878	26.83521	0.6129	26.83521	1.20861	26.8352	1.8387	26.83521	2.43347
110.847	0.31451	26.8549	0.01246	26.8549	0.61288	26.8549	1.2096	26.8549	1.83298	26.8549	2.42426
110.947	0.3141	26.87459	0.01555	26.87459	0.61831	26.87459	1.21476	26.8746	1.83432	26.87459	2.42541
111.047	0.31403	26.89428	0.01838	26.89428	0.61948	26.89428	1.21187	26.8943	1.83016	26.89428	2.41912
111.147	0.31462	26.91397	0.02134	26.91397	0.6239	26.91397	1.21141	26.914	1.82488	26.91397	2.41476
111.247	0.31489	26.93366	0.02021	26.93366	0.62182	26.93366	1.20816	26.9337	1.82516	26.93366	2.40767
111.347	0.31492	26.95335	0.02434	26.95335	0.62243	26.95335	1.21053	26.9534	1.81756	26.95335	2.41483
111.447	0.3154	26.97304	0.02088	26.97304	0.62435	26.97304	1.21229	26.973	1.81509	26.97304	2.40453
111.547	0.31594	26.99273	0.02007	26.99273	0.62789	26.99273	1.20976	26.9927	1.81081	26.99273	2.40653
111.647	0.31628	27.01241	0.02329	27.01241	0.62381	27.01241	1.2124	27.0124	1.81184	27.01241	2.40012
111.747	0.31672	27.0321	0.0269	27.0321	0.6174	27.0321	1.21209	27.0321	1.81018	27.0321	2.40757
111.847	0.31703	27.05179	0.02006	27.05179	0.61582	27.05179	1.21286	27.0518	1.80509	27.05179	2.40897
111.947	0.31747	27.07148	0.02101	27.07148	0.61568	27.07148	1.20884	27.0715	1.80905	27.07148	2.4065
112.047	0.31781	27.09117	0.02663	27.09117	0.61628	27.09117	1.21023	27.0912	1.80834	27.09117	2.41511
112.147	0.31838	27.11086	0.02272	27.11086	0.61345	27.11086	1.20879	27.1109	1.80755	27.11086	2.41155
112.247	0.31873	27.13055	0.02905	27.13055	0.60905	27.13055	1.21034	27.1306	1.81152	27.13055	2.41434
112.347	0.31895	27.15024	0.02844	27.15024	0.60816	27.15024	1.20895	27.1502	1.80762	27.15024	2.41187
112.447	0.31938	27.16993	0.02673	27.16993	0.60482	27.16993	1.21458	27.1699	1.81046	27.16993	2.40957
112.547	0.31953	27.18962	0.02502	27.18962	0.611	27.18962	1.21174	27.1896	1.80755	27.18962	2.41607
112.647	0.31983	27.2093	0.01625	27.2093	0.60172	27.2093	1.21143	27.2093	1.81158	27.2093	2.41577
112.747	0.32033	27.22899	0.0229	27.22899	0.60376	27.22899	1.20508	27.229	1.82261	27.22899	2.41851
112.847	0.32103	27.24868	0.01594	27.24868	0.60593	27.24868	1.20994	27.2487	1.81627	27.24868	2.42016
112.947	0.32132	27.26837	0.00898	27.26837	0.60272	27.26837	1.20953	27.2684	1.81805	27.26837	2.42441
113.047	0.32175	27.28806	0.01252	27.28806	0.6176	27.28806	1.20669	27.2881	1.81746	27.28806	2.41958
113.147	0.32204	27.30775	0.01159	27.30775	0.60795	27.30775	1.21144	27.3078	1.81893	27.30775	2.42795
113.247	0.32247	27.32744	0.0106	27.32744	0.61012	27.32744	1.2084	27.3274	1.81471	27.32744	2.42663
113.347	0.3229	27.34713	0.01122	27.34713	0.61016	27.34713	1.21165	27.3471	1.82062	27.34713	2.42646
113.447	0.32305	27.36682	0.0064	27.36682	0.61565	27.36682	1.21109	27.3668	1.8224	27.36682	2.42575
113.547	0.32369	27.38651	0.00612	27.38651	0.61432	27.38651	1.20815	27.3865	1.81575	27.38651	2.42473
113.647	0.32405	27.4062	0.01102	27.4062	0.6123	27.4062	1.21006	27.4062	1.81578	27.4062	2.42511
113.747	0.32468	27.42588	0.00906	27.42588	0.60965	27.42588	1.21378	27.4259	1.81407	27.42588	2.42233

113.847	0.3249	27.44557	0.01617	27.44557	0.61414	27.44557	1.21486	27.4456	1.81354	27.44557	2.42453
113.947	0.32533	27.46526	0.01958	27.46526	0.61287	27.46526	1.20934	27.4653	1.8207	27.46526	2.4249
114.047	0.3259	27.48495	0.02209	27.48495	0.61248	27.48495	1.21497	27.485	1.81411	27.48495	2.42274
114.147	0.32633	27.50464	0.02265	27.50464	0.62097	27.50464	1.21389	27.5046	1.81945	27.50464	2.42354
114.247	0.32766	27.52433	0.02237	27.52433	0.62195	27.52433	1.21353	27.5243	1.81961	27.52433	2.42635
114.347	0.32761	27.54402	0.02702	27.54402	0.62381	27.54402	1.21601	27.544	1.81502	27.54402	2.4306
114.447	0.32748	27.56371	0.02576	27.56371	0.62705	27.56371	1.21508	27.5637	1.82262	27.56371	2.42535
114.547	0.32776	27.5834	0.02639	27.5834	0.62453	27.5834	1.21813	27.5834	1.82053	27.5834	2.43512
114.647	0.3282	27.60309	0.03311	27.60309	0.62758	27.60309	1.21597	27.6031	1.82219	27.60309	2.43592
114.747	0.32903	27.62278	0.03595	27.62278	0.6265	27.62278	1.21793	27.6228	1.82878	27.62278	2.4337
114.847	0.32933	27.64247	0.03943	27.64247	0.62629	27.64247	1.21582	27.6425	1.82782	27.64247	2.43414
114.947	0.32982	27.66215	0.04855	27.66215	0.62972	27.66215	1.21783	27.6622	1.8331	27.66215	2.43803
115.047	0.33039	27.68184	0.05197	27.68184	0.63164	27.68184	1.2137	27.6818	1.82864	27.68184	2.4389
115.147	0.3306	27.70153	0.05785	27.70153	0.62468	27.70153	1.2165	27.7015	1.82974	27.70153	2.43407
115.247	0.33117	27.72122	0.06087	27.72122	0.6276	27.72122	1.21087	27.7212	1.83002	27.72122	2.43445
115.347	0.33154	27.74091	0.06585	27.74091	0.62615	27.74091	1.20819	27.7409	1.82981	27.74091	2.43683
115.447	0.33177	27.7606	0.06894	27.7606	0.63026	27.7606	1.20571	27.7606	1.83503	27.7606	2.43424
115.547	0.33198	27.78029	0.07709	27.78029	0.62874	27.78029	1.2037	27.7803	1.83919	27.78029	2.43602
115.647	0.33209	27.79998	0.07778	27.79998	0.6301	27.79998	1.2005	27.8	1.83254	27.79998	2.43337
115.747	0.33387	27.81967	0.08392	27.81967	0.63221	27.81967	1.20221	27.8197	1.83264	27.81967	2.43199
115.847	0.33368	27.83936	0.07839	27.83936	0.62957	27.83936	1.20655	27.8394	1.83199	27.83936	2.42177
115.947	0.33402	27.85905	0.0822	27.85905	0.62379	27.85905	1.20903	27.8591	1.83221	27.85905	2.42682
116.047	0.33425	27.87873	0.07868	27.87873	0.63047	27.87873	1.20175	27.8787	1.82325	27.87873	2.42247
116.147	0.33426	27.89842	0.07937	27.89842	0.62626	27.89842	1.20278	27.8984	1.82585	27.89842	2.4251
116.247	0.33464	27.91811	0.08558	27.91811	0.63007	27.91811	1.20118	27.9181	1.82657	27.91811	2.41967
116.347	0.33519	27.9378	0.08076	27.9378	0.63631	27.9378	1.20491	27.9378	1.8213	27.9378	2.42108
116.447	0.33539	27.95749	0.08197	27.95749	0.63172	27.95749	1.20682	27.9575	1.82602	27.95749	2.41922
116.547	0.33556	27.97718	0.07955	27.97718	0.63496	27.97718	1.20063	27.9772	1.816	27.97718	2.41754
116.647	0.33613	27.99687	0.07817	27.99687	0.62938	27.99687	1.20171	27.9969	1.81535	27.99687	2.4178
116.747	0.33692	28.01656	0.07433	28.01656	0.6405	28.01656	1.20295	28.0166	1.81645	28.01656	2.41643
116.847	0.33719	28.03625	0.07217	28.03625	0.63623	28.03625	1.20084	28.0363	1.82261	28.03625	2.41863
116.947	0.33746	28.05594	0.06865	28.05594	0.64147	28.05594	1.2027	28.0559	1.81459	28.05594	2.41774
117.047	0.33787	28.07562	0.0698	28.07562	0.633	28.07562	1.20245	28.0756	1.81506	28.07562	2.41018
117.147	0.33781	28.09531	0.0631	28.09531	0.63368	28.09531	1.19744	28.0953	1.8206	28.09531	2.41269
117.247	0.33785	28.115	0.06775	28.115	0.63279	28.115	1.20018	28.115	1.8137	28.115	2.41477
117.347	0.33839	28.13469	0.0628	28.13469	0.63909	28.13469	1.19786	28.1347	1.81992	28.13469	2.41346
117.447	0.33865	28.15438	0.05714	28.15438	0.6372	28.15438	1.20101	28.1544	1.81702	28.15438	2.41481
117.547	0.33891	28.17407	0.05914	28.17407	0.63887	28.17407	1.20535	28.1741	1.80975	28.17407	2.41198
117.647	0.33966	28.19376	0.05555	28.19376	0.63291	28.19376	1.20205	28.1938	1.81579	28.19376	2.41552
117.747	0.34025	28.21345	0.05061	28.21345	0.63352	28.21345	1.20546	28.2135	1.8147	28.21345	2.41184
117.847	0.34072	28.23314	0.0489	28.23314	0.63138	28.23314	1.20118	28.2331	1.81649	28.23314	2.41228
117.947	0.34108	28.25283	0.0461	28.25283	0.63424	28.25283	1.20046	28.2528	1.81165	28.25283	2.40515
118.047	0.34138	28.27252	0.04627	28.27252	0.62866	28.27252	1.20505	28.2725	1.81094	28.27252	2.41148
118.147	0.34197	28.29221	0.04269	28.29221	0.62964	28.29221	1.2081	28.2922	1.81873	28.29221	2.40804

118.247	0.34238	28.31189	0.04955	28.31189	0.62913	28.31189	1.20955	28.3119	1.81814	28.31189	2.4123
118.347	0.34244	28.33158	0.04337	28.33158	0.6313	28.33158	1.20728	28.3316	1.81343	28.33158	2.4056
118.447	0.34282	28.35127	0.04348	28.35127	0.62835	28.35127	1.20863	28.3513	1.81503	28.35127	2.4061
118.547	0.34372	28.37096	0.04723	28.37096	0.63027	28.37096	1.20935	28.371	1.81069	28.37096	2.40461
118.647	0.34428	28.39065	0.03846	28.39065	0.63358	28.39065	1.21121	28.3907	1.80911	28.39065	2.40735
118.747	0.34484	28.41034	0.03546	28.41034	0.62668	28.41034	1.21189	28.4103	1.80546	28.41034	2.40077
118.847	0.34552	28.43003	0.04426	28.43003	0.62366	28.43003	1.21447	28.43	1.80081	28.43003	2.4
118.947	0.34619	28.44972	0.03828	28.44972	0.61895	28.44972	1.21479	28.4497	1.80803	28.44972	2.40146
119.047	0.34684	28.46941	0.04079	28.46941	0.62175	28.46941	1.21355	28.4694	1.80126	28.46941	2.39989
119.147	0.34715	28.4891	0.035	28.4891	0.61867	28.4891	1.21505	28.4891	1.80073	28.4891	2.40262
119.247	0.34755	28.50879	0.02701	28.50879	0.61966	28.50879	1.21583	28.5088	1.80265	28.50879	2.40323
119.347	0.34818	28.52847	0.02829	28.52847	0.61307	28.52847	1.21919	28.5285	1.7995	28.52847	2.39797
119.447	0.34881	28.54816	0.02756	28.54816	0.61925	28.54816	1.22161	28.5482	1.80103	28.54816	2.4024
119.547	0.34932	28.56785	0.02722	28.56785	0.61811	28.56785	1.21454	28.5679	1.80264	28.56785	2.39586
119.647	0.34993	28.58754	0.02611	28.58754	0.61697	28.58754	1.22131	28.5875	1.79555	28.58754	2.40332
119.747	0.35074	28.60723	0.0233	28.60723	0.61546	28.60723	1.22023	28.6072	1.79809	28.60723	2.39617
119.847	0.35154	28.62692	0.02128	28.62692	0.61444	28.62692	1.21873	28.6269	1.8	28.62692	2.40127
119.947	0.35195	28.64661	0.02048	28.64661	0.61781	28.64661	1.21554	28.6466	1.80245	28.64661	2.40194
120.047	0.35271	28.6663	0.01787	28.6663	0.60772	28.6663	1.22442	28.6663	1.80083	28.6663	2.40037
120.147	0.35304	28.68599	0.01844	28.68599	0.61559	28.68599	1.2175	28.686	1.79677	28.68599	2.39953
120.247	0.35423	28.70568	0.01681	28.70568	0.61151	28.70568	1.21792	28.7057	1.80047	28.70568	2.40105
120.347	0.35414	28.72536	0.0201	28.72536	0.60855	28.72536	1.21699	28.7254	1.80223	28.72536	2.40117
120.447	0.35479	28.74505	0.0206	28.74505	0.60923	28.74505	1.21963	28.7451	1.80292	28.74505	2.40469
120.547	0.35526	28.76474	0.01365	28.76474	0.60596	28.76474	1.2185	28.7647	1.80355	28.76474	2.40846
120.647	0.3557	28.78443	0.01539	28.78443	0.60882	28.78443	1.21685	28.7844	1.80644	28.78443	2.40695
120.747	0.35619	28.80412	0.01278	28.80412	0.612	28.80412	1.21737	28.8041	1.81269	28.80412	2.41447
120.847	0.35673	28.82381	0.01374	28.82381	0.61148	28.82381	1.21665	28.8238	1.81333	28.82381	2.41048
120.947	0.35774	28.8435	0.01048	28.8435	0.61429	28.8435	1.21479	28.8435	1.81146	28.8435	2.414
121.047	0.35797	28.86319	0.00768	28.86319	0.60927	28.86319	1.21376	28.8632	1.8144	28.86319	2.41382
121.147	0.35808	28.88288	0.01	28.88288	0.60894	28.88288	1.21387	28.8829	1.8126	28.88288	2.41577
121.247	0.35854	28.90257	0.01725	28.90257	0.61293	28.90257	1.21237	28.9026	1.81492	28.90257	2.41565
121.347	0.35881	28.92226	0.01639	28.92226	0.61517	28.92226	1.21485	28.9223	1.81355	28.92226	2.41736
121.447	0.35873	28.94195	0.01216	28.94195	0.61172	28.94195	1.21067	28.942	1.81837	28.94195	2.4173
121.547	0.3594	28.96164	0.01364	28.96164	0.61715	28.96164	1.20732	28.9616	1.81825	28.96164	2.41579
121.647	0.35954	28.98132	0.01531	28.98132	0.60969	28.98132	1.21161	28.9813	1.8192	28.98132	2.42598
121.747	0.35999	29.00101	0.01283	29.00101	0.61042	29.00101	1.21218	29.001	1.8194	29.00101	2.42423
121.847	0.36035	29.0207	0.01243	29.0207	0.61379	29.0207	1.20784	29.0207	1.81684	29.0207	2.42163
121.947	0.36086	29.04039	0.01961	29.04039	0.60733	29.04039	1.21249	29.0404	1.8146	29.04039	2.41218
122.047	0.3613	29.06008	0.01999	29.06008	0.60732	29.06008	1.21146	29.0601	1.81492	29.06008	2.41898
122.147	0.36133	29.07977	0.02088	29.07977	0.60661	29.07977	1.20511	29.0798	1.81568	29.07977	2.41517
122.247	0.36186	29.09946	0.01828	29.09946	0.60209	29.09946	1.20734	29.0995	1.81369	29.09946	2.42063
122.347	0.36187	29.11915	0.01191	29.11915	0.61109	29.11915	1.20729	29.1192	1.81176	29.11915	2.41536
122.447	0.36252	29.13884	0.01871	29.13884	0.60426	29.13884	1.20626	29.1388	1.81045	29.13884	2.41131
122.547	0.36273	29.15853	0.01324	29.15853	0.61	29.15853	1.20554	29.1585	1.8164	29.15853	2.4164

122.647	0.36269	29.17822	0.01206	29.17822	0.60423	29.17822	1.20317	29.1782	1.81165	29.17822	2.41174
122.747	0.36307	29.1979	0.00972	29.1979	0.60641	29.1979	1.20555	29.1979	1.81154	29.1979	2.41399
122.847	0.3635	29.21759	0.00893	29.21759	0.60714	29.21759	1.21128	29.2176	1.81329	29.21759	2.41085
122.947	0.36416	29.23728	0.01021	29.23728	0.60669	29.23728	1.20457	29.2373	1.80912	29.23728	2.40971
123.047	0.36441	29.25697	0.00883	29.25697	0.6028	29.25697	1.20426	29.257	1.81418	29.25697	2.4185
123.147	0.3646	29.27666	9.76E-04	29.27666	0.60304	29.27666	1.20623	29.2767	1.81438	29.27666	2.4162
123.247	0.36493	29.29635	0.00628	29.29635	0.6054	29.29635	1.20726	29.2964	1.81058	29.29635	2.41839
123.347	0.36499	29.31604	0.00568	29.31604	0.60689	29.31604	1.20923	29.316	1.81821	29.31604	2.41561
123.447	0.36537	29.33573	0.0034	29.33573	0.60525	29.33573	1.20169	29.3357	1.81372	29.33573	2.41368
123.547	0.36565	29.35542	4.06E-04	29.35542	0.60398	29.35542	1.20133	29.3554	1.81191	29.35542	2.41496
123.647	0.36586	29.3751	4.58E-04	29.3751	0.60666	29.3751	1.20459	29.3751	1.80886	29.3751	2.41327
123.747	0.36577	29.39479	-3.98E-04	29.39479	0.60108	29.39479	1.19798	29.3948	1.80987	29.39479	2.41273
123.847	0.36648	29.41448	1.08E-04	29.41448	0.6	29.41448	1.2	29.4145	1.81494	29.41448	2.4185
123.947	0.36662	29.43417	0.00386	29.43417	0.59944	29.43417	1.20436	29.4342	1.81382	29.43417	2.41893
124.047	0.3667	29.45386	-0.00108	29.45386	0.59919	29.45386	1.20257	29.4539	1.81451	29.45386	2.41767
124.147	0.36668	29.47355	-0.00278	29.47355	0.6017	29.47355	1.2042	29.4736	1.81577	29.47355	2.41319
124.247	0.36704	29.49324	0.00486	29.49324	0.6042	29.49324	1.20504	29.4932	1.81397	29.49324	2.41865
124.347	0.36729	29.51293	0.00141	29.51293	0.60339	29.51293	1.20383	29.5129	1.81291	29.51293	2.41969
124.447	0.3676	29.53262	0.00522	29.53262	0.60427	29.53262	1.20581	29.5326	1.81011	29.53262	2.42085
124.547	0.36785	29.55231	-0.00102	29.55231	0.60152	29.55231	1.20325	29.5523	1.81593	29.55231	2.41559
124.647	0.36803	29.572	0.00669	29.572	0.60328	29.572	1.20307	29.572	1.81056	29.572	2.41505
124.747	0.36829	29.59169	0.00635	29.59169	0.6019	29.59169	1.20319	29.5917	1.81338	29.59169	2.41524
124.847	0.36827	29.61138	0.00375	29.61138	0.60604	29.61138	1.20156	29.6114	1.81439	29.61138	2.41901
124.947	0.3686	29.63106	0.00548	29.63106	0.60641	29.63106	1.20024	29.6311	1.80915	29.63106	2.41344
125.047	0.36903	29.65075	0.00696	29.65075	0.60867	29.65075	1.19897	29.6508	1.81103	29.65075	2.4129
125.147	0.36954	29.67044	0.00617	29.67044	0.60511	29.67044	1.20385	29.6704	1.81166	29.67044	2.41431
125.247	0.37011	29.69013	0.00804	29.69013	0.6083	29.69013	1.20521	29.6901	1.8063	29.69013	2.41298
125.347	0.37059	29.70982	-1.15E-05	29.70982	0.60987	29.70982	1.20002	29.7098	1.80849	29.70982	2.40693
125.447	0.37095	29.72951	0.00769	29.72951	0.60486	29.72951	1.2004	29.7295	1.80537	29.72951	2.40773
125.547	0.37138	29.7492	0.00781	29.7492	0.60825	29.7492	1.20533	29.7492	1.80601	29.7492	2.40689
125.647	0.37184	29.76889	0.00935	29.76889	0.606	29.76889	1.20319	29.7689	1.81008	29.76889	2.40441
125.747	0.37185	29.78858	0.00766	29.78858	0.60099	29.78858	1.2046	29.7886	1.8079	29.78858	2.40388
125.847	0.37191	29.80827	0.01056	29.80827	0.606	29.80827	1.20282	29.8083	1.80641	29.80827	2.40504
125.947	0.37216	29.82796	6.29E-04	29.82796	0.60989	29.82796	1.19788	29.828	1.8001	29.82796	2.39983
126.047	0.37227	29.84764	0.00379	29.84764	0.60613	29.84764	1.20638	29.8476	1.8018	29.84764	2.39936
126.147	0.37268	29.86733	0.00488	29.86733	0.60952	29.86733	1.2064	29.8673	1.80093	29.86733	2.40525
126.247	0.373	29.88702	0.00571	29.88702	0.60214	29.88702	1.20327	29.887	1.79819	29.88702	2.3972
126.347	0.37342	29.90671	0.01206	29.90671	0.6052	29.90671	1.20453	29.9067	1.80332	29.90671	2.40242
126.447	0.37339	29.9264	0.00543	29.9264	0.60521	29.9264	1.20373	29.9264	1.8022	29.9264	2.4037
126.547	0.37368	29.94609	0.00924	29.94609	0.60265	29.94609	1.20809	29.9461	1.8069	29.94609	2.4028
126.647	0.37458	29.96578	0.00223	29.96578	0.6019	29.96578	1.20496	29.9658	1.80253	29.96578	2.40354
126.747	0.37493	29.98547	0.00176	29.98547	0.59996	29.98547	1.20442	29.9855	1.80173	29.98547	2.40422
126.847	0.375	30.00516	0	30.00516	0.60021	30.00516	1.20439	30.0052	1.80055	30.00516	2.40271
126.947	0.37533	30.02485	-2.79E-04	30.02485	0.59859	30.02485	1.20751	30.0249	1.8048	30.02485	2.40291

127.047	0.37554	30.04453	0.00132	30.04453	0.60279	30.04453	1.20826	30.0445	1.80056	30.04453	2.40243
127.147	0.37613	30.06422	9.79E-04	30.06422	0.60411	30.06422	1.2091	30.0642	1.80507	30.06422	2.39838
127.247	0.37659	30.08391	0.00148	30.08391	0.60042	30.08391	1.20546	30.0839	1.80345	30.08391	2.40342
127.347	0.37707	30.1036	8.11E-04	30.1036	0.6003	30.1036	1.20616	30.1036	1.80134	30.1036	2.40701
127.447	0.37738	30.12329	0.00741	30.12329	0.60111	30.12329	1.2083	30.1233	1.80447	30.12329	2.40623
127.547	0.37765	30.14298	0.00486	30.14298	0.60012	30.14298	1.20357	30.143	1.80335	30.14298	2.40521
127.647	0.37771	30.16267	0.00406	30.16267	0.605	30.16267	1.20653	30.1627	1.80073	30.16267	2.40662
127.747	0.37776	30.18236	0.00216	30.18236	0.6005	30.18236	1.20005	30.1824	1.80762	30.18236	2.40827
127.847	0.37799	30.20205	0	30.20205	0.60144	30.20205	1.20581	30.2021	1.8	30.20205	2.40967
127.947	0.37876	30.22174	0.00645	30.22174	0.60101	30.22174	1.20511	30.2217	1.80425	30.22174	2.40477
128.047	0.37933	30.24143	0.00616	30.24143	0.60627	30.24143	1.20384	30.2414	1.80494	30.24143	2.4046
128.147	0.37962	30.26112	0.00347	30.26112	0.60402	30.26112	1.20572	30.2611	1.80225	30.26112	2.40649
128.247	0.38024	30.2808	0.00636	30.2808	0.59595	30.2808	1.20007	30.2808	1.80431	30.2808	2.4079
128.347	0.38037	30.30049	0.00814	30.30049	0.60484	30.30049	1.20469	30.3005	1.80718	30.30049	2.41349
128.447	0.38076	30.32018	0.00545	30.32018	0.60322	30.32018	1.20476	30.3202	1.80293	30.32018	2.40429
128.547	0.38107	30.33987	0.00562	30.33987	0.60172	30.33987	1.20081	30.3399	1.804	30.33987	2.40563
128.647	0.38138	30.35956	0.0096	30.35956	0.60166	30.35956	1.20471	30.3596	1.80687	30.35956	2.40952
128.747	0.38185	30.37925	7.54E-04	30.37925	0.61024	30.37925	1.20039	30.3793	1.80674	30.37925	2.40608
128.847	0.38206	30.39894	0.00416	30.39894	0.61224	30.39894	1.20522	30.3989	1.80449	30.39894	2.40397
128.947	0.38247	30.41863	0.0097	30.41863	0.60393	30.41863	1.20504	30.4186	1.80399	30.41863	2.40913
129.047	0.38309	30.43832	0.00643	30.43832	0.60681	30.43832	1.19773	30.4383	1.80724	30.43832	2.40848
129.147	0.38348	30.45801	0.01081	30.45801	0.60757	30.45801	1.20348	30.458	1.80537	30.45801	2.40843
129.247	0.38436	30.4777	0.01032	30.4777	0.60532	30.4777	1.20155	30.4777	1.80443	30.4777	2.40651
129.347	0.38434	30.49739	0.01165	30.49739	0.60664	30.49739	1.20369	30.4974	1.80262	30.49739	2.40652
129.447	0.38414	30.51707	0.01739	30.51707	0.60714	30.51707	1.20278	30.5171	1.8118	30.51707	2.41053
129.547	0.38464	30.53676	0.01347	30.53676	0.60952	30.53676	1.20462	30.5368	1.80268	30.53676	2.40794
129.647	0.38556	30.55645	0.02148	30.55645	0.60527	30.55645	1.20428	30.5565	1.80505	30.55645	2.41165
129.747	0.38598	30.57614	0.0256	30.57614	0.61047	30.57614	1.20435	30.5761	1.80174	30.57614	2.41172
129.847	0.38614	30.59583	0.02596	30.59583	0.61254	30.59583	1.20335	30.5958	1.81117	30.59583	2.40307
129.947	0.38663	30.61552	0.02988	30.61552	0.61142	30.61552	1.20063	30.6155	1.80811	30.61552	2.40757
130.047	0.38712	30.63521	0.03478	30.63521	0.61624	30.63521	1.2035	30.6352	1.80605	30.63521	2.41267
130.147	0.38756	30.6549	0.04084	30.6549	0.61944	30.6549	1.20766	30.6549	1.80199	30.6549	2.41062
130.247	0.38753	30.67459	0.03867	30.67459	0.62702	30.67459	1.20556	30.6746	1.81017	30.67459	2.40779
130.347	0.38838	30.69427	0.0485	30.69427	0.62646	30.69427	1.20621	30.6943	1.80523	30.69427	2.40629
130.447	0.38833	30.71396	0.0506	30.71396	0.6341	30.71396	1.20582	30.714	1.81461	30.71396	2.41012
130.547	0.38861	30.73365	0.05822	30.73365	0.63648	30.73365	1.20853	30.7337	1.80673	30.73365	2.41195
130.647	0.38895	30.75334	0.06228	30.75334	0.63317	30.75334	1.20819	30.7533	1.80992	30.75334	2.41027
130.747	0.38946	30.77303	0.07035	30.77303	0.64851	30.77303	1.2107	30.773	1.81061	30.77303	2.41076
130.847	0.38989	30.79272	0.06721	30.79272	0.64031	30.79272	1.21181	30.7927	1.80479	30.79272	2.41242
130.947	0.39015	30.81241	0.06919	30.81241	0.64107	30.81241	1.20884	30.8124	1.80829	30.81241	2.4097
131.047	0.39061	30.8321	0.06222	30.8321	0.64527	30.8321	1.21016	30.8321	1.80067	30.8321	2.40584
131.147	0.39067	30.85179	0.0629	30.85179	0.64046	30.85179	1.20951	30.8518	1.80254	30.85179	2.40622
131.247	0.39102	30.87148	0.05729	30.87148	0.64159	30.87148	1.20943	30.8715	1.80342	30.87148	2.4095
131.347	0.39186	30.89117	0.04935	30.89117	0.63452	30.89117	1.21178	30.8912	1.80392	30.89117	2.41291

131.447	0.3922	30.91086	0.04562	30.91086	0.63472	30.91086	1.21186	30.9109	1.79998	30.91086	2.41038
131.547	0.39252	30.93054	0.03813	30.93054	0.62796	30.93054	1.21091	30.9305	1.80523	30.93054	2.40845
131.647	0.39297	30.95023	0.03739	30.95023	0.62609	30.95023	1.21124	30.9502	1.80773	30.95023	2.41029
131.747	0.3927	30.96992	0.02964	30.96992	0.6276	30.96992	1.21029	30.9699	1.80579	30.96992	2.40782
131.847	0.39302	30.98961	0.02286	30.98961	0.62254	30.98961	1.21197	30.9896	1.80141	30.98961	2.40959
131.947	0.39331	31.0093	0.02322	31.0093	0.61791	31.0093	1.21447	31.0093	1.80366	31.0093	2.4096
132.047	0.3935	31.02899	0.01424	31.02899	0.61717	31.02899	1.21006	31.029	1.80672	31.02899	2.41059
132.147	0.39382	31.04868	0.01641	31.04868	0.61185	31.04868	1.20931	31.0487	1.80391	31.04868	2.41066
132.247	0.39391	31.06837	0.01307	31.06837	0.61299	31.06837	1.21445	31.0684	1.79985	31.06837	2.40462
132.347	0.39405	31.08806	0.0085	31.08806	0.61568	31.08806	1.20838	31.0881	1.80266	31.08806	2.409
132.447	0.39424	31.10775	0.01392	31.10775	0.61068	31.10775	1.21233	31.1078	1.80497	31.10775	2.41392
132.547	0.3946	31.12744	0.01305	31.12744	0.60844	31.12744	1.20838	31.1274	1.80716	31.12744	2.40472
132.647	0.39511	31.14713	0.01386	31.14713	0.61182	31.14713	1.20975	31.1471	1.8081	31.14713	2.41274
132.747	0.39522	31.16681	0.01272	31.16681	0.60657	31.16681	1.20988	31.1668	1.80972	31.16681	2.41275
132.847	0.39533	31.1865	0.01094	31.1865	0.60464	31.1865	1.20681	31.1865	1.8066	31.1865	2.40562
132.947	0.39583	31.20619	0.01357	31.20619	0.6074	31.20619	1.20792	31.2062	1.81072	31.20619	2.40569
133.047	0.39606	31.22588	0.00887	31.22588	0.60916	31.22588	1.2048	31.2259	1.80828	31.22588	2.4105
133.147	0.39617	31.24557	0.01208	31.24557	0.60641	31.24557	1.20375	31.2456	1.81047	31.24557	2.41027
133.247	0.39615	31.26526	0.0077	31.26526	0.60648	31.26526	1.20744	31.2653	1.80947	31.26526	2.41313
133.347	0.39655	31.28495	0.01013	31.28495	0.60492	31.28495	1.21	31.285	1.80584	31.28495	2.40933
133.447	0.39666	31.30464	0.00693	31.30464	0.60399	31.30464	1.20935	31.3046	1.80366	31.30464	2.40929
133.547	0.39704	31.32433	0.01169	31.32433	0.60463	31.32433	1.20675	31.3243	1.8084	31.32433	2.40403
133.647	0.3973	31.34402	0.01212	31.34402	0.60307	31.34402	1.20838	31.344	1.8109	31.34402	2.4075
133.747	0.39765	31.3637	0.00994	31.3637	0.60489	31.3637	1.20815	31.3637	1.81228	31.3637	2.40624
133.847	0.39772	31.38339	0.00803	31.38339	0.60252	31.38339	1.21034	31.3834	1.80634	31.38339	2.40729
133.947	0.39832	31.40308	0.00722	31.40308	0.60021	31.40308	1.20727	31.4031	1.80153	31.40308	2.40555
134.047	0.39884	31.42277	0.00563	31.42277	0.60328	31.42277	1.20761	31.4228	1.8074	31.42277	2.4055
134.147	0.39908	31.44246	0.00268	31.44246	0.60417	31.44246	1.208	31.4425	1.80246	31.44246	2.40794
134.247	0.39919	31.46215	5.93E-05	31.46215	0.60211	31.46215	1.20958	31.4622	1.80096	31.46215	2.40311
134.347	0.39964	31.48184	0.00145	31.48184	0.60199	31.48184	1.21363	31.4818	1.80465	31.48184	2.40349
134.447	0.4001	31.50153	0.00369	31.50153	0.60043	31.50153	1.20695	31.5015	1.8059	31.50153	2.40314
134.547	0.40024	31.52122	0.00314	31.52122	0.60307	31.52122	1.21297	31.5212	1.80596	31.52122	2.40886
134.647	0.4007	31.54091	0.00395	31.54091	0.60007	31.54091	1.20675	31.5409	1.8079	31.54091	2.40251
134.747	0.40059	31.5606	6.80E-04	31.5606	0.6014	31.5606	1.21158	31.5606	1.80071	31.5606	2.4058
134.847	0.40119	31.58028	0	31.58028	0.60247	31.58028	1.21037	31.5803	1.80046	31.58028	2.40139
134.947	0.40173	31.59997	0.00395	31.59997	0.60629	31.59997	1.21334	31.6	1.80102	31.59997	2.40462
135.047	0.40213	31.61966	0.00491	31.61966	0.60192	31.61966	1.20124	31.6197	1.80202	31.61966	2.40645
135.147	0.40278	31.63935	0.0014	31.63935	0.60143	31.63935	1.20999	31.6394	1.80402	31.63935	2.40241
135.247	0.40323	31.65904	0.00573	31.65904	0.60112	31.65904	1.2047	31.659	1.80427	31.65904	2.40637
135.347	0.40325	31.67873	4.70E-04	31.67873	0.60288	31.67873	1.20463	31.6787	1.79996	31.67873	2.40274
135.447	0.40349	31.69842	0.00325	31.69842	0.59838	31.69842	1.20089	31.6984	1.80496	31.69842	2.40155
135.547	0.40369	31.71811	-0.00175	31.71811	0.59752	31.71811	1.2052	31.7181	1.80102	31.71811	2.40647
135.647	0.40394	31.7378	0.00284	31.7378	0.60541	31.7378	1.20389	31.7378	1.80533	31.7378	2.40388
135.747	0.40411	31.75749	0.00808	31.75749	0.59878	31.75749	1.2065	31.7575	1.80083	31.75749	2.40208

135.847	0.40467	31.77718	0.00606	31.77718	0.60298	31.77718	1.20586	31.7772	1.80058	31.77718	2.40495
135.947	0.40476	31.79687	0.00664	31.79687	0.60362	31.79687	1.20264	31.7969	1.80083	31.79687	2.40424
136.047	0.40498	31.81656	0.0076	31.81656	0.59962	31.81656	1.20303	31.8166	1.80389	31.81656	2.40632
136.147	0.40531	31.83624	0.00175	31.83624	0.60188	31.83624	1.20275	31.8362	1.80027	31.83624	2.40233
136.247	0.40556	31.85593	9.01E-04	31.85593	0.60195	31.85593	1.2034	31.8559	1.80351	31.85593	2.40489
136.347	0.40552	31.87562	0.00154	31.87562	0.59983	31.87562	1.20374	31.8756	1.80701	31.87562	2.40661
136.447	0.40595	31.89531	0.00425	31.89531	0.5984	31.89531	1.20335	31.8953	1.79858	31.89531	2.40444
136.547	0.40617	31.915	8.72E-04	31.915	0.6001	31.915	1.19946	31.915	1.80383	31.915	2.40749
136.647	0.40658	31.93469	-3.05E-04	31.93469	0.59811	31.93469	1.20238	31.9347	1.80051	31.93469	2.40351
136.747	0.4066	31.95438	0.00533	31.95438	0.60268	31.95438	1.19864	31.9544	1.80626	31.95438	2.4091
136.847	0.40663	31.97407	0.00473	31.97407	0.60401	31.97407	1.20136	31.9741	1.80551	31.97407	2.40451
136.947	0.40673	31.99376	3.79E-04	31.99376	0.60796	31.99376	1.20113	31.9938	1.80445	31.99376	2.40798
137.047	0.4066	32.01345	0.00394	32.01345	0.6019	32.01345	1.19899	32.0135	1.8037	32.01345	2.40915
137.147	0.40654	32.03313	0.00308	32.03313	0.60147	32.03313	1.20207	32.0331	1.80288	32.03313	2.40826
137.247	0.40615	32.05282	0.00567	32.05282	0.60648	32.05282	1.20205	32.0528	1.8027	32.05282	2.40488
137.347	0.40629	32.07251	0.00994	32.07251	0.60668	32.07251	1.2028	32.0725	1.80207	32.07251	2.40793
137.447	0.40652	32.0922	0.00571	32.0922	0.60212	32.0922	1.20103	32.0922	1.80332	32.0922	2.40879
137.547	0.40713	32.11189	0.00577	32.11189	0.60463	32.11189	1.19987	32.1119	1.80463	32.11189	2.40681
137.647	0.40743	32.13158	0.01043	32.13158	0.6057	32.13158	1.20315	32.1316	1.80394	32.13158	2.40531
137.747	0.40695	32.15127	0.00529	32.15127	0.60966	32.15127	1.20659	32.1513	1.80894	32.15127	2.40345
137.847	0.40677	32.17096	0.00963	32.17096	0.6071	32.17096	1.20203	32.171	1.80394	32.17096	2.40335
137.947	0.40654	32.19065	0.01001	32.19065	0.60855	32.19065	1.20495	32.1907	1.80519	32.19065	2.4033
138.047	0.40682	32.21033	0.01169	32.21033	0.61075	32.21033	1.20297	32.2103	1.80238	32.21033	2.40853
138.147	0.40687	32.23003	0.01012	32.23003	0.608	32.23003	1.20522	32.23	1.80057	32.23003	2.40582
138.247	0.40626	32.24971	0.0105	32.24971	0.6112	32.24971	1.20758	32.2497	1.80088	32.24971	2.40651
138.347	0.40597	32.26941	0.00861	32.26941	0.61284	32.26941	1.20213	32.2694	1.80394	32.26941	2.40349
138.447	0.40591	32.28909	0.01197	32.28909	0.60878	32.28909	1.20624	32.2891	1.79594	32.28909	2.40539
138.547	0.4059	32.30878	0.01248	32.30878	0.61273	32.30878	1.20767	32.3088	1.80275	32.30878	2.40698
138.647	0.40557	32.32847	0.01694	32.32847	0.6108	32.32847	1.20941	32.3285	1.80194	32.32847	2.40651
138.747	0.40533	32.34816	0.01039	32.34816	0.6135	32.34816	1.21181	32.3482	1.79875	32.34816	2.40368
138.847	0.40526	32.36785	0.01511	32.36785	0.61439	32.36785	1.20766	32.3679	1.8	32.36785	2.4
138.947	0.40455	32.38754	0.01795	32.38754	0.61571	32.38754	1.21007	32.3875	1.80158	32.38754	2.40591
139.047	0.40441	32.40723	0.01749	32.40723	0.6109	32.40723	1.20948	32.4072	1.79853	32.40723	2.40503
139.147	0.40411	32.42692	0.01683	32.42692	0.62086	32.42692	1.21117	32.4269	1.79673	32.42692	2.40554
139.247	0.40355	32.44661	0.02026	32.44661	0.61862	32.44661	1.21042	32.4466	1.80256	32.44661	2.4043
139.347	0.4032	32.46629	0.01694	32.46629	0.61894	32.46629	1.21242	32.4663	1.79864	32.46629	2.40615
139.447	0.40272	32.48598	0.02264	32.48598	0.61601	32.48598	1.20806	32.486	1.80203	32.48598	2.40709
139.547	0.40204	32.50567	0.01893	32.50567	0.61608	32.50567	1.20866	32.5057	1.80279	32.50567	2.40239
139.647	0.4017	32.52536	0.02398	32.52536	0.62066	32.52536	1.20652	32.5254	1.80431	32.52536	2.40181
139.747	0.4015	32.54505	0.02209	32.54505	0.61059	32.54505	1.21265	32.5451	1.7967	32.54505	2.4056
139.847	0.40142	32.56474	0.02279	32.56474	0.60872	32.56474	1.2102	32.5647	1.80046	32.56474	2.4063
139.947	0.40036	32.58443	0.02486	32.58443	0.61574	32.58443	1.21148	32.5844	1.8016	32.58443	2.40384
140.047	0.40035	32.60412	0.01895	32.60412	0.60943	32.60412	1.20815	32.6041	1.80149	32.60412	2.41005
140.147	0.40013	32.62381	0.02186	32.62381	0.61307	32.62381	1.20989	32.6238	1.80082	32.62381	2.40433

140.247	0.39973	32.6435	0.01847	32.6435	0.61615	32.6435	1.21168	32.6435	1.79815	32.6435	2.40714
140.347	0.39932	32.66319	0.01983	32.66319	0.60883	32.66319	1.20639	32.6632	1.80173	32.66319	2.40396
140.447	0.39867	32.68287	0.02098	32.68287	0.60465	32.68287	1.20885	32.6829	1.80336	32.68287	2.40654
140.547	0.39827	32.70256	0.01786	32.70256	0.60841	32.70256	1.2048	32.7026	1.80113	32.70256	2.40917
140.647	0.39794	32.72225	0.01539	32.72225	0.60855	32.72225	1.20995	32.7223	1.80402	32.72225	2.40429
140.747	0.39729	32.74194	0.02108	32.74194	0.60731	32.74194	1.2057	32.7419	1.8026	32.74194	2.40553
140.847	0.39681	32.76163	0.01738	32.76163	0.60394	32.76163	1.20542	32.7616	1.80274	32.76163	2.40708
140.947	0.39634	32.78132	0.0162	32.78132	0.60363	32.78132	1.20731	32.7813	1.80675	32.78132	2.4065
141.047	0.39624	32.80101	0.01224	32.80101	0.60915	32.80101	1.20497	32.801	1.80333	32.80101	2.40756
141.147	0.3951	32.8207	0.00795	32.8207	0.60735	32.8207	1.20402	32.8207	1.80297	32.8207	2.40329
141.247	0.39486	32.84039	0.01157	32.84039	0.60072	32.84039	1.20369	32.8404	1.80242	32.84039	2.40253
141.347	0.39423	32.86008	0.01195	32.86008	0.60743	32.86008	1.20151	32.8601	1.80819	32.86008	2.40838
141.447	0.39382	32.87976	0.00785	32.87976	0.60224	32.87976	1.19658	32.8798	1.80764	32.87976	2.40598
141.547	0.39316	32.89946	0.00719	32.89946	0.601	32.89946	1.2008	32.8995	1.80428	32.89946	2.40365
141.647	0.3921	32.91914	0.0053	32.91914	0.60408	32.91914	1.20372	32.9191	1.80136	32.91914	2.40447
141.747	0.39172	32.93883	0.00361	32.93883	0.59996	32.93883	1.19596	32.9388	1.80881	32.93883	2.40056
141.847	0.3915	32.95852	0.00587	32.95852	0.6031	32.95852	1.20043	32.9585	1.80351	32.95852	2.4001
141.947	0.39133	32.97821	0.00579	32.97821	0.60116	32.97821	1.20444	32.9782	1.79997	32.97821	2.40413
142.047	0.39042	32.9979	0.0041	32.9979	0.60274	32.9979	1.20251	32.9979	1.79842	32.9979	2.40659
142.147	0.38983	33.01759	0.00396	33.01759	0.60419	33.01759	1.19759	33.0176	1.80812	33.01759	2.40371
142.247	0.38929	33.03728	0.00265	33.03728	0.59919	33.03728	1.20289	33.0373	1.8007	33.03728	2.40465
142.347	0.38815	33.05697	0.0066	33.05697	0.60133	33.05697	1.20184	33.057	1.80634	33.05697	2.4068
142.447	0.38761	33.07666	0.00425	33.07666	0.60465	33.07666	1.20033	33.0767	1.80123	33.07666	2.40471
142.547	0.38694	33.09634	0.00373	33.09634	0.6046	33.09634	1.19943	33.0963	1.79731	33.09634	2.40371
142.647	0.38604	33.11604	6.68E-04	33.11604	0.60361	33.11604	1.20251	33.116	1.80163	33.11604	2.40095
142.747	0.38448	33.13572	0.0015	33.13572	0.60569	33.13572	1.19619	33.1357	1.79684	33.13572	2.4037
142.847	0.38416	33.15541	0	33.15541	0.6	33.15541	1.20062	33.1554	1.8026	33.15541	2.40337
142.947	0.38347	33.1751	0.00586	33.1751	0.60315	33.1751	1.20101	33.1751	1.80193	33.1751	2.40734
143.047	0.38274	33.19479	0.00289	33.19479	0.6058	33.19479	1.19903	33.1948	1.80625	33.19479	2.40246
143.147	0.38171	33.21448	-9.12E-04	33.21448	0.60244	33.21448	1.20382	33.2145	1.80289	33.21448	2.40007
143.247	0.3807	33.23417	0.00319	33.23417	0.60359	33.23417	1.1975	33.2342	1.80066	33.23417	2.40506
143.347	0.38037	33.25386	-0.0023	33.25386	0.6073	33.25386	1.20228	33.2539	1.80036	33.25386	2.4017
143.447	0.38015	33.27355	-4.62E-04	33.27355	0.60738	33.27355	1.20196	33.2736	1.80213	33.27355	2.40361
143.547	0.37932	33.29324	0.00144	33.29324	0.60928	33.29324	1.20173	33.2932	1.79902	33.29324	2.40097
143.647	0.37832	33.31293	0.00477	33.31293	0.6053	33.31293	1.2012	33.3129	1.80184	33.31293	2.40415
143.747	0.37729	33.33261	0.00738	33.33261	0.61051	33.33261	1.20201	33.3326	1.79905	33.33261	2.40673
143.847	0.37568	33.3523	0.0061	33.3523	0.60215	33.3523	1.20183	33.3523	1.8	33.3523	2.40118
143.947	0.37506	33.37199	0.00898	33.37199	0.60636	33.37199	1.20084	33.372	1.79803	33.37199	2.40248
144.047	0.37383	33.39168	0.00342	33.39168	0.61202	33.39168	1.20459	33.3917	1.80286	33.39168	2.4027
144.147	0.37261	33.41137	0.00649	33.41137	0.61054	33.41137	1.19977	33.4114	1.7997	33.41137	2.40648
144.247	0.37244	33.43106	0.00372	33.43106	0.61544	33.43106	1.20089	33.4311	1.80347	33.43106	2.40336
144.347	0.37188	33.45075	0.00459	33.45075	0.61014	33.45075	1.20051	33.4508	1.80456	33.45075	2.40818
144.447	0.37052	33.47044	0.00545	33.47044	0.60866	33.47044	1.20225	33.4704	1.80552	33.47044	2.40227
144.547	0.36976	33.49013	0.00982	33.49013	0.61094	33.49013	1.19738	33.4901	1.79905	33.49013	2.4106

144.647	0.36887	33.50982	0.00932	33.50982	0.61359	33.50982	1.19984	33.5098	1.80514	33.50982	2.41124
144.747	0.36715	33.52951	0.01116	33.52951	0.60998	33.52951	1.20349	33.5295	1.80472	33.52951	2.4129
144.847	0.36595	33.54919	0.01286	33.54919	0.60718	33.54919	1.20006	33.5492	1.8015	33.54919	2.41269
144.947	0.36506	33.56889	0.00984	33.56889	0.61365	33.56889	1.20325	33.5689	1.80559	33.56889	2.41024
145.047	0.36375	33.58857	0.0142	33.58857	0.61004	33.58857	1.20039	33.5886	1.80417	33.58857	2.40899
145.147	0.36188	33.60826	0.01332	33.60826	0.60919	33.60826	1.20073	33.6083	1.80632	33.60826	2.40805
145.247	0.36096	33.62795	0.01645	33.62795	0.60189	33.62795	1.20392	33.628	1.80829	33.62795	2.41457
145.347	0.36013	33.64764	0.01764	33.64764	0.60848	33.64764	1.20349	33.6476	1.80069	33.64764	2.41381
145.447	0.35883	33.66733	0.01338	33.66733	0.60794	33.66733	1.20378	33.6673	1.81009	33.66733	2.41269
145.547	0.35724	33.68702	0.01574	33.68702	0.60972	33.68702	1.19999	33.687	1.80861	33.68702	2.41102
145.647	0.3557	33.70671	0.01213	33.70671	0.60962	33.70671	1.20338	33.7067	1.80976	33.70671	2.41063
145.747	0.35423	33.7264	0.00878	33.7264	0.60839	33.7264	1.20275	33.7264	1.80941	33.7264	2.41205
145.847	0.35352	33.74609	0.00971	33.74609	0.60847	33.74609	1.2048	33.7461	1.80625	33.74609	2.40923
145.947	0.35306	33.76577	0.00688	33.76577	0.60856	33.76577	1.20509	33.7658	1.81059	33.76577	2.41059
146.047	0.35106	33.78547	0.00866	33.78547	0.6052	33.78547	1.20497	33.7855	1.8088	33.78547	2.40759
146.147	0.34945	33.80515	0.00453	33.80515	0.60911	33.80515	1.20676	33.8052	1.81145	33.80515	2.40859
146.247	0.3482	33.82484	0.00695	33.82484	0.61357	33.82484	1.20581	33.8248	1.80597	33.82484	2.40899
146.347	0.34681	33.84453	0.00892	33.84453	0.60972	33.84453	1.20156	33.8445	1.80787	33.84453	2.40665
146.447	0.34507	33.86422	0.00687	33.86422	0.60799	33.86422	1.20754	33.8642	1.80696	33.86422	2.41201
146.547	0.34333	33.88391	0.00572	33.88391	0.60995	33.88391	1.20447	33.8839	1.80799	33.88391	2.40465
146.647	0.34187	33.9036	0.00348	33.9036	0.61342	33.9036	1.20895	33.9036	1.80795	33.9036	2.40995
146.747	0.34025	33.92329	0.00817	33.92329	0.60719	33.92329	1.208	33.9233	1.8056	33.92329	2.41053
146.847	0.34003	33.94298	0.00495	33.94298	0.61115	33.94298	1.20829	33.943	1.812	33.94298	2.40728
146.947	0.33678	33.96267	0.00588	33.96267	0.61431	33.96267	1.21045	33.9627	1.81021	33.96267	2.41004
147.047	0.33572	33.98236	0.00591	33.98236	0.60682	33.98236	1.20857	33.9824	1.80586	33.98236	2.40619
147.147	0.33498	34.00204	0.00353	34.00204	0.6081	34.00204	1.20334	34.002	1.81245	34.00204	2.40737
147.247	0.33227	34.02173	0.00531	34.02173	0.60756	34.02173	1.20916	34.0217	1.81116	34.02173	2.40662
147.347	0.33018	34.04142	0.00909	34.04142	0.60634	34.04142	1.2063	34.0414	1.81125	34.04142	2.4098
147.447	0.32926	34.06111	0.01074	34.06111	0.60523	34.06111	1.20427	34.0611	1.8074	34.06111	2.41298
147.547	0.32679	34.0808	0.01057	34.0808	0.60707	34.0808	1.20901	34.0808	1.80599	34.0808	2.41101
147.647	0.32556	34.10049	0.00586	34.10049	0.60873	34.10049	1.20847	34.1005	1.80714	34.10049	2.40776
147.747	0.3239	34.12018	0.00394	34.12018	0.606	34.12018	1.20525	34.1202	1.80629	34.12018	2.40816
147.847	0.32223	34.13987	0.00312	34.13987	0.61034	34.13987	1.20751	34.1399	1.81112	34.13987	2.41116
147.947	0.32034	34.15956	0.00263	34.15956	0.60737	34.15956	1.20439	34.1596	1.80746	34.15956	2.40658
148.047	0.3188	34.17924	0.00583	34.17924	0.6089	34.17924	1.20174	34.1792	1.80799	34.17924	2.40661
148.147	0.31713	34.19894	0.0056	34.19894	0.60755	34.19894	1.2025	34.1989	1.81007	34.19894	2.40652
148.247	0.31475	34.21862	0.00919	34.21862	0.60463	34.21862	1.20269	34.2186	1.80972	34.21862	2.40419
148.347	0.3128	34.23831	-0.00109	34.23831	0.6046	34.23831	1.20433	34.2383	1.80556	34.23831	2.40943
148.447	0.31041	34.258	0.00477	34.258	0.60462	34.258	1.20529	34.258	1.80815	34.258	2.40855
148.547	0.30853	34.27769	0.00882	34.27769	0.60396	34.27769	1.20362	34.2777	1.80536	34.27769	2.40397
148.647	0.30776	34.29738	0.00392	34.29738	0.60023	34.29738	1.20128	34.2974	1.80476	34.29738	2.40582
148.747	0.30624	34.31707	9.60E-04	34.31707	0.60614	34.31707	1.20235	34.3171	1.79929	34.31707	2.40403
148.847	0.30393	34.33676	0.00611	34.33676	0.60586	34.33676	1.2012	34.3368	1.8	34.33676	2.4
148.947	0.30175	34.35645	0.00523	34.35645	0.61089	34.35645	1.20655	34.3565	1.80345	34.35645	2.40122

149.047	0.29986	34.37614	-0.00298	34.37614	0.60636	34.37614	1.20318	34.3761	1.80572	34.37614	2.40239
149.147	0.29826	34.39582	0.00159	34.39582	0.6015	34.39582	1.20487	34.3958	1.80136	34.39582	2.39888
149.247	0.29616	34.41552	0.00214	34.41552	0.60785	34.41552	1.20764	34.4155	1.80163	34.41552	2.40416
149.347	0.29546	34.4352	2.85E-04	34.4352	0.60269	34.4352	1.20385	34.4352	1.80265	34.4352	2.39817
149.447	0.29379	34.4549	0.0031	34.4549	0.60434	34.4549	1.2057	34.4549	1.80372	34.4549	2.40291
149.547	0.29107	34.47458	0.00248	34.47458	0.59924	34.47458	1.2016	34.4746	1.80105	34.47458	2.40201
149.647	0.28903	34.49427	0.00329	34.49427	0.60666	34.49427	1.20112	34.4943	1.80588	34.49427	2.4019
149.747	0.28715	34.51396	6.58E-04	34.51396	0.60118	34.51396	1.20766	34.514	1.80284	34.51396	2.40306
149.847	0.28554	34.53365	0.00315	34.53365	0.60446	34.53365	1.2	34.5337	1.80235	34.53365	2.40368
149.947	0.28337	34.55334	0.00376	34.55334	0.60743	34.55334	1.20547	34.5533	1.80037	34.55334	2.40581
150.047	0.28177	34.57303	0.00341	34.57303	0.60546	34.57303	1.20288	34.573	1.80126	34.57303	2.39843
150.147	0.27988	34.59272	-0.00149	34.59272	0.60337	34.59272	1.20722	34.5927	1.79922	34.59272	2.40832
150.247	0.27785	34.61241	8.73E-04	34.61241	0.61053	34.61241	1.202	34.6124	1.79549	34.61241	2.40675
150.347	0.27611	34.6321	0.00428	34.6321	0.60699	34.6321	1.19828	34.6321	1.802	34.6321	2.40143
150.447	0.27471	34.65178	-3.63E-04	34.65178	0.60546	34.65178	1.20184	34.6518	1.79589	34.65178	2.40477
150.547	0.27381	34.67147	0.00297	34.67147	0.60349	34.67147	1.20504	34.6715	1.80235	34.67147	2.40769
150.647	0.27251	34.69116	0.00411	34.69116	0.60534	34.69116	1.20617	34.6912	1.80143	34.69116	2.40316
150.747	0.27059	34.71085	0.00116	34.71085	0.6018	34.71085	1.20513	34.7109	1.79894	34.71085	2.40565
150.847	0.26955	34.73054	0.00702	34.73054	0.60333	34.73054	1.20601	34.7305	1.80296	34.73054	2.40385
150.947	0.26767	34.75023	0.00491	34.75023	0.61019	34.75023	1.20399	34.7502	1.80254	34.75023	2.40398
151.047	0.2659	34.76992	0.0032	34.76992	0.60741	34.76992	1.20285	34.7699	1.79624	34.76992	2.4029
151.147	0.26415	34.78961	0.00868	34.78961	0.61207	34.78961	1.20042	34.7896	1.80101	34.78961	2.40261
151.247	0.2624	34.8093	0.00417	34.8093	0.61229	34.8093	1.20537	34.8093	1.80265	34.8093	2.40474
151.347	0.26092	34.82899	0.00226	34.82899	0.61195	34.82899	1.20811	34.829	1.80011	34.82899	2.3979
151.447	0.26007	34.84867	0.00501	34.84867	0.61211	34.84867	1.20826	34.8487	1.80219	34.84867	2.4021
151.547	0.25844	34.86837	0.00868	34.86837	0.61333	34.86837	1.20712	34.8684	1.8012	34.86837	2.39798
151.647	0.2571	34.88805	0.0054	34.88805	0.6148	34.88805	1.2068	34.8881	1.80509	34.88805	2.40569
151.747	0.25554	34.90774	0.00667	34.90774	0.61246	34.90774	1.20618	34.9077	1.80705	34.90774	2.40395
151.847	0.2541	34.92743	0.00968	34.92743	0.6123	34.92743	1.20432	34.9274	1.80688	34.92743	2.40523
151.947	0.25244	34.94712	0.01361	34.94712	0.61234	34.94712	1.20251	34.9471	1.80065	34.94712	2.40397
152.047	0.25113	34.96681	0.01163	34.96681	0.61313	34.96681	1.20493	34.9668	1.8026	34.96681	2.40743
152.147	0.24974	34.9865	0.01997	34.9865	0.61679	34.9865	1.2055	34.9865	1.80087	34.9865	2.40787
152.247	0.24845	35.00619	0.01372	35.00619	0.61144	35.00619	1.20896	35.0062	1.7987	35.00619	2.40509
152.347	0.24719	35.02588	0.01155	35.02588	0.61154	35.02588	1.20885	35.0259	1.80153	35.02588	2.40735
152.447	0.24668	35.04557	0.01074	35.04557	0.60989	35.04557	1.20859	35.0456	1.80998	35.04557	2.4053
152.547	0.2451	35.06525	0.01667	35.06525	0.60924	35.06525	1.20688	35.0653	1.80738	35.06525	2.40482
152.647	0.24332	35.08495	0.01671	35.08495	0.60921	35.08495	1.20946	35.085	1.80264	35.08495	2.40447
152.747	0.24247	35.10463	0.01363	35.10463	0.61006	35.10463	1.20501	35.1046	1.80641	35.10463	2.40624
152.847	0.24192	35.12432	0.01801	35.12432	0.60728	35.12432	1.20465	35.1243	1.80586	35.12432	2.40698
152.947	0.23958	35.14401	0.01364	35.14401	0.60925	35.14401	1.20615	35.144	1.80619	35.14401	2.40469
153.047	0.23869	35.1637	0.01685	35.1637	0.60998	35.1637	1.21198	35.1637	1.80652	35.1637	2.41137
153.147	0.23739	35.18339	0.01877	35.18339	0.60952	35.18339	1.20924	35.1834	1.80667	35.18339	2.40544
153.247	0.23553	35.20308	0.01641	35.20308	0.61012	35.20308	1.21156	35.2031	1.80937	35.20308	2.40654
153.347	0.23439	35.22277	0.01457	35.22277	0.60715	35.22277	1.20531	35.2228	1.80289	35.22277	2.40558

153.447	0.23341	35.24246	0.01454	35.24246	0.60844	35.24246	1.20655	35.2425	1.80653	35.24246	2.40772
153.547	0.23243	35.26215	0.01957	35.26215	0.60936	35.26215	1.2037	35.2622	1.80299	35.26215	2.40652
153.647	0.23157	35.28184	0.01364	35.28184	0.61033	35.28184	1.2067	35.2818	1.80544	35.28184	2.41065
153.747	0.23054	35.30153	0.01452	35.30153	0.60618	35.30153	1.20592	35.3015	1.80565	35.30153	2.40872
153.847	0.22913	35.32121	0.01728	35.32121	0.60953	35.32121	1.20664	35.3212	1.80498	35.32121	2.41001
153.947	0.22845	35.3409	0.01434	35.3409	0.61264	35.3409	1.20488	35.3409	1.80044	35.3409	2.4065
154.047	0.22742	35.36059	0.01315	35.36059	0.61024	35.36059	1.20793	35.3606	1.80333	35.36059	2.40888
154.147	0.22621	35.38028	0.00943	35.38028	0.61178	35.38028	1.20927	35.3803	1.80753	35.38028	2.4075
154.247	0.22498	35.39997	0.01343	35.39997	0.60844	35.39997	1.20689	35.4	1.80293	35.39997	2.40757
154.347	0.22358	35.41966	0.00886	35.41966	0.61843	35.41966	1.20565	35.4197	1.80201	35.41966	2.40485
154.447	0.22233	35.43935	0.01273	35.43935	0.6072	35.43935	1.20627	35.4394	1.80184	35.43935	2.4082
154.547	0.22129	35.45904	0.01309	35.45904	0.60781	35.45904	1.20802	35.459	1.79761	35.45904	2.40791
154.647	0.2201	35.47873	0.00684	35.47873	0.60916	35.47873	1.20673	35.4787	1.80694	35.47873	2.40816
154.747	0.21856	35.49842	0.0076	35.49842	0.6087	35.49842	1.20647	35.4984	1.80389	35.49842	2.41175
154.847	0.21857	35.5181	0.00744	35.5181	0.6088	35.5181	1.20517	35.5181	1.8041	35.5181	2.41007
154.947	0.21774	35.53779	0.00826	35.53779	0.60991	35.53779	1.20559	35.5378	1.80424	35.53779	2.40117
155.047	0.21662	35.55748	0.00603	35.55748	0.61264	35.55748	1.20548	35.5575	1.80132	35.55748	2.40645
155.147	0.21579	35.57717	0.00582	35.57717	0.60724	35.57717	1.20398	35.5772	1.80247	35.57717	2.40168
155.247	0.21439	35.59686	0.00541	35.59686	0.60703	35.59686	1.20439	35.5969	1.80555	35.59686	2.40588
155.347	0.21327	35.61655	0.00623	35.61655	0.60895	35.61655	1.2077	35.6166	1.80344	35.61655	2.40577
155.447	0.21243	35.63624	0.00685	35.63624	0.61156	35.63624	1.21095	35.6362	1.80271	35.63624	2.40748
155.547	0.21145	35.65593	0.0104	35.65593	0.60884	35.65593	1.20486	35.6559	1.8026	35.65593	2.40652
155.647	0.21104	35.67562	0.00973	35.67562	0.60789	35.67562	1.20785	35.6756	1.80868	35.67562	2.4032
155.747	0.2095	35.69531	0.0064	35.69531	0.60905	35.69531	1.2063	35.6953	1.80176	35.69531	2.40812
155.847	0.20957	35.715	0.00689	35.715	0.61266	35.715	1.21027	35.715	1.80072	35.715	2.40177
155.947	0.20845	35.73468	0.00234	35.73468	0.60582	35.73468	1.20728	35.7347	1.80642	35.73468	2.40766
156.047	0.20783	35.75438	0.00653	35.75438	0.60643	35.75438	1.20846	35.7544	1.79988	35.75438	2.4084
156.147	0.20748	35.77406	0.00606	35.77406	0.60672	35.77406	1.20846	35.7741	1.80353	35.77406	2.40345
156.247	0.20572	35.79375	0.0074	35.79375	0.60658	35.79375	1.208	35.7938	1.80361	35.79375	2.40522
156.347	0.20495	35.81344	0.00991	35.81344	0.60737	35.81344	1.20789	35.8134	1.80413	35.81344	2.40784
156.447	0.20419	35.83313	0.00451	35.83313	0.60923	35.83313	1.20433	35.8331	1.80121	35.83313	2.40591
156.547	0.2037	35.85282	0.00741	35.85282	0.61209	35.85282	1.20614	35.8528	1.80773	35.85282	2.40459
156.647	0.20328	35.87251	0.00706	35.87251	0.61057	35.87251	1.20882	35.8725	1.80556	35.87251	2.40806
156.747	0.20245	35.8922	0.00659	35.8922	0.61105	35.8922	1.20836	35.8922	1.80408	35.8922	2.40583
156.847	0.20162	35.91189	0.00605	35.91189	0.61222	35.91189	1.20949	35.9119	1.80766	35.91189	2.40178
156.947	0.20119	35.93158	0.00577	35.93158	0.61214	35.93158	1.21042	35.9316	1.8083	35.93158	2.40289
157.047	0.20026	35.95126	0.00919	35.95126	0.60956	35.95126	1.21151	35.9513	1.80932	35.95126	2.40963
157.147	0.19964	35.97095	0.01443	35.97095	0.61092	35.97095	1.21264	35.971	1.80828	35.97095	2.40261
157.247	0.19889	35.99064	0.0126	35.99064	0.61146	35.99064	1.20732	35.9906	1.80667	35.99064	2.41141
157.347	0.19847	36.01033	0.01141	36.01033	0.61076	36.01033	1.20691	36.0103	1.80419	36.01033	2.40973
157.447	0.19792	36.03002	0.00938	36.03002	0.61093	36.03002	1.20799	36.03	1.81096	36.03002	2.4038
157.547	0.1974	36.04971	0.0093	36.04971	0.61748	36.04971	1.20737	36.0497	1.80636	36.04971	2.41127
157.647	0.19707	36.0694	0.01013	36.0694	0.61196	36.0694	1.20753	36.0694	1.80913	36.0694	2.40814
157.747	0.19622	36.08909	0.00881	36.08909	0.61613	36.08909	1.2098	36.0891	1.80827	36.08909	2.40785

157.847	0.19574	36.10878	0.00718	36.10878	0.61367	36.10878	1.20944	36.1088	1.80898	36.10878	2.41016
157.947	0.19579	36.12847	0.00787	36.12847	0.61629	36.12847	1.20954	36.1285	1.80869	36.12847	2.4069
158.047	0.19548	36.14816	0.00799	36.14816	0.61277	36.14816	1.20887	36.1482	1.80702	36.14816	2.41007
158.147	0.19528	36.16785	0.00856	36.16785	0.61375	36.16785	1.20707	36.1679	1.8061	36.16785	2.40621
158.247	0.19516	36.18753	0.00854	36.18753	0.61273	36.18753	1.21182	36.1875	1.80725	36.18753	2.4081
158.347	0.19448	36.20722	0.00697	36.20722	0.60909	36.20722	1.20707	36.2072	1.80683	36.20722	2.40557
158.447	0.19395	36.22691	0.0067	36.22691	0.61308	36.22691	1.20955	36.2269	1.80341	36.22691	2.40819
158.547	0.19364	36.2466	0.00163	36.2466	0.60918	36.2466	1.20356	36.2466	1.80374	36.2466	2.40439
158.647	0.19346	36.26629	0.00641	36.26629	0.61129	36.26629	1.21177	36.2663	1.7982	36.26629	2.40573
158.747	0.19327	36.28598	0.0084	36.28598	0.61028	36.28598	1.20614	36.286	1.80416	36.28598	2.40357
158.847	0.19284	36.30567	0.00748	36.30567	0.61032	36.30567	1.20676	36.3057	1.80205	36.30567	2.40091
158.947	0.19241	36.32536	0.00494	36.32536	0.60743	36.32536	1.20841	36.3254	1.80364	36.32536	2.40305
159.047	0.19221	36.34505	0.00778	36.34505	0.61123	36.34505	1.20991	36.3451	1.80078	36.34505	2.40391
159.147	0.19192	36.36473	-7.76E-05	36.36473	0.61791	36.36473	1.20521	36.3647	1.80224	36.36473	2.40369
159.247	0.19168	36.38443	0.01191	36.38443	0.60814	36.38443	1.20899	36.3844	1.80376	36.38443	2.40194
159.347	0.19147	36.40411	0.01092	36.40411	0.60893	36.40411	1.20677	36.4041	1.80772	36.40411	2.40238
159.447	0.19077	36.42381	0.00514	36.42381	0.60767	36.42381	1.21038	36.4238	1.79999	36.42381	2.39967
159.547	0.19076	36.44349	7.16E-04	36.44349	0.61034	36.44349	1.20641	36.4435	1.80395	36.44349	2.40114
159.647	0.19024	36.46318	0.00472	36.46318	0.6047	36.46318	1.20971	36.4632	1.80309	36.46318	2.40546
159.747	0.19027	36.48287	0.00523	36.48287	0.60931	36.48287	1.20641	36.4829	1.79811	36.48287	2.40372
159.847	0.18995	36.50256	0.0058	36.50256	0.61074	36.50256	1.20631	36.5026	1.80413	36.50256	2.40088
159.947	0.19001	36.52225	0.01046	36.52225	0.61097	36.52225	1.20579	36.5223	1.80203	36.52225	2.39708
160.047	0.18994	36.54194	0.00656	36.54194	0.61302	36.54194	1.206	36.5419	1.8068	36.54194	2.40213
160.147	0.18943	36.56163	0.00836	36.56163	0.60894	36.56163	1.20616	36.5616	1.80213	36.56163	2.40329
160.247	0.18954	36.58132	0.00809	36.58132	0.60918	36.58132	1.20761	36.5813	1.8019	36.58132	2.4038
160.347	0.18893	36.60101	0.00912	36.60101	0.60873	36.60101	1.2073	36.601	1.80261	36.60101	2.40199
160.447	0.18872	36.62069	0.00295	36.62069	0.61172	36.62069	1.20534	36.6207	1.80126	36.62069	2.39886
160.547	0.18924	36.64038	0.0078	36.64038	0.6092	36.64038	1.20555	36.6404	1.80165	36.64038	2.40572
160.647	0.18888	36.66007	0.01318	36.66007	0.60743	36.66007	1.21082	36.6601	1.80111	36.66007	2.40483
160.747	0.18861	36.67976	0.01304	36.67976	0.61167	36.67976	1.20405	36.6798	1.80038	36.67976	2.40672
160.847	0.18837	36.69945	0.00816	36.69945	0.61016	36.69945	1.204	36.6995	1.8009	36.69945	2.4014
160.947	0.18821	36.71914	0.01594	36.71914	0.60758	36.71914	1.20478	36.7191	1.80524	36.71914	2.40469
161.047	0.1883	36.73883	0.01891	36.73883	0.60963	36.73883	1.20881	36.7388	1.80295	36.73883	2.40338
161.147	0.1881	36.75852	0.01482	36.75852	0.60743	36.75852	1.20256	36.7585	1.80116	36.75852	2.4077
161.247	0.18786	36.77821	0.00917	36.77821	0.6041	36.77821	1.20546	36.7782	1.80293	36.77821	2.40723
161.347	0.18795	36.7979	0.01085	36.7979	0.61134	36.7979	1.20422	36.7979	1.80189	36.7979	2.40688
161.447	0.18781	36.81758	0.0098	36.81758	0.61327	36.81758	1.20329	36.8176	1.80684	36.81758	2.40702
161.547	0.18779	36.83728	0.01128	36.83728	0.61463	36.83728	1.2082	36.8373	1.80499	36.83728	2.40279
161.647	0.18761	36.85696	0.01147	36.85696	0.60924	36.85696	1.20733	36.857	1.80301	36.85696	2.41172
161.747	0.18775	36.87665	0.0088	36.87665	0.61185	36.87665	1.20666	36.8767	1.80328	36.87665	2.40155
161.847	0.18767	36.89634	0.00931	36.89634	0.6084	36.89634	1.20248	36.8963	1.80312	36.89634	2.40823
161.947	0.18763	36.91603	0.01002	36.91603	0.61039	36.91603	1.20573	36.916	1.80339	36.91603	2.40601
162.047	0.18759	36.93572	0.00826	36.93572	0.60706	36.93572	1.20351	36.9357	1.80322	36.93572	2.40809
162.147	0.18734	36.95541	0.01189	36.95541	0.61311	36.95541	1.20259	36.9554	1.80018	36.95541	2.40531

162.247	0.1877	36.9751	0.00708	36.9751	0.60941	36.9751	1.20755	36.9751	1.80402	36.9751	2.40685
162.347	0.18739	36.99479	0.00708	36.99479	0.60533	36.99479	1.20512	36.9948	1.80085	36.99479	2.40705
162.447	0.18754	37.01448	0.00778	37.01448	0.60907	37.01448	1.20229	37.0145	1.80837	37.01448	2.40725
162.547	0.18768	37.03416	0.0044	37.03416	0.60762	37.03416	1.20167	37.0342	1.80702	37.03416	2.4052
162.647	0.18762	37.05386	0.00466	37.05386	0.60423	37.05386	1.20389	37.0539	1.80267	37.05386	2.40364
162.747	0.18766	37.07354	0.00439	37.07354	0.6064	37.07354	1.20348	37.0735	1.80132	37.07354	2.40117
162.847	0.18725	37.09323	0.00186	37.09323	0.60533	37.09323	1.20648	37.0932	1.80259	37.09323	2.40313
162.947	0.1872	37.11292	0.00153	37.11292	0.60412	37.11292	1.19765	37.1129	1.80161	37.11292	2.40575
163.047	0.1878	37.13261	0.00133	37.13261	0.60104	37.13261	1.20168	37.1326	1.80276	37.13261	2.40401
163.147	0.18778	37.1523	0.0058	37.1523	0.60791	37.1523	1.20107	37.1523	1.8049	37.1523	2.40482
163.247	0.18789	37.17199	0.00255	37.17199	0.61059	37.17199	1.202	37.172	1.80611	37.17199	2.40199
163.347	0.18764	37.19168	1.65E-05	37.19168	0.60313	37.19168	1.20216	37.1917	1.80195	37.19168	2.40298
163.447	0.18737	37.21137	7.66E-05	37.21137	0.6065	37.21137	1.19834	37.2114	1.8016	37.21137	2.4053
163.547	0.18734	37.23106	0.00124	37.23106	0.60792	37.23106	1.19994	37.2311	1.80224	37.23106	2.40701
163.647	0.18768	37.25075	-0.0035	37.25075	0.60447	37.25075	1.20134	37.2508	1.80295	37.25075	2.40351
163.747	0.18717	37.27044	0.00168	37.27044	0.60489	37.27044	1.20072	37.2704	1.80491	37.27044	2.40523
163.847	0.18729	37.29012	0.00155	37.29012	0.6	37.29012	1.20326	37.2901	1.80168	37.29012	2.40773
163.947	0.18739	37.30981	0.00466	37.30981	0.60291	37.30981	1.19975	37.3098	1.80371	37.30981	2.40332
164.047	0.18755	37.3295	0.00102	37.3295	0.60583	37.3295	1.20084	37.3295	1.80935	37.3295	2.40467
164.147	0.18767	37.34919	0.00134	37.34919	0.60348	37.34919	1.19939	37.3492	1.80469	37.34919	2.41039
164.247	0.18781	37.36888	0.0029	37.36888	0.60126	37.36888	1.2042	37.3689	1.8054	37.36888	2.40822
164.347	0.18779	37.38857	0.00458	37.38857	0.60443	37.38857	1.19987	37.3886	1.80361	37.38857	2.40254
164.447	0.18781	37.40826	0.00549	37.40826	0.60528	37.40826	1.2008	37.4083	1.80095	37.40826	2.40838
164.547	0.18794	37.42795	0.00386	37.42795	0.60844	37.42795	1.20137	37.428	1.80484	37.42795	2.40313
164.647	0.18804	37.44764	0.00334	37.44764	0.60697	37.44764	1.2006	37.4476	1.80368	37.44764	2.4029
164.747	0.18801	37.46733	0.00308	37.46733	0.60882	37.46733	1.20034	37.4673	1.80508	37.46733	2.40656
164.847	0.18767	37.48701	0.00469	37.48701	0.60704	37.48701	1.20608	37.487	1.80616	37.48701	2.40482
164.947	0.18761	37.5067	0.00197	37.5067	0.60038	37.5067	1.20402	37.5067	1.79856	37.5067	2.4038
165.047	0.18773	37.52639	0.00702	37.52639	0.60673	37.52639	1.19849	37.5264	1.80496	37.52639	2.40109
165.147	0.18804	37.54608	0.00598	37.54608	0.60295	37.54608	1.19726	37.5461	1.80273	37.54608	2.40457
165.247	0.18807	37.56577	0.00475	37.56577	0.60593	37.56577	1.20077	37.5658	1.80269	37.56577	2.40634
165.347	0.18795	37.58546	-0.00128	37.58546	0.60258	37.58546	1.19969	37.5855	1.80553	37.58546	2.40588
165.447	0.18809	37.60515	0.00611	37.60515	0.60212	37.60515	1.20057	37.6052	1.80167	37.60515	2.40056
165.547	0.188	37.62484	0.0052	37.62484	0.60603	37.62484	1.19815	37.6248	1.80895	37.62484	2.39961
165.647	0.18816	37.64453	0.00124	37.64453	0.606	37.64453	1.19888	37.6445	1.80091	37.64453	2.40175
165.747	0.18869	37.66422	0.00111	37.66422	0.60672	37.66422	1.19965	37.6642	1.80393	37.66422	2.40165
165.847	0.18881	37.68391	0.00182	37.68391	0.60288	37.68391	1.20219	37.6839	1.8037	37.68391	2.40415
165.947	0.18885	37.70359	-0.0022	37.70359	0.60247	37.70359	1.2024	37.7036	1.80316	37.70359	2.40399
166.047	0.189	37.72329	3.26E-04	37.72329	0.60445	37.72329	1.2007	37.7233	1.80219	37.72329	2.40534
166.147	0.18929	37.74297	0.00227	37.74297	0.60248	37.74297	1.19734	37.743	1.80446	37.74297	2.39851
166.247	0.18938	37.76266	0.0059	37.76266	0.60364	37.76266	1.20117	37.7627	1.80192	37.76266	2.40156
166.347	0.18949	37.78235	0.00804	37.78235	0.60505	37.78235	1.20122	37.7824	1.80132	37.78235	2.39733
166.447	0.1897	37.80204	0.00311	37.80204	0.60346	37.80204	1.2005	37.802	1.80178	37.80204	2.4065
166.547	0.19014	37.82173	0.00304	37.82173	0.60687	37.82173	1.20448	37.8217	1.80368	37.82173	2.40507

166.647	0.19022	37.84142	0.00207	37.84142	0.6009	37.84142	1.19979	37.8414	1.79989	37.84142	2.40193
166.747	0.19045	37.86111	0.00265	37.86111	0.60288	37.86111	1.2018	37.8611	1.80529	37.86111	2.40547
166.847	0.19079	37.8808	0.00311	37.8808	0.60848	37.8808	1.2	37.8808	1.80556	37.8808	2.40646
166.947	0.19091	37.90049	0.00576	37.90049	0.60463	37.90049	1.19535	37.9005	1.80096	37.90049	2.40775
167.047	0.19109	37.92017	0.00375	37.92017	0.60285	37.92017	1.2032	37.9202	1.80686	37.92017	2.41134
167.147	0.19152	37.93987	0.01115	37.93987	0.60533	37.93987	1.20216	37.9399	1.8025	37.93987	2.40239
167.247	0.19173	37.95955	0.00596	37.95955	0.60486	37.95955	1.20861	37.9596	1.80109	37.95955	2.40774
167.347	0.19197	37.97924	8.99E-04	37.97924	0.60233	37.97924	1.20453	37.9792	1.80099	37.97924	2.40231
167.447	0.19224	37.99893	0.00609	37.99893	0.60055	37.99893	1.19854	37.9989	1.80545	37.99893	2.40166
167.547	0.19231	38.01862	0.00369	38.01862	0.60403	38.01862	1.20091	38.0186	1.80379	38.01862	2.40307
167.647	0.19262	38.03831	0.00187	38.03831	0.60306	38.03831	1.20117	38.0383	1.80125	38.03831	2.40551
167.747	0.19292	38.058	0.00446	38.058	0.60272	38.058	1.20318	38.058	1.79871	38.058	2.40578
167.847	0.19279	38.07769	0.01192	38.07769	0.60382	38.07769	1.2025	38.0777	1.80398	38.07769	2.40464
167.947	0.19324	38.09738	0.00647	38.09738	0.60391	38.09738	1.20199	38.0974	1.79969	38.09738	2.40745
168.047	0.19338	38.11707	0.00271	38.11707	0.59907	38.11707	1.20105	38.1171	1.8049	38.11707	2.4051
168.147	0.19332	38.13676	0.0057	38.13676	0.59773	38.13676	1.20312	38.1368	1.80124	38.13676	2.40785
168.247	0.1934	38.15644	0.01043	38.15644	0.60395	38.15644	1.19821	38.1564	1.80189	38.15644	2.40781
168.347	0.19355	38.17613	0.00771	38.17613	0.60067	38.17613	1.19836	38.1761	1.80303	38.17613	2.40322
168.447	0.19399	38.19582	0.00505	38.19582	0.60046	38.19582	1.20373	38.1958	1.80406	38.19582	2.40967
168.547	0.19414	38.21551	5.75E-04	38.21551	0.60844	38.21551	1.20156	38.2155	1.80708	38.21551	2.4032
168.647	0.19409	38.2352	0.01004	38.2352	0.59996	38.2352	1.19861	38.2352	1.81085	38.2352	2.40013
168.747	0.19425	38.25489	0.00725	38.25489	0.60194	38.25489	1.19938	38.2549	1.80875	38.25489	2.40124
168.847	0.19407	38.27458	0.00628	38.27458	0.6031	38.27458	1.20217	38.2746	1.80584	38.27458	2.40423
168.947	0.19412	38.29427	0.00952	38.29427	0.60119	38.29427	1.1984	38.2943	1.80261	38.29427	2.40855
169.047	0.19443	38.31396	0.00362	38.31396	0.60148	38.31396	1.20247	38.314	1.80339	38.31396	2.4059
169.147	0.19442	38.33364	0.00369	38.33364	0.60095	38.33364	1.20108	38.3336	1.80435	38.33364	2.40234
169.247	0.19423	38.35334	0.00395	38.35334	0.60874	38.35334	1.20376	38.3533	1.79894	38.35334	2.40115
169.347	0.19464	38.37302	0.00972	38.37302	0.60327	38.37302	1.20076	38.373	1.80571	38.37302	2.40905
169.447	0.19448	38.39272	0.0044	38.39272	0.60524	38.39272	1.19745	38.3927	1.79942	38.39272	2.40434
169.547	0.19428	38.4124	0.00408	38.4124	0.60378	38.4124	1.20437	38.4124	1.80126	38.4124	2.40509
169.647	0.1944	38.43209	0.00285	38.43209	0.60394	38.43209	1.2039	38.4321	1.80509	38.43209	2.40468
169.747	0.19459	38.45178	0.00804	38.45178	0.60303	38.45178	1.1992	38.4518	1.8048	38.45178	2.40367
169.847	0.1941	38.47147	0.00544	38.47147	0.60501	38.47147	1.20209	38.4715	1.8042	38.47147	2.40102
169.947	0.19404	38.49116	0.01057	38.49116	0.59803	38.49116	1.20467	38.4912	1.80016	38.49116	2.40147
170.047	0.1941	38.51085	0.00992	38.51085	0.60351	38.51085	1.20353	38.5109	1.80106	38.51085	2.40367
170.147	0.1941	38.53054	0.00564	38.53054	0.60179	38.53054	1.20146	38.5305	1.80008	38.53054	2.40799
170.247	0.19442	38.55023	0.00214	38.55023	0.60514	38.55023	1.20265	38.5502	1.80267	38.55023	2.40734
170.347	0.19438	38.56992	0.00454	38.56992	0.60242	38.56992	1.19759	38.5699	1.80401	38.56992	2.40409
170.447	0.19394	38.5896	0.00227	38.5896	0.60064	38.5896	1.20481	38.5896	1.80253	38.5896	2.40029
170.547	0.1937	38.60929	0.00571	38.60929	0.60162	38.60929	1.19867	38.6093	1.80505	38.60929	2.4051
170.647	0.19365	38.62898	0.00298	38.62898	0.60735	38.62898	1.1982	38.629	1.80751	38.62898	2.39839
170.747	0.19346	38.64867	0.00447	38.64867	0.60112	38.64867	1.20062	38.6487	1.80454	38.64867	2.39707
170.847	0.19324	38.66836	0	38.66836	0.60097	38.66836	1.2	38.6684	1.8	38.66836	2.4
170.947	0.19337	38.68805	0.00515	38.68805	0.60313	38.68805	1.19715	38.6881	1.80235	38.68805	2.40159

171.047	0.19326	38.70774	0.01049	38.70774	0.60104	38.70774	1.20008	38.7077	1.79957	38.70774	2.40772
171.147	0.19305	38.72743	0.00578	38.72743	0.60064	38.72743	1.19898	38.7274	1.79985	38.72743	2.40106
171.247	0.19291	38.74712	0.00218	38.74712	0.60586	38.74712	1.20129	38.7471	1.80457	38.74712	2.40713
171.347	0.19263	38.76681	0.00441	38.76681	0.60283	38.76681	1.20092	38.7668	1.80441	38.76681	2.40696
171.447	0.19285	38.7865	0.00547	38.7865	0.60255	38.7865	1.19791	38.7865	1.8002	38.7865	2.40285
171.547	0.19273	38.80618	0.00374	38.80618	0.5999	38.80618	1.20338	38.8062	1.80654	38.80618	2.40395
171.647	0.19275	38.82587	0.00513	38.82587	0.603	38.82587	1.20089	38.8259	1.79826	38.82587	2.40353
171.747	0.19288	38.84556	0.00165	38.84556	0.60904	38.84556	1.20345	38.8456	1.80323	38.84556	2.40373
171.847	0.19233	38.86525	0.00421	38.86525	0.6	38.86525	1.2005	38.8653	1.80295	38.86525	2.40034
171.947	0.19232	38.88494	0.00158	38.88494	0.59907	38.88494	1.201	38.8849	1.7998	38.88494	2.40417
172.047	0.19196	38.90463	0.00478	38.90463	0.60076	38.90463	1.1996	38.9046	1.80033	38.90463	2.40612
172.147	0.19176	38.92432	0.00403	38.92432	0.6019	38.92432	1.19793	38.9243	1.79842	38.92432	2.4031
172.247	0.19194	38.94401	0.00496	38.94401	0.60284	38.94401	1.20076	38.944	1.80283	38.94401	2.40468
172.347	0.19149	38.9637	0.01043	38.9637	0.60185	38.9637	1.19822	38.9637	1.80524	38.9637	2.40281
172.447	0.1916	38.98339	0.00715	38.98339	0.60304	38.98339	1.20131	38.9834	1.80546	38.98339	2.40258
172.547	0.19158	39.00307	0.00329	39.00307	0.60586	39.00307	1.20393	39.0031	1.80336	39.00307	2.40217
172.647	0.19146	39.02277	0.00104	39.02277	0.60587	39.02277	1.20051	39.0228	1.8034	39.02277	2.40454
172.747	0.19126	39.04245	0.00788	39.04245	0.60056	39.04245	1.2008	39.0425	1.80006	39.04245	2.40667
172.847	0.19118	39.06214	0.00401	39.06214	0.6	39.06214	1.20152	39.0621	1.80796	39.06214	2.40498
172.947	0.19119	39.08183	0.00236	39.08183	0.60306	39.08183	1.19975	39.0818	1.79956	39.08183	2.40451
173.047	0.1911	39.10152	0.00919	39.10152	0.60413	39.10152	1.20144	39.1015	1.80109	39.10152	2.40294
173.147	0.19105	39.12121	0.0063	39.12121	0.60738	39.12121	1.20101	39.1212	1.79975	39.12121	2.40089
173.247	0.1908	39.1409	0.00535	39.1409	0.60769	39.1409	1.20539	39.1409	1.80272	39.1409	2.40042
173.347	0.1905	39.16059	0.00292	39.16059	0.60575	39.16059	1.20574	39.1606	1.80344	39.16059	2.4051
173.447	0.18973	39.18028	0.0115	39.18028	0.59886	39.18028	1.20087	39.1803	1.80028	39.18028	2.40268
173.547	0.18995	39.19997	0.00557	39.19997	0.60418	39.19997	1.19921	39.2	1.80025	39.19997	2.3982
173.647	0.18991	39.21965	0.00929	39.21965	0.60236	39.21965	1.20405	39.2197	1.79847	39.21965	2.40034
173.747	0.19015	39.23935	0.01081	39.23935	0.60912	39.23935	1.19898	39.2394	1.80063	39.23935	2.40435
173.847	0.19011	39.25903	0.0048	39.25903	0.60768	39.25903	1.20191	39.259	1.80585	39.25903	2.40296
173.947	0.18995	39.27872	0.0082	39.27872	0.60787	39.27872	1.2052	39.2787	1.80263	39.27872	2.40237
174.047	0.19018	39.29841	0.01102	39.29841	0.60849	39.29841	1.19941	39.2984	1.80198	39.29841	2.39838
174.147	0.19012	39.3181	0.01455	39.3181	0.61124	39.3181	1.19754	39.3181	1.80126	39.3181	2.40033
174.247	0.19021	39.33779	0.00861	39.33779	0.60886	39.33779	1.20362	39.3378	1.79848	39.33779	2.39755
174.347	0.19	39.35748	0.01305	39.35748	0.61186	39.35748	1.20108	39.3575	1.80176	39.35748	2.40313
174.447	0.18979	39.37717	0.00886	39.37717	0.61236	39.37717	1.20215	39.3772	1.80423	39.37717	2.39799
174.547	0.19002	39.39686	0.01201	39.39686	0.60441	39.39686	1.20394	39.3969	1.79564	39.39686	2.40042
174.647	0.18996	39.41655	0.01366	39.41655	0.61305	39.41655	1.20187	39.4166	1.80423	39.41655	2.40328
174.747	0.19011	39.43624	0.01057	39.43624	0.60641	39.43624	1.20129	39.4362	1.79908	39.43624	2.40299
174.847	0.18998	39.45592	0.00794	39.45592	0.61254	39.45592	1.20236	39.4559	1.80567	39.45592	2.40203
174.947	0.18971	39.47561	0.00953	39.47561	0.6121	39.47561	1.19976	39.4756	1.80352	39.47561	2.40168
175.047	0.19022	39.4953	0.01429	39.4953	0.60897	39.4953	1.20672	39.4953	1.8008	39.4953	2.40163
175.147	0.19016	39.51499	0.00894	39.51499	0.61154	39.51499	1.20144	39.515	1.79915	39.51499	2.40394
175.247	0.1903	39.53468	0.01305	39.53468	0.61435	39.53468	1.19957	39.5347	1.80024	39.53468	2.40164
175.347	0.19026	39.55437	0.01704	39.55437	0.60934	39.55437	1.20772	39.5544	1.80009	39.55437	2.39784

175.447	0.1902	39.57406	0.01662	39.57406	0.61103	39.57406	1.20528	39.5741	1.80012	39.57406	2.39882
175.547	0.19036	39.59375	0.01814	39.59375	0.61109	39.59375	1.20031	39.5938	1.79997	39.59375	2.40313
175.647	0.1906	39.61344	0.01246	39.61344	0.60458	39.61344	1.20324	39.6134	1.80119	39.61344	2.40466
175.747	0.19078	39.63313	0.01048	39.63313	0.60652	39.63313	1.20163	39.6331	1.80384	39.63313	2.39915
175.847	0.1906	39.65282	0.02108	39.65282	0.60877	39.65282	1.20358	39.6528	1.8	39.65282	2.40061
175.947	0.19048	39.6725	0.01372	39.6725	0.60458	39.6725	1.20104	39.6725	1.79883	39.6725	2.39953
176.047	0.19063	39.6922	0.01414	39.6922	0.6072	39.6922	1.19958	39.6922	1.80048	39.6922	2.39748
176.147	0.1906	39.71188	0.01528	39.71188	0.60645	39.71188	1.2005	39.7119	1.80281	39.71188	2.39761
176.247	0.19053	39.73157	0.01745	39.73157	0.60307	39.73157	1.20141	39.7316	1.80045	39.73157	2.39593
176.347	0.19121	39.75126	0.01197	39.75126	0.60601	39.75126	1.20192	39.7513	1.79709	39.75126	2.40145
176.447	0.19095	39.77095	0.01252	39.77095	0.60444	39.77095	1.20335	39.771	1.7968	39.77095	2.40134
176.547	0.19116	39.79064	0.01249	39.79064	0.59687	39.79064	1.20029	39.7906	1.80619	39.79064	2.40171
176.647	0.19124	39.81033	0.01836	39.81033	0.59906	39.81033	1.20457	39.8103	1.79653	39.81033	2.40451
176.747	0.19148	39.83002	0.01029	39.83002	0.60099	39.83002	1.20528	39.83	1.80054	39.83002	2.40167
176.847	0.19105	39.84971	0.01148	39.84971	0.60462	39.84971	1.2	39.8497	1.8	39.84971	2.40017
176.947	0.19118	39.8694	0.00828	39.8694	0.60124	39.8694	1.20394	39.8694	1.79916	39.8694	2.40054
177.047	0.19145	39.88908	0.00838	39.88908	0.60418	39.88908	1.20323	39.8891	1.796	39.88908	2.39703
177.147	0.19143	39.90878	0.00673	39.90878	0.60117	39.90878	1.2096	39.9088	1.7976	39.90878	2.39941
177.247	0.19167	39.92846	0.00501	39.92846	0.59892	39.92846	1.2047	39.9285	1.80394	39.92846	2.39863
177.347	0.19155	39.94815	0.00848	39.94815	0.60217	39.94815	1.20394	39.9482	1.80372	39.94815	2.40028
177.447	0.19156	39.96784	0.00858	39.96784	0.60455	39.96784	1.20132	39.9678	1.80394	39.96784	2.3992
177.547	0.19164	39.98753	0.00667	39.98753	0.60355	39.98753	1.20103	39.9875	1.79535	39.98753	2.40405
177.647	0.19151	40.00722	0.00515	40.00722	0.60079	40.00722	1.20321	40.0072	1.80575	40.00722	2.39879
177.747	0.19142										
177.847	0.19189										
177.947	0.1918										
178.047	0.1916										
178.147	0.19172										
178.247	0.1918										
178.347	0.19214										
178.447	0.19202										
178.547	0.19221										
178.647	0.19245										
178.747	0.19229										
178.847	0.19224										
178.947	0.19257										
179.047	0.1929										
179.147	0.19266										
179.247	0.19249										
179.347	0.19226										
179.447	0.19204										
179.547	0.19213										
179.647	0.19216										
179.747	0.19229										

179.847	0.192
179.947	0.19189
180.047	0.19236
180.147	0.19235
180.247	0.19228
180.347	0.1923
180.447	0.1923
180.547	0.19238
180.647	0.19295
180.747	0.19322
180.847	0.19336
180.947	0.19326
181.047	0.19372
181.147	0.19348
181.247	0.1936
181.347	0.19387
181.447	0.19381
181.547	0.19418
181.647	0.19443
181.747	0.19441
181.847	0.19482
181.947	0.19501
182.047	0.19578
182.147	0.19613
182.247	0.19612
182.347	0.19629
182.447	0.19658
182.547	0.19667
182.647	0.19702
182.747	0.19733
182.847	0.19743
182.947	0.19802
183.047	0.19865
183.147	0.19911
183.247	0.19961
183.347	0.19999
183.447	0.20028
183.547	0.20065
183.647	0.20109
183.747	0.20124
183.847	0.20169
183.947	0.20219
184.047	0.20257
184.147	0.20266

184.247	0.20297
184.347	0.20331
184.447	0.20341
184.547	0.20377
184.647	0.20397
184.747	0.20434
184.847	0.20457
184.947	0.20481
185.047	0.20507
185.147	0.2053
185.247	0.20549
185.347	0.20581
185.447	0.20672
185.547	0.20649
185.647	0.20652
185.747	0.20704
185.847	0.20702
185.947	0.20716
186.047	0.20737
186.147	0.20785
186.247	0.2078
186.347	0.20781
186.447	0.20869
186.547	0.20862
186.647	0.20886
186.747	0.20884
186.847	0.20923
186.947	0.20938
187.047	0.20972
187.147	0.20982
187.247	0.21003
187.347	0.21019
187.447	0.21038
187.547	0.21062
187.647	0.21113
187.747	0.21133
187.847	0.21126
187.947	0.21166
188.047	0.21183
188.147	0.21221
188.247	0.21204
188.347	0.21255
188.447	0.21234
188.547	0.2126

188.647	0.21311
188.747	0.21346
188.847	0.21338
188.947	0.21339
189.047	0.21328
189.147	0.21347
189.247	0.21387
189.347	0.21417
189.447	0.2145
189.547	0.21429
189.647	0.21415
189.747	0.21441
189.847	0.21451
189.947	0.21418
190.047	0.21406
190.147	0.2137
190.247	0.21343
190.347	0.21308
190.447	0.21281
190.547	0.21221
190.647	0.21201
190.747	0.21184
190.847	0.21106
190.947	0.21029
191.047	0.20928
191.147	0.20833
191.247	0.20729
191.347	0.20588
191.447	0.20478
191.547	0.2026
191.647	0.20102
191.747	0.20001
191.847	0.19791
191.947	0.19578
192.047	0.1933
192.147	0.19073
192.247	0.18829
192.347	0.18488
192.447	0.18206
192.547	0.17819
192.647	0.17512
192.747	0.17048
192.847	0.16581
192.947	0.16088

193.047	0.1565
193.147	0.1515
193.247	0.1455
193.347	0.13936
193.447	0.13242
193.547	0.1255
193.647	0.11819
193.747	0.11058
193.847	0.10281
193.947	0.09381
194.047	0.08418
194.147	0.07456
194.247	0.06402
194.347	0.05284
194.447	0.04243
194.547	0.03177
194.647	0.02023
194.747	0.00762
194.847	-0.00544
194.947	-0.0183
195.047	-0.03253
195.147	-0.04652
195.247	-0.06052
195.347	-0.0749
195.447	-0.08918
195.547	-0.1046
195.647	-0.12027
195.747	-0.13556
195.847	-0.15164
195.947	-0.1674
196.047	-0.18303
196.147	-0.19857
196.247	-0.21347
196.347	-0.22851
196.447	-0.24217
196.547	-0.25627
196.647	-0.26978
196.747	-0.28351
196.847	-0.297
196.947	-0.30798
197.047	-0.31872
197.147	-0.32881
197.247	-0.33807
197.347	-0.3445

197.447	-0.35039
197.547	-0.35629
197.647	-0.36205
197.747	-0.36668
197.847	-0.36874
197.947	-0.36929
198.047	-0.36952
198.147	-0.36897
198.247	-0.36784
198.347	-0.36397
198.447	-0.3597
198.547	-0.35339
198.647	-0.34754
198.747	-0.34074
198.847	-0.33187
198.947	-0.32281
199.047	-0.31373
199.147	-0.30391
199.247	-0.29187
199.347	-0.27946
199.447	-0.26729
199.547	-0.25518
199.647	-0.2428
199.747	-0.22938
199.847	-0.21524
199.947	-0.20145
200.047	-0.18775
200.147	-0.17326
200.247	-0.15899
200.347	-0.14493
200.447	-0.13104
200.547	-0.11694
200.647	-0.1027
200.747	-0.08891
200.847	-0.07485
200.947	-0.06225
201.047	-0.04858
201.147	-0.03515
201.247	-0.02254
201.347	-0.01082
201.447	0.0015
201.547	0.01302
201.647	0.02325
201.747	0.03389

201.847	0.04434
201.947	0.05377
202.047	0.06244
202.147	0.07134
202.247	0.07853
202.347	0.08591
202.447	0.09243
202.547	0.099
202.647	0.10496
202.747	0.11047
202.847	0.11533
202.947	0.11985
203.047	0.12267
203.147	0.12693
203.247	0.12904
203.347	0.13331
203.447	0.13555
203.547	0.13706
203.647	0.1397
203.747	0.14117
203.847	0.14333
203.947	0.14469
204.047	0.14572
204.147	0.14662
204.247	0.14754
204.347	0.14806
204.447	0.14893
204.547	0.14984
204.647	0.15047
204.747	0.1512
204.847	0.15157
204.947	0.15171
205.047	0.15267
205.147	0.1523
205.247	0.15244
205.347	0.15246
205.447	0.15285
205.547	0.15292
205.647	0.15309
205.747	0.15322
205.847	0.15301
205.947	0.15308
206.047	0.15328
206.147	0.15329

206.247	0.15345
206.347	0.15366
206.447	0.15354
206.547	0.15324
206.647	0.15313
206.747	0.15331
206.847	0.15335
206.947	0.1533
207.047	0.15313
207.147	0.15324
207.247	0.15344
207.347	0.15356
207.447	0.15403
207.547	0.15358
207.647	0.15365
207.747	0.15357
207.847	0.15352
207.947	0.15365
208.047	0.15346
208.147	0.15333
208.247	0.15384
208.347	0.15395
208.447	0.15391
208.547	0.15369
208.647	0.15392
208.747	0.15403
208.847	0.15389
208.947	0.15401
209.047	0.15384
209.147	0.15353
209.247	0.15385
209.347	0.15387
209.447	0.15364
209.547	0.15373
209.647	0.15389
209.747	0.15345
209.847	0.15368
209.947	0.15342
210.047	0.15365
210.147	0.15378
210.247	0.15351
210.347	0.15359
210.447	0.15331
210.547	0.15338

210.647	0.15355
210.747	0.15363
210.847	0.1535
210.947	0.1535
211.047	0.15324
211.147	0.15316
211.247	0.15303
211.347	0.15332
211.447	0.1532
211.547	0.15313
211.647	0.15286
211.747	0.15272
211.847	0.15274
211.947	0.15281
212.047	0.1529
212.147	0.15277
212.247	0.1521
212.347	0.15204
212.447	0.1522
212.547	0.152
212.647	0.1517
212.747	0.15187
212.847	0.15196
212.947	0.15182
213.047	0.15183
213.147	0.15175
213.247	0.15171
213.347	0.15142
213.447	0.15143
213.547	0.15174
213.647	0.1516
213.747	0.15206
213.847	0.15184
213.947	0.15173
214.047	0.1514
214.147	0.15178
214.247	0.15196
214.347	0.15199
214.447	0.15196
214.547	0.15235
214.647	0.15233
214.747	0.15256
214.847	0.15246
214.947	0.15268

215.047	0.15332
215.147	0.15304
215.247	0.15357
215.347	0.15369
215.447	0.15368
215.547	0.15345
215.647	0.15327
215.747	0.15361
215.847	0.1541
215.947	0.15452
216.047	0.15454
216.147	0.15432
216.247	0.15415
216.347	0.15389
216.447	0.15352
216.547	0.15392
216.647	0.15409
216.747	0.1541
216.847	0.15412
216.947	0.15388
217.047	0.15399
217.147	0.15348
217.247	0.1538
217.347	0.15359
217.447	0.15339
217.547	0.15332
217.647	0.15342
217.747	0.15306
217.847	0.15285
217.947	0.15293
218.047	0.15317
218.147	0.1531
218.247	0.15319
218.347	0.15297
218.447	0.15255
218.547	0.15263
218.647	0.15214
218.747	0.15221
218.847	0.15222
218.947	0.15216
219.047	0.15224
219.147	0.15181
219.247	0.1519
219.347	0.15175

219.447	0.15185
219.547	0.15143
219.647	0.15114
219.747	0.15065
219.847	0.15028
219.947	0.14942
220.047	0.14956
220.147	0.14943
220.247	0.14923
220.347	0.14887
220.447	0.14874
220.547	0.14821
220.647	0.14786
220.747	0.14809
220.847	0.14803
220.947	0.14745
221.047	0.14731
221.147	0.14704
221.247	0.14689
221.347	0.14675
221.447	0.14654
221.547	0.14648
221.647	0.14612
221.747	0.14605
221.847	0.14584
221.947	0.14549
222.047	0.1455
222.147	0.1455
222.247	0.14536
222.347	0.14529
222.447	0.14518
222.547	0.14488
222.647	0.14485
222.747	0.14489
222.847	0.1447
222.947	0.1447
223.047	0.14478
223.147	0.14457
223.247	0.14421
223.347	0.14419
223.447	0.14386
223.547	0.1436
223.647	0.14332
223.747	0.14306

223.847	0.14314
223.947	0.14333
224.047	0.14331
224.147	0.14293
224.247	0.14321
224.347	0.14384
224.447	0.14365
224.547	0.14349
224.647	0.14335
224.747	0.14373
224.847	0.14381
224.947	0.14372
225.047	0.14325
225.147	0.14312
225.247	0.14305
225.347	0.14294
225.447	0.14294
225.547	0.14309
225.647	0.14332
225.747	0.14336
225.847	0.14341
225.947	0.14342
226.047	0.14334
226.147	0.14316
226.247	0.14328
226.347	0.14323
226.447	0.14291
226.547	0.14322
226.647	0.143
226.747	0.14314
226.847	0.14307
226.947	0.14331
227.047	0.1435
227.147	0.14311
227.247	0.14315
227.347	0.1431
227.447	0.14322
227.547	0.1434
227.647	0.14316
227.747	0.14313
227.847	0.14291
227.947	0.14366
228.047	0.14394
228.147	0.14385

228.247	0.14327
228.347	0.1432
228.447	0.14331
228.547	0.14346
228.647	0.14332
228.747	0.14377
228.847	0.14381
228.947	0.14387
229.047	0.14414
229.147	0.14394
229.247	0.14415
229.347	0.1441
229.447	0.14426
229.547	0.14472
229.647	0.14487
229.747	0.1453
229.847	0.14564
229.947	0.14585
230.047	0.14594
230.147	0.14647
230.247	0.14674
230.347	0.14708
230.447	0.14743
230.547	0.14754
230.647	0.1479
230.747	0.14824
230.847	0.14809
230.947	0.14852
231.047	0.14914
231.147	0.14958
231.247	0.15012
231.347	0.15088
231.447	0.15123
231.547	0.15156
231.647	0.15182
231.747	0.15236
231.847	0.15274
231.947	0.15329
232.047	0.15393
232.147	0.15399
232.247	0.15395
232.347	0.15432
232.447	0.15469
232.547	0.15478

232.647	0.15533
232.747	0.1557
232.847	0.15601
232.947	0.15639
233.047	0.15677
233.147	0.15708
233.247	0.15739
233.347	0.15747
233.447	0.15794
233.547	0.15802
233.647	0.15865
233.747	0.15876
233.847	0.15879
233.947	0.15925
234.047	0.15913
234.147	0.1595
234.247	0.15962
234.347	0.15956
234.447	0.16
234.547	0.16026
234.647	0.16025
234.747	0.16044
234.847	0.16042
234.947	0.1606
235.047	0.16078
235.147	0.16127
235.247	0.16111
235.347	0.16168
235.447	0.1612
235.547	0.16119
235.647	0.16157
235.747	0.16229
235.847	0.16218
235.947	0.16242
236.047	0.16257
236.147	0.16277
236.247	0.16273
236.347	0.16309
236.447	0.16321
236.547	0.16357
236.647	0.16397
236.747	0.16399
236.847	0.16387
236.947	0.16412

237.047	0.16434
237.147	0.16402
237.247	0.16448
237.347	0.16459
237.447	0.16472
237.547	0.16494
237.647	0.16505
237.747	0.16566
237.847	0.1654
237.947	0.16545
238.047	0.16594
238.147	0.16578
238.247	0.16647
238.347	0.16649
238.447	0.16658
238.547	0.16644
238.647	0.16675
238.747	0.16706
238.847	0.16708
238.947	0.16717
239.047	0.16763
239.147	0.16801
239.247	0.16788
239.347	0.16835
239.447	0.1691
239.547	0.16912
239.647	0.16942
239.747	0.16937
239.847	0.16974
239.947	0.16991
240.047	0.17053
240.147	0.17091
240.247	0.17122
240.347	0.17161
240.447	0.1716
240.547	0.17134
240.647	0.17188
240.747	0.17187
240.847	0.17236
240.947	0.17296
241.047	0.17315
241.147	0.17391
241.247	0.17407
241.347	0.17451

241.447	0.17451
241.547	0.1744
241.647	0.1747
241.747	0.17527
241.847	0.17491
241.947	0.1753
242.047	0.17584
242.147	0.17594
242.247	0.17612
242.347	0.17636
242.447	0.1762
242.547	0.17654
242.647	0.17702
242.747	0.17699
242.847	0.17719
242.947	0.17737
243.047	0.17712
243.147	0.17747
243.247	0.17774
243.347	0.17815
243.447	0.17806
243.547	0.1787
243.647	0.1791
243.747	0.17913
243.847	0.17903
243.947	0.17895
244.047	0.17937
244.147	0.1793
244.247	0.17931
244.347	0.17972
244.447	0.18006
244.547	0.18021
244.647	0.18015
244.747	0.18032
244.847	0.1807
244.947	0.18081
245.047	0.18089
245.147	0.18084
245.247	0.1808
245.347	0.18115
245.447	0.1811
245.547	0.18119
245.647	0.18107
245.747	0.18123

245.847	0.18138
245.947	0.18157
246.047	0.18188
246.147	0.18179
246.247	0.18208
246.347	0.18267
246.447	0.1826
246.547	0.18302
246.647	0.18315
246.747	0.18348
246.847	0.18364
246.947	0.18395
247.047	0.18382
247.147	0.18388
247.247	0.18431
247.347	0.18472
247.447	0.18504
247.547	0.18518
247.647	0.18567
247.747	0.18579
247.847	0.18585
247.947	0.18631
248.047	0.18655
248.147	0.18635
248.247	0.18643
248.347	0.18673
248.447	0.18659
248.547	0.18692
248.647	0.1873
248.747	0.18723
248.847	0.1871
248.947	0.18719
249.047	0.18713
249.147	0.18767
249.247	0.18798
249.347	0.1883
249.447	0.1881
249.547	0.18842
249.647	0.1882
249.747	0.18807
249.847	0.18808
249.947	0.18817
250.047	0.1881
250.147	0.18826

250.247	0.18857
250.347	0.18853
250.447	0.18858
250.547	0.1887
250.647	0.18886
250.747	0.18865
250.847	0.18816
250.947	0.18854
251.047	0.18854
251.147	0.18815
251.247	0.18777
251.347	0.18811
251.447	0.188
251.547	0.18827
251.647	0.18812
251.747	0.18764
251.847	0.18727
251.947	0.18727
252.047	0.18699
252.147	0.18682
252.247	0.18729
252.347	0.18704
252.447	0.18689
252.547	0.18675
252.647	0.18692
252.747	0.18679
252.847	0.18674
252.947	0.18666
253.047	0.18662
253.147	0.18661
253.247	0.1864
253.347	0.1862
253.447	0.18645
253.547	0.18644
253.647	0.18609
253.747	0.18626
253.847	0.18582
253.947	0.18575
254.047	0.18532
254.147	0.18533
254.247	0.18535
254.347	0.18529
254.447	0.18515
254.547	0.18516

254.647	0.18496
254.747	0.18482
254.847	0.18499
254.947	0.18515
255.047	0.18493
255.147	0.18464
255.247	0.18436
255.347	0.18393
255.447	0.18415
255.547	0.18389
255.647	0.18385
255.747	0.18399
255.847	0.18378
255.947	0.18388
256.047	0.18395
256.147	0.18404
256.247	0.18428
256.347	0.18441
256.447	0.18432
256.547	0.18456
256.647	0.18445
256.747	0.18446
256.847	0.18485
256.947	0.18494
257.047	0.18456
257.147	0.18474
257.247	0.18476
257.347	0.18467
257.447	0.18438
257.547	0.18431
257.647	0.18444
257.747	0.18476
257.847	0.18469
257.947	0.18467
258.047	0.18453
258.147	0.18468
258.247	0.1848
258.347	0.18478
258.447	0.18478
258.547	0.18484
258.647	0.18535
258.747	0.1854
258.847	0.18541
258.947	0.18545

259.047	0.18541
259.147	0.18525
259.247	0.18519
259.347	0.18524
259.447	0.18522
259.547	0.18578
259.647	0.18549
259.747	0.18527
259.847	0.18516
259.947	0.18518
260.047	0.18533
260.147	0.18586
260.247	0.18582
260.347	0.18535
260.447	0.18538
260.547	0.18539
260.647	0.18571
260.747	0.18554
260.847	0.18527
260.947	0.18516
261.047	0.18559
261.147	0.18566
261.247	0.18558
261.347	0.18544
261.447	0.18544
261.547	0.18547
261.647	0.18525
261.747	0.18535
261.847	0.18539
261.947	0.18523
262.047	0.18495
262.147	0.1849
262.247	0.18512
262.347	0.18491
262.447	0.1854
262.547	0.18491
262.647	0.18502
262.747	0.18492
262.847	0.18555
262.947	0.18615
263.047	0.18654
263.147	0.18656
263.247	0.18658
263.347	0.18669

263.447	0.18731
263.547	0.18755
263.647	0.18764
263.747	0.18787
263.847	0.18804
263.947	0.18852
264.047	0.18853
264.147	0.18885
264.247	0.18871
264.347	0.18873
264.447	0.18897
264.547	0.18937
264.647	0.18939
264.747	0.18968
264.847	0.18971
264.947	0.19019
265.047	0.19027
265.147	0.19055
265.247	0.19067
265.347	0.19088
265.447	0.19052
265.547	0.1908
265.647	0.19067
265.747	0.19097
265.847	0.1912
265.947	0.19092
266.047	0.19101
266.147	0.19101
266.247	0.19143
266.347	0.19161
266.447	0.19187
266.547	0.19203
266.647	0.19215
266.747	0.19161
266.847	0.19167
266.947	0.1923
267.047	0.19203
267.147	0.19175
267.247	0.19183
267.347	0.19225
267.447	0.19216
267.547	0.19206
267.647	0.19182
267.747	0.19183

267.847	0.19226
267.947	0.19235
268.047	0.19235
268.147	0.19209
268.247	0.19216
268.347	0.19234
268.447	0.19211
268.547	0.19246
268.647	0.19248
268.747	0.19236
268.847	0.19213
268.947	0.19234
269.047	0.19235
269.147	0.19244
269.247	0.19235
269.347	0.19241
269.447	0.19241
269.547	0.19233
269.647	0.19231
269.747	0.19222
269.847	0.19198
269.947	0.19192
270.047	0.19203
270.147	0.1918
270.247	0.19184
270.347	0.19169
270.447	0.19178
270.547	0.19173
270.647	0.19201
270.747	0.19165
270.847	0.19128
270.947	0.19124
271.047	0.19142
271.147	0.19158
271.247	0.19176
271.347	0.19156
271.447	0.19169
271.547	0.19194
271.647	0.19186
271.747	0.19158
271.847	0.19185
271.947	0.19209
272.047	0.19195
272.147	0.19213

272.247	0.19222
272.347	0.19239
272.447	0.19271
272.547	0.19304
272.647	0.19283
272.747	0.19269
272.847	0.19294
272.947	0.19364
273.047	0.19366
273.147	0.19368
273.247	0.19392
273.347	0.19426
273.447	0.19426
273.547	0.19436
273.647	0.19469
273.747	0.19502
273.847	0.19533
273.947	0.19597
274.047	0.19597
274.147	0.19622
274.247	0.19717
274.347	0.19727
274.447	0.1976
274.547	0.19792
274.647	0.19869
274.747	0.1987
274.847	0.19898
274.947	0.19967
275.047	0.19977
275.147	0.19994
275.247	0.20005
275.347	0.20103
275.447	0.20129
275.547	0.20178
275.647	0.20195
275.747	0.20258
275.847	0.20313
275.947	0.20347
276.047	0.20386
276.147	0.20386
276.247	0.20514
276.347	0.20569
276.447	0.20665
276.547	0.20702

276.647	0.20781
276.747	0.2083
276.847	0.20845
276.947	0.2091
277.047	0.20918
277.147	0.20953
277.247	0.21007
277.347	0.21087
277.447	0.21111
277.547	0.21152
277.647	0.21191
277.747	0.21278
277.847	0.21288
277.947	0.21313
278.047	0.21376
278.147	0.21409
278.247	0.21464
278.347	0.21528
278.447	0.21591
278.547	0.21647
278.647	0.21765
278.747	0.21822
278.847	0.21791
278.947	0.21928
279.047	0.21936
279.147	0.22037
279.247	0.22156
279.347	0.22154
279.447	0.22173
279.547	0.22187
279.647	0.22319
279.747	0.22387
279.847	0.22389
279.947	0.22449
280.047	0.2253

Figure 4e

Wavelength (nm)	Intensity (a.u.)	Intensity (a.u.)	Intensity (a.u.)	Intensity (a.u.)	Intensity (a.u.)	Intensity (a.u.)	Intensity (a.u.)	Intensity (a.u.)	Intensity (a.u.)
450.01	0.00664	0.00496	0.00416	0.00208	0.00955	7.47E-05	1.77E-04	2.02E-04	0.00459
450.8	0.00716	0.00501	0.00451	0.00224	0.01014	9.33E-05	1.59E-04	2.21E-04	0.00448
451.6	0.0077	0.00525	0.00472	0.00232	0.01085	1.12E-04	1.59E-04	2.39E-04	0.0045
452.39	0.00836	0.00572	0.00512	0.0027	0.01178	1.87E-04	1.77E-04	1.84E-04	0.00456
453.18	0.00912	0.00572	0.00533	0.00273	0.01251	1.87E-04	2.48E-04	2.94E-04	0.00484
453.97	0.0097	0.00611	0.00556	0.00295	0.01359	2.05E-04	1.59E-04	2.94E-04	0.00476
454.77	0.01061	0.00637	0.006	0.00338	0.01457	3.17E-04	2.83E-04	4.23E-04	0.00476
455.56	0.01165	0.00676	0.00644	0.00348	0.01551	1.49E-04	2.12E-04	4.41E-04	0.00484
456.35	0.01285	0.0072	0.00725	0.00387	0.01686	3.55E-04	4.07E-04	3.86E-04	0.00491
457.14	0.01396	0.00778	0.00754	0.00433	0.01806	3.36E-04	3.36E-04	5.15E-04	0.00482
457.94	0.01512	0.00839	0.00811	0.00473	0.0195	4.11E-04	4.25E-04	5.70E-04	0.00489
458.73	0.01674	0.0088	0.00879	0.0052	0.02109	3.36E-04	4.60E-04	5.52E-04	0.00518
459.52	0.01817	0.00947	0.00933	0.00555	0.02275	3.92E-04	4.95E-04	6.80E-04	0.0051
460.31	0.01993	0.01064	0.01025	0.00623	0.02518	5.97E-04	5.31E-04	9.01E-04	0.0051
461.1	0.0219	0.01114	0.01118	0.00667	0.02634	5.04E-04	5.84E-04	8.09E-04	0.00533
461.89	0.02404	0.01225	0.01205	0.00749	0.02899	7.28E-04	8.14E-04	9.74E-04	0.00525
462.69	0.02601	0.01298	0.01309	0.00814	0.03109	6.72E-04	6.37E-04	0.00103	0.00534
463.48	0.02859	0.01433	0.01419	0.00901	0.03359	7.84E-04	9.20E-04	0.00118	0.00551
464.27	0.03114	0.01559	0.0156	0.00993	0.03639	8.21E-04	0.00101	0.0014	0.00559
465.06	0.03389	0.01691	0.01658	0.01079	0.03922	9.52E-04	0.00108	0.00138	0.00557
465.85	0.03673	0.01821	0.01809	0.01184	0.043	0.00112	0.00126	0.00164	0.00566
466.64	0.0404	0.01996	0.02004	0.01283	0.04608	0.00129	0.00129	0.00169	0.00593
467.43	0.04441	0.02176	0.0217	0.01428	0.0502	0.00136	0.00161	0.00208	0.00593
468.22	0.04878	0.0238	0.02356	0.0157	0.05461	0.00146	0.00165	0.00219	0.00628
469.01	0.05289	0.02562	0.02558	0.01725	0.05948	0.00166	0.00191	0.00239	0.00628
469.81	0.05761	0.02811	0.02748	0.01864	0.06431	0.00187	0.00203	0.00259	0.00662
470.6	0.06298	0.03017	0.02967	0.02031	0.06996	0.00203	0.0023	0.00281	0.00662
471.39	0.0694	0.03347	0.03235	0.02191	0.07637	0.00231	0.0026	0.00307	0.00694
472.18	0.07541	0.03598	0.03493	0.02406	0.08277	0.00237	0.00271	0.00335	0.00724
472.97	0.08283	0.03917	0.03793	0.02611	0.09039	0.00278	0.00304	0.00382	0.00744
473.76	0.09033	0.0427	0.04099	0.02836	0.09793	0.00308	0.00347	0.00412	0.00773
474.55	0.09825	0.04604	0.04421	0.03109	0.10611	0.00329	0.00386	0.00456	0.00808
475.34	0.10785	0.05029	0.04771	0.0335	0.11594	0.00373	0.00435	0.00482	0.00842
476.13	0.11772	0.05432	0.05099	0.03591	0.12583	0.00405	0.00474	0.00531	0.00866
476.92	0.12871	0.05892	0.0556	0.03917	0.13647	0.0045	0.00536	0.00566	0.00906
477.71	0.14016	0.0641	0.0596	0.04198	0.14763	0.00508	0.0057	0.00631	0.00934
478.5	0.15283	0.06891	0.06472	0.04531	0.16075	0.00543	0.00625	0.00678	0.0099
479.29	0.16702	0.07496	0.06951	0.04947	0.17325	0.00601	0.00695	0.00735	0.01026
480.08	0.18162	0.0812	0.07518	0.05287	0.18656	0.00672	0.0077	0.00822	0.01086
480.87	0.19682	0.08738	0.08083	0.05751	0.20201	0.00709	0.00856	0.00866	0.01144

481.66	0.21294	0.09464	0.0879	0.06164	0.21702	0.00788	0.00906	0.00936	0.01198
482.45	0.23087	0.10368	0.09522	0.06632	0.23309	0.00857	0.01009	0.01006	0.01241
483.24	0.24929	0.11192	0.10392	0.07217	0.25075	0.00937	0.01092	0.01094	0.0133
484.03	0.2689	0.12156	0.11176	0.07763	0.26898	0.01032	0.01203	0.01177	0.01421
484.82	0.28958	0.13238	0.12159	0.0833	0.28742	0.01122	0.01327	0.01281	0.01487
485.61	0.31187	0.14387	0.13143	0.09011	0.3066	0.0124	0.01433	0.01414	0.01613
486.39	0.33409	0.15598	0.14217	0.09755	0.32696	0.0135	0.01554	0.01513	0.01688
487.18	0.3555	0.16951	0.15326	0.10519	0.34672	0.01438	0.01681	0.01677	0.01811
487.97	0.38081	0.18399	0.16529	0.11333	0.36831	0.01572	0.01851	0.01813	0.01948
488.76	0.40425	0.19973	0.17871	0.12302	0.38831	0.01701	0.02033	0.01991	0.02083
489.55	0.43063	0.21642	0.19289	0.13226	0.4107	0.01856	0.02169	0.02164	0.02213
490.34	0.45528	0.23382	0.2084	0.14226	0.43293	0.02007	0.02382	0.02372	0.02337
491.13	0.47978	0.25366	0.22431	0.15412	0.45558	0.02177	0.02562	0.02611	0.0253
491.92	0.50613	0.27375	0.24139	0.16617	0.4781	0.02384	0.02826	0.02892	0.0271
492.7	0.53175	0.29387	0.25867	0.17842	0.5004	0.02569	0.03038	0.03146	0.02909
493.49	0.55623	0.31721	0.27683	0.19231	0.52213	0.02828	0.03274	0.03482	0.03075
494.28	0.58156	0.33928	0.29692	0.20649	0.54544	0.03062	0.03553	0.03771	0.03345
495.07	0.60752	0.36351	0.31651	0.22141	0.56766	0.03321	0.03835	0.04188	0.0358
495.86	0.63164	0.38872	0.33741	0.23801	0.58835	0.03611	0.04172	0.04594	0.03837
496.65	0.65587	0.41322	0.35845	0.25518	0.61022	0.03902	0.04551	0.05067	0.04122
497.43	0.67985	0.43945	0.37955	0.27247	0.6315	0.04283	0.04925	0.05521	0.04424
498.22	0.70305	0.46559	0.4008	0.2912	0.65176	0.04598	0.05335	0.06054	0.04724
499.01	0.72388	0.48961	0.42384	0.30994	0.67224	0.05086	0.0582	0.06679	0.05048
499.8	0.74537	0.51594	0.44673	0.33071	0.69035	0.05541	0.06314	0.07367	0.05444
500.59	0.76687	0.54198	0.46973	0.35032	0.71064	0.06054	0.0694	0.08053	0.05834
501.37	0.78744	0.56857	0.49239	0.37237	0.72862	0.06631	0.07543	0.08775	0.0623
502.16	0.80739	0.59493	0.51632	0.39454	0.747	0.07223	0.08202	0.097	0.06704
502.95	0.82504	0.61888	0.53904	0.41742	0.76575	0.07877	0.08978	0.10566	0.07124
503.74	0.84402	0.64321	0.56309	0.43974	0.781	0.08683	0.09787	0.11509	0.07589
504.52	0.86099	0.66612	0.58666	0.4629	0.79873	0.09478	0.10624	0.12581	0.08092
505.31	0.87673	0.68972	0.60962	0.48718	0.81364	0.10384	0.1164	0.13588	0.08705
506.1	0.89158	0.71251	0.63241	0.51178	0.8276	0.11383	0.12659	0.14815	0.09279
506.88	0.90666	0.73123	0.65431	0.53488	0.84162	0.12464	0.13761	0.15982	0.09866
507.67	0.91615	0.75232	0.67559	0.55766	0.8537	0.1353	0.14954	0.17308	0.10447
508.46	0.92962	0.77086	0.6971	0.58275	0.86512	0.14782	0.16228	0.18725	0.11197
509.24	0.93953	0.79024	0.71749	0.60565	0.87668	0.16136	0.17589	0.19962	0.11822
510.03	0.94995	0.80628	0.73851	0.63093	0.8893	0.17458	0.18934	0.21494	0.12607
510.82	0.95974	0.82641	0.7574	0.65286	0.89793	0.19059	0.20493	0.23	0.13311
511.61	0.96692	0.83862	0.77602	0.67538	0.90612	0.20641	0.22039	0.24664	0.14063
512.39	0.97502	0.85453	0.79346	0.69801	0.91682	0.22407	0.23718	0.26281	0.14935
513.18	0.98324	0.86754	0.81123	0.72159	0.92505	0.24261	0.25437	0.27943	0.15823
513.96	0.98669	0.88162	0.82687	0.74149	0.93208	0.26066	0.27282	0.29758	0.16754
514.75	0.99078	0.89474	0.84334	0.76318	0.93997	0.28123	0.29207	0.31626	0.17695
515.54	0.99328	0.90558	0.85896	0.78487	0.94543	0.30183	0.31189	0.3342	0.18606

516.32	0.99587	0.91824	0.87462	0.80452	0.95148	0.32387	0.33222	0.35314	0.19565
517.11	1	0.92782	0.88724	0.82409	0.95825	0.3465	0.35326	0.37136	0.2069
517.89	0.99898	0.93872	0.89982	0.84039	0.96019	0.36965	0.37492	0.39099	0.21775
518.68	0.9988	0.94722	0.91152	0.8553	0.9657	0.39319	0.39575	0.41083	0.22829
519.47	0.99944	0.95396	0.92291	0.87319	0.97122	0.41728	0.41751	0.43008	0.24101
520.25	0.99966	0.96395	0.9334	0.8873	0.97615	0.44269	0.4414	0.45187	0.2514
521.04	0.99789	0.97115	0.9434	0.90334	0.97967	0.46834	0.46398	0.47189	0.26391
521.82	0.99737	0.9778	0.95229	0.91624	0.98407	0.49395	0.48771	0.49206	0.27701
522.61	0.99623	0.98296	0.96049	0.92943	0.98725	0.51951	0.5108	0.51248	0.28926
523.39	0.99184	0.98936	0.96789	0.94167	0.98941	0.54649	0.53497	0.52993	0.30227
524.18	0.98839	0.99087	0.97407	0.9508	0.99156	0.57044	0.55741	0.55107	0.31765
524.97	0.9855	0.99376	0.98138	0.96119	0.99371	0.5957	0.58165	0.57209	0.33156
525.75	0.98219	0.99868	0.98607	0.96947	0.9966	0.62311	0.60522	0.59253	0.34495
526.54	0.9766	0.99913	0.98988	0.9745	0.99816	0.6471	0.62715	0.6113	0.36018
527.32	0.96886	0.99976	0.9931	0.98085	0.99759	0.67107	0.64982	0.62936	0.37389
528.11	0.96521	1	0.99619	0.9865	1	0.69519	0.67213	0.64796	0.38998
528.89	0.95743	0.99918	0.99709	0.99142	0.99789	0.71826	0.69437	0.6669	0.40455
529.67	0.95059	0.99701	0.99803	0.99519	0.99749	0.73934	0.71605	0.68563	0.41966
530.46	0.94466	0.99629	1	0.99722	0.9975	0.761	0.73737	0.70266	0.43523
531.24	0.93538	0.99454	0.99758	0.99778	0.99796	0.7808	0.75307	0.71884	0.45062
532.03	0.92742	0.9907	0.99698	1	0.99766	0.79998	0.77458	0.73487	0.46688
532.81	0.91771	0.98567	0.99632	0.99688	0.99406	0.81962	0.79249	0.75138	0.4813
533.6	0.91081	0.98147	0.99368	0.99671	0.99243	0.83724	0.81164	0.7662	0.4976
534.38	0.90422	0.97635	0.98958	0.99568	0.98929	0.853	0.82714	0.77984	0.51298
535.17	0.89263	0.97102	0.98782	0.99435	0.987	0.86769	0.84382	0.7932	0.5277
535.95	0.88421	0.96363	0.98417	0.98862	0.98374	0.88317	0.85798	0.80771	0.54433
536.73	0.87671	0.95864	0.98079	0.98626	0.98134	0.89665	0.87192	0.82007	0.55958
537.52	0.86662	0.95025	0.97444	0.98118	0.97974	0.90998	0.88571	0.83362	0.57565
538.3	0.8569	0.94312	0.96973	0.97928	0.977	0.92159	0.90041	0.84402	0.59086
539.09	0.84569	0.93475	0.96461	0.97273	0.97277	0.93315	0.9131	0.85505	0.60543
539.87	0.83833	0.92871	0.9587	0.968	0.96706	0.94102	0.92258	0.86478	0.62058
540.65	0.83051	0.91811	0.95291	0.9616	0.96425	0.95055	0.93184	0.87533	0.63708
541.44	0.81926	0.91139	0.94745	0.95705	0.95933	0.95732	0.94238	0.88576	0.65195
542.22	0.81084	0.90395	0.94029	0.94995	0.95574	0.96541	0.94991	0.89335	0.6651
543	0.80236	0.89463	0.93345	0.94235	0.95333	0.9702	0.95875	0.90243	0.68034
543.79	0.79145	0.88507	0.92796	0.93676	0.94569	0.97707	0.96511	0.90942	0.69486
544.57	0.78361	0.87471	0.92178	0.92887	0.9404	0.98243	0.97208	0.91703	0.70881
545.35	0.77535	0.86493	0.9154	0.92095	0.9363	0.9846	0.9774	0.92477	0.72177
546.14	0.7644	0.85962	0.90706	0.91426	0.9325	0.98934	0.98238	0.93016	0.73548
546.92	0.75434	0.84913	0.90134	0.90708	0.92671	0.99233	0.98666	0.93727	0.74804
547.7	0.74533	0.84048	0.89319	0.90134	0.92319	0.99591	0.98891	0.94442	0.76169
548.49	0.73819	0.83045	0.88629	0.89324	0.91683	0.998	0.99283	0.94858	0.77392
549.27	0.72855	0.82136	0.87969	0.88505	0.91051	0.99673	0.99526	0.95503	0.78654
550.05	0.71933	0.81395	0.87283	0.87624	0.90366	0.99834	0.99676	0.95862	0.79809

550.83	0.71125	0.80485	0.8658	0.86953	0.89737	0.99755	0.99597	0.96349	0.80831
551.62	0.70272	0.79479	0.85973	0.86122	0.89372	1	0.99839	0.96876	0.81876
552.4	0.69402	0.78651	0.85152	0.85233	0.88663	0.99968	0.99857	0.97178	0.83063
553.18	0.68642	0.77927	0.84618	0.84733	0.88122	0.99909	0.99798	0.97496	0.84178
553.96	0.67919	0.77246	0.83788	0.83933	0.87524	0.9981	1	0.97926	0.85386
554.75	0.67249	0.76199	0.8323	0.83182	0.87082	0.99666	0.99681	0.98404	0.86322
555.53	0.66485	0.75393	0.82523	0.826	0.86432	0.99658	0.99549	0.98803	0.87441
556.31	0.65693	0.74699	0.81941	0.81687	0.85836	0.99604	0.99579	0.9897	0.88342
557.09	0.64819	0.73943	0.81172	0.80919	0.85188	0.99279	0.99414	0.99121	0.89283
557.87	0.63892	0.73236	0.80483	0.8008	0.84537	0.98811	0.99052	0.99513	0.89902
558.66	0.63443	0.72293	0.79799	0.79528	0.83843	0.98421	0.988	0.99553	0.90766
559.44	0.62394	0.71372	0.79146	0.78878	0.83164	0.98098	0.98768	0.99581	0.91678
560.22	0.61556	0.70646	0.78455	0.77777	0.82318	0.97849	0.98101	0.99776	0.92383
561	0.60865	0.70008	0.77804	0.77168	0.81724	0.97426	0.97841	0.99816	0.92938
561.78	0.60061	0.69141	0.77019	0.763	0.81123	0.96977	0.97524	1	0.93618
562.56	0.59375	0.68268	0.76314	0.75622	0.80377	0.96563	0.97073	0.99915	0.94164
563.34	0.58577	0.67381	0.75651	0.74791	0.79747	0.9619	0.96557	0.99847	0.94751
564.13	0.57827	0.66907	0.75051	0.73893	0.78981	0.95669	0.96311	0.99844	0.95297
564.91	0.56941	0.65933	0.74174	0.73122	0.78204	0.95208	0.95764	0.99822	0.95781
565.69	0.56233	0.65352	0.7354	0.72526	0.77385	0.9461	0.95307	0.99857	0.96435
566.47	0.55325	0.64605	0.7287	0.71688	0.76816	0.94099	0.94934	0.99893	0.96812
567.25	0.54595	0.63787	0.72163	0.70984	0.76087	0.93481	0.94605	0.99614	0.97266
568.03	0.54053	0.63154	0.71502	0.70304	0.75395	0.92995	0.94122	0.99678	0.97819
568.81	0.53267	0.62604	0.70854	0.6938	0.74558	0.92353	0.93589	0.99465	0.97935
569.59	0.52307	0.61557	0.70003	0.68571	0.7386	0.9167	0.93094	0.99279	0.98344
570.37	0.51639	0.60995	0.6934	0.67895	0.73146	0.91201	0.92628	0.99152	0.98511
571.15	0.50773	0.60328	0.68693	0.66932	0.72378	0.9052	0.92115	0.98959	0.99027
571.93	0.50318	0.59485	0.68031	0.66236	0.71766	0.89769	0.91678	0.98827	0.98974
572.71	0.49381	0.58815	0.67157	0.65601	0.70907	0.89332	0.91041	0.98606	0.99338
573.5	0.48836	0.57993	0.66561	0.64721	0.70297	0.8864	0.90565	0.98327	0.99376
574.28	0.48002	0.57228	0.65882	0.6405	0.69583	0.87996	0.90029	0.9802	0.99546
575.06	0.47176	0.5679	0.65206	0.63226	0.68857	0.87404	0.89556	0.97865	0.99692
575.84	0.46598	0.55934	0.64499	0.62338	0.68111	0.8665	0.8907	0.9775	0.99801
576.62	0.45935	0.55104	0.63838	0.61663	0.67319	0.85989	0.88284	0.9734	0.99788
577.4	0.45319	0.54544	0.62994	0.61053	0.66616	0.85318	0.87983	0.97047	1
578.18	0.44461	0.53775	0.62266	0.60108	0.6594	0.84639	0.87435	0.96913	0.99871
578.96	0.43809	0.53201	0.61601	0.59412	0.65099	0.83969	0.86775	0.96373	0.99902
579.73	0.43051	0.52331	0.60927	0.58618	0.6432	0.83332	0.86288	0.96056	0.99946
580.51	0.42398	0.51644	0.60299	0.5776	0.63727	0.82632	0.85648	0.95847	0.99844
581.29	0.4178	0.5084	0.59511	0.57077	0.63119	0.81799	0.85207	0.95569	0.99659
582.07	0.41191	0.50209	0.58902	0.56316	0.62365	0.81125	0.84719	0.95194	0.99871
582.85	0.40507	0.49789	0.58201	0.55703	0.61519	0.80405	0.84205	0.94655	0.99458
583.63	0.39855	0.48729	0.57564	0.54978	0.60958	0.79761	0.83593	0.94398	0.99477
584.41	0.39226	0.48323	0.56748	0.54145	0.60079	0.79234	0.8303	0.94025	0.99334

585.19	0.38608	0.47539	0.5613	0.53418	0.59341	0.78461	0.8256	0.93589	0.99111
585.97	0.3792	0.46851	0.55507	0.52794	0.58663	0.77709	0.81863	0.92993	0.98847
586.75	0.37337	0.46177	0.54686	0.52041	0.58062	0.76919	0.81261	0.92534	0.98547
587.53	0.36691	0.45616	0.54084	0.51292	0.5723	0.76255	0.80672	0.92188	0.98404
588.31	0.3616	0.44857	0.53307	0.50601	0.567	0.75506	0.80038	0.91841	0.97952
589.08	0.35705	0.44218	0.52765	0.49899	0.56024	0.74798	0.79568	0.9139	0.97868
589.86	0.34961	0.43565	0.52135	0.49212	0.55388	0.74083	0.78814	0.9083	0.97547
590.64	0.3451	0.42963	0.51539	0.48527	0.54625	0.73415	0.78476	0.90486	0.97219
591.42	0.33898	0.42403	0.5087	0.47809	0.5384	0.72814	0.77779	0.89861	0.96771
592.2	0.33335	0.41614	0.50182	0.47068	0.53204	0.7203	0.76943	0.89368	0.96469
592.98	0.32663	0.41105	0.49437	0.46454	0.52606	0.7127	0.7668	0.88978	0.96159
593.76	0.323	0.40448	0.48926	0.45826	0.52033	0.70723	0.75975	0.8864	0.95796
594.53	0.31765	0.3988	0.48175	0.45186	0.51463	0.6995	0.75308	0.88077	0.95293
595.31	0.31297	0.39217	0.47693	0.44529	0.50681	0.69282	0.74682	0.87458	0.94905
596.09	0.3085	0.38788	0.47096	0.43853	0.50092	0.68652	0.74135	0.86855	0.94509
596.87	0.30265	0.38066	0.46466	0.43249	0.49456	0.67754	0.73516	0.8634	0.94166
597.65	0.29788	0.37557	0.45945	0.42532	0.48851	0.67067	0.72852	0.85901	0.93705
598.42	0.29186	0.36924	0.4521	0.41954	0.48071	0.6644	0.72208	0.85309	0.93326
599.2	0.28833	0.36406	0.44686	0.41363	0.4761	0.65731	0.71619	0.84788	0.92775
599.98	0.28332	0.35914	0.44203	0.40827	0.46912	0.65174	0.70964	0.84136	0.92321
600.76	0.27935	0.35177	0.43429	0.40134	0.46304	0.64258	0.70566	0.83492	0.91696
601.53	0.27361	0.34687	0.42799	0.39535	0.45662	0.63707	0.6989	0.82986	0.91066
602.31	0.26942	0.34184	0.42145	0.38977	0.45056	0.63044	0.69209	0.82391	0.90759
603.09	0.26491	0.33718	0.41668	0.38443	0.44439	0.62368	0.6846	0.81711	0.89973
603.87	0.26038	0.33176	0.41122	0.37793	0.43832	0.61569	0.67747	0.80911	0.89598
604.64	0.25587	0.32625	0.40592	0.37331	0.43221	0.60869	0.67307	0.80571	0.88957
605.42	0.2523	0.32127	0.4004	0.36663	0.42598	0.60098	0.66652	0.79973	0.88548
606.2	0.24919	0.31697	0.39519	0.36146	0.42047	0.59566	0.66034	0.7935	0.87912
606.97	0.24482	0.31223	0.3892	0.3565	0.41675	0.58803	0.65442	0.78773	0.87158
607.75	0.24037	0.30759	0.38561	0.35131	0.40973	0.58198	0.64746	0.78293	0.86871
608.53	0.23604	0.30239	0.37936	0.34566	0.40378	0.57544	0.64206	0.77408	0.86142
609.3	0.23195	0.29673	0.37536	0.34061	0.39954	0.56921	0.63584	0.76943	0.85699
610.08	0.22858	0.29374	0.36953	0.33551	0.39358	0.56131	0.62916	0.76224	0.85105
610.86	0.22573	0.28851	0.36401	0.3288	0.3872	0.55532	0.6238	0.75375	0.84492
611.63	0.22156	0.28546	0.35955	0.32602	0.38266	0.55007	0.61899	0.74857	0.84124
612.41	0.21797	0.2798	0.35564	0.32167	0.37769	0.54404	0.6126	0.74494	0.83672
613.19	0.21491	0.27644	0.35131	0.31714	0.37302	0.53713	0.60816	0.74072	0.83005
613.96	0.21072	0.27386	0.34694	0.3132	0.36715	0.53293	0.60331	0.73494	0.82643
614.74	0.20831	0.26892	0.34216	0.30738	0.36285	0.52683	0.59668	0.72963	0.82134
615.51	0.2047	0.26439	0.33744	0.30397	0.35692	0.51988	0.59112	0.7225	0.81446
616.29	0.20135	0.26042	0.33376	0.29892	0.35292	0.51462	0.58502	0.71799	0.80884
617.07	0.19772	0.25676	0.32987	0.29475	0.34823	0.50739	0.57967	0.71203	0.8019
617.84	0.19417	0.25264	0.32488	0.2911	0.34237	0.50256	0.5742	0.70545	0.79528
618.62	0.19233	0.2496	0.31978	0.28533	0.33732	0.49563	0.56778	0.69911	0.78765

619.39	0.18862	0.24564	0.31546	0.28134	0.33282	0.48936	0.56276	0.69257	0.78129
620.17	0.18451	0.2408	0.31129	0.27702	0.32837	0.4818	0.55663	0.68615	0.77531
620.94	0.18186	0.23673	0.30679	0.27224	0.32303	0.47614	0.55002	0.67955	0.76951
621.72	0.17809	0.23372	0.30155	0.26818	0.31785	0.47013	0.54294	0.67344	0.76164
622.49	0.17563	0.22981	0.29748	0.26366	0.31353	0.46402	0.53892	0.66736	0.75732
623.27	0.17296	0.22708	0.29418	0.26088	0.30932	0.4604	0.53434	0.66225	0.75232
624.05	0.17029	0.22385	0.29076	0.25653	0.30572	0.45549	0.52883	0.65727	0.7448
624.82	0.16791	0.22077	0.28709	0.25375	0.30163	0.44942	0.52574	0.65272	0.74005
625.6	0.16542	0.21739	0.28369	0.25112	0.2987	0.44453	0.52066	0.64734	0.73549
626.37	0.16337	0.21512	0.28048	0.24716	0.29355	0.43931	0.51744	0.64315	0.73075
627.15	0.16105	0.21286	0.2783	0.24438	0.29189	0.43589	0.51307	0.63903	0.72526
627.92	0.15846	0.20998	0.27379	0.24049	0.28719	0.43049	0.50742	0.63401	0.72074
628.69	0.15578	0.20703	0.27112	0.2375	0.28353	0.42583	0.50406	0.62798	0.71344
629.47	0.15299	0.20439	0.2673	0.23359	0.27929	0.42066	0.49789	0.62276	0.70934
630.24	0.15154	0.20129	0.26283	0.22977	0.27483	0.41569	0.49314	0.61801	0.7021
631.02	0.14848	0.19747	0.2597	0.22712	0.27138	0.40902	0.48861	0.61042	0.69461
631.79	0.14629	0.19429	0.25667	0.22243	0.26827	0.40613	0.48269	0.60638	0.68916
632.57	0.1436	0.19136	0.25228	0.21931	0.26362	0.39939	0.47747	0.60136	0.68323
633.34	0.14134	0.18872	0.24939	0.2167	0.26012	0.39482	0.4732	0.59411	0.67665
634.12	0.13925	0.1857	0.24419	0.21286	0.25523	0.38983	0.46837	0.58954	0.67035
634.89	0.13631	0.18247	0.24149	0.20956	0.25192	0.38419	0.46278	0.58145	0.66448
635.66	0.13464	0.17972	0.23867	0.20712	0.24766	0.37915	0.45874	0.57551	0.65793
636.44	0.13167	0.17681	0.23465	0.20313	0.24409	0.37284	0.45282	0.57098	0.6509
637.21	0.12953	0.1733	0.23147	0.1992	0.23927	0.36853	0.44818	0.564	0.644
637.98	0.12758	0.17166	0.22796	0.19647	0.23601	0.36358	0.44367	0.55854	0.63956
638.76	0.12486	0.16871	0.22512	0.19306	0.23262	0.35824	0.43678	0.55203	0.63247
639.53	0.12317	0.166	0.22116	0.19043	0.22908	0.35333	0.43383	0.54635	0.62604
640.31	0.12129	0.16292	0.21717	0.18647	0.22531	0.34882	0.42771	0.54116	0.6182
641.08	0.11884	0.16006	0.21428	0.18352	0.22239	0.34407	0.42431	0.53565	0.61117
641.85	0.11626	0.15809	0.21158	0.18042	0.21787	0.33868	0.41884	0.52813	0.60406
642.63	0.11475	0.15507	0.20824	0.17815	0.2155	0.33424	0.41325	0.523	0.59856
643.4	0.11311	0.15314	0.20498	0.1754	0.21111	0.32899	0.40854	0.51763	0.59166
644.17	0.11036	0.1502	0.20161	0.17292	0.20877	0.32494	0.40426	0.51042	0.58542
644.94	0.1094	0.14762	0.19944	0.16953	0.2048	0.32098	0.40039	0.50553	0.57891
645.72	0.10669	0.14506	0.1961	0.16622	0.20236	0.31534	0.39511	0.50081	0.57308
646.49	0.10497	0.14311	0.1931	0.1641	0.19846	0.31025	0.39133	0.49443	0.56674
647.26	0.10366	0.14014	0.1901	0.16113	0.1961	0.30715	0.38533	0.48915	0.5614
648.04	0.10154	0.13832	0.18707	0.15934	0.19279	0.30268	0.38066	0.48305	0.55495
648.81	0.09981	0.13632	0.1841	0.156	0.19019	0.29867	0.37583	0.47981	0.54799
649.58	0.09843	0.13368	0.18091	0.15349	0.18718	0.29342	0.37225	0.47218	0.54259
650.35	0.0964	0.13171	0.17845	0.15065	0.18403	0.28945	0.36822	0.46681	0.53706
651.13	0.09426	0.12928	0.1755	0.1477	0.1803	0.28435	0.36349	0.46128	0.52841
651.9	0.09268	0.12672	0.17285	0.14543	0.17704	0.28058	0.35748	0.45441	0.52089
652.67	0.09059	0.12427	0.17015	0.14253	0.1743	0.27602	0.35372	0.44944	0.51481

653.44	0.08901	0.12182	0.16764	0.14036	0.17202	0.27253	0.34877	0.44409	0.51065
654.21	0.088	0.11974	0.16405	0.1389	0.16905	0.26772	0.3458	0.43874	0.50473
654.99	0.08652	0.11842	0.16284	0.13577	0.16618	0.26471	0.34205	0.43464	0.49897
655.76	0.0853	0.11679	0.15977	0.13441	0.1637	0.26055	0.33783	0.42959	0.49389
656.53	0.08375	0.11454	0.15731	0.13164	0.1612	0.25754	0.33313	0.42468	0.4882
657.3	0.08231	0.11293	0.15491	0.1292	0.15838	0.25368	0.32975	0.42043	0.48127
658.07	0.08062	0.11068	0.15298	0.12695	0.15607	0.24951	0.32534	0.41519	0.4771
658.84	0.07898	0.1091	0.15101	0.12514	0.15401	0.24638	0.32184	0.41056	0.47232
659.62	0.07808	0.10715	0.14852	0.12345	0.15087	0.24468	0.31849	0.40554	0.46593
660.39	0.07665	0.10578	0.14636	0.12166	0.14885	0.23975	0.31418	0.40061	0.46002
661.16	0.07563	0.10437	0.14399	0.11946	0.14685	0.23645	0.31072	0.39646	0.45544
661.93	0.07441	0.10242	0.14196	0.11736	0.14498	0.23344	0.30726	0.39206	0.45038
662.7	0.07297	0.10123	0.13955	0.1154	0.14209	0.22931	0.30379	0.38682	0.44393
663.47	0.07122	0.09921	0.13797	0.11357	0.13951	0.22706	0.30057	0.38212	0.43935
664.24	0.0703	0.09722	0.13503	0.11169	0.13744	0.22203	0.29613	0.37838	0.43318
665.01	0.0695	0.09583	0.13276	0.10976	0.13467	0.21959	0.29236	0.3736	0.42718
665.79	0.06785	0.09384	0.13133	0.10773	0.13283	0.21636	0.28855	0.36858	0.4233
666.56	0.06695	0.09262	0.12869	0.10645	0.13023	0.21378	0.28507	0.3641	0.41856
667.33	0.06553	0.09065	0.12671	0.10408	0.12807	0.20997	0.28169	0.35912	0.41209
668.1	0.06428	0.08931	0.12475	0.10248	0.12651	0.20624	0.27797	0.35577	0.40729
668.87	0.06326	0.0877	0.12301	0.10021	0.12417	0.20349	0.27516	0.34998	0.40095
669.64	0.06246	0.0864	0.12041	0.09893	0.1216	0.20088	0.27093	0.34582	0.39643
670.41	0.06134	0.08484	0.11943	0.09765	0.12003	0.1976	0.2678	0.34152	0.39272
671.18	0.06055	0.08309	0.1176	0.09553	0.11767	0.19414	0.26463	0.33694	0.38623
671.95	0.05909	0.08215	0.11524	0.09412	0.11614	0.19164	0.26088	0.3338	0.38205
672.72	0.05841	0.08096	0.11464	0.09285	0.11393	0.18863	0.25743	0.32922	0.37747
673.49	0.05702	0.07925	0.1118	0.09103	0.11225	0.1857	0.25465	0.32437	0.37229
674.26	0.05624	0.07871	0.11017	0.08935	0.11034	0.18316	0.25169	0.32121	0.36734
675.03	0.05488	0.07695	0.10876	0.08828	0.1088	0.18048	0.24702	0.3176	0.36314
675.8	0.05436	0.07535	0.10703	0.0863	0.10699	0.17769	0.24444	0.31227	0.35868
676.57	0.05318	0.07448	0.10532	0.08499	0.10507	0.17543	0.24177	0.30881	0.35386
677.34	0.05195	0.07255	0.10306	0.08405	0.10344	0.17165	0.23841	0.30389	0.34829
678.11	0.05133	0.07242	0.10136	0.08208	0.10186	0.16985	0.23591	0.29868	0.34415
678.88	0.05081	0.07045	0.10031	0.08077	0.09958	0.1667	0.232	0.29639	0.34023
679.65	0.04957	0.06947	0.09873	0.07982	0.09757	0.16461	0.2289	0.29256	0.33591
680.42	0.0486	0.0685	0.09631	0.07775	0.09675	0.16162	0.22687	0.28879	0.33006
681.19	0.04816	0.0672	0.09557	0.0764	0.09488	0.15949	0.22367	0.28517	0.32599
681.96	0.04692	0.06583	0.09415	0.07536	0.09299	0.15645	0.22043	0.28078	0.32245
682.72	0.04644	0.0647	0.09246	0.07403	0.09114	0.15382	0.21707	0.27772	0.31829
683.49	0.0455	0.06325	0.09067	0.07295	0.09006	0.15169	0.21484	0.27399	0.31351
684.26	0.04451	0.06262	0.08916	0.07137	0.08819	0.14907	0.2117	0.27035	0.3091
685.03	0.04417	0.06139	0.08771	0.07069	0.08738	0.14719	0.20916	0.26693	0.30469
685.8	0.04295	0.06076	0.08678	0.06903	0.0854	0.14443	0.2065	0.26379	0.3022
686.57	0.04189	0.05961	0.08487	0.06804	0.08403	0.14276	0.20388	0.25927	0.29758

687.34	0.04175	0.05848	0.08425	0.06681	0.08265	0.14008	0.20098	0.25625	0.29344
688.11	0.04064	0.05733	0.08283	0.06611	0.08157	0.13819	0.19833	0.25259	0.28883
688.87	0.04018	0.0564	0.08132	0.06437	0.07925	0.13606	0.19562	0.24912	0.28386
689.64	0.03962	0.0559	0.07969	0.0635	0.07838	0.13334	0.19319	0.24551	0.28095
690.41	0.03868	0.05469	0.07881	0.06278	0.07778	0.13171	0.19042	0.24353	0.27686
691.18	0.03812	0.05395	0.07695	0.06138	0.07568	0.12998	0.18813	0.23969	0.27326
691.95	0.03746	0.05317	0.07599	0.06009	0.07424	0.12809	0.18544	0.2363	0.26991
692.72	0.03655	0.05224	0.07474	0.05946	0.07346	0.12521	0.18313	0.23226	0.26565
693.48	0.03607	0.05116	0.07348	0.05782	0.07164	0.12344	0.18021	0.23049	0.26205
694.25	0.03565	0.05042	0.0725	0.05705	0.07081	0.12096	0.17736	0.22671	0.25901
695.02	0.03521	0.04955	0.07111	0.05626	0.06975	0.11973	0.17578	0.22364	0.25522
695.79	0.03389	0.04856	0.07008	0.05507	0.06852	0.1175	0.17359	0.22064	0.25252
696.55	0.03359	0.04804	0.06883	0.05481	0.06746	0.11676	0.17083	0.21733	0.24887
697.32	0.0331	0.04671	0.06764	0.05336	0.06565	0.11403	0.16879	0.21439	0.24425
698.09	0.03224	0.04587	0.06713	0.0529	0.06499	0.11276	0.16637	0.21145	0.24131
698.86	0.0317	0.04511	0.06572	0.05152	0.06402	0.10989	0.1646	0.20819	0.23763
699.62	0.031	0.04463	0.06437	0.0506	0.06227	0.10864	0.16166	0.20593	0.23366
700.39	0.03092	0.04389	0.06321	0.04987	0.06187	0.10625	0.15926	0.20168	0.23049
701.16	0.03014	0.0429	0.06197	0.04908	0.06053	0.10492	0.15775	0.19986	0.22694
701.93	0.02939	0.04192	0.06085	0.04795	0.05949	0.10333	0.15503	0.19698	0.22398
702.69	0.02929	0.04136	0.06013	0.04734	0.05828	0.10167	0.15243	0.19391	0.22102
703.46	0.02873	0.0406	0.05953	0.04647	0.05728	0.09988	0.15048	0.19128	0.21813
704.23	0.02845	0.03991	0.05811	0.04548	0.05643	0.09837	0.14869	0.1892	0.21492
704.99	0.02779	0.03891	0.05728	0.04461	0.05523	0.09695	0.14671	0.18517	0.21097
705.76	0.02721	0.03876	0.05604	0.04423	0.05443	0.09495	0.14496	0.18304	0.20873
706.53	0.02643	0.03806	0.05553	0.04359	0.05381	0.0935	0.14253	0.1813	0.20656
707.29	0.02617	0.03735	0.05435	0.04231	0.0531	0.0923	0.14064	0.17811	0.20257
708.06	0.02593	0.03687	0.0536	0.04159	0.05164	0.09079	0.13944	0.17589	0.20018
708.83	0.02532	0.03648	0.05269	0.04116	0.05085	0.08913	0.13721	0.17394	0.19788
709.59	0.02504	0.03529	0.05185	0.04074	0.05014	0.0876	0.13512	0.17048	0.19518
710.36	0.02428	0.03494	0.05134	0.03954	0.04868	0.08653	0.13323	0.16912	0.19205
711.13	0.02384	0.03399	0.04978	0.03874	0.04785	0.08459	0.13112	0.16591	0.18859
711.89	0.02376	0.0336	0.04914	0.03814	0.04728	0.08388	0.12886	0.1635	0.18646
712.66	0.0229	0.03308	0.04862	0.03736	0.0461	0.08218	0.1273	0.1616	0.18243
713.42	0.02294	0.03269	0.04742	0.03662	0.04563	0.08093	0.12574	0.15883	0.18042
714.19	0.02242	0.03197	0.04678	0.03633	0.04494	0.07919	0.12379	0.15568	0.17757
714.96	0.02188	0.03134	0.04586	0.03533	0.04379	0.07796	0.12174	0.1542	0.17498
715.72	0.02131	0.03106	0.04472	0.03452	0.04289	0.07591	0.11981	0.1517	0.172
716.49	0.02085	0.02978	0.04399	0.03434	0.04206	0.07471	0.11811	0.14872	0.1693
717.25	0.02077	0.02976	0.04307	0.03324	0.0413	0.07376	0.11624	0.14627	0.16622
718.02	0.02021	0.02894	0.04232	0.03278	0.04034	0.07206	0.1139	0.14348	0.16311
718.78	0.01967	0.02811	0.04167	0.03188	0.03986	0.07104	0.11224	0.1418	0.15987
719.55	0.01943	0.0277	0.0409	0.03143	0.03868	0.06926	0.11028	0.13829	0.15737
720.31	0.01883	0.02694	0.04	0.03079	0.03761	0.06797	0.1084	0.13669	0.15448

721.08	0.01857	0.02666	0.03913	0.03015	0.03717	0.06687	0.1058	0.13423	0.15182
721.84	0.01813	0.02614	0.03818	0.02945	0.03627	0.06516	0.10468	0.13188	0.15004
722.61	0.01803	0.0254	0.03751	0.02913	0.03561	0.06417	0.10277	0.12954	0.14665
723.37	0.01756	0.0248	0.03665	0.02845	0.03506	0.06252	0.1012	0.1277	0.1446
724.14	0.01732	0.02482	0.03642	0.02807	0.03447	0.06166	0.09932	0.12539	0.14244
724.9	0.0168	0.0241	0.03541	0.02741	0.03369	0.06068	0.09865	0.12426	0.14079
725.67	0.0167	0.02382	0.03539	0.02683	0.03287	0.05985	0.09746	0.12202	0.13849
726.43	0.0162	0.02324	0.03448	0.02635	0.03255	0.05909	0.09584	0.12055	0.13688
727.2	0.01618	0.02287	0.03407	0.02606	0.03215	0.05769	0.09485	0.11954	0.13476
727.96	0.01584	0.02278	0.03327	0.02573	0.03183	0.05765	0.09384	0.1184	0.13294
728.73	0.0155	0.02241	0.03284	0.02544	0.03123	0.05644	0.09297	0.11625	0.13102
729.49	0.01542	0.0222	0.03267	0.02498	0.03088	0.05556	0.0921	0.11538	0.12937
730.25	0.01526	0.02181	0.03227	0.02469	0.03048	0.05539	0.09104	0.11404	0.12941
731.02	0.01498	0.02165	0.03142	0.02428	0.02974	0.05433	0.08945	0.11244	0.12714
731.78	0.01462	0.02094	0.03149	0.02384	0.02967	0.05412	0.08878	0.11108	0.12615
732.55	0.01464	0.021	0.03104	0.02338	0.02927	0.05276	0.08779	0.1098	0.12425
733.31	0.01432	0.02057	0.03076	0.02324	0.02858	0.05235	0.08692	0.10869	0.12247
734.07	0.01404	0.02016	0.03046	0.023	0.02828	0.0516	0.08563	0.10781	0.12178
734.84	0.01414	0.01988	0.02948	0.02273	0.02769	0.051	0.08497	0.1061	0.11962
735.6	0.01361	0.01951	0.02932	0.02242	0.02726	0.05	0.08364	0.10461	0.11799
736.36	0.01361	0.01938	0.02865	0.02183	0.02709	0.04944	0.08265	0.10283	0.11672
737.13	0.01345	0.01888	0.02823	0.02172	0.0266	0.04871	0.0815	0.10174	0.11516
737.89	0.01307	0.01856	0.0276	0.02106	0.02615	0.04759	0.08009	0.1004	0.11306
738.65	0.01295	0.01856	0.02755	0.02114	0.02575	0.04723	0.07949	0.09886	0.11158
739.42	0.01261	0.01788	0.02698	0.02058	0.02525	0.04632	0.07807	0.09722	0.11032
740.18	0.01267	0.01777	0.02649	0.02027	0.02483	0.04542	0.07681	0.0958	0.10837
740.94	0.01213	0.01771	0.02616	0.0198	0.02414	0.0446	0.07607	0.09503	0.10711
741.71	0.01207	0.01686	0.02546	0.01939	0.02397	0.04415	0.07458	0.09321	0.10509
742.47	0.01167	0.01678	0.02505	0.01903	0.0234	0.04307	0.07374	0.09141	0.10451
743.23	0.01173	0.01643	0.02472	0.01901	0.02291	0.0427	0.07253	0.09055	0.10224
744	0.01135	0.01621	0.02437	0.01835	0.02265	0.04178	0.07179	0.08919	0.101
744.76	0.01135	0.01615	0.02381	0.01797	0.02227	0.041	0.07057	0.0876	0.09853
745.52	0.01099	0.0155	0.02341	0.0177	0.02173	0.04081	0.06951	0.08678	0.09718
746.28	0.01079	0.01543	0.02319	0.01749	0.02152	0.03977	0.06883	0.0852	0.09596
747.05	0.01077	0.01472	0.02265	0.01724	0.0208	0.03891	0.06767	0.0842	0.09493
747.81	0.01019	0.01485	0.02242	0.01689	0.02055	0.03851	0.06676	0.08209	0.09286
748.57	0.00993	0.01437	0.02158	0.01676	0.02026	0.03756	0.06583	0.08088	0.09133
749.33	0.00991	0.0142	0.02137	0.01609	0.01979	0.03704	0.06468	0.08027	0.08999
750.09	0.00987	0.01402	0.02081	0.01585	0.01931	0.03642	0.0634	0.07863	0.08877
750.86	0.00968	0.01359	0.02037	0.01539	0.01924	0.03571	0.06308	0.07753	0.08784
751.62	0.00964	0.01359	0.02009	0.0155	0.0188	0.03553	0.062	0.07608	0.08613
752.38	0.00938	0.01314	0.01995	0.0151	0.01856	0.03456	0.06098	0.07505	0.08506
753.14	0.00926	0.01311	0.0196	0.01486	0.01795	0.03409	0.05997	0.07387	0.08371
753.9	0.00898	0.01288	0.01925	0.01471	0.01782	0.03375	0.0591	0.07297	0.08223

754.67	0.00892	0.01257	0.01893	0.01418	0.01752	0.0328	0.05795	0.07144	0.08107
755.43	0.00872	0.01246	0.01858	0.01406	0.01736	0.03226	0.05809	0.07075	0.08041
756.19	0.00828	0.01184	0.01833	0.0136	0.0169	0.03181	0.05631	0.06979	0.07884
756.95	0.00842	0.01197	0.0177	0.01357	0.01639	0.03135	0.05597	0.06893	0.0772
757.71	0.00816	0.0116	0.01739	0.01323	0.01624	0.03065	0.05478	0.06716	0.07617
758.47	0.00814	0.01147	0.01718	0.01306	0.01584	0.03021	0.05399	0.06648	0.0746
759.23	0.00788	0.01127	0.01697	0.01287	0.01572	0.02944	0.05317	0.06519	0.07319
759.99	0.0078	0.01092	0.01647	0.01239	0.01509	0.02894	0.0525	0.064	0.07197
760.76	0.0078	0.01077	0.01614	0.01254	0.01518	0.02858	0.05167	0.06326	0.07073
761.52	0.0075	0.01058	0.016	0.0121	0.01478	0.02793	0.05055	0.06214	0.06961
762.28	0.00738	0.01025	0.01551	0.01198	0.01459	0.02761	0.0496	0.06133	0.0688
763.04	0.00718	0.0101	0.0153	0.01172	0.01404	0.02677	0.04882	0.06006	0.06755
763.8	0.0071	0.01012	0.01484	0.01131	0.01404	0.02675	0.04825	0.05946	0.06683
764.56	0.00696	0.00984	0.01476	0.01128	0.01381	0.02612	0.04764	0.05799	0.06541
765.32	0.0068	0.00947	0.01444	0.01099	0.01347	0.0253	0.04687	0.0571	0.06453
766.08	0.00644	0.00941	0.01416	0.01089	0.01315	0.02532	0.04606	0.05631	0.06351
766.84	0.00646	0.00919	0.01365	0.01046	0.01305	0.02485	0.04523	0.05545	0.06308
767.6	0.00658	0.00897	0.01353	0.01039	0.0127	0.02412	0.04445	0.05453	0.06216
768.36	0.00632	0.00878	0.0133	0.01015	0.01255	0.02395	0.0444	0.05389	0.06091
769.12	0.00624	0.00858	0.01325	0.00997	0.0121	0.02328	0.04369	0.05319	0.05999
769.88	0.00636	0.00874	0.01283	0.00988	0.0121	0.02324	0.0428	0.05216	0.059
770.64	0.00592	0.00822	0.01276	0.00939	0.01182	0.02272	0.04266	0.05194	0.05853
771.4	0.00606	0.00837	0.01249	0.00962	0.01165	0.02229	0.04148	0.05107	0.05729
772.16	0.00581	0.008	0.0123	0.00939	0.01144	0.0221	0.04126	0.05038	0.05703
772.92	0.00575	0.00815	0.01205	0.00933	0.0113	0.02181	0.04049	0.04938	0.0565
773.68	0.00563	0.00796	0.0119	0.0091	0.01106	0.02136	0.0401	0.04898	0.05553
774.44	0.00551	0.00759	0.01169	0.00903	0.01081	0.02104	0.03976	0.04808	0.05478
775.2	0.00549	0.00752	0.01169	0.00879	0.01052	0.02098	0.03925	0.04727	0.05419
775.96	0.00539	0.00733	0.01126	0.00865	0.01042	0.02061	0.03836	0.04694	0.05309
776.72	0.00541	0.00733	0.01118	0.00858	0.01043	0.02024	0.03815	0.0462	0.05198
777.48	0.00521	0.00713	0.01095	0.00841	0.01005	0.01973	0.03785	0.04565	0.05209
778.24	0.00515	0.00709	0.01072	0.00812	0.01002	0.01943	0.03689	0.04473	0.05067
779	0.00499	0.007	0.01056	0.00794	0.00972	0.01927	0.0365	0.04411	0.05031
779.76	0.00501	0.007	0.0103	0.00792	0.00981	0.01889	0.0358	0.04324	0.04881
780.51	0.00479	0.00661	0.01025	0.00771	0.00936	0.0185	0.03532	0.04317	0.04883
781.27	0.00477	0.0065	0.00986	0.00759	0.00924	0.01805	0.03504	0.04225	0.04774
782.03	0.00481	0.00637	0.00995	0.00734	0.00908	0.01775	0.03417	0.04129	0.04743
782.79	0.00459	0.00635	0.00958	0.0073	0.00894	0.01742	0.03397	0.0408	0.04634
783.55	0.00451	0.00624	0.0094	0.0071	0.00886	0.01691	0.03344	0.04008	0.04574
784.31	0.00447	0.0059	0.00923	0.00689	0.00866	0.01701	0.03272	0.03958	0.04493
785.07	0.00441	0.00587	0.00916	0.00684	0.00835	0.01675	0.03233	0.03909	0.04418
785.82	0.00439	0.00566	0.00886	0.00671	0.0082	0.01609	0.03151	0.03837	0.04362
786.58	0.00409	0.00561	0.00868	0.00659	0.00808	0.01592	0.03139	0.03765	0.04285
787.34	0.00419	0.00564	0.0084	0.00654	0.00799	0.01578	0.03084	0.03694	0.04197

788.1	0.00395	0.00535	0.00823	0.00654	0.00766	0.01542	0.0304	0.03659	0.04165
788.86	0.00383	0.00533	0.00811	0.00614	0.0078	0.01508	0.03008	0.03616	0.04116
789.62	0.00375	0.00531	0.00793	0.00611	0.00752	0.0148	0.0292	0.03519	0.0406
790.37	0.00387	0.00522	0.00786	0.00597	0.0073	0.01473	0.02893	0.03471	0.03976
791.13	0.00377	0.00501	0.00777	0.00592	0.00723	0.01451	0.02886	0.03444	0.03861
791.89	0.00367	0.00494	0.00756	0.0057	0.00698	0.01421	0.02783	0.03341	0.03869
792.65	0.00359	0.00481	0.00747	0.00556	0.00702	0.01374	0.02789	0.03309	0.03777
793.4	0.00349	0.00475	0.00733	0.00548	0.00678	0.01352	0.02697	0.03249	0.03747
794.16	0.00345	0.00455	0.00681	0.00529	0.00659	0.01337	0.02675	0.03199	0.0364
794.92	0.00339	0.00468	0.00705	0.00553	0.00669	0.01344	0.02628	0.03159	0.0364
795.68	0.00323	0.00434	0.00684	0.00514	0.00627	0.01275	0.02601	0.031	0.03537
796.43	0.00325	0.00421	0.00661	0.00515	0.00613	0.01277	0.02557	0.03048	0.0348
797.19	0.00319	0.00414	0.00668	0.00502	0.00619	0.01227	0.0253	0.02997	0.03443
797.95	0.00311	0.00403	0.00647	0.00495	0.00589	0.01189	0.02477	0.02932	0.03357
798.7	0.00299	0.00408	0.00626	0.00469	0.00584	0.01187	0.02437	0.02916	0.03308
799.46	0.00295	0.00403	0.00586	0.00485	0.00577	0.0115	0.02405	0.02868	0.03272
800.22	0.00295	0.00386	0.00595	0.00459	0.00563	0.01157	0.02359	0.02835	0.03205
800.98	0.00285	0.00371	0.00577	0.00444	0.00553	0.01105	0.02339	0.02776	0.03162
801.73	0.00265	0.00375	0.00572	0.0044	0.00548	0.01101	0.02283	0.02695	0.0313
802.49	0.00275	0.00351	0.00565	0.00418	0.00518	0.0106	0.0227	0.0267	0.03075
803.25	0.00263	0.00349	0.00549	0.00423	0.00518	0.01062	0.02231	0.02646	0.03055
804	0.00267	0.0034	0.00528	0.0041	0.00509	0.01029	0.0218	0.02611	0.02959
804.76	0.00237	0.00334	0.00525	0.00394	0.00501	0.01023	0.02111	0.02552	0.02939
805.51	0.00253	0.00345	0.00516	0.00398	0.00484	0.00999	0.02115	0.02526	0.02886
806.27	0.00243	0.00314	0.00518	0.00389	0.00463	0.00969	0.0207	0.02436	0.02843
807.03	0.00225	0.00312	0.00482	0.00377	0.0047	0.00952	0.02021	0.02416	0.02798
807.78	0.00235	0.00306	0.00475	0.00372	0.00451	0.00947	0.0201	0.02379	0.02759
808.54	0.00235	0.00308	0.00461	0.00365	0.00449	0.00932	0.01971	0.02357	0.02698
809.29	0.00243	0.00295	0.00456	0.00362	0.00438	0.00909	0.01971	0.02324	0.02676
810.05	0.00217	0.00273	0.00451	0.00348	0.00433	0.00881	0.01936	0.02304	0.02616

Supplementary Figure 17

Relative pressure *P* /*P* 0	Uptake (cm^3·g^{-1}, STP)	Relative pressure *P* /*P* (	Uptake (cm^3·g^{-1}, STP)	Relative pressure *P* /*P* (	Uptake (cm^3·g^{-1}, STP)	Relative pressure *P* /*P* (	Uptake (cm^3·g^{-1}, STP)
0.0044	1.8501	0.95028	14.7273	0.00658	0.09146	0.99376	1.90844
0.00786	4.018	0.90519	15.2156	0.0116	0.11256	0.94257	1.00622
0.01197	6.1753	0.85495	15.6055	0.02324	0.13413	0.90639	0.84091
0.02293	8.1727	0.80527	15.9163	0.03475	0.1529	0.87128	0.7552
0.03358	8.7201	0.75544	16.1694	0.04005	0.15889	0.83611	0.68153
0.04556	9.0048	0.70554	16.3688	0.05794	0.18253	0.80115	0.63831
0.05714	9.2338	0.65484	16.5033	0.08541	0.21881	0.76596	0.60638
0.06043	9.3418	0.60583	16.5988	0.12039	0.26055	0.73104	0.57626
0.07033	9.4989	0.55508	16.6247	0.15529	0.29239	0.6959	0.55476
0.08014	9.6407	0.50488	16.6005	0.19023	0.31906	0.66089	0.53201
0.09045	9.7445	0.4748	16.5608	0.22518	0.34398	0.62587	0.515
0.10073	9.8087	0.4248	16.4811	0.2794	0.37586	0.59079	0.49012
0.11013	9.934	0.37477	16.3812	0.31432	0.39773	0.55579	0.46393
0.12015	10.0137	0.32496	16.2607	0.34927	0.40884	0.5209	0.44594
0.13019	10.0933	0.27503	16.1124	0.38415	0.41491	0.48591	0.40117
0.14009	10.1867	0.22488	15.9613	0.41916	0.43462	0.45078	0.37742
0.15065	10.2709	0.17512	15.7707	0.45424	0.44436	0.41577	0.35688
0.16072	10.3552	0.15004	15.6575	0.48916	0.4537	0.38076	0.34138
0.18009	10.453	0.12502	15.5478	0.52409	0.458	0.34575	0.31427
0.20038	10.56	0.10011	15.4418	0.55912	0.47914	0.3107	0.29298
0.22562	10.6771	0.08016	15.3256	0.59407	0.47994	0.27565	0.25991
0.24996	10.8024	0.06015	15.1712	0.62906	0.48935	0.24062	0.22254
0.29988	10.9528	0.05011	15.057	0.66404	0.49817	0.20561	0.19737
0.35043	11.1159	0.04005	14.949	0.69904	0.51787	0.17059	0.16947
0.39968	11.2951	0.03029	14.7577	0.734	0.52414	0.13559	0.12504
0.45051	11.4993	0.02019	14.4473	0.76902	0.54982	0.10216	0.09036
0.50051	11.7264	0.01024	13.4268	0.80391	0.58554	0.0724	0.06253
0.55044	11.9633			0.83897	0.6299	0.04267	0.01283
0.60048	12.2299			0.87389	0.68475		
0.65025	12.5305			0.9089	0.76945		
0.70055	12.8535			0.92527	0.83672		
0.75031	13.2042			0.95998	1.04168		
0.80042	13.566			0.99376	1.90844		
0.85034	13.9517						
0.9005	14.3325						
0.95028	14.7273						

Supplementary Figure 18

Relative pressure *P* /*P* 0	Uptake (cm^3·g^{-1}, STP)	Relative pressure *P* /*P* 0	Uptake (cm^3·g^{-1}, STP)	Relative pressure *P* /*P* (	Uptake (cm^3·g^{-1}, STP)	Relative pressure *P* /*P*	Uptake (cm^3·g^{-1}, STP)
3.59E-04	1.96428	0.94605	100.51759	0.004	0.29423	0.99436	3.81087
0.00169	4.49223	0.92969	101.1416	0.00615	0.37752	0.94219	2.21319
0.00353	7.04066	0.88128	101.86631	0.01009	0.43388	0.9065	1.91771
0.00679	8.78214	0.82565	101.68771	0.02327	0.49044	0.87131	1.76554
0.01089	9.71156	0.77572	101.2623	0.0349	0.50936	0.83621	1.66454
0.02094	11.222	0.7254	100.72814	0.04648	0.53405	0.8012	1.60672
0.02956	12.49038	0.67529	100.0931	0.05039	0.54176	0.76613	1.50598
0.04071	13.60864	0.62447	99.35107	0.08566	0.58491	0.73117	1.33467
0.04953	14.42625	0.57484	98.50496	0.1206	0.64816	0.71512	1.2325
0.06028	15.19999	0.52476	97.38345	0.17485	0.6953	0.68015	0.96908
0.06945	15.8202	0.47937	92.74258	0.2097	0.74502	0.62597	0.66391
0.08045	16.54341	0.44079	74.73543	0.24457	0.7672	0.59095	0.56934
0.09008	17.09493	0.4307	70.41144	0.2794	0.78149	0.55584	0.50483
0.10036	17.67736	0.4075	59.44505	0.31425	0.79191	0.52083	0.47201
0.10998	18.1691	0.38366	48.60644	0.34915	0.81455	0.48582	0.445
0.11993	18.69042	0.37492	44.92319	0.38427	0.80894	0.45079	0.39654
0.13023	19.22349	0.32858	36.86533	0.41922	0.79722	0.41572	0.34117
0.1403	19.63826	0.27268	34.24053	0.45425	0.79937	0.38088	0.33391
0.14995	20.08375	0.22421	30.47638	0.4891	0.79662	0.34582	0.34397
0.16	20.47548	0.17174	27.40212	0.5241	0.80762	0.31074	0.33575
0.18035	21.19371	0.15148	26.13285	0.55913	0.79288	0.27572	0.31039
0.20088	21.84341	0.12523	24.25979	0.59404	0.80661	0.24064	0.28036
0.22537	22.57394	0.09987	22.38935	0.62914	0.8131	0.20562	0.26612
0.25102	23.26727	0.07999	20.95077	0.66398	0.82937	0.17059	0.23223
0.29989	24.29908	0.0605	19.29326	0.69903	0.89168	0.13559	0.20505
0.35112	25.33819	0.04992	18.29744	0.73396	0.98308	0.10215	0.11056
0.40058	26.17049	0.04012	17.28274	0.76901	1.13709	0.07236	0.02381
0.44934	26.95525	0.03021	16.09871	0.80391	1.34237	0.04264	-0.0545
0.50076	27.64564	0.0203	14.60537	0.81993	1.45446		
0.55036	28.26606	0.00982	12.41045	0.85503	1.67516		
0.60049	28.85299			0.89001	1.79467		
0.65026	29.38185			0.94376	2.09255		
0.70052	29.89488			0.96	2.30119		
0.75042	30.39761			0.99436	3.81087		
0.80049	30.92246						
0.82878	52.39248						
0.84931	59.7437						
0.88379	77.87087						
0.89639	80.41749						
0.93223	98.91547						
0.94605	100.51759						

Supplementary Figure 19

Relative pressure *P* /*P* 0	Uptake ($cm^3·g^{-1}$, STP)	Relative pressure *P* /*P* 0	Uptake ($cm^3·g^{-1}$, STP)	Relative pressure *P* /*P* 0	Uptake ($cm^3·g^{-1}$, STP)	Relative pressure *P* /*P* 0	Uptake ($cm^3·g^{-1}$, STP)
9.16E-03	1.35682	0.95555	91.69054	0.00662	0.12119	0.9931	2.82635
0.01963	2.25493	0.90602	91.63448	0.01162	0.14302	0.94315	1.09259
0.021	3.1929	0.85651	91.36891	0.02324	0.18073	0.90643	0.89418
0.03234	3.78251	0.80711	90.96701	0.03063	0.20566	0.8712	0.81941
0.04466	4.33168	0.77521	90.64417	0.04041	0.21825	0.83613	0.75272
0.04987	4.579	0.7242	90.11599	0.05794	0.25431	0.80107	0.70165
0.06114	4.85094	0.67462	89.51834	0.0854	0.30121	0.76603	0.65875
0.07025	5.03973	0.62453	88.80428	0.1204	0.33679	0.73092	0.62037
0.07973	5.35256	0.5756	87.99789	0.15531	0.37173	0.69597	0.5851
0.09026	5.49583	0.52478	86.96303	0.20958	0.41169	0.66087	0.55921
0.10059	5.65721	0.4755	85.36795	0.24452	0.43035	0.62589	0.5309
0.10996	5.88999	0.44145	73.79266	0.27941	0.46746	0.59079	0.52976
0.12027	5.95835	0.43204	62.59908	0.31429	0.48209	0.55574	0.50629
0.1304	6.12535	0.42409	54.41107	0.34922	0.50344	0.52094	0.49127
0.14026	6.40376	0.40986	43.49278	0.38412	0.52352	0.48572	0.47704
0.14968	6.60122	0.38416	33.0633	0.41907	0.52554	0.45082	0.44004
0.15989	6.871	0.36138	30.69477	0.45419	0.53825	0.41579	0.42638
0.17998	7.02396	0.31231	28.0372	0.48914	0.56227	0.38075	0.40421
0.19984	7.40334	0.26227	25.43853	0.52408	0.59113	0.34575	0.38525
0.22508	7.68457	0.22693	22.9186	0.55907	0.58892	0.31066	0.36293
0.25019	8.20359	0.17198	20.16983	0.59402	0.60197	0.27568	0.32246
0.29941	9.41858	0.13554	18.26598	0.62908	0.61911	0.24063	0.28419
0.34929	10.53406	0.12509	17.63934	0.66402	0.61091	0.2056	0.23274
0.3993	11.71979	0.09965	16.11003	0.69905	0.62317	0.17058	0.19977
0.44965	13.00247	0.08032	14.85418	0.73403	0.63142	0.13562	0.15765
0.5003	14.50011	0.06052	13.30241	0.76894	0.66865	0.10214	0.11892
0.54862	16.90699	0.04983	12.32782	0.80399	0.67952	0.07237	0.06944
0.6051	18.75212	0.03995	11.36784	0.83898	0.72254	0.04264	0.01349
0.64851	19.91914	0.02993	10.42739	0.87391	0.77008		
0.70044	20.93485	0.02049	9.4048	0.90894	0.86679		
0.74965	21.80662			0.92506	0.91675		
0.80042	22.62558			0.95985	1.14263		
0.84987	23.52457			0.9931	2.82635		
0.86595	43.63142						
0.8799	59.82056						
0.89759	69.33898						
0.92878	84.66794						
0.95555	91.69054						

Supplementary Figure 20

Relative pressure *P* /*P* (	Uptake ($cm^3 \cdot g^{-1}$, STP)	Relative pressure *P* /*P* (	Uptake ($cm^3 \cdot g^{-1}$, STP)	Relative pressure *P* /*P* (	Uptake ($cm^3 \cdot g^{-1}$, STP)	Relative pressure *P* /*P* (	Uptake ($cm^3 \cdot g^{-1}$, STP)
0.00146	1.79439	0.95027	43.62713	0.00199	0.17071	0.99484	4.78709
0.00366	3.0497	0.92484	45.09642	0.00374	0.28153	0.9418	3.22137
0.00626	3.94427	0.87398	46.27475	0.00523	0.37784	0.90637	2.85754
0.00855	4.40997	0.82558	46.69084	0.0101	0.45546	0.87128	2.68652
0.01001	4.68052	0.77778	46.84168	0.02326	0.57469	0.83616	2.57814
0.01946	6.31493	0.73123	46.86113	0.03494	0.62555	0.8011	2.44402
0.03062	7.85488	0.67464	46.66913	0.04643	0.73135	0.76596	2.52812
0.03809	18.31981	0.62485	46.38619	0.05043	0.74089	0.73106	2.31727
0.05135	22.38249	0.57436	45.98402	0.08566	0.89525	0.696	2.10581
0.05956	23.35304	0.52454	45.51483	0.12057	0.95952	0.66092	1.96689
0.0706	24.19752	0.47488	44.93636	0.15549	1.06383	0.62589	1.91842
0.08069	24.77582	0.42451	44.23801	0.2097	1.2198	0.59088	1.84461
0.0906	25.31976	0.3749	43.36949	0.24455	1.32103	0.55583	1.76536
0.10016	25.79324	0.32498	42.28398	0.2794	1.39672	0.52092	1.69725
0.11055	26.27728	0.27492	40.98373	0.3143	1.42351	0.4858	1.56961
0.12009	26.73764	0.22511	39.33347	0.34923	1.54605	0.45091	1.48815
0.13013	27.14422	0.17502	37.30845	0.38424	1.55571	0.41585	1.39323
0.1405	27.56466	0.14993	36.13145	0.41918	1.6491	0.38081	1.35184
0.15009	27.95438	0.12508	34.77226	0.4542	1.7365	0.34578	1.22817
0.16015	28.28018	0.10013	33.14383	0.48917	1.75036	0.31076	1.1308
0.18059	28.91933	0.08003	31.66613	0.52411	1.76898	0.27571	0.99775
0.2002	29.50281	0.06029	29.83667	0.55906	1.84321	0.24066	0.85539
0.22585	30.17349	0.05009	28.56104	0.5941	1.82639	0.20561	0.78627
0.25088	30.84162	0.04002	27.13718	0.62902	1.83822	0.17064	0.61009
0.30051	31.9295	0.03021	25.48187	0.66407	1.87175	0.13562	0.42747
0.3503	32.96034	0.02041	23.12122	0.69898	1.93915	0.10219	0.281
0.3996	33.96338			0.73401	2.01818	0.07241	0.13738
0.45066	34.88319			0.76893	2.11192	0.04263	-0.06829
0.50049	35.83305			0.80395	2.23954		
0.55063	36.70392			0.83891	2.37787		
0.60053	37.60658			0.87399	2.43369		
0.65057	38.49748			0.90882	2.53277		
0.70035	39.36952			0.94389	2.8221		
0.75058	40.24044			0.97881	3.47621		
0.80027	41.08385			0.99484	4.78709		
0.85056	41.94768						
0.90036	42.79087						
0.95027	43.62713						
